# Hexaphenyl-1,2-Diphosphonium Dication [Ph_3_P–PPh_3_]^2+^: Superacid, Superoxidant, or Super Reagent?

**DOI:** 10.1021/jacs.5c01271

**Published:** 2025-04-24

**Authors:** Fabian Dankert, Simon P. Muhm, Chandan Nandi, Sergi Danés, Sneha Mullassery, Petra Herbeck-Engel, Bernd Morgenstern, Robert Weiss, Pedro Salvador, Dominik Munz

**Affiliations:** † Coordination Chemistry, Saarland University, Campus C4.1, D-66123 Saarbrücken, Germany; ‡ Institut de Química Computacional I Catàlisi, Departament de Química, Universitat de Girona, C/M. Aurelia Capmany 69, 17003 Girona, Spain; § INM Leibniz Institute for New Materials, Campus D2.2, 66123 Saarbrücken, Germany; ∥ Inorganic Solid-State Chemistry, Saarland University, Campus C4.1, D-66123 Saarbrücken, Germany; ⊥ Friedrich-Alexander-Universität (FAU) Erlangen-Nürnberg, Nikolaus-Fiebiger-Str. 10, 91058 Erlangen, Germany

## Abstract

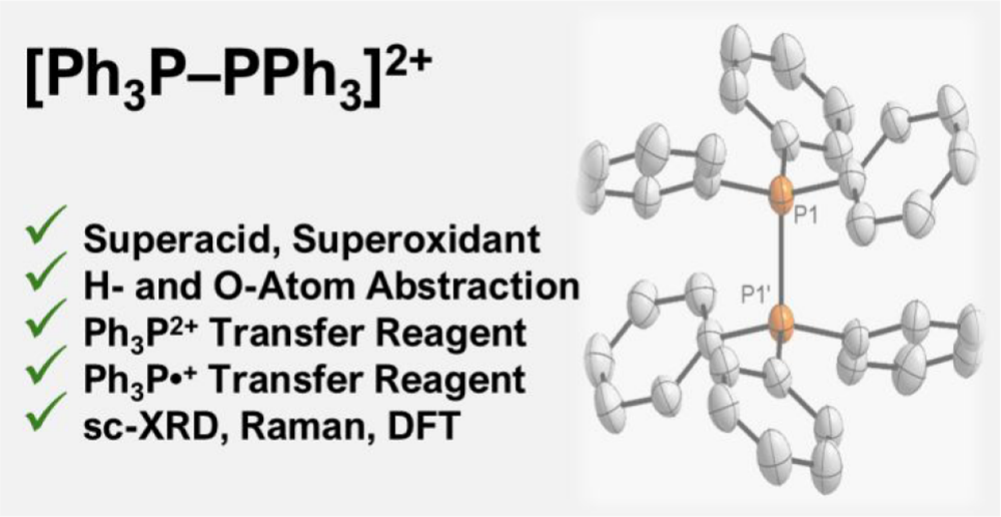

The oxidation of triphenylphosphine by perfluorinated phenazinium^F^ aluminate in difluorobenzene affords hexaaryl-1,2-diphosphonium dialuminate **1**. Dication **1**^**2+**^ is valence isoelectronic with elusive hexaphenylethane, where instead the formation of a mixture of the trityl radical and Gomberg’s dimer is favored. Quantum-chemical calculations in combination with Raman/IR spectroscopies rationalize the stability of the P–P bonded dimer in **1**^**2+**^ and suggest, akin to the halogens, facile homolytic as well as heterolytic scission. Thus, **1**^**2+**^ serves as a surrogate of both the triphenylphosphorandiylium dication (Ph_3_P^2+^) and the triphenylphosphine radical monocation (Ph_3_P^·+^). Treating **1** with dimethylaminopyridine (DMAP) or *^t^*Bu_3_P replaces triphenylphosphine under heterolytic P–P bond scission. Qualifying as a superoxidant (*E* vs Fc/Fc^+^ = +1.44 V), **1** oxidizes trimethylphosphine. Based on halide abstraction experiments (**^–^**BF_4_, **^–^**PF_6_, **^–^**SbCl_6_, **^–^**SbF_6_) as well as the deoxygenation of triethylphosphine oxide, triflate anions as well as toluic acid, **1** also features Lewis superacidity. The controlled hydrolysis affords Hendrickson’s reagent, which itself finds broad use as a dehydration agent. Formally, homolytic P–P bond scission occurs with diphenyldisulfide (PhSSPh) and the triple bonds in benzo- and acetonitrile. The irradiation by light cleaves the P–P bond homolytically and generates transient triphenylphosphine radical cations, which engage in H-atom abstraction as well as CH phosphoranylation.

## Introduction

**Scheme 1:**
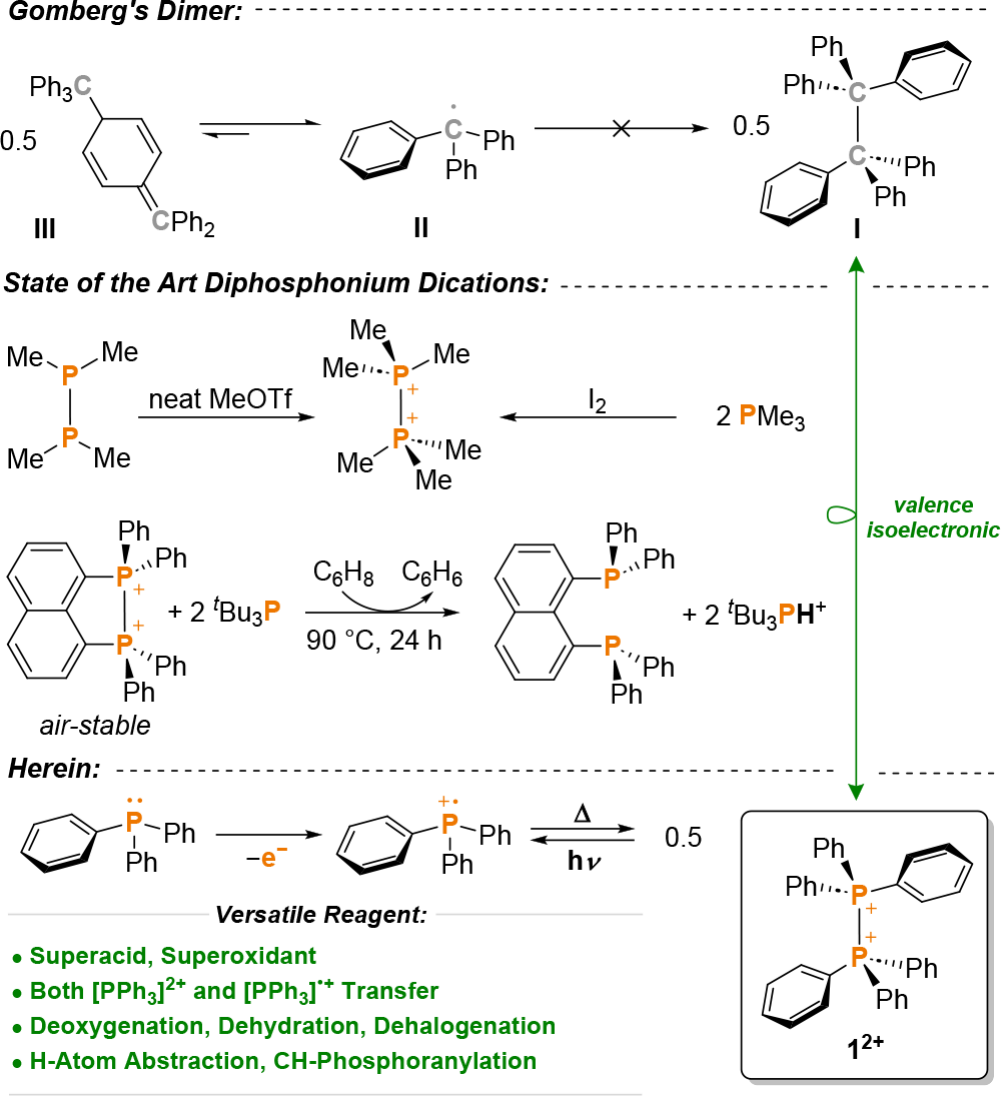
Triphenylmethyl Radicals Associate to Gomberg’s Dimer (Top), yet the Isovalence Electronic Triphenylphosphine Radical Cation Affords the Hexaphenyl-1,2-diphosphonium Dication (Bottom)

In the quest for hexaphenylethane (Scheme 1, **I**), Moses Gomberg reported the triphenylmethyl (trityl) radical **II** in 1900.^1^ The discovery of “trivalent carbon” is considered nowadays the beginning of organic radical chemistry. Later scrutiny revealed that the trityl radical had been obtained only in marginal yield, as it dimerizes instead through addition to the *para*-position of one phenyl substituent (Gomberg’s dimer **III**).^2^ Whereas heavier hexaphenylsilaethane is isolable,^3^ corresponding addition reactions to the *para*-position also occur for the valence isoelectronic triphenylboryl radical anion^4^ and the triphenylamine radical cation.^5^ In the 1970s, it was found that the decoration of the phenyl substituents shifts the equilibrium to respective hexaarylethane dimers.^6^ Phenyl group decoration similarly allows for a shift of the equilibrium toward monocationic triarylamine radical monomers^7^ and dianionic diboron(6) dimers.^8^ The adduct of triphenylphosphine with the trityl cation [Ph_3_P–CPh_3_]^+^ is isolable, yet the isomer derived from *para*-addition is thermodynamically more stable.^9^

In stark contrast, both the hexaphenyl-1,2-diphosphonium dication as well as the triphenylphosphine radical cation remain elusive.^10^ Triphenylphosphine radical cations are believed to serve as transient key intermediates in (photo)redox catalysis,^11^ as well as even arene hydrogenation by water.^12^ It is known furthermore that the sterically encumbered trimesitylphosphine radical cation does not dimerize.^13^ This radical may be obtained in crystalline form upon treating (Mes)_3_P with Al(C_6_F_5_)_3_,^13b^ thus bridging the fields of Frustrated Lewis Pairs (FLPs)^14^ and radical chemistry.^15^ Another phosphine radical cation was stabilized thanks to three bulky halogenated naphthyl substituents, where the halogen atoms directly interact with the radical site.^16^ The treatment of red phosphorus by primary alkyl iodides^17^ as well as the permethylation of diphosphines R_2_P–PR_2_ by an excess of methyl triflate (R = Me) affords respective dimers, namely peraliphatic ^+^P–P^+^ vicinal diphosphonium dications.^18^ The [Me_3_P–PMe_3_]^2+^ dication has been the subject of various studies, including its generation through oxidation of pyrophoric PMe_3_ by electrochemistry^19^ or iodine,^20^ heavy group 15 oxidants such as Sb(V),^21^ and copper(II)^20^ complexes. The electronic structure of the unusual vicinal dicationic^22^^+^P–P^+^ bond has been investigated computationally and spectroscopically.^23^ It has been argued (i) that the ^+^P–P^+^ bond is more susceptible to homolytic than heterolytic cleavage and (ii) that the vicinal cationic charges infer covalency. Although known since the 1980s and in stark contrast to transient triphenylphosphine radical cations (*vide supra*), these peraliphatic dications have not yet found application in catalysis or organic synthesis. It was reported, however, that they readily react with nucleophiles such as water, thiols, dipropyl disulfide, and secondary amines, yet not with aromatic CH bonds or olefins.^19,24^

Enforcing two phosphorus centers into proximity by a rigid bridge represents an alternative approach to 1,2-diphosphonium dications.^25^ Treating the naphthalene-linked P^V^/P^III^ precursor (Ph_2_F_2_P)(C_10_H_6_)(PPh_2_) with the triethylsilylium cation afforded the respective ^+^P–P^+^ dication. This compound is persistent in the air and shows a moderate acceptor number (AN) of 30.^26^ The addition of the Lewis base P*^t^*Bu_3_ generates a powerful FLP of high hydridophilicity, as was demonstrated by hydride abstraction from triethylsilane (24 h, room temperature) and even the C–H dehydrogenation of cyclohexadiene (24 h, 90 °C). Encouraged by the bond-activation chemistry by formal phosphorus dications,^27^ constrained phosphenium monocations,^28^ the emergence of organopnictogen redox catalysis,^29^ as well as reports on formal carbon dications,^30^ we turned our attention toward oxidizing triphenylphosphine by an “innocent” oxidant. The fluorinated phenazinium radical cations reported by the Krossing group appeared to be a promising choice,^31^ as they are prepared with aluminate anions.^32^ Here, we report that triphenylphosphine is swiftly oxidized by such “deelectronators”, thereby affording the hexaaryl-1,2-diphosphonium dication **1**^**2+**^. Spectroscopic as well as computational investigations rationalize the stability of **1**^**2+**^ with respect to Gomberg’s dimer. Reactivity studies demonstrate (i) both homo- and heterolytic cleavage of the P–P bond, (ii) Lewis (super)acidity, (iii) H-atom abstraction capabilities, and (iv) that dication **1**^**2+**^ serves as a powerful deoxygenation and phosphoranylation agent.

## Results and Discussion

**Scheme 2:**
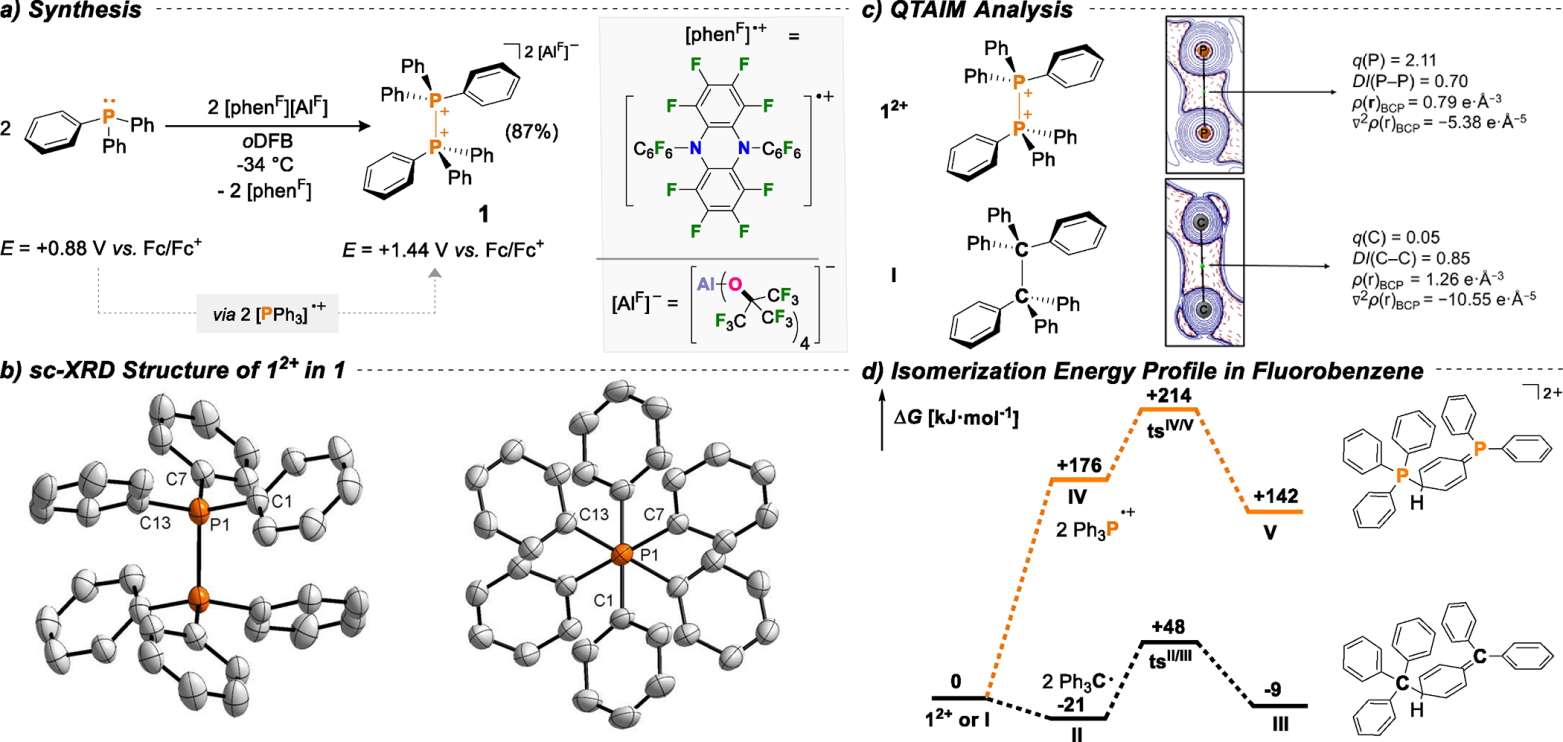
(a) Synthesis of **1**; (b) sc-XRD Structure of **1^2+^** in Single-Crystals of 1·C_6_H_2_F_4_; (c) QTAIM Analysis of **1^2+^** and I, Ph_3_C–CPh_3_; Blue Lines Indicate Charge Depletion (∇^2^ρ(*r*) > 0), Red Dashed Lines Show Charge Concentration (∇^2^ρ(*r*) < 0); the Solid Black Lines Are Bond Paths, the Green Dots Are Bond Critical Points (BCP); *q,* Partial Charges; DI, Delocalization Indices; ∇^2^ρ(*r*)_BCP_, Laplacian at the BCP; ρ(*r*)_BCP_, Electron Density at the BCP; (d) Energy Profile for the Formation of Gomberg’s-dimer at the CPCM(PhF)-PBE0-D3/def2-TZVPP//PBE0-D3/def2-SVP Level of Theory Anions and cocrystallized C_6_H_2_F_4_ are omitted for clarity, thermal ellipsoids are given at 50% probability. Selected bond lengths [Å] and angles [°] in **1**: P1–P1, 2.262(3); P1–C1, 1.794(4); P1–C7, 1.786(4); P1–C13, 1.794(4); C1–P1–C7, 111.5(2); C7–P1–C13, 113.2(2); C1–P1–C13, 112.2(2); C13–P1–P1, 105.8(2); C7–P1–P1, 106.6(2); C13–P1–P1, 105.8(2); C13–P1–P1-C13, 180°.

Treating triphenylphosphine in cold *ortho*-difluorobenzene (*o*DFB) or 1,2,3,4-tetrafluorobenzene (TFB) with two equivalents of [phen^F^][Al^F^] ([phen^F^] = [perfluoro-5,10-bis(perfluorophenyl)-5,10-dihydrophenazinium]^·+^; [Al^F^] = [Al{OC(CF_3_)_3_}_4_]^−^) or [ant^Cl,F^][Al^F^] (see SI) afforded a faint yellow solution (Scheme 2a). A crystalline, colorless precipitate formed upon slow warming of the solution to room temperature. The precipitate was identified as **1** according to the ^31^P NMR spectroscopic analysis with a signal at 17.6 ppm (c.f*.* [Me_3_P–PMe_3_][OTf]_2_ 28.4 ppm),^18^ and was isolated in 87% crystalline yield after workup. Compound **1** is thermally robust, with decomposition starting around +260 °C (Figure S18). Single-crystal X-ray diffraction (sc-XRD) confirmed the formation of hexaphenyl-1,2-diphosphonium dialuminate [Ph_3_P–PPh_3_][Al^F^]_2_ (**1**, Scheme 2b). In the solid-state structure, an inversion center is found between the two phosphorus atoms, which implies a staggered conformation of the phenyl groups. Although the crystallographic data are of moderate quality (see SI for details), they enabled the determination of reasonably reliable structural metrics for the dication in **1**. The P–P bond of 2.262(3) Å is elongated in comparison to the PMe_3_-congener (2.198(2) Å).^18^ The comparison with triphenylphosphine reveals that the P–C bonds in **1** are shorter (**1**, 1.794(4), 1.786(4), 1.794(4) Å; PPh_3_, ∼1.823 Å) and that the C–P–C angles (**1**, 111.5(2), 113.2(2), 112.2(2)°; PPh_3_, ∼102°) are larger, thus indicating significant planarization and charge-delocalization.

The Raman (IR, respectively; Figures S15–S17) vibrational spectroscopy revealed bands at  = 191 and 611 cm^**–**1^, and a weak feature at 450 cm^**–**1^. Based on computations (*vide infra*), we assign these signals to the P–P stretch, which couples with phenyl-based modes ( = 202, 449, and 581 cm^–1^; Figure S125). Whereas it has been argued^3b,23b^ that neither the P–P bond length nor corresponding stretching frequency (Badger’s and Gordy’s rules) are an accurate measure for the P–P bond’s strength, we note that these values are similar to the ones reported for the diphosphonium dication in [Me_3_P–PMe_3_][OTf]_2_ ( = 207, 456, 692 cm^**–**1^;  = 212, 444, 670 cm^**–**1^; Figure S126). The cyclovoltammetric analysis in pentafluorobenzene (Figure S110) revealed that dimeric **1** is a potent oxidant with a redox potential of *E* = +1.44 V vs Fc/Fc^+^, which exceeds that of monomeric PPh_3_ (*E* = ∼0.88 V vs Fc/Fc^+^) by almost 0.6 V.

Quantum chemical calculations at the density functional theory (DFT) level were performed to understand the stability of **1**^**2+**^. The topological analysis of the electron density (QTAIM; Scheme 2c) affords a bond critical point (bcp) connecting the two phosphorus atoms. The negative sign of the Laplacian at the bcp (∇^2^ρ(*r*_bcp_) = −5.38 e Å^–5^) and the bond order (DI = 0.70) are consistent with a covalent interaction and local charge accumulation, coupled to charge depletion in the area opposed to the P–P bond. The QTAIM descriptors are similar to those reported for [Me_3_P–PMe_3_]^2+^ (ρ(*r*_bcp_) = 0.82 e Å^–3^; ∇^2^ρ(*r*_bcp_) = −4.45 e Å^–5^), yet enhanced covalency is found for carbon-congener **I** (ρ(*r*_bcp_) = 1.26 e Å^–3^; ∇^2^ρ(*r*_bcp_) = −10.55 e Å^–5^; DI = 0.85). Energy Decomposition Analysis coupled with Natural Orbitals for Chemical Valence (EDA-NOCV^33^; Tables S5–S10) was performed to understand the nature of the P–P bond in **1**^**2+**^ in the gas phase and to put it into context with related molecules. The interaction energy in **1**^**2+**^ (Δ*E*_int_ = −122 kJ mol^–1^) profits from a high dispersion contribution (Δ*E*_disp_ = −80 kJ mol^–1^). Dispersion energy is virtually the same for Ph_3_C–CPh_3_ (Δ*E*_disp_ = −81 kJ mol^–1^; Δ*E*_int_ = −49 kJ mol^–1^) despite the difference of the C–C and P–P distances, yet it is smaller for [Me_3_P–PMe_3_]^2+^ (Δ*E*_disp_= −27 kJ mol^–1^). We note that the electrostatic contribution largely balances with Pauli repulsion (**1**^**2+**^, Δ*E*_Pauli_ + Δ*E*_elstat_ = (792–206) = +586 kJ mol^–1^; [Me_3_P–PMe_3_]^2+^, Δ*E*_Pauli_ + Δ*E*_elstat_ = (543 + 7) = +551 kJ mol^–1^), just as is the case for the σ-interaction (**1**^**2+**^, Δ*E*_orb-σ_ = −483 kJ mol^–1^; [Me_3_P–PMe_3_]^2+^, Δ*E*_orb-σ_ = −486 kJ mol^–1^).

Hence, the enhanced gas-phase stability of **1**^**2+**^ (−*D*_e_ = −87 kJ mol^–1^) in respect to [Me_3_P–PMe_3_]^2+^ (−*D*_e_ = −19 kJ mol^–1^; Δ*E*_int_ = −42 kJ mol^–1^) is essentially due to dispersive stabilization (*vide supra*) as well as collective secondary orbital interactions (Figures S129 and S130) with the phosphines’ substituents (**1**^**2+**^, Δ*E*_orb-rest_ = −145 kJ mol^–1^; [Me_3_P–PMe_3_]^2+^, Δ*E*_Orb-rest_ = −80 kJ mol^–1^).

Bickelhaupt and colleagues computationally compared the C–C bond in Ph_3_C–CPh_3_ with the Si–Si bond in Ph_3_Si–SiPh_3_.^34^ As both molecules’ Δ*E*_int_ values are comparable, it was suggested that the dissociation of Ph_3_C–CPh_3_ is more facile than that of Ph_3_Si–SiPh_3_ due to the much higher strain *viz.* preparation energy penalty (ΔΔ*E*_prep_ = +267 kJ mol^–1^).^34^ The strain energy in **1**^**2+**^ is likewise much smaller (Δ*E*_prep_ = +34 kJ mol^–1^) compared to that of Ph_3_C–CPh_3_ (Δ*E*_prep_ = +329 kJ mol^–1^). We thus conclude that the P–P bond in **1**^**2+**^ (−*D*_e_ = −87 kJ mol^–1^) is weaker than the Si–Si bond in Ph_3_Si–SiPh_3_ due to electrostatics, but stronger than the C–C bond in Ph_3_C–CPh_3_ (−*D*_e_ = −49 kJ mol^–1^) due to the reduced preparation energy. Indeed, [Ph_3_N–NPh_3_]^2+^ is thermodynamically not stable, as it suffers from both a large preparation energy as well as electrostatic repulsion between two formally cationic charged nitrogen atoms with a short N–N distance (Figure S128). In fact, monocationic [Ph_3_P–PPh_2_]^+^ and neutral Ph_2_P–PPh_2_ show only minor changes in the optimized P–P bond lengths with respect to the dication **1**^**2+**^. However, these compounds feature strong P–P bonds ([Ph_3_P–PPh_2_]^+^, –*D*_e_ = −309 kJ mol^–1^; [Ph_2_P–PPh_2_],–*D*_e_ = −226 kJ mol^–1^), mostly attributable to more favorable electrostatics Δ*E*_elstat_ (Table S5).

We furthermore computed the free-energy profile for the formation of Gomberg’s dimer **III** and the corresponding P-congener **V** in fluorobenzene solution (Scheme 2d). Solvation renders the homolytic P–P bond cleavage in **1**^**2+**^ stronger endergonic (**IV**, Δ*G* = +176 kJ mol^–1^) than in the gas phase, where this reaction is facile (Figure S118; Δ*G*_gas_ = +33 kJ mol^–1^). As dearomatization is not coupled to efficient charge-delocalization, addition product **V** is also very high in energy (Δ*G* = +142 kJ mol^–1^). Opposed to **1**^**2+**^, solvation does not play a crucial role for the dissociation of neutral **I** into trityl radicals **II** (Δ*G* = −21 kJ mol^–1^, Figure S119; Δ*G*_gas_ = −14 kJ mol^–1^; Figure S120) and neither for the further transformation to Gomberg’s dimer **III** (Δ*G* = −9 kJ mol^–1^, Figure S119; Δ*G*_gas_ = −5 kJ mol^–1^, Figure S120), which profits from the generation of a strong C=C double bond.

**Scheme 3:**
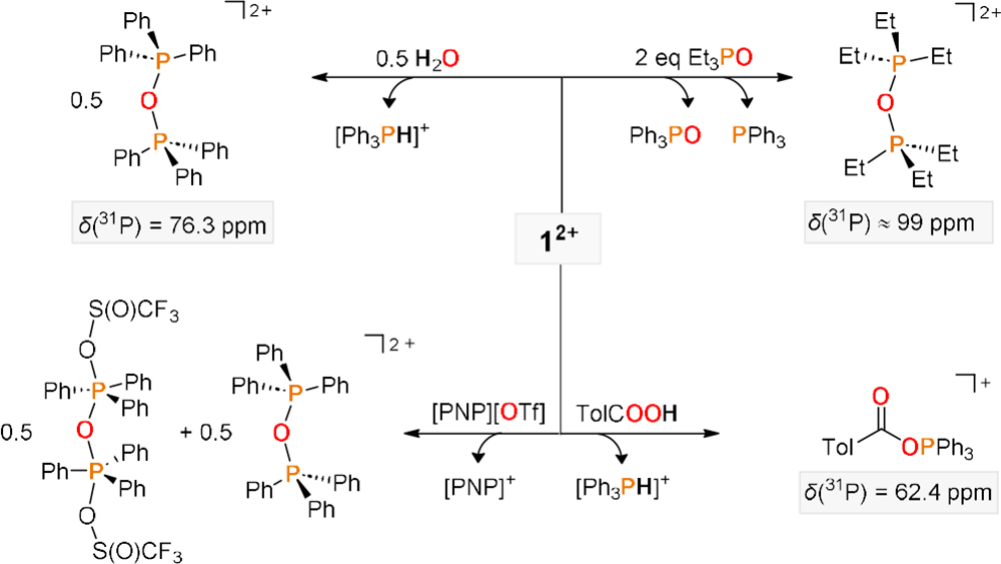
Deoxygenation of Et_3_PO, Water, *p*-Toluic Acid, and [PNP][OTf] by **1^2+^**; [Al^F^] Anions Are Omitted for Clarity; All Reactions Were Conducted in *o*DFB at Ambient Temperature with Quantitative Conversion upon Mixing

Efforts were conducted to gauge the Lewis-acidity of **1** (Ph_3_P^2+^, respectively),^35^ as computations predict exceedingly strong *Fluoride Ion Abstraction* (FIA = 1237 kJ mol^–1^; FIA_solv_ = 511 kJ mol^–1^) capabilities as well as a high *Hydride Ion Affinity* (HIA = 1318 kJ mol^–1^; HIA_solv_ = 574 kJ mol^–1^; Table S12). Indeed, both the addition of *para*-fluorobenzonitrile^36^ and triethylphosphine oxide (Gutmann–Beckett method)^37^ proved unproductive. Dication **1**^**2+**^ adds to nitriles under P–P bond cleavage (*vide infra*) instead of forming stable adducts, whereas triethylphosphine oxide leads to oxygen transfer, thereby generating a stoichiometric mixture of triphenylphosphine oxide, triphenylphosphine, and [Et_3_P–O–PEt_3_]^2+^ (Scheme 3).

Consequently, we also explored the reaction with other oxygen-containing molecules such as water,^12^ a triflate salt, and *p*-toluic acid. The controlled partial hydrolysis cleanly generated the aluminate analogue of Hendrickson’s reagent [(Ph_3_P)_2_O][OTf]_2_ (“POP”)^38^ and a stoichiometric amount of triphenylphosphonium salt [Ph_3_PH]^+^. This reaction is remarkable, as Hendrickson’s reagent itself finds use for challenging dehydration reactions.^39^ In fact, and highlighting exceeding oxophilicity, we observed the abstraction of oxygen atoms upon adding the triflate salt [PNP][OTf] (PNP = [Ph_3_PNPPh_3_]^+^) to **1**.^40^ The reaction with *p*-toluic acid generated the respective acyloxyphosphonium salt, which serves as the activated mixed-anhydride intermediate in the Appel^41^ and Mitsunobu^42^ reactions.

**Scheme 4:**
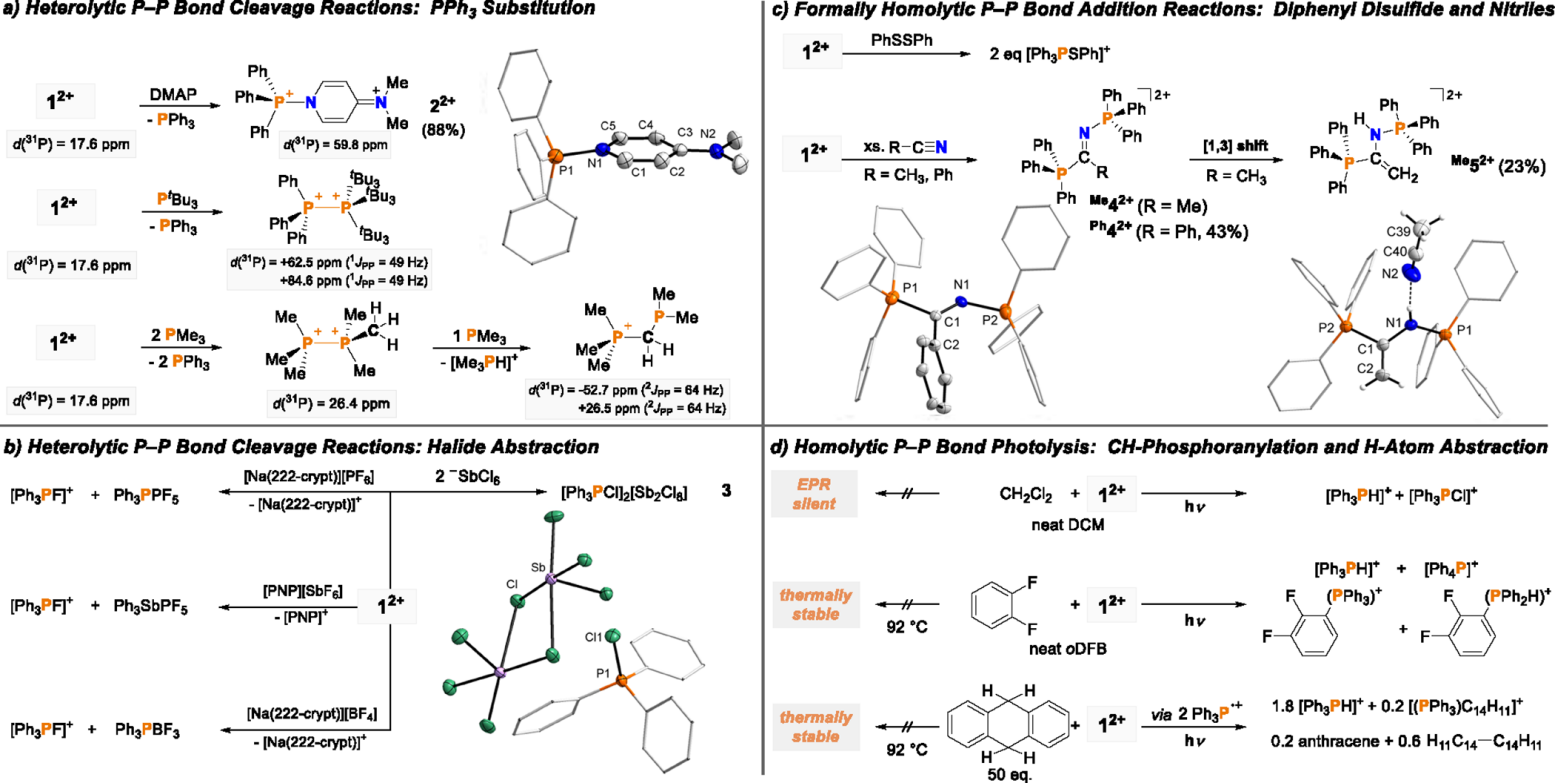
Heterolytic (a, b) and Homolytic (c, d) Bond Cleavage Reactions of **1**; [Al^F^] Anions Are Omitted for Clarity All Reactions were performed in *o*DFB and proceeded quantitatively. Most hydrogen atoms within crystal structure representations are omitted for clarity, thermal ellipsoids are given at 50% probability. Only one of four independent [PPh_3_Cl]^+^ moieties and a symmetry-generated part of the anion in **3** are shown for clarity (see ESI for details). Selected bond lengths [Å] for 2^2+^: P1–N1, 1.719(2); N1–C1, 1.384(4); C1–C2, 1.339(4); C2–C3, 1.423(4); C4–C5, 1.329(4); C3–C4, 1.433(4); C3–N2, 1.320(4). See SI for further metrics.

Further detailed reactivity studies were conducted to gauge the interplay of heterolytic *versus* homolytic P–P bond cleavage. Dimethylaminopyridine (DMAP) readily replaces triphenylphosphine in *o*DFB solution at room temperature, thereby generating P(V) and P(III) products under heterolytic P–P bond cleavage (**2**, Scheme 4a). sc-XRD crystallography unambiguously confirmed the structure of **2**,^43^ where both the P–N (1.719(2) Å) as well as C=NMe_2_ (1.320(4) Å) bond lengths in the solid state are consistent with the values found for [Me_3_P(DMAP)] [OTf]_2_ (P–N, 1.720(3); C=NMe_2_ 1.319(4) Å),^24^ thus indicating pronounced delocalization of cationic charge onto the DMAP substituent. Note that the equilibrium lies in the case of [Me_3_P(DMAP)][OTf]_2_ at the side of [Me_3_P–PMe_3_][OTf]_2_. The substitution of PPh_3_ proceeds according to quantum-chemical calculations barrierless through an S_N_2-type mechanism (Figure S131), thereby highlighting the Lewis acidity of **1**. We analyzed the P–P bond polarization along the reaction coordinate by the occupations of σ-type effective fragment orbitals (EFOs)^44^ of the triphenylphosphine fragments (Figure S132).^45^ DMAP polarizes the P–P bond already at a long distance (*d*_P–N_ = 2.931 Å), rendering the former **1**^**2+**^ effectively a triphenylphosphine-stabilized triphenylphosphorandiylium dication.

The corresponding substitution reaction to the mixed biscationic diphosphonium dication [Ph_3_P–P*^t^*Bu_3_]^2+^ and PPh_3_ was obtained in the reaction with P*^t^*Bu_3_. The homodimer [Me_3_P–PMe_3_]^2+^ is formed upon treatment with trimethylphosphine, which (i) indicates that **1**^**2+**^ is a stronger oxidant (less stable, respectively) than [Me_3_P–PMe_3_]^2+^, and (ii) suggests that dispersion plays a role in the stabilization of [Ph_3_P–P*^t^*Bu_3_]^2+^. Running this reaction with overall three equivalents of PMe_3_ generated [Me_3_PH]^+^ and [Me_3_PCH_2_PMe_2_]^+^,^21b,21d^ hence delineating why [Me_3_P–PMe_3_]^2+^ is opposed to **1** of moderate synthetic use in the presence of bases. Eventually, salt **1** was found to cleanly generate corresponding halotriphenylphosphonium salts in the halide abstraction reactions with the ^–^BF_4_, ^–^PF_6_, ^–^SbCl_6_, and even ^–^SbF_6_ anions, which commonly serves as a benchmark for Lewis-superacidity (Scheme 4b).^35c,46^

Compound **1** also engages in formally homolytic P–P bond addition reactions (Scheme 4c). Insertion into the S–S bond was obtained in the reaction with diphenyldisulfide, thereby affording the [Ph_3_PSPh]^+^ cations. The transfer of two Ph_3_P^·+^ substituents was also observed in the reaction with unsaturated π-systems, namely, organic nitriles. Keeping **1** at room temperature over 2 days in acetonitrile led to the quantitative conversion to the addition product ^**Me**^**5**, which was isolated in crystalline form in 23% yield. According to the in situ ^31^P NMR spectroscopic analysis, this reaction involves the tautomerization of intermediate ^**Me**^**4**. Indeed, ^**Ph**^**4** was isolated upon reaction with benzonitrile. The addition reaction to nitriles highlights the group-transfer capabilities of **1**^**2+**^, as electron-deficient nitriles are difficult to activate under oxidative conditions. In fact, related nitrile addition reactions are only known for tungsten(0) complexes^47^ and triphosphiranes,^48^ yet not phosphine derivatives. DFT calculations indicate that the addition to nitriles is only formally homolytic in nature and instead proceeds stepwise via substitution of PPh_3_ (Figures S133 and S134).

Compound **1** is stable in dichloromethane at room temperature, and EPR is silent, hence indicating that the homolytic P–P bond scission is thermally challenging. However, irradiation in dichloromethane by a xenon lamp afforded the chlorotriphenylphosphonium cation, tentatively suggesting photolytic P–P bond cleavage (Scheme 4d). In *ortho*-difluorobenzene, **1** is stable even upon refluxing overnight, according to the NMR spectroscopic analysis. Again, the irradiation of the sample led to a swift reaction at room temperature, namely, the CH-phosphoranylation of the solvent under the generation of [Ph_3_PH]^+^ within 3 h. This formally electrophilic aromatic substitution is unusual, as it proceeds with an electron-deficient arene. It thus prospects synthetically useful substitution reactions with electron-rich arenes. Experimental evidence for homolytic P–P bond photolysis came from irradiation in the presence of 50 equiv of 9,10-dihydroanthracene (DHA; BDFE = 305 kJ mol^**–**1^).^49^ Quantitative conversion was obtained within less than 30 min, thereby generating [Ph_3_PH]^+^ as the major product. Strikingly, the NMR-spectroscopic analysis (Figures S95–100) revealed that anthracene formed substoichiometrically (0.2 equiv), whereas the homocoupled hydroanthracenyl dimer (0.6 equiv) formed majorly. This observation confirms hydroanthracenyl radicals as intermediates and hence a radical H-atom abstraction mechanism.

## Conclusions

We report that the oxidation of triphenylphosphine furnishes the hexaphenyl-1,2-diphosphonium dication in the form of its perfluorinated alkoxy aluminate salt **1**. This reaction involves the dimerization of the transient triphenylphosphine radical cation. Highlighting cooperativity, this dimerization boosts the redox-potential by +0.6 V and hence generates a potent oxidant with *E* = +1.44 V vs Fc/Fc^+^, which oxidizes trimethylphosphine. Quantum chemical calculations rationalize that **1**^**2+**^, valence isoelectronic with hexaphenylethane, does not form the equivalent of Gomberg’s dimer, as the phosphorus addition product is thermodynamically disfavored. Mimicking halogens, salt **1** may react by both heterolytic and homolytic pathways. Leading to reactivity akin to Frustrated Lewis Pairs (FLPs), heterolytic P–P bond cleavage dominates in the absence of light, hence rendering **1** a surrogate of the triphenylphosphorandiylium dication Ph_3_P^2+^. It serves as a precursor for the mixed diphosphonium dication [Ph_3_P–P*^t^*Bu_3_]^2+^ and as a superacidic halide- and oxygen-abstraction and/or dehydration reagent. Diphosphoranylation of the electron-deficient C≡N multiple bond occurs in the reaction with benzo- and acetonitrile. The irradiation with UV-light induces homolytic P–P bond cleavage to triphenylphosphine radical cations Ph_3_P^·+^, which engage in H-atom abstraction and arene CH phosphoranylation. In short, compound **1** complements our knowledge on the trityl radical and improves our understanding of oxidation catalysis with ubiquitous triphenylphosphine and corresponding transient triphenylphosphine radical cations. It thereby presents itself as a powerful Lewis acid, oxidant, as well as a powerful H-atom abstraction, deoxygenation, and phosphoranylation agent.

## Supporting Information Available

Synthetic procedures, spectroscopic data, computational and crystallographic details (PDF)

## Abbreviations

[Al^F^]: [Al{OC(CF_3_)_3_}_4_]
bcp: bond critical point
BDFE: bond dissociation free energy
DHA: 9,10-dihydroanthracene
DMAP: dimethylaminopyridine
DFT: density functional theory
DHA: dihydroanthracene
EDA-NOCV: energy decomposition analysis with natural orbitals for chemical valence
Fc: ferrocene
FLP: frustrated Lewis pair
IR: infrared
*o*DFB: 1,2-difluorobenzene
[phen^F^]^·+^: [perfluoro-5,10-bis(perfluorophenyl)-5,10-dihydrophenazinium]^·+^
PFB: pentafluorobenzene
NMR: nuclear magnetic resonance
QTAIM: quantum theory of atoms in molecules
sc-XRD: single-crystal X-ray diffractometry
TFB: 1,2,3,4-tetrafluorobenzene
