# Supplementary material for: Hexaphenyl-1,2-Diphosphonium Dication [Ph3P–PPh3]2+: Superacid, Superoxidant, or Super Reagent?
Source: J Am Chem Soc. 2025 Apr 24;147(18):15369–76. doi: 10.1021/jacs.5c01271 (PMC12063048; doi:10.1021/jacs.5c01271)
Supplement: Supplementary file 1 — ja5c01271_si_001.pdf [file ja5c01271_si_001.pdf]

SUPPORTING INFORMATION

Hexaphenyl-1,2-Diphosphonium Dication $[Ph_3P–PPh_3]^{2+}$: Superacid, Superoxidant, or Super Reagent?

Fabian Dankert,[a] Simon P. Muhm,[a] Chandan Nandi,[a] Sergi Danés,[b] Sneha Mullassery,[a] Petra Herbeck-Engel,[c] Bernd Morgenstern,[d] Robert Weiss,*[e] Pedro Salvador,*[b] Dominik Munz*[a]

[a] Saarland University, Coordination Chemistry, Campus C4.1, D-66123 Saarbrücken, Germany.

[b] Institut de Química Computacional i Catàlisi and Departament de Química, Universitat de Girona, 17003 Girona, Catalonia, Spain.

[c] INM Leibniz Institute for New Materials, Campus D2.2, D-66123 Saarbrücken, Germany.

[d] Saarland University, Inorganic Solid-State Chemistry, Campus C4.1, D-66123 Saarbrücken, Germany.

[e] Friedrich-Alexander-Universität (FAU) Erlangen-Nürnberg, Nikolaus-Fiebiger-Str. 10, D-91058 Erlangen, Germany.

Table of Contents

1 Experimental Details S3

2 Syntheses S6

2.1 [9,10-Dichlorooctafluorocenium][Al^F] (= [ant^{ClF}][Al^F]) S6

2.2 [Ph_3P–PPh_3][Al^F]$_2$ (**1**) S8

2.3 Reaction of **1** with DMAP: [Ph_3P(DMAP)][Al^F]$_2$ S18

2.4 Reaction of **1** with P^tBu_3: [Ph_3P–P^tBu_3]$^{2+}$ S23

2.5 Reaction of **1** with PMe_3: [$Me_2PCH_2PMe_3$][Al^F], [Me_3PH][Al^F] and PPh_3 ..... S25

2.6 Reaction of **1** with [PNP][OTf]: [Ph_3P−O−PPh_3][Al^F]$_2$, {(Ph_3P)$_2$O}(Tf)$_2$, and [PNP][Al^F] S30

2.7 Reaction of **1** with TolCOOH S33

2.8 Reaction of **1** with $OPEt_3$: $OPPh_3$, PPh_3 and [Et_3P−O−PEt_3][Al^F]$_2$ S37

2.9 Absence of a Reaction of **1** with *p*-Fluorobenzonitrile S41

2.10 Reaction of **1** with $SbCl_6^-$ (**3**) S43

2.11 Reaction of **1** with [Na(222crypt)][BF_4]: [$FPPh_3$][Al^F] S46

2.12 Reaction of **1** with [Na(222crypt)][PF_6]: [$FPPh_3$][Al^F] and F_5PPPh_3 ..... S49

2.13 Reaction of **1** with [PNP][SbF_6]: [$FPPh_3$][Al^F] and Ph_3PSbF_5 ..... S52

2.14 Reaction of **1** with PhSSPh: [Ph_3PSPh][Al^F] S56

2.15 Reaction of **1** with 1 eq. Water: [Ph_3PH][Al^F] and [Ph_3P−O−PPh_3][Al^F]$_2$ S60

2.16 Reaction of **1** with Benzonitrile (^{Ph}4) S63

2.17 Reaction of **1** with Acetonitrile (^{Me}5) S69

3 Irradiation, EPR and Heating of **1** S76

3.1 EPR analysis of **1** S76

3.2 Irradiation and Heating of **1** in Dichloromethane S77

3.3 Irradiation of **1** in the Presence of DHA S80

3.4 Irradiation of **1** in the Absence of DHA S85

3.5 Heating **1** in the Presence of DHA S90

4 Electrochemical Studies S92

5 X-ray structure elucidation and refinement S94

6 Computational Details S102

8 References S118

1 Experimental Details

General Information. If not stated otherwise, all manipulations were carried out under oxygen- and moisture-free conditions under an inert atmosphere of dinitrogen using standard Schlenk techniques or an UNILab pro MBraun glovebox. 1,2-difluorobenzene (1,2-DFB, *o*DFB), 1,2,3,4-tetrafluorobenzene (TFB) and pentafluorobenzene (PFB) were refluxed over CaH_2 for at least 2 d, distilled, and stored over activated 3 Å molecular sieves. All other solvents were collected from the solvent-purification system (SPS), degassed by freeze-pump-thaw, and stored over 3 Å molecular sieves. Molecular sieves were washed with copious amounts of distilled water in an Erlenmeyer flask (3×) in order to remove traces of chloride, and activated by heating to 250 °C under high vacuum for at least 5 days. Benzene, pentane and hexane were stored over a mirror of potassium. Reactants were either obtained from common commercial suppliers, or synthesized according to the literature in case of ([K][BAr^{F}_{20}],[1] [$ClPPh_3$][OTf][2] and [$phen^F$][Al^F][3]. The [$n$$Bu_4$][$BAr^{F}_{20}$] salt was prepared by stirring [K][BAr^{F}_{20}] with a stoichiometric amount [$n$$Bu_4$][Cl] overnight in dry dichloromethane. The salts [PNP][OTf] and [PNP][SbF_6] were synthesized using a modified literature reported method[4] by stirring [PNP][Cl] with a stoichiometric amount of [Ag][OTf] and [K][SbF_6], respectively, overnight in dry dichloromethane. All glassware was pre-dried in an oven at 140 °C overnight and flame-dried directly prior to usage. C_6D_6 was obtained dry and packaged under argon and was stored over a mirror of potassium, CD_2Cl_2 (deuteration degree: 99.5%) was degassed by freeze-pump-thaw and dried as well as stored over 3 Å sieves. $CDCl_3$ (deuteration degree: 99.8%) was used as received. NMR samples were prepared inside the glovebox in NMR tubes equipped with gas-tight *J. Young* valves.

Melting points were determined using an Electrothermal IA9200 Programmable Digital Melting Point Apparatus

High resolution mass spectrometry (HRMS) HRMS-APPI spectra were recorded on a Quadrupole Linear Ion Trap (QqLIT), AB Sciex API 5500 QTRAP. Electrospray-ionization MS (ESI-MS) measurements were performed on a UHR-TOF Bruker Daltonik (Bremen, Germany) maXis plus, an ESI-quadrupole time-of-flight (qToF) mass spectrometer capable of a resolution of at least 60.000 FWHM.

FTIR spectra for solid samples were recorded using a Bruker Vertex 70 spectrometer (Bruker Optics, Ettlingen, Germany), from 4500 to 400 cm^{-1} on attenuated total re-

flectance (ATR) mode. IR spectra were obtained as an average of 16 scans with a resolution of 2 cm^{-1}. Relative intensities are reported according to the abbreviations: weak (=w), medium (=m), strong (=s).

Raman spectra were recorded on a Horiba Raman HR Evolution Confocal Raman Microscope equipped with a Laser λ^{exc} = 633 nm and a 100er LWD objective. The laser-power input was varied between 25% and 100%. The measurement times were varied between 10 and 60 s. 4 mg of **1** were filled in a Büchi quartz capillary and the solid was compressed with a blunt needle. The capillary was then sealed with Critoseal® wax. The measurement was conducted by positioning the sample into the focus of the microscope. The NMR spectroscopic analysis after the Raman experiment confirmed the absence of hydrolysis/decomposition during the Raman experiment.

EPR experiments were conducted at room temperature with a continuous wave (CW) Bruker Magnettech MiniScope MS-5000 EPR spectrometer at a microwave frequency of 100 kHz, with a magnetic field modulation of 0.2 mT and a microwave power of 10 mW (10 dB).

UV-Vis electronic absorption spectra were recorded on Shimadzu UV-1900i and Shimadzu UV-2600 UV-Vis spectrophotometers with a path length of 1 cm.

NMR spectra spectra were recorded either on a Bruker Biospin Avance II+ 400 MHz WB or a Bruker Avance III HD 300 MHz spectrometer at a probe temperature of 298 K if not otherwise indicated. The chemical shifts δ are calculated in ppm; the solvent residual signals of incomplete deuterated solvent molecules were used as internal reference for the ^{1}H NMR spectra, and the respective carbon solvent signals for the ^{13}C NMR spectra. NMR spectra recorded in *o*DFB were locked using a flame-sealed capillary filled with perdeuterated DMSO or perdeuterated toluene; where applicable, $OPMe_3$ or $OPEt_3$ was added to the capillary as reference for quantitative $^{31}P\{^{1}H\}$ NMR experiments. $^{31}P\{^{1}H\}$ spectra of the crude reaction mixtures were recorded with a relaxation time D1= 30 s to allow for quantification. Test runs with various samples revealed convergence of the integrals around 25 s. NMR multiplicities are abbreviated as follows: s = singlet, d = doublet, t = triplet, q = quartet, quint = quintet, hept = heptet, m = multiplet, br = broad signal, ps = pseudo (weak *J*-couplings that are not resolved in the spectra). Coupling constants *J* are given in Hz.

Cyclovoltammetry measurements were recorded with an $EmStat^{3+}$ Blue potentiostat (PalmSens) in a nitrogen filled glovebox, a glassy carbon working electrode, platinum counter electrode, a non-aqueous Ag/Ag^{+} pseudo reference electrode, as well as $[nBu_4][Al^F]$ (0.1 M) as supporting electrolyte. After the initial measurements, ferrocene was added as internal standard, and the voltammograms were corrected by positioning the redox potential of ferrocene to 0 V. Potentials may be converted to the Standard

Hydrogen Electrode (SHE) by positioning the Fc/Fc^+ redox couple to +0.7 V ($E[Fc/Fc^+]$ = +0.46 V *vs.* SCE in CH_2Cl_2[5]; E[SCE] = +0.24 *vs.* SHE[6]). The data were processed with the PSTrace software package (PalmSens). The $[NBu_4][Al^F]$ was synthesized from $[NBu_4]$Br and $LiAl^F$ according to a literature procedure.[7]

CHNS Elemental analyses were carried out with an Elementar vario Micro Cube instrument.

Differential Scanning Calorimetry (DSC) was performed with a DSC 204 F1 Phoenix calorimeter (NETZSCH-Gerätebau GmbH, Selb, Germany) using aluminum crucibles with pierced lids under nitrogen (100 mL/min) applying a heating rate of 10 K/min and a cooling rate of 15 K/min in the temperature range between 0 and 400 °C.

2 Syntheses

2.1 [9,10-Dichlorooctafluorocenium][Al^F] (= [ant^{ClF}][Al^F])

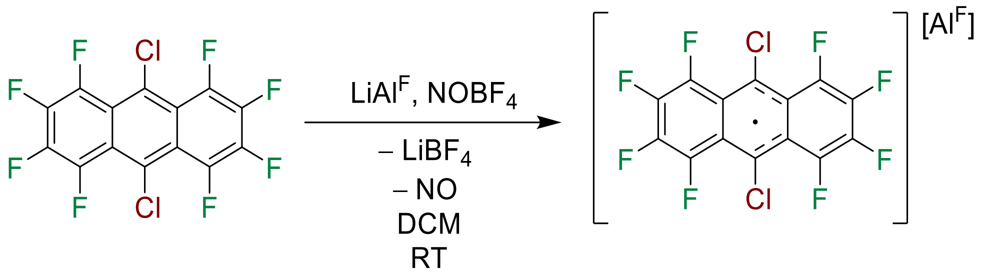

The synthesis presented herein is an alternative route to [ant^{ClF}][WCA], as reported by by KROSSING and co-workers for [WCA] = [$F(Al(OR^F)_3)_2$]$^-$.[8] Inside the glovebox, two vials are charged with 0.325 g of 9,10-dichlorooctafluoroanthracene (=ant^{ClF}) (1.0 eq, 0.831 mmol), 0.804 g $LiAl^F$ (Al^F = [$Al\{(OC(CF_3)_3\}_4$]$^-$; 1.0 eq, 0.831 mmol) and 0.097 g $NOBF_4$ (1.0 eq, 0.831 mmol). The yellow suspension is stirred overnight to obtain a dark green solution. $LiBF_4$ precipitates as a fine-white powder. Then, the deep green suspensions are filtered *via* an oven dried fine-filter frit and the residue is extracted with another 20 mL of DCM. The filtrate is separated into four different vials and the solvent of every batch is then removed under reduced pressure. Every batch is washed with 20 mL of *n*-pentane using a sonicator. After decanting the *n*-pentane inside the glovebox as well as drying the green residue *in-vacuo*, [ant^{ClF}][Al^F] is obtained as a microcrystalline dark-green powder in 58% yield (0.650 g, 0.479 mmol).

^{19}F NMR (CD_2Cl_2, 282.4 MHz): δ = –75.0 (s, –C*F*$_3$) ppm.

^{27}Al NMR (CD_2Cl_2, 78.2 MHz): δ = 34.8 (s, *Al*$\{OC(CF_3)_3\}_4$) ppm.

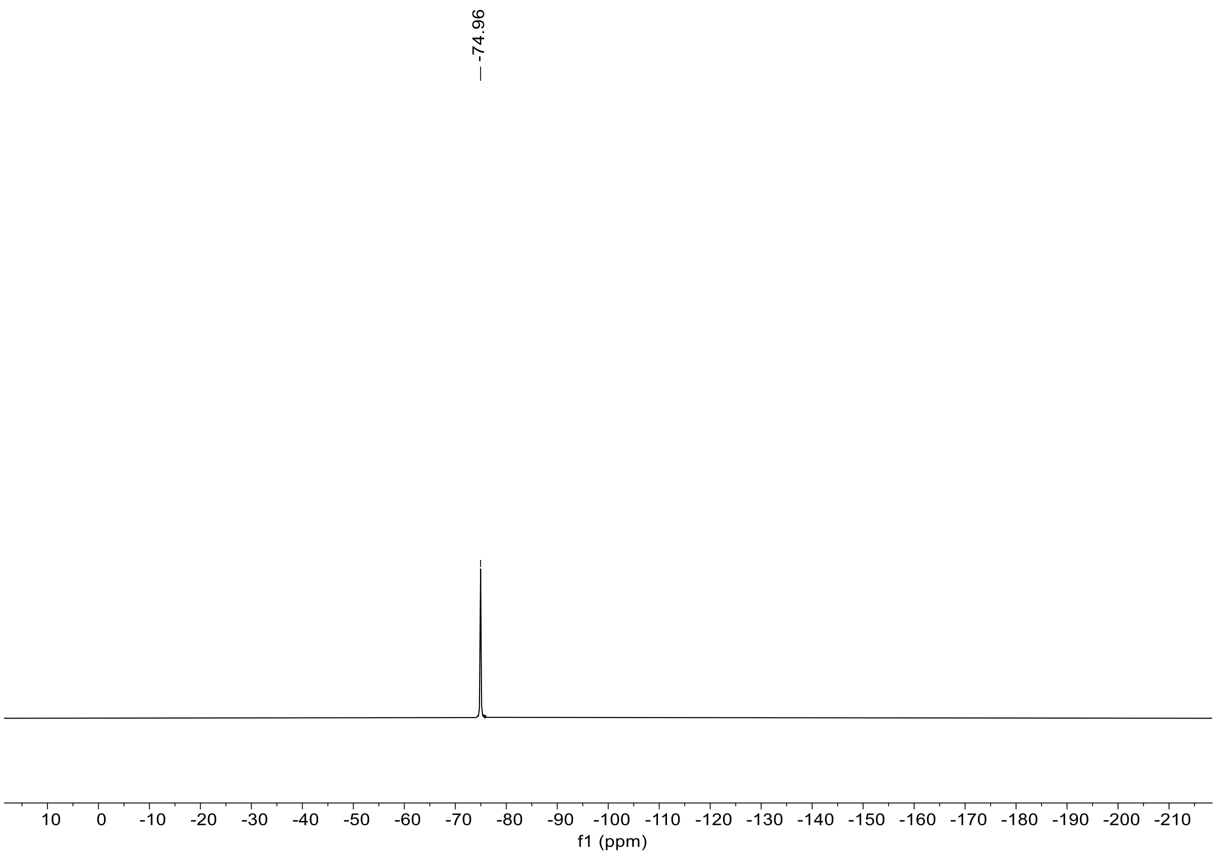

Figure S1. ^{19}F NMR spectrum of the anion in $[ant^{ClF}][Al^F]$ (CD_2Cl_2, 282.4 MHz).

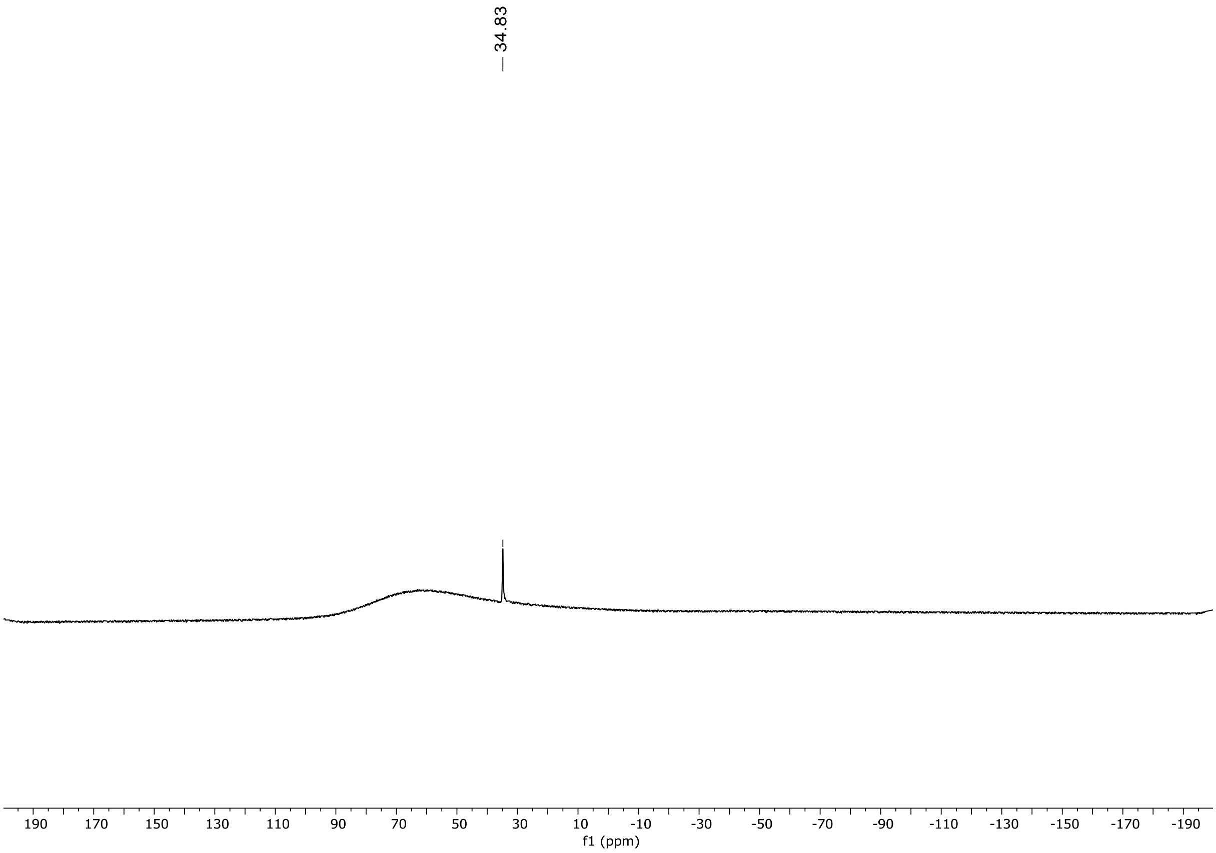

Figure S2. ^{27}Al NMR spectrum of the anion in $[ant^{ClF}][Al^F]$ (CD_2Cl_2, 78.2 MHz).

2.2 [Ph_3P–PPh_3][Al^F]$_2$ (1)

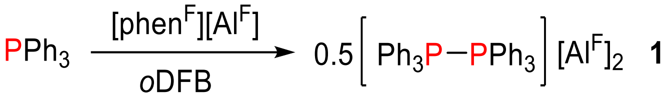

In a flame dried glass vial, [phen^F][Al^F] (314 mg, 0.193 mmol, 1 eq.) was dissolved in 0.5 mL of *o*DFB (or TFB) and cooled to a frozen solid (−34 °C/−42 °C). PPh_3 (54 mg, 0.206 mmol, 1.07 eq.) was added and the suspension was allowed to warm up to room temperature under stirring, upon which an orange suspension formed. The crude $^{31}P\{^1H\}$ NMR spectroscopic analysis at this stage revealed the next-to-quantitative pre-cipitation of the product as well as minor CH-phosphoranylation and [Ph_3PH]$^+$ formation (Figure S3, S4). The supernatant was decanted after 0.5 h, and the residue was washed 3× with 1 mL of a 1:1 mixture of pentafluorobenzene:pentane. The colorless solid was dried *in vacuo* to afford 206 mg (87%) of the title compound **1**. Colorless crystals suitable for sc-XRD were obtained by storing a saturated solution in TFB at −35 °C for 2 hours.

Note: [ant^{ClF}][Al^F] may be also used as oxidant. In this case, the yield is a bit lower (~75%) as [PPh_3Cl]$^+$ forms as byproduct. [PPh_3Cl]$^+$ may be removed in the workup by the washing/precipitation steps.

Note: **1** converts in MeCN to **^{Me}5** over the course of 48 hours.

^{1}H NMR (*o*DFB with DMSO-d_6 capillary, 400.1 MHz): δ = 7.34 (tr, 6H, CH_{Ar}), 7.07 (m, 12H, CH_{Ar}), 6.72 (m, 12H, CH_{Ar}) ppm.

^{31}P{^{1}H} NMR (*o*DFB with DMSO-d_6 capillary,162.0 MHz,): δ = 17.6 (s, Ph_3*P*) ppm.

^{19}F NMR (*o*DFB with DMSO-d_6 capillary, 282.4 MHz): δ = −76.0 (s, –CF_3) ppm.

^{1}H NMR (CD_3CN, 400.1 MHz): δ = 8.12–8.06 (m, 6H, CH_{Ar}), 7.76–7.57 (m, 12H, CH_{Ar}), 7.29–7.22 (m, 12H, CH_{Ar}) ppm.

^{13}C{^{1}H} NMR (CD_3CN, 100.6 MHz): δ = 139.4 (s, *p*-CH$_{Ar}$), 136.9 (dd, $^3J_{CP}$ = 6.8 Hz, $^4J_{CP}$ = 2.2 Hz *m*-CH$_{Ar}$), 136.9 (tr, $^2J_{CP}$ = 7.2 Hz, $^2J_{CP}$ = 7.2 Hz, $^2J_{CP}$ = 2.8 Hz, *o*-CH$_{Ar}$), 122.2 (q, $^1J_{CF}$ = 292 Hz, –CF_3), 111.2 (dtr, $^1J_{CP}$ = 80 Hz, $^1J_{CP}$ = 44 Hz, *ipso*-CH$_{Ar}$) ppm.

^{19}F NMR (CD_3CN, 282.4 MHz): δ = –76.0 (s, –CF_3) ppm.

^{27}Al NMR (*o*DFB with DMSO-d_6 capillary, 78.2 MHz): δ = 34.4 (s, *Al*{OC(CF_3)$_3$}$_4$) ppm.

^{31}P{^{1}H} NMR (CD_3CN, 162.0 MHz): δ = 21.4 (s, Ph_3*P*$^+$) ppm.

m.p.: 260 °C (decomposition; see Figure S18 for the DSC).

Raman (λ_{exc} = 633 nm): $\tilde{\nu}$ = 1099 (s), 1084 (s), 1026 (s), 998 (s), 794 (Al^F), 742 (Al^F), 611 (m), 563 (Al^F), 517(Al^F), 450 (w), 331 (Al^F), 270 (m), 243 (m), 231 (Al^F) 191 (s) cm^{-1}.

IR: $\tilde{\nu}$ = 1583 (w), 748 (m), 690 (m), 493 (s) cm^{-1}.

UV-Vis (TFB): λ =295 nm.

CHN calc. for $C_{68}H_{30}Al_2F_{72}O_8P_2$ (found) in %: C 33.22 (33.78), H 1.23 (0.92), N 0.00 (0.00).

ESI-HRMS (in *o*DFB):
m/z calcd. for $C_{18}H_{15}P^+$: 262.0911; found 262.0911.
APPI-HRMS (in *o*DFB):
m/z calcd. for $C_{24}H_{18}F_2P^+$: 375.1114; found 375.1186.
m/z calcd. for $C_{18}H_{16}P^+$: 263.0990; found 263.1008.
m/z calcd. for $C_{18}H_{16}OP$: 279.0939; found 279.0970.

APPI-HRMS (in $CDCl_3$):
m/z calcd. for $C_{24}H_{18}F_2P^+$: 375.1114; found 375.1213.

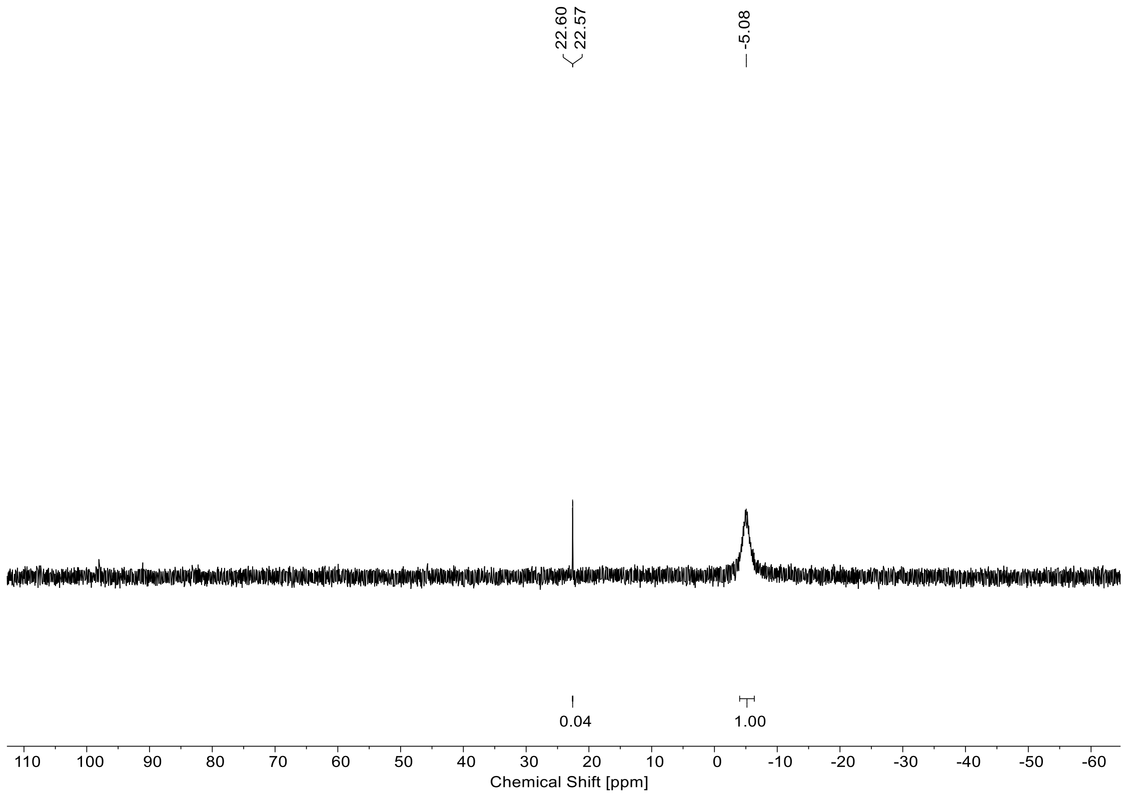

Figure S3. Crude $^{31}P\{^1H\}$ NMR spectrum of $[Ph_3P–PPh_3][Al^F]_2$ (*o*DFB with DMSO-d_6 capillary, 162.0 MHz, D1 = 30 s). The product has almost quantitatively precipitated from the solution and/or exchanges with PPh_3 and $[Ph_3PH]^+$ (δ = −5.1 ppm). One doublet ($^4J_{PF}$ = 4.9 Hz) at 22.6 ppm indicates CH phosphoranylation of *o*DFB in the *meta*-position.[9]

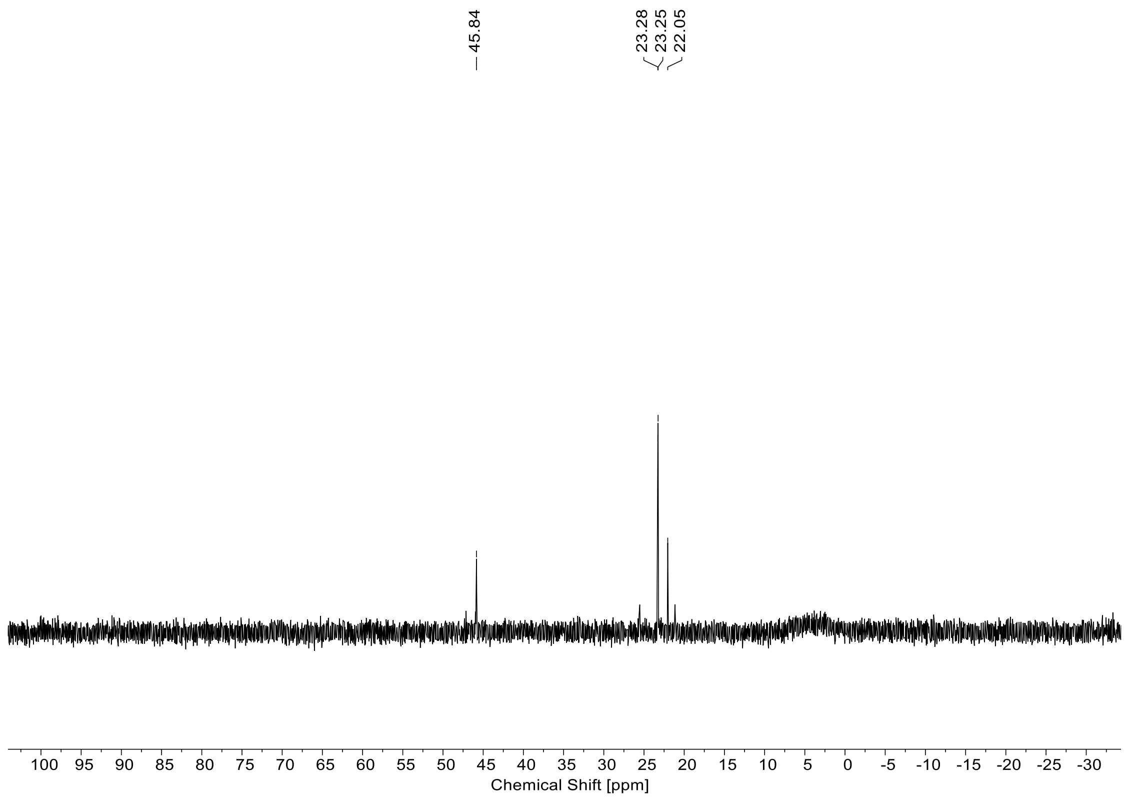

Figure S4. $^{31}P\{^{1}H\}$ NMR spectrum of the supernatant of the reaction mixture after washing with pentane ($CDCl_3$, 162.0 MHz, D1 = 30). The NMR spectrum suggests the minor formation of $[Ph_3PH]^+$ with concomitant CH-phosphoranylation of *o*DFB in the *meta*-position.[9]

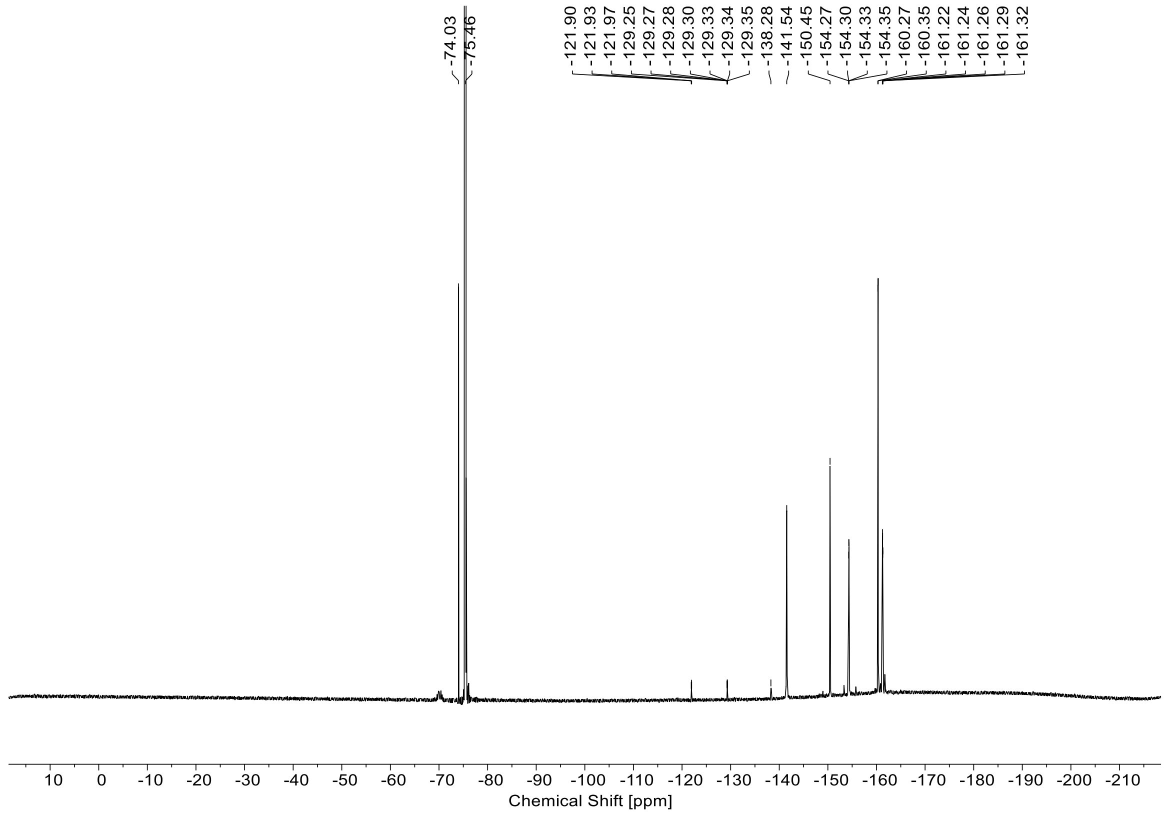

Figure S5. ^{19}F NMR spectrum of the supernatant of the reaction mixture ($CDCl_3$, 282.4 MHz). Resonances at δ = −141.54, −150.45, −154.33, −160.31 and 161.32 ppm belong to phen^F. The two multiplets at δ = −121.93 and −129.30 ppm are attributed to the CH phosphoranylation product (see section 3).

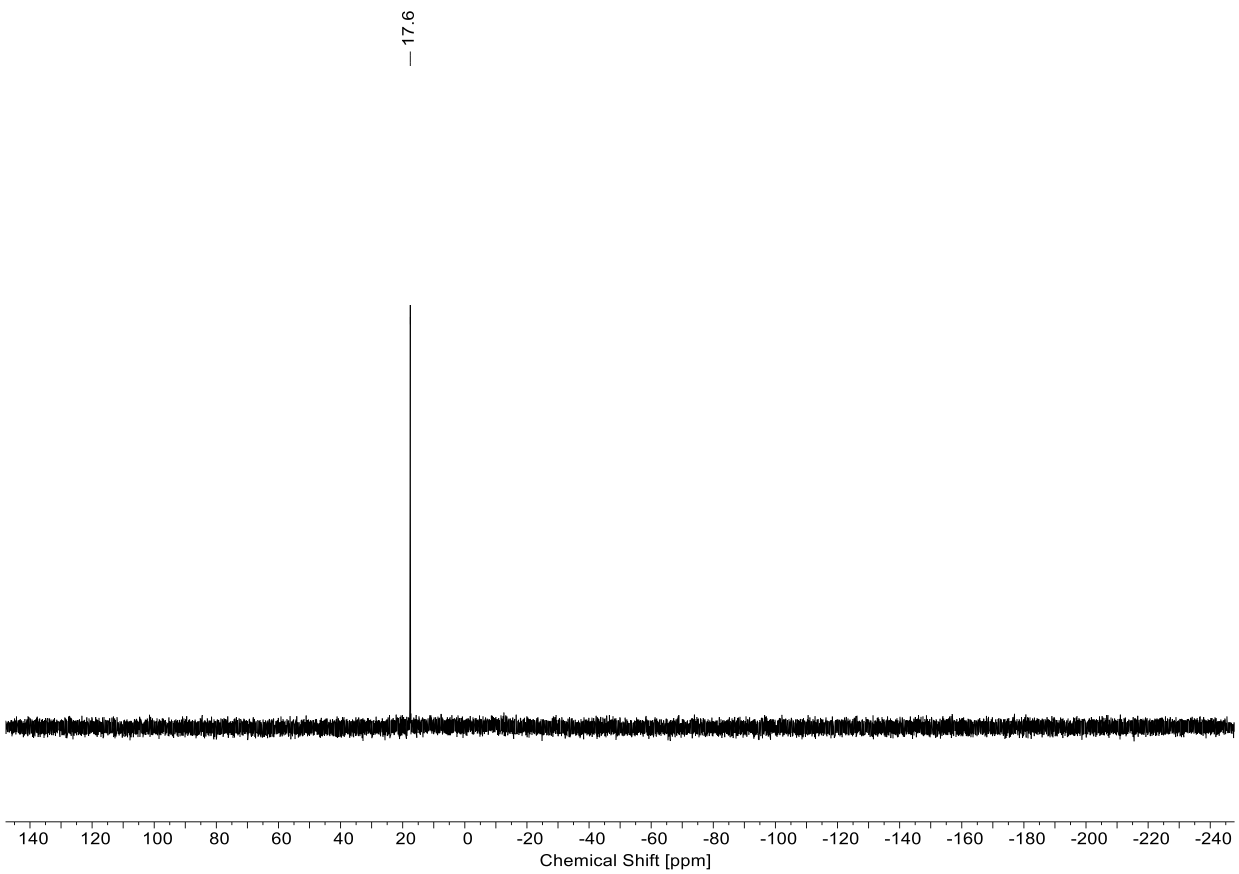

Figure S6. $^{31}P\{^1H\}$ NMR spectrum of $[Ph_3P–PPh_3][Al^F]_2$ (*o*DFB with DMSO-d_6 capillary, 162.0 MHz).

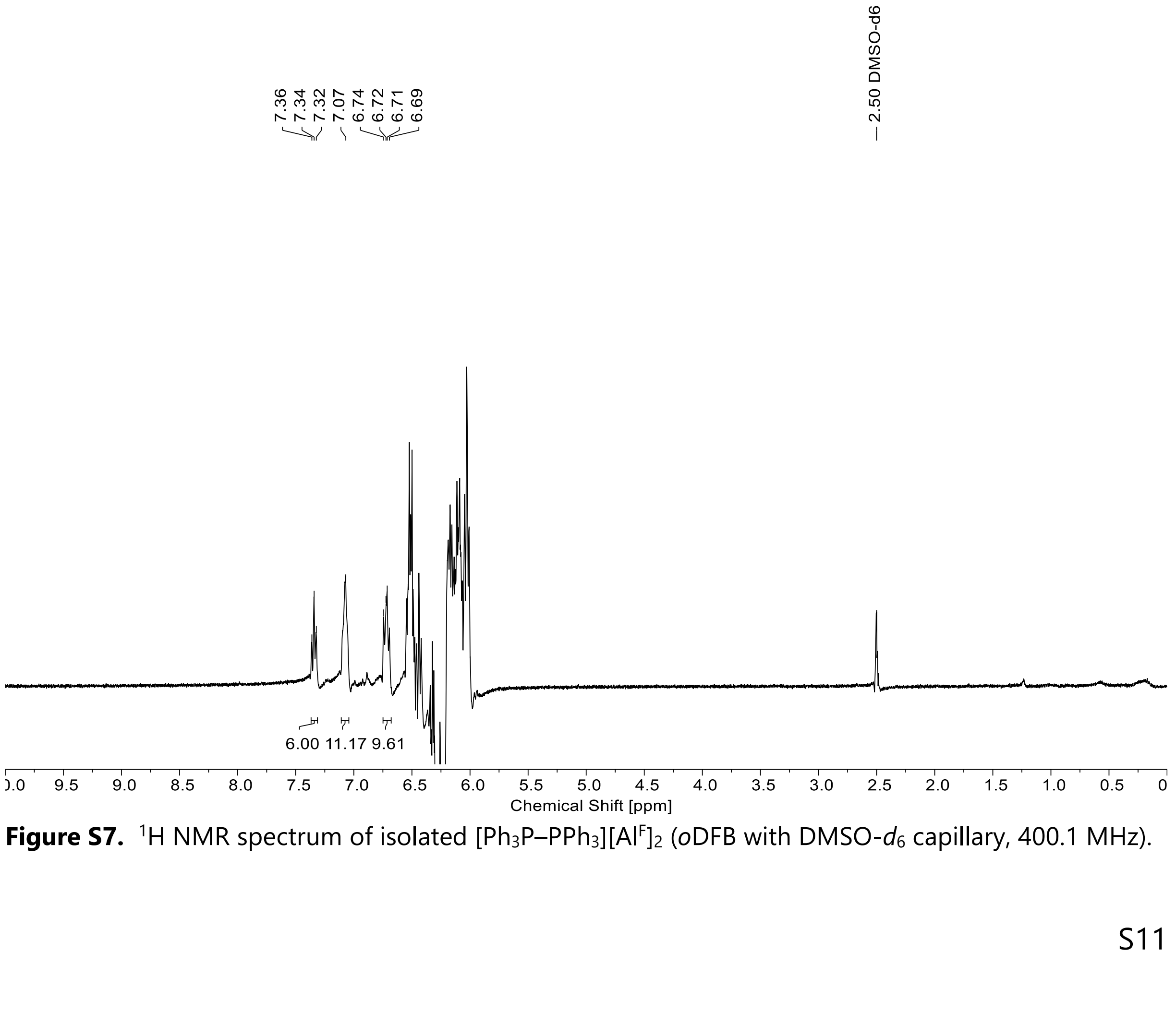

Figure S7. ^1H NMR spectrum of isolated $[Ph_3P–PPh_3][Al^F]_2$ (*o*DFB with DMSO-d_6 capillary, 400.1 MHz).

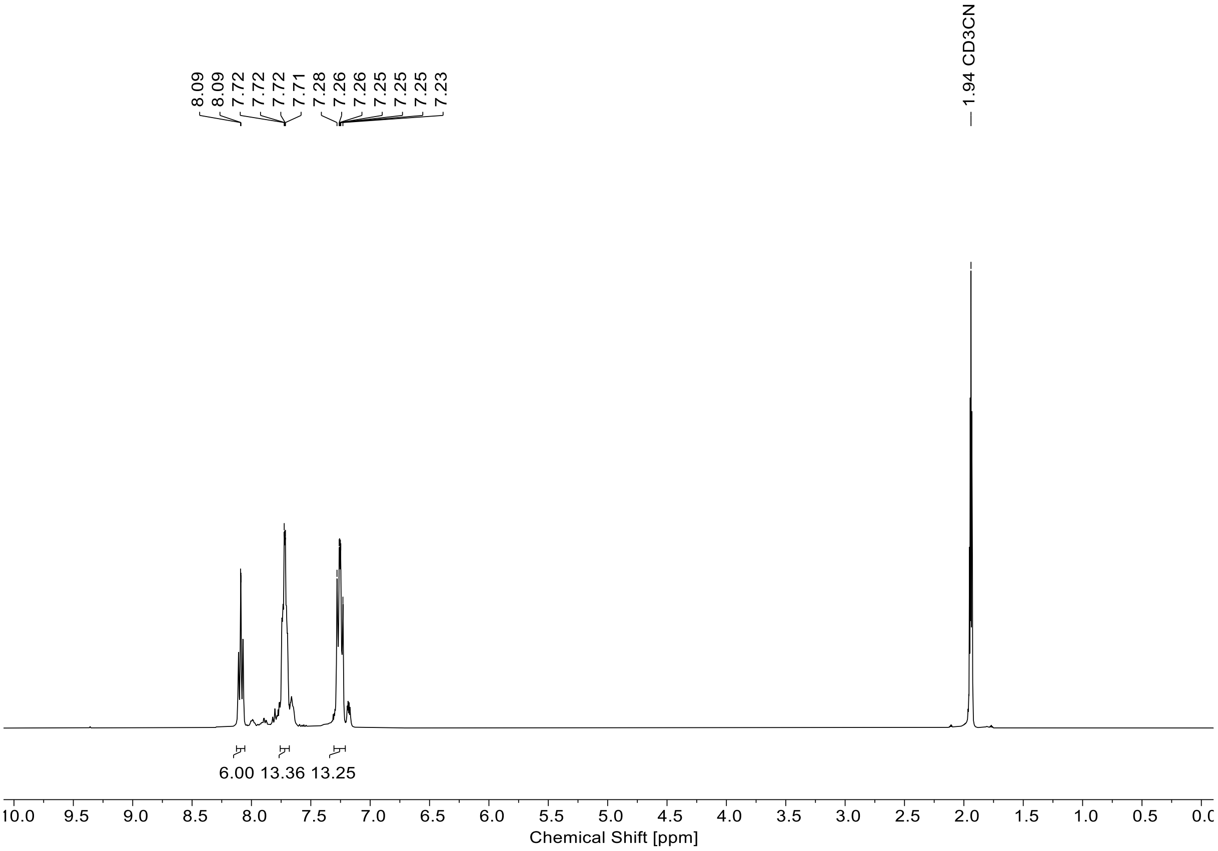

Figure S8. ^{1}H NMR spectrum of $[Ph_3P–PPh_3][Al^F]_2$ (CD_3CN, 400.1 MHz).

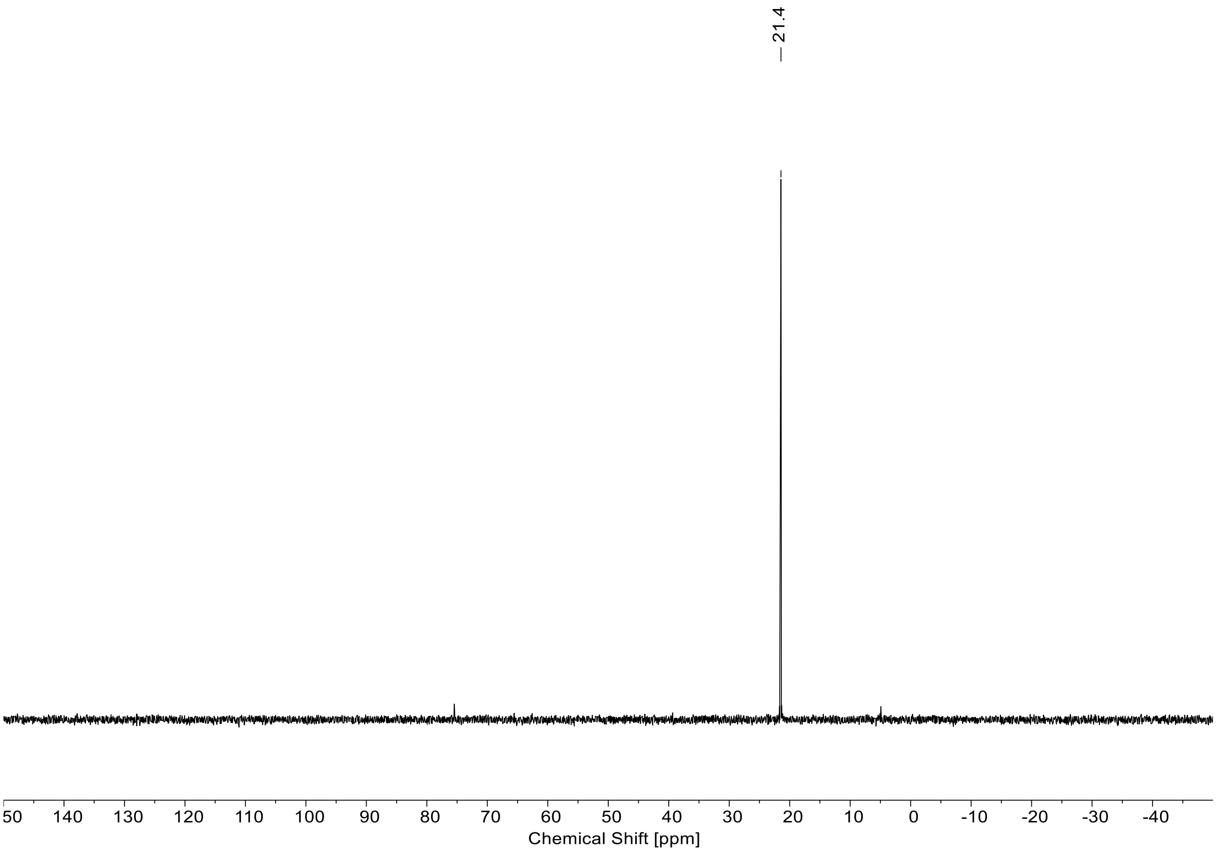

Figure S9. ^{31}P{^{1}H} NMR spectrum of $[Ph_3P–PPh_3][Al^F]_2$ (CD_3CN, 121.5 MHz).

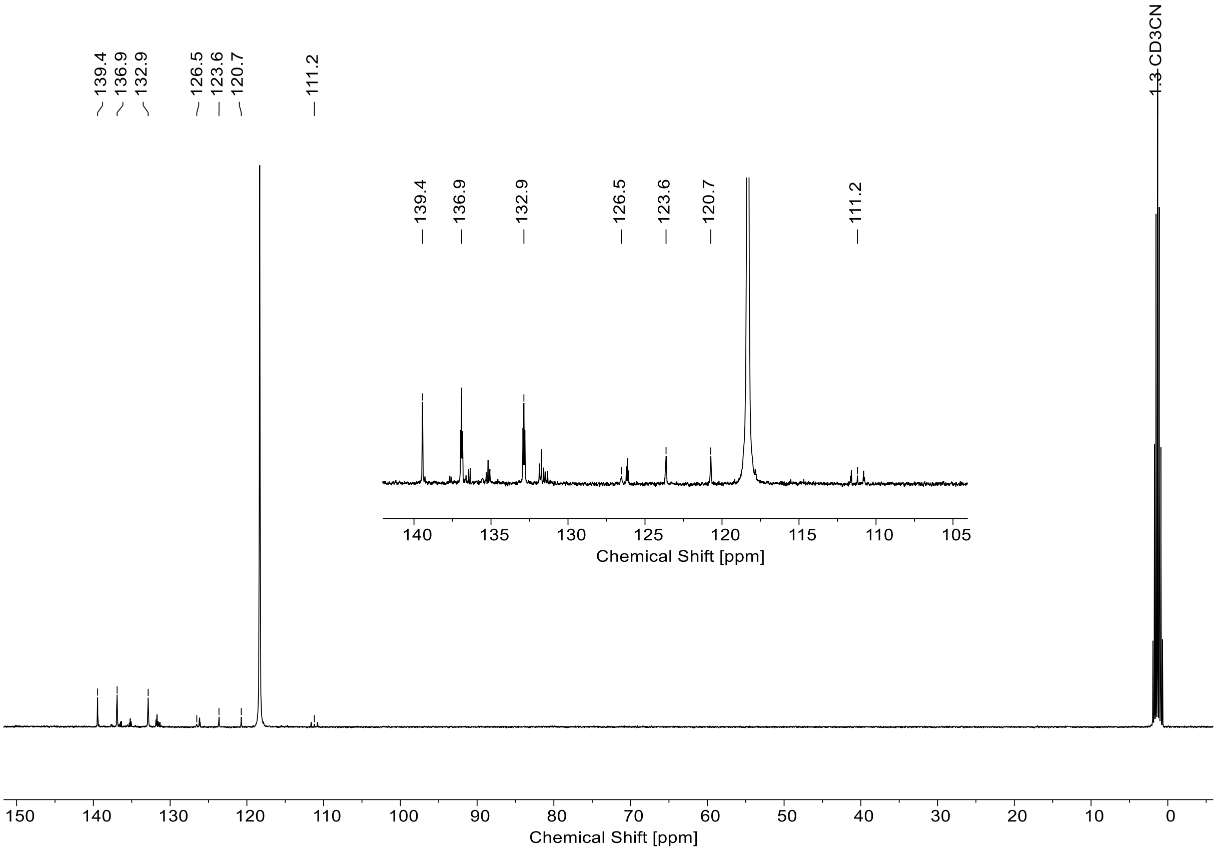

Figure S10. $^{13}C\{^{1}H\}$ NMR spectrum of $[Ph_3P–PPh_3][Al^F]_2$ (CD_3CN, 100.6 MHz).

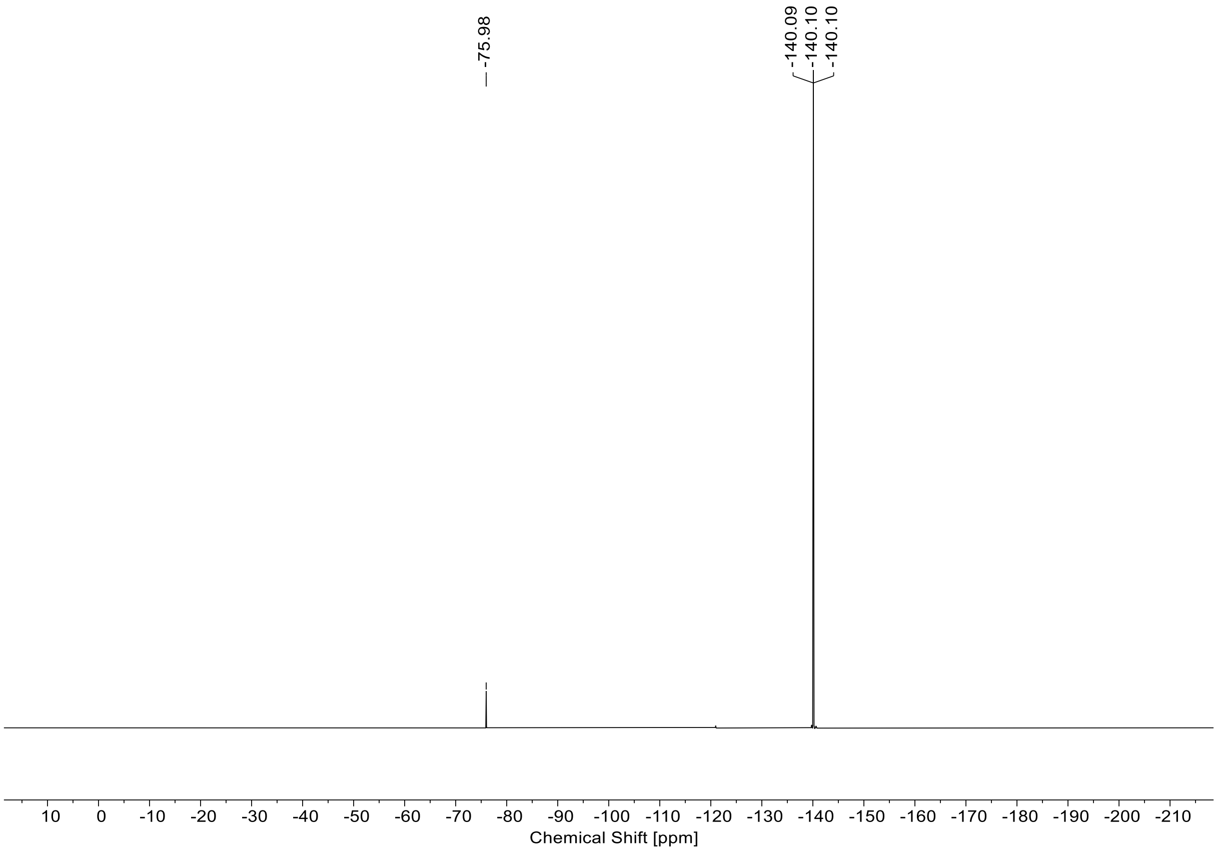

Figure S11. ^{19}F NMR spectrum of $[Ph_3P–PPh_3][Al^F]_2$ (*o*DFB with DMSO-d_6 capillary, 282.4 MHz).

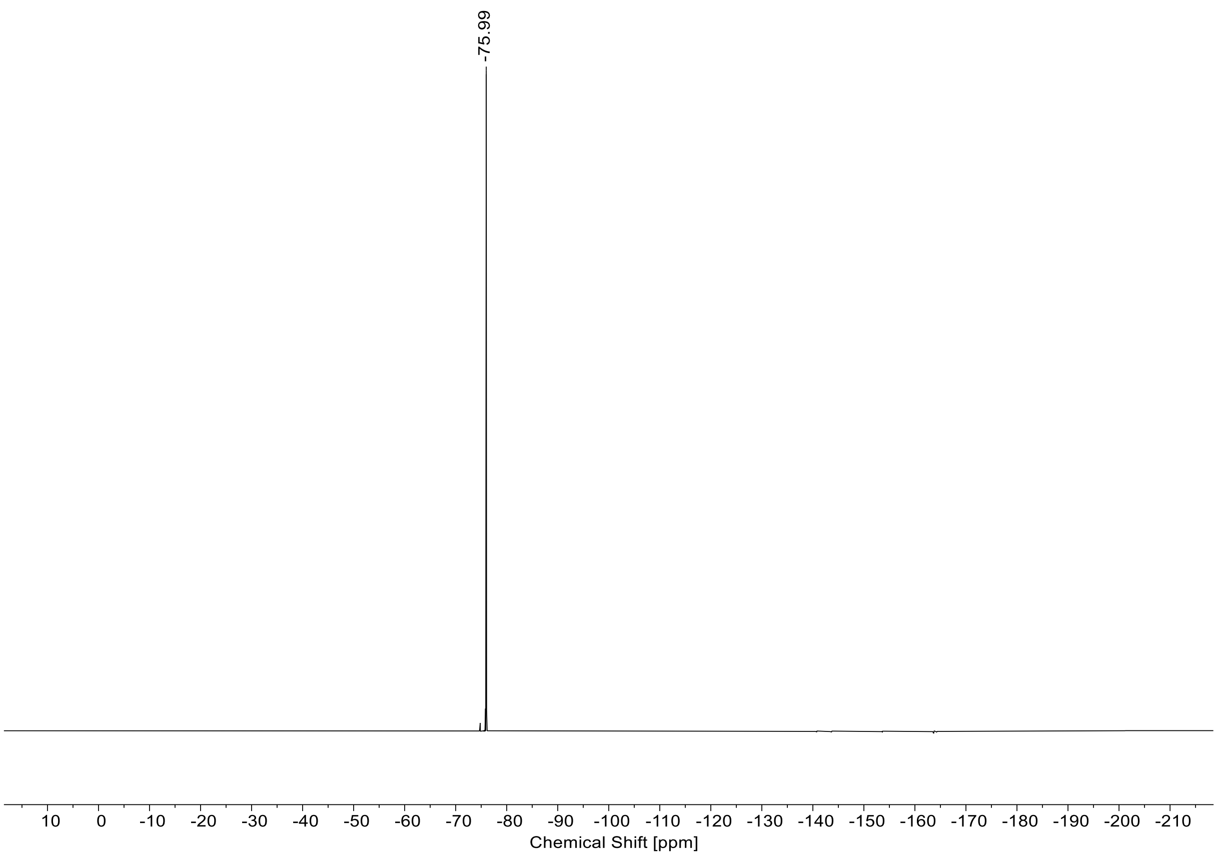

Figure S12. ^{19}F NMR spectrum of $[Ph_3P–PPh_3][Al^F]_2$ (CD_3CN, 282.4 MHz).

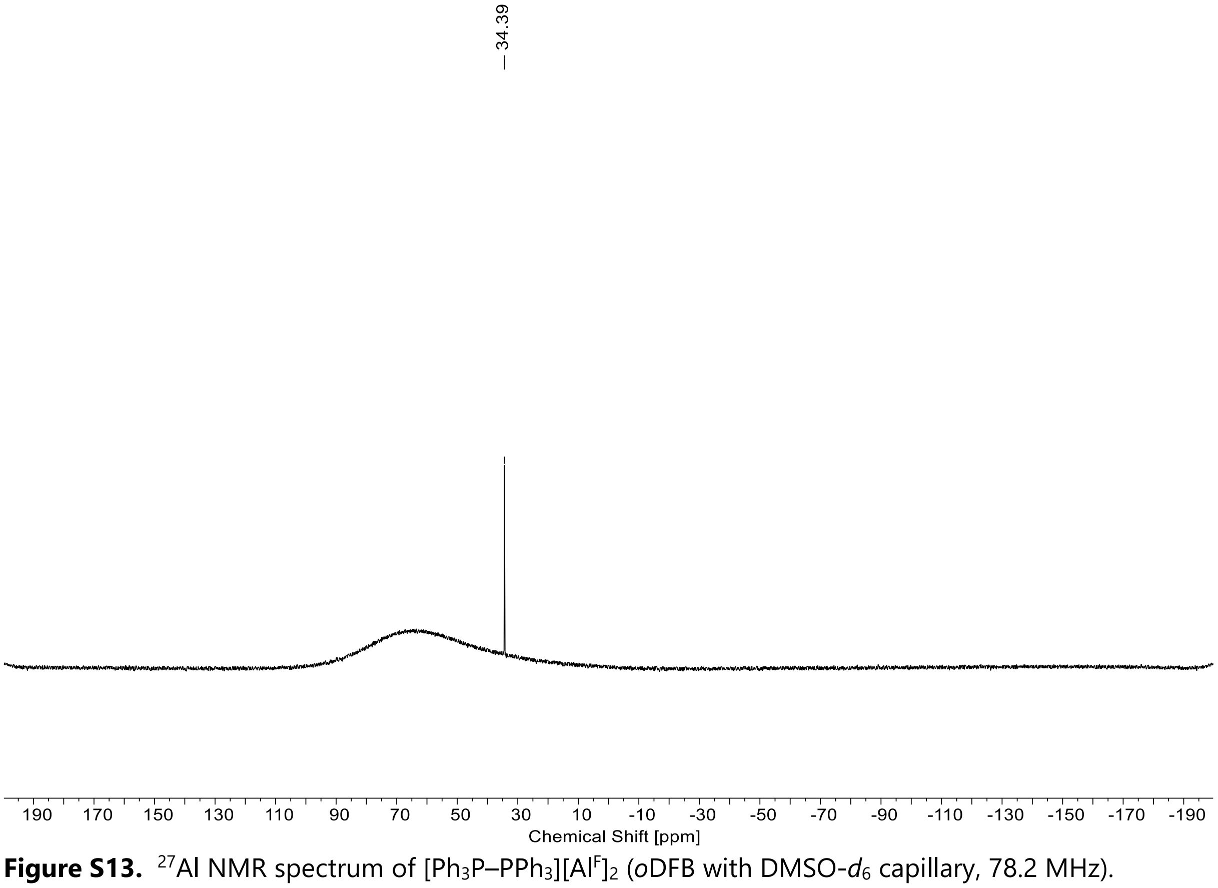

Figure S13. ^{27}Al NMR spectrum of $[Ph_3P–PPh_3][Al^F]_2$ (*o*DFB with DMSO-d_6 capillary, 78.2 MHz).

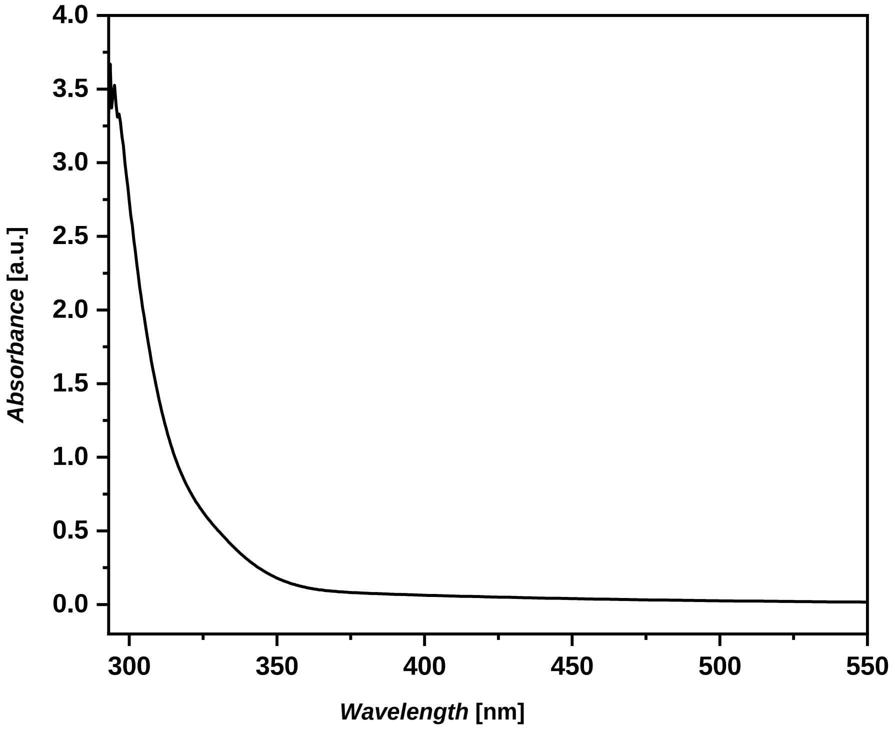

Figure S14. UV-Vis electronic absorption spectrum of $[Ph_3P–PPh_3][Al^F]_2$ in TFB.

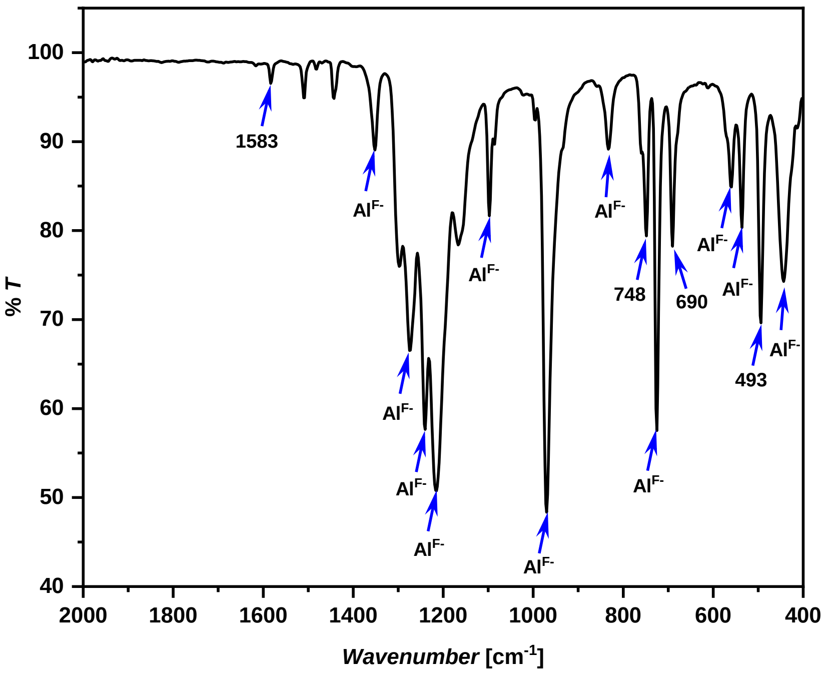

Figure S15. ATR-IR spectrum of $[Ph_3P–PPh_3][Al^F]_2$. Vibrations pertinent to the $[Al^F]$ anion were assigned based on the literature values for $[Li][Al^F]$.[7]

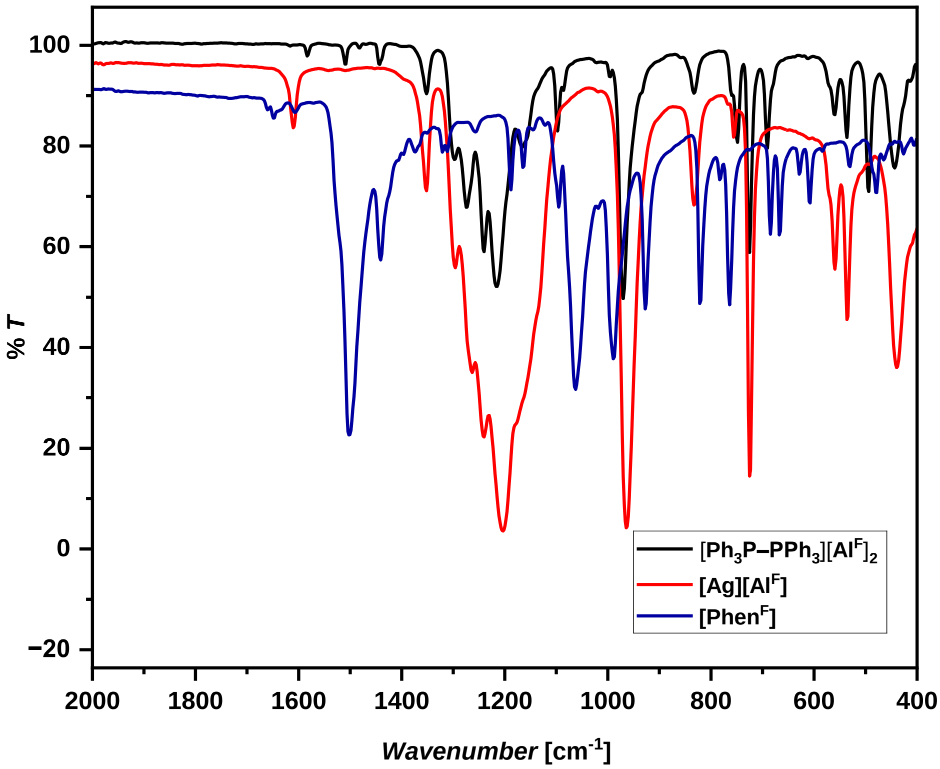

Figure S16. ATR-IR spectrum of $[Ph_3P–PPh_3][Al^F]_2$ and comparison with spectra of $phen^F$ as well as $[Ag][Al^F]$.

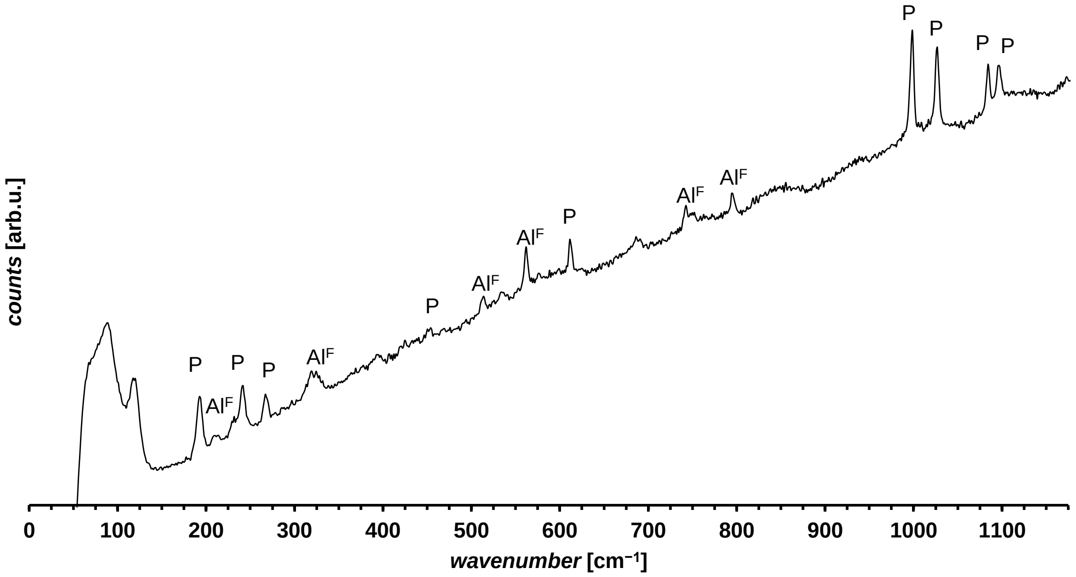

Figure S17. RAMAN spectrum of a solid sample of **1**. P designates modes attributed to the cation, $[Al^F]$ modes attributed to the $[Al^F]$ anion based on the literature values for $[Li][Al^F]$.[10]

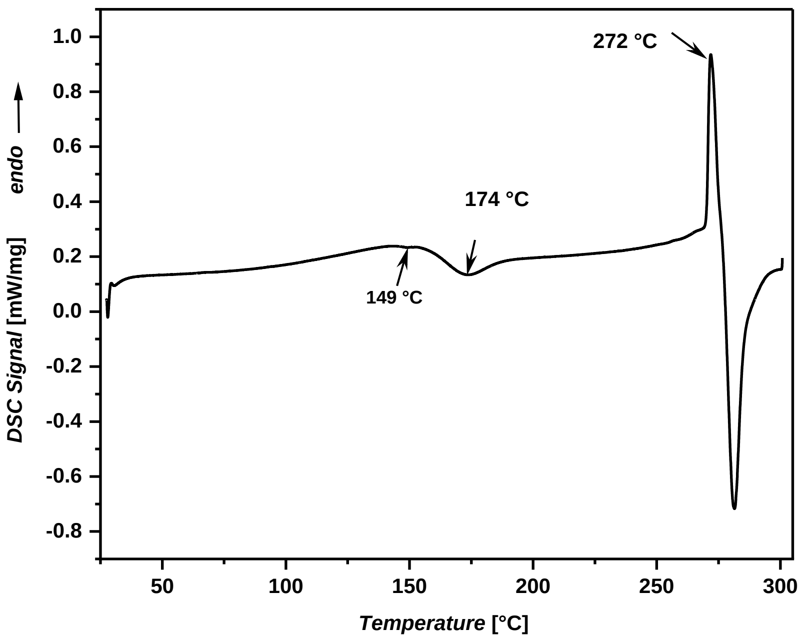

Figure S18. Differential Scanning Calorimetry (DSC) of **1**.

2.3 Reaction of 1 with DMAP: $[Ph_3P(DMAP)][Al^F]_2$

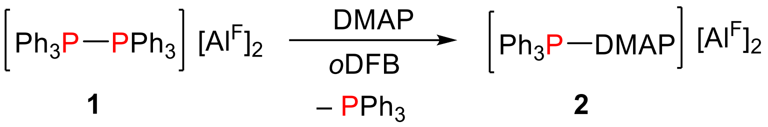

DMAP (1.5 mg, 0.012 mg, 1 eq.) was added to a suspension of 30 mg of $[Ph_3P–PPh_3][Al^F]_2$ (1.0 eq, 0.004 mmol) in 0.4 mL of *o*DFB in a *J. Young* NMR tube, upon which a clear solution formed, followed by precipitation. The ^{31}P-NMR spectroscopic analysis at this stage indicated the quantitative conversion to **2** and triphenylphosphine (Figure S19). Pentane was added to afford a colorless precipitate, which was washed with pentane (2 × 1 mL) and benzene in the cold (0.5 mol) and dried *in vacuo* to afford 25 mg of **2** (88%). For the NMR-spectroscopic analysis, $NaBArF_{20}$ was added to enhance solubility.

Single crystals suitable for X-ray diffraction analysis were obtained by vapor diffusion of pentane into a concentrated *o*DFB solution.

^{1}H NMR (CD_2Cl_2, 400.1 MHz): δ = 8.22 (tr, 3H, CH_{Ar}), 7.98–7.91 (m, 7H, $CH_{Ar,}$ and CH_{Ar} of free DMAP), 7.73–7.67 (m, 8H, CH_{Ar} of PPh_3 and CH_{Ar} of DMAP), 7.01 (d, $^3J_{HH}$ = 8.3 Hz, 2H, CH_{Ar} of DMAP), 7.01 (d, $^3J_{HH}$ = 6.9 Hz, 2H, CH_{Ar} of free DMAP), 3.46 (s, 6H, CH_3) 3.29 (s, 3H, CH_3, of free DMAP) ppm.

^{13}C{^{1}H} NMR (CD_2Cl_2, 75.5 MHz): δ = 150.0 (br s, C_{Ar} of DMAP), 146.8 (br s, CH_{Ar} of DMAP), 139.9 (d, $CH_{Ar,}$ $^4J_{CP}$ = 2.9 Hz), 135.0 (d, $CH_{Ar,}$ $^3J_{CP}$ = 12.0 Hz), 132.5 (d, $CH_{Ar,}$ $^2J_{CP}$ = 14.1 Hz), 112.0 (d, $^1J_{CP}$ = 97.7 Hz, C_{ipso} of PPh_3), 110.3 (s, CH_{Ar} of DMAP), 41.9 (s, CH_3) ppm.

^{31}P{^{1}H} NMR (CD_2Cl_2, 162.0 MHz): δ = 60.1 (s, Ph_3P) ppm.

^{31}P{^{1}H} NMR (*o*DFB with DMSO-d_6 capillary, 121.5 MHz): δ = 58.7 (s, Ph_3P) ppm.

^{19}F NMR (CD_2Cl_2 282.4 MHz): δ = −75.7 (s, –CF_3) ppm.

^{27}Al NMR (CD_2Cl_2, 78.2 MHz): δ = 34.73 (s, $Al\{OC(CF_3)_3\}_4$) ppm.

CHN calc. for $C_{57}H_{25}Al_2F_{72}N_2O_8P$ (found) in %: C 29.53 (29.22), H 1.09 (1.06), N 1.21 (1.12).

m.p.: 180 °C (decomposition).

APPI-HRMS (in *o*DFB):

m/z calcd. for $C_{18}H_{16}P^{+}$: 263.0990; found 263.0999.

m/z calcd. for $C_{18}H_{16}OP$: 279.0939; found 279.0946.

m/z calcd. for $C_7H_{11}N_2$: 123.0922; found 123.0923 (small signal).

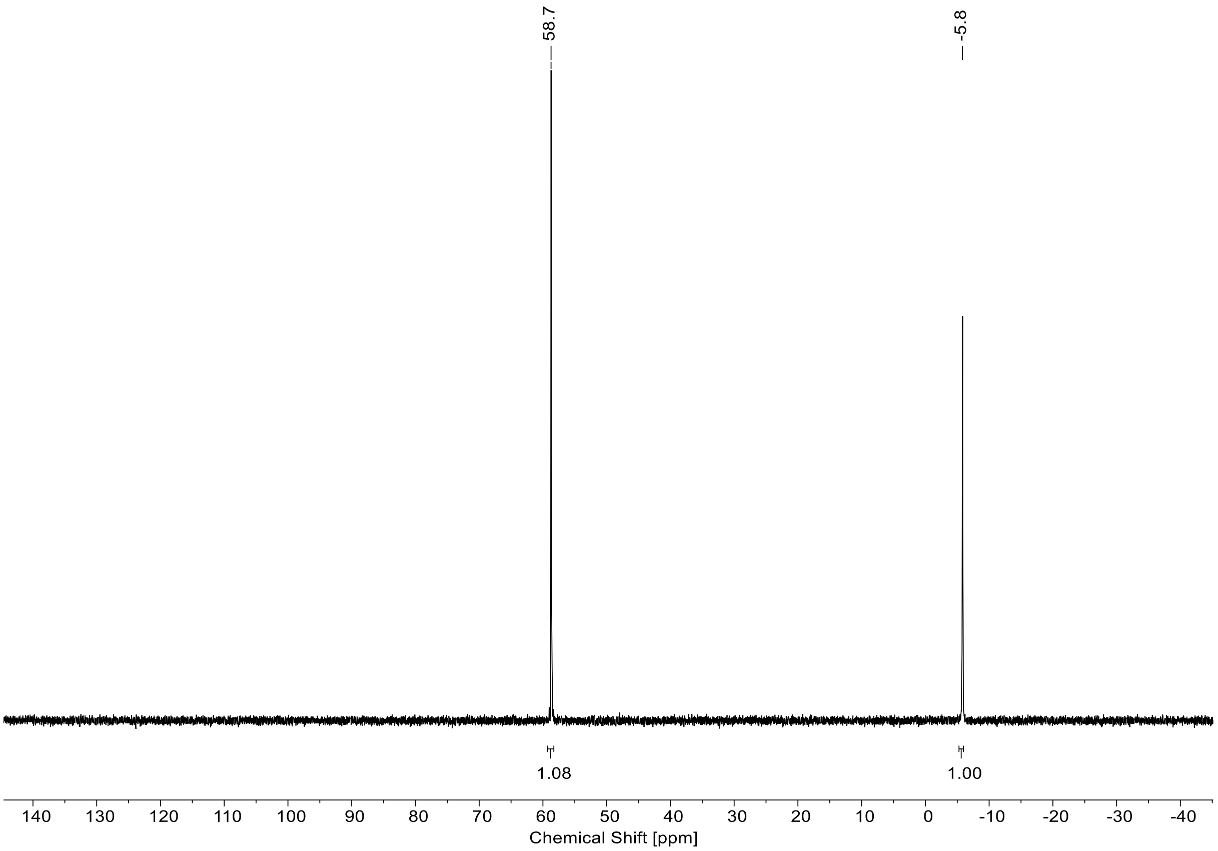

Figure S19. $^{31}P\{^1H\}$ NMR spectrum after the addition of 1 equiv. DMAP to **1** showing a 1:1 ratio of **2** and free PPh_3 (*o*DFB with DMSO-d_6 capillary, 162.0 MHz, D1 = 30 s).

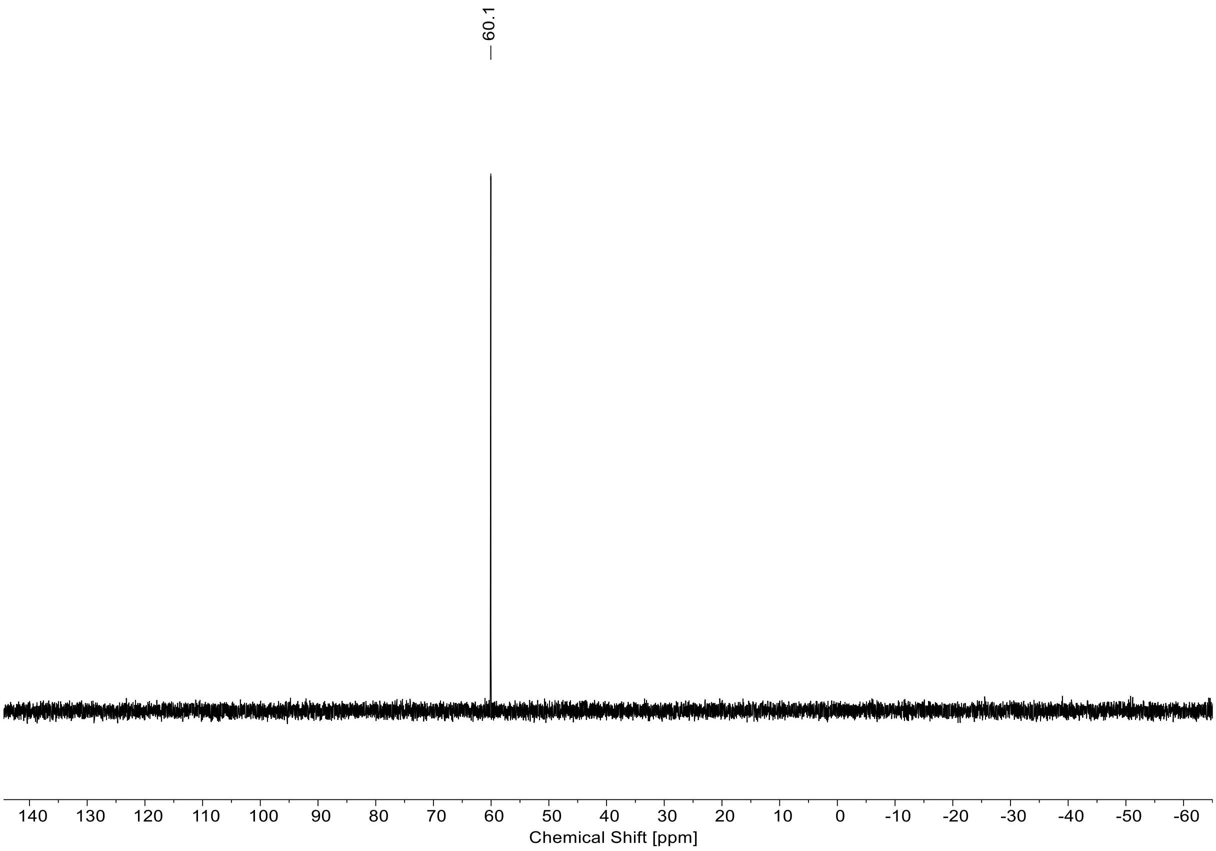

Figure S20. $^{31}P\{H\}$ NMR spectrum of **2** (CD_2Cl_2, 162.0 MHz).

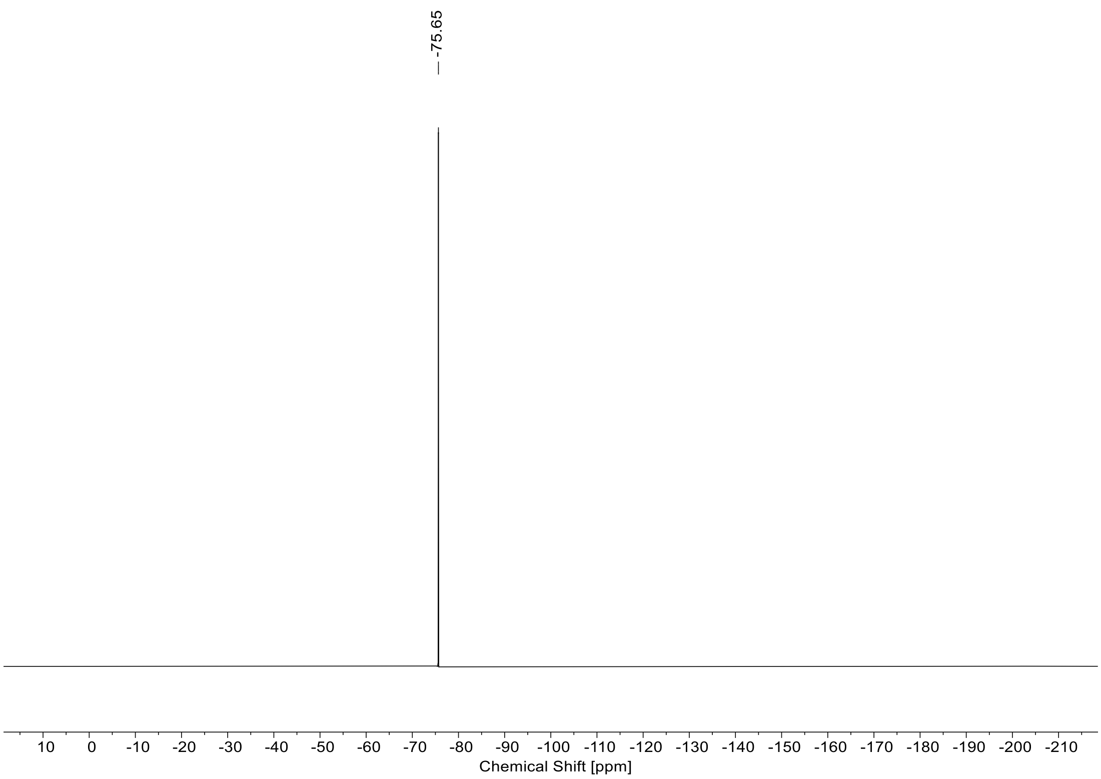

Figure S21. ^{19}F NMR spectrum of **2** (CD_2Cl_2, 282.4 MHz).

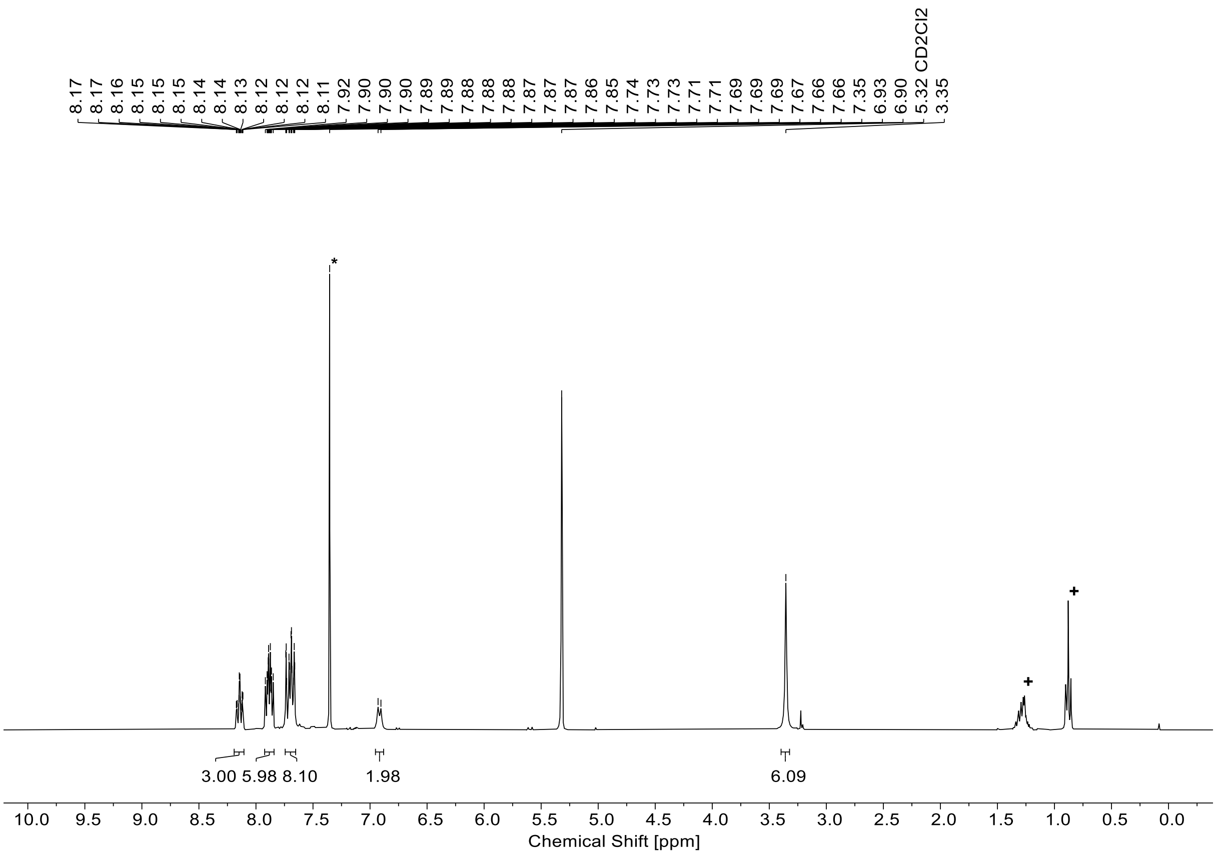

Figure S22. ^{1}H NMR spectrum of **2** (CD_2Cl_2, 400.1 MHz; + = n-pentane, * = benzene); $NaBArF_{20}$ was added to enhance solubility.

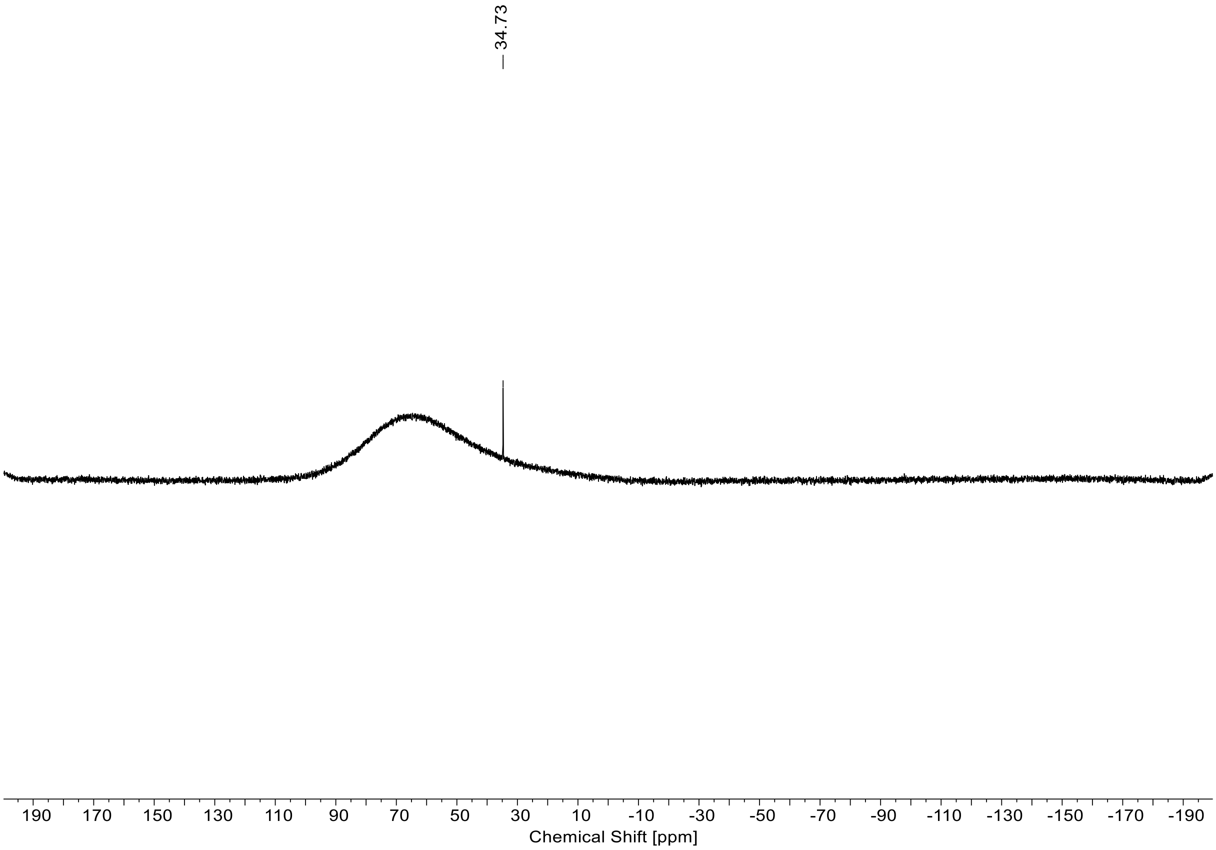

Figure S23. ^{27}Al NMR spectrum of **2** (CD_2Cl_2, 78.2 MHz).

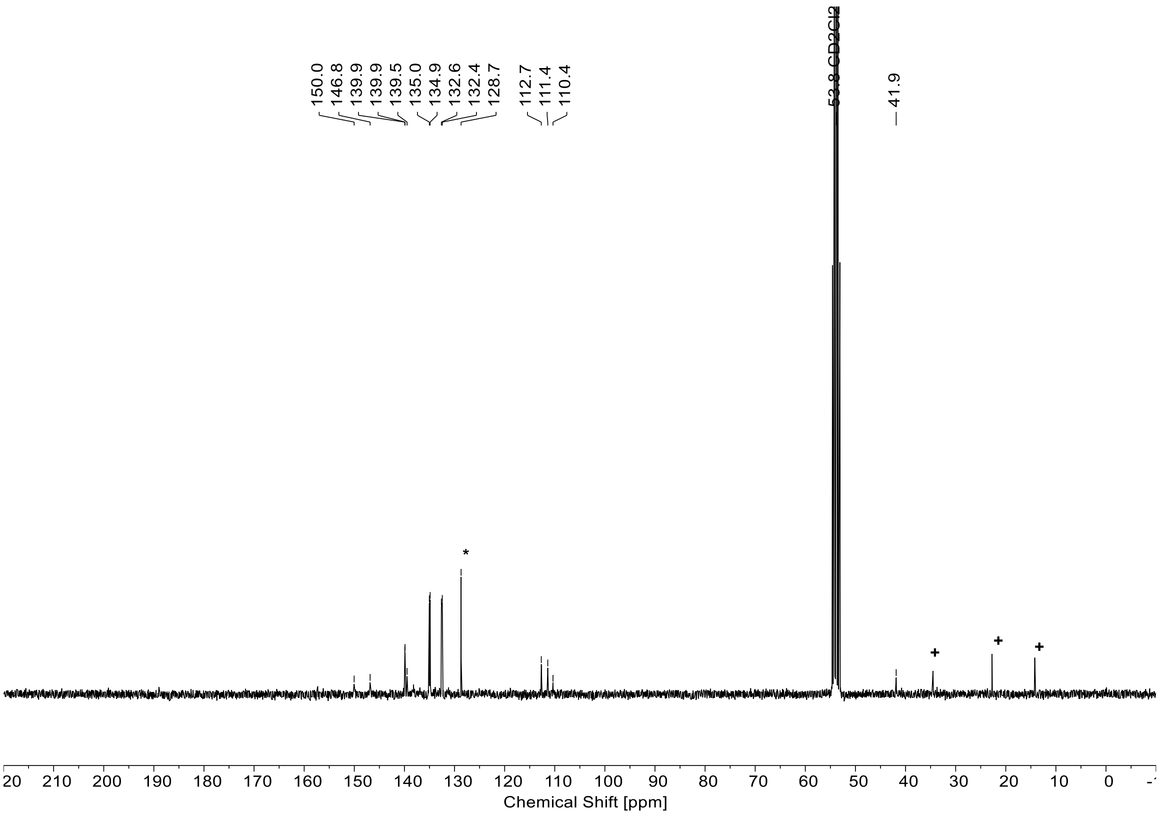

Figure S24. $^{13}C\{^1H\}$ NMR of **2** (CD_2Cl_2, 75.5 MHz + = pentane * = benzene); Na[$BArF_{20}$] was added to enhance solubility.

2.4 Reaction of 1 with P^{t}Bu$_3$: [Ph$_3$P–P^{t}Bu$_3$]$^{2+}$

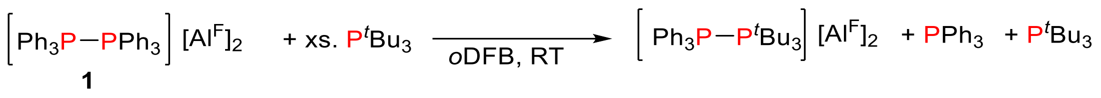

7 mg of [Ph$_3$P–PPh$_3$][Al^F]$_2$ (1.0 eq, 0.003 mmol) were suspended in 0.4 mL of *o*DFB in a *J. Young* NMR tube. 0.2 mL of a stock solution (10 mg mL^{-1} in *o*DFB) of P^{t}Bu$_3$ (~4 eq 0.012 mmol) were added. The colorless solution was analyzed by ^{1}H NMR and ^{31}P{^{1}H} NMR spectroscopies.

^{1}H NMR (*o*DFB with DMSO-*d*$_6$ capillary, 400.1 MHz): δ = 7.28–7.23 (m, 6H, C*H*$_{Ar}$), 7.08–7.04 (m, 3H, C*H*$_{Ar}$), 6.94–6.89 (m, 6H, C*H*$_{Ar}$), 4.23 (d, $^1J_{HP}$ = 432 Hz, [^{t}Bu$_3$*P*–H]$^+$), 0.83 (d, $^3J_{HP}$ = 10 Hz, C(C*H*$_3$)$_3$, [^{t}Bu$_3$*P*–H]$^+$), 0.59 (d, $^3J_{HP}$ = 10 Hz, C(C*H*$_3$)$_3$) ppm.

^{31}P{^{1}H} NMR (*o*DFB with DMSO-*d*$_6$ capillary,162.0 MHz,): δ = 84.7 (d, $^2J_{PP}$ = 49 Hz, Ph$_3$*P*–), δ = 62.5 (d, $^2J_{PP}$ = 49 Hz, –*P*^{t}Bu$_3$), δ = 60.5 (s, [^{t}Bu$_3$*P*–H]$^+$), δ = −5.9 (s, *P*Ph$_3$) ppm.

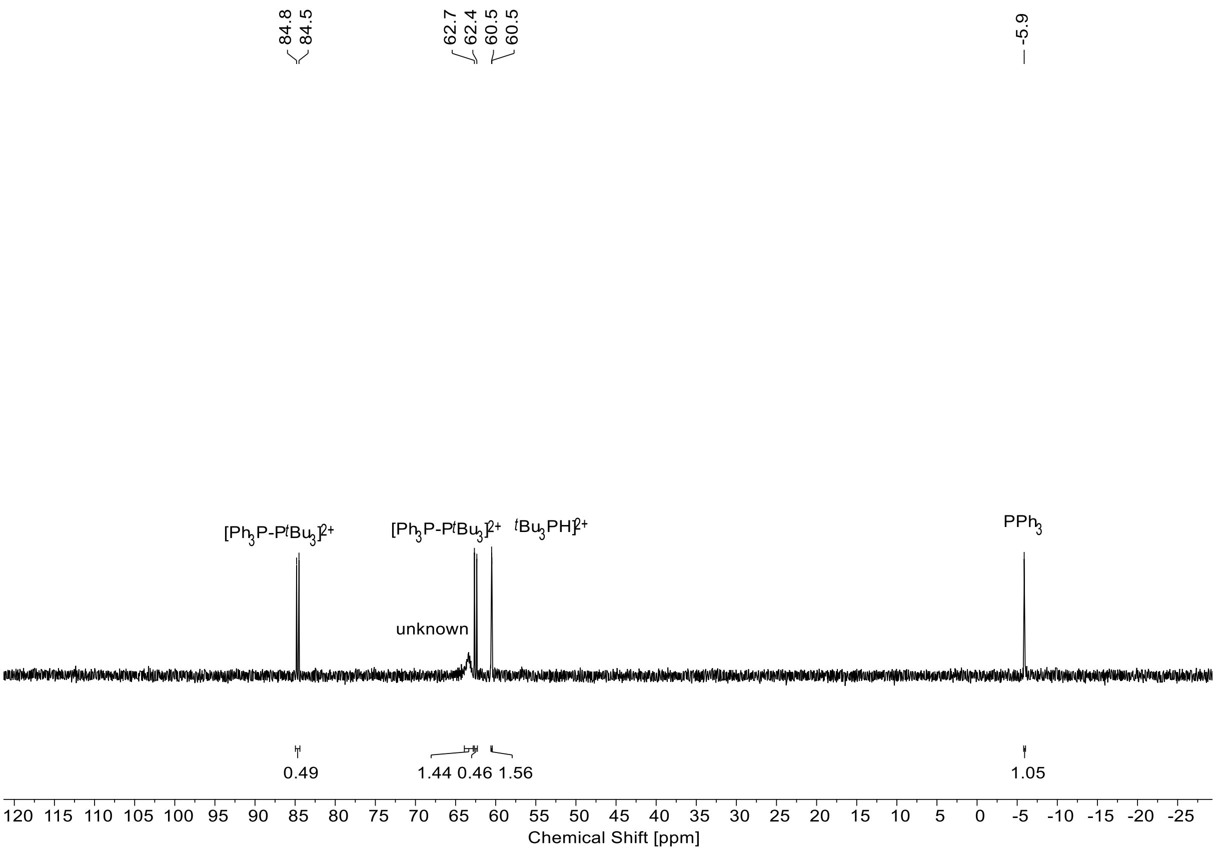

Figure S25. ^{31}P{^{1}H} NMR spectrum (*o*DFB with DMSO-*d*6 capillary, 162.0 MHz, D1 = 25 s) showing the quantitative formation of [Ph$_3$P–P^{t}Bu$_3$][Al^F]$_2$ (δ = 84.7 ppm, δ = 62.6 ppm, $^1J_{PP}$ = 49 Hz), unknown species (δ = 62.7 ppm), [^{t}Bu$_3$P–H]$^+$ (δ = 60.5 ppm), and free PPh$_3$ (δ = −5.9 ppm).

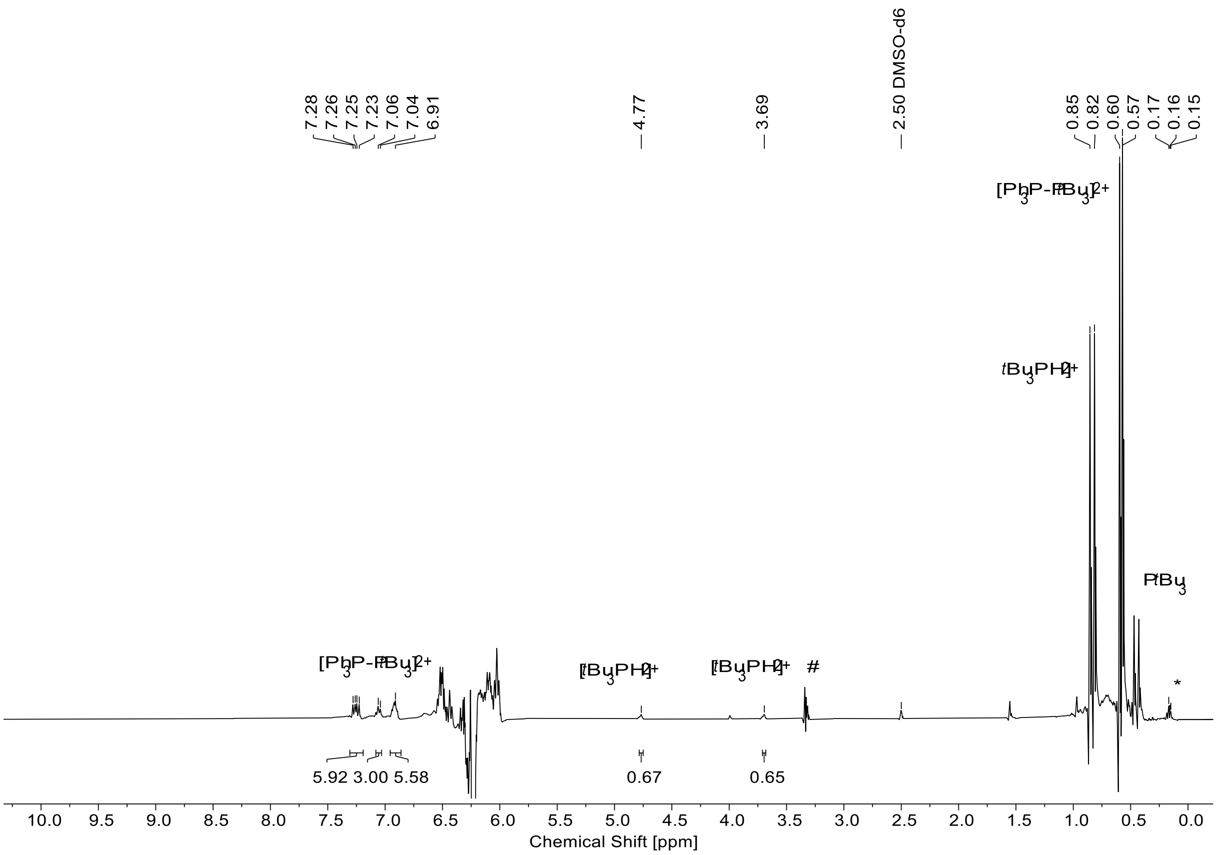

Figure S26. ^{1}H NMR spectrum (*o*DFB with DMSO-*d6* capillary, 162.0 MHz, D1 = 25 s, * = pentane, # = H_2O in capillary) showing the quantitative formation of [Ph_3P–P^tBu_3][Al^F]$_2$ (δ = 7.28, 7.26, 7.25, 0.59 ppm), [$^tBu_3P$–H]$^+$ (δ = 4.23, 0.83 ppm) and one equivalent of free PPh_3 (superimposed by *o*DFB).

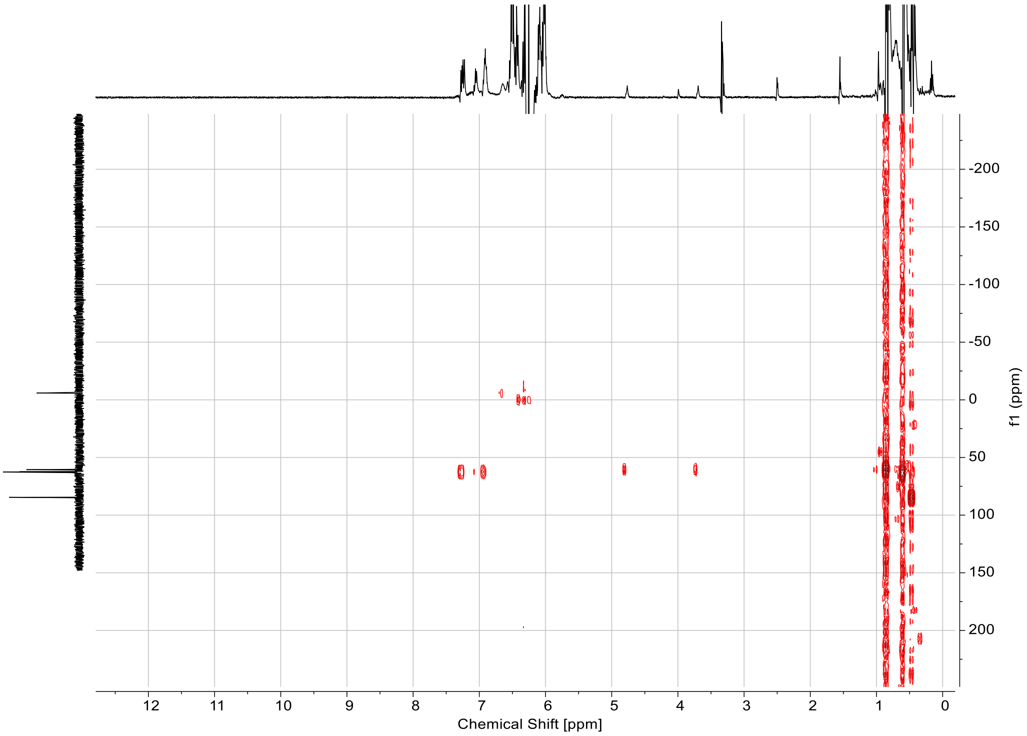

Figure S27. ^1H–^{31}P correlated HMBC NMR spectrum (oDFB with DMSO-*d6* capillary, 400.1/162.0 MHz).

2.5 Reaction of 1 with PMe3: [Me2PCH2PMe3][AlF], [Me3PH][AlF] and PPh3

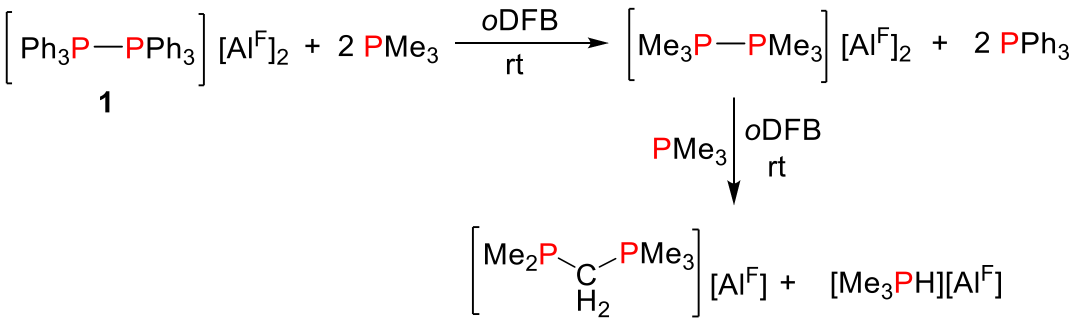

40μL of PMe3 (407 mM stock solution in *o*DFB, 2 equiv.) were added at room temperature to a *J. Young* NMR tube containing 20 mg of [Ph3P–PPh3][AlF]2 (**1**, 1.0 eq, 0.008 mmol) suspended in *o*DFB. A homogeneous pale-yellow solution formed, yet colorless precipitate formed again within 1 day. The crude reaction mixture was analyzed by NMR spectroscopies in *o*DFB with a DMSO-*d*6 capillary, which revealed the formation of $[Me_3P–PMe_3]^{2+}$ [11], and two equivalents of free PPh3.

Adding another equivalent of PMe_3 (20μL of 407 mM stock solution in *o*DFB) leads to the deprotonation of $[Me_3P–PMe_3]^{2+}$ to afford the rearrangement product $[Me_3PCH_2PMe_2]^+$.[11]

^{1}H NMR of $[Me_3P–PMe_3][$ (*o*DFB, DMSO-d_6 capillary, 400.1 MHz): δ = 2.32 (m, 18H, $[CH_3P−PCH_3][Al^F]_2$) ppm.

^{31}P{^{1}H} NMR of $[Me_3P–PMe_3][Al^F]_2$ (*o*DFB, DMSO-d_6 capillary, 162.0 MHz): δ = 26.4 (s, $[Me_3P−PMe_3][Al^F]_2$) ppm.

^{31}P{^{1}H} NMR of deprotonation product (*o*DFB, DMSO-d_6 capillary, 162.0 MHz): δ = 26.5 (d, $^2J_{PP}$ = 64 Hz, $[Me_2P−CH_2−PMe_3][Al^F]$), −52.7 (d, $^2J_{PP}$ = 64 Hz, $[PMe_2−CH_2−PMe_3][Al^F]$) ppm.

^{31}P{^{1}H} NMR (*o*DFB, DMSO-d_6 capillary, 162.0 MHz): δ = 26.5 (d, $^2J_{PP}$ = 64 Hz, $[Me_2P−CH_2−PMe_3]^+$), −2.6 (s, Me_3PH^+), −4.9 (s, PPh_3), −52.7 (d, $^2J_{PP}$ = 64 Hz, $[Me_2P−CH_2−PMe_3]^2$), -60.1 (PMe_3) ppm.

^{19}F NMR (*o*DFB, DMSO-d_6 capillary, 282.4 MHz): δ = –76.0 (s, –CF_3) ppm.

^{27}Al NMR (*o*DFB, DMSO-d_6 capillary, 78.2 MHz): δ = 34.5 (s, *Al*{$OC(CF_3)_3$}$_4$) ppm.

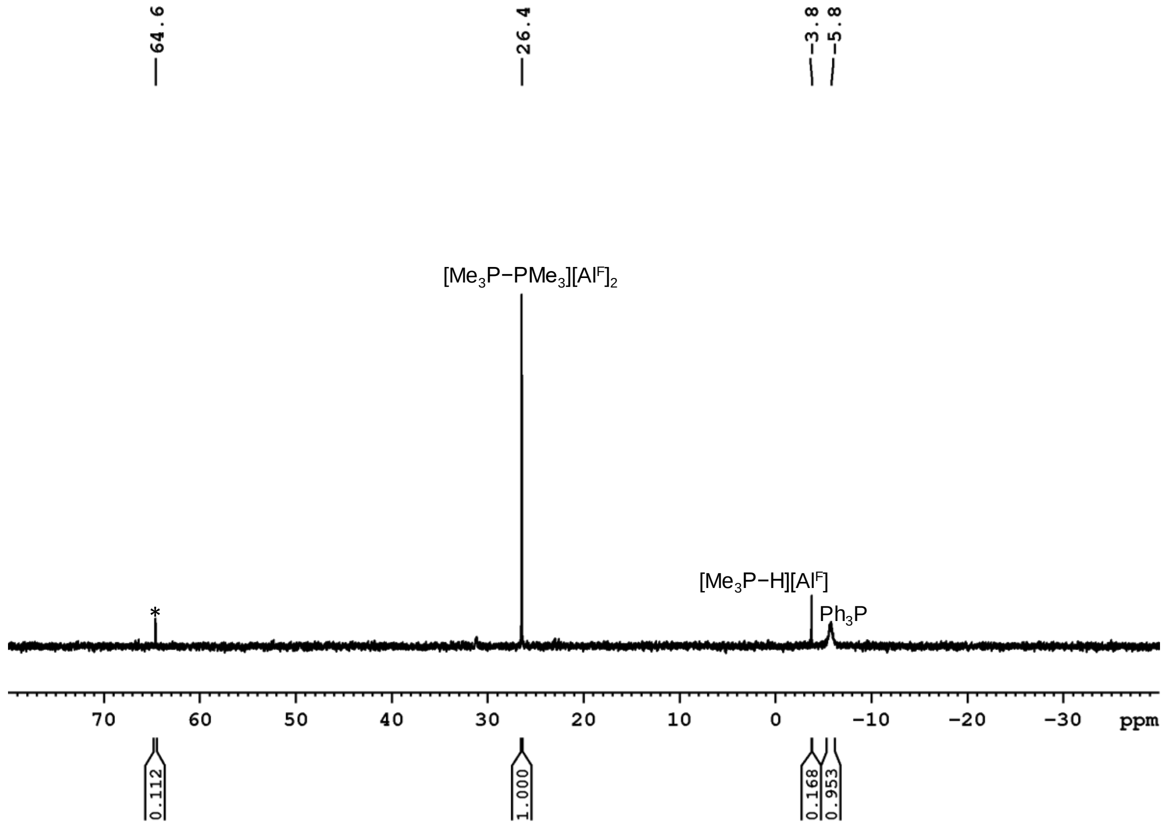

Figure S28. Crude $^{31}P\{^{1}H\}$ NMR spectrum obtained upon addition of 2 equiv. of PMe_3 to $[Ph_3P–PPh_3][Al^F]_2$ (oDFB with DMSO-d_6 capillary, 162.0 MHz, D1 = 30 s; *unidentified impurity).

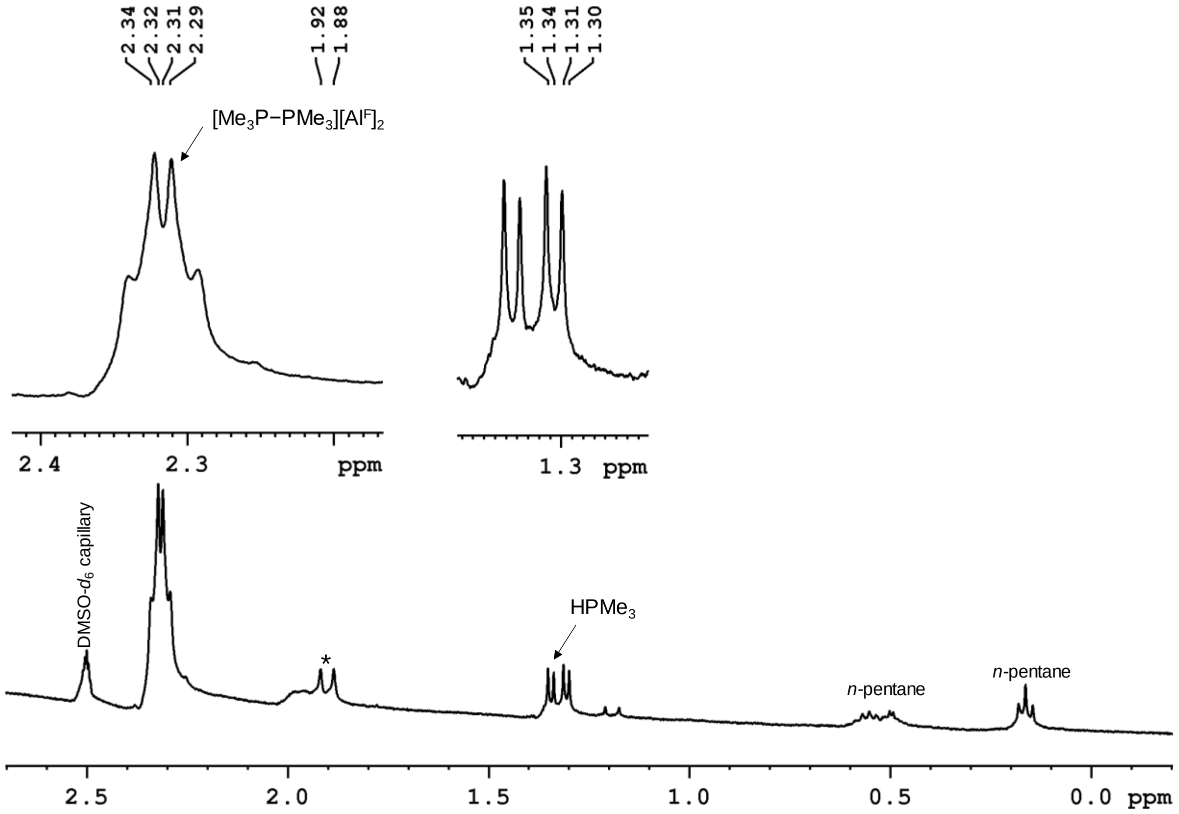

Figure S29. Crude ^{1}H NMR spectrum obtained upon addition of 2 equiv. of PMe_3 to $[Ph_3P–PPh_3][Al^F]_2$ (oDFB with DMSO-d_6 capillary, 400.1 MHz; *unidentified PMe_3-derivative).

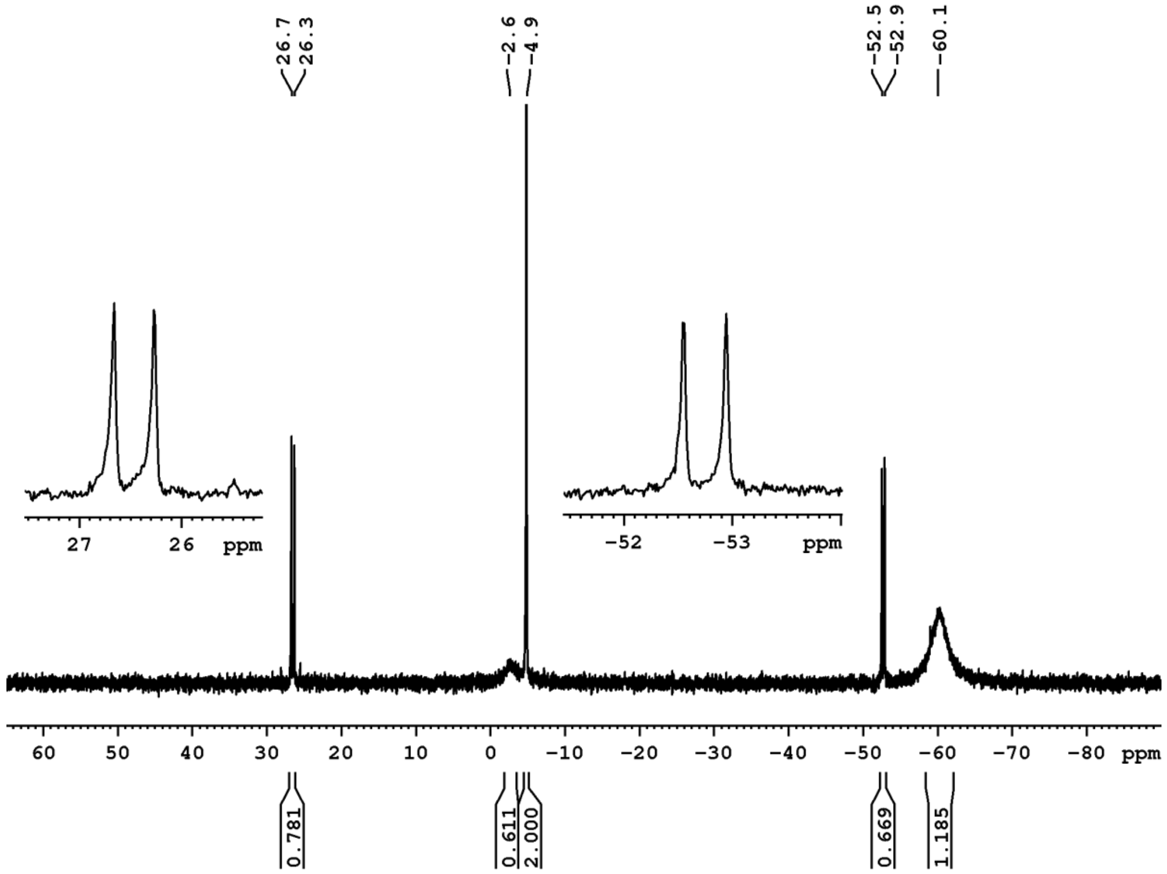

Figure S30. Crude $^{31}P\{^{1}H\}$ NMR spectrum obtained upon addition of PMe_3 to $[Ph_3P–PPh_3][Al^F]_2$ (*o*DFB with DMSO-d_6 capillary, 162.0 MHz, D1 = 30s).

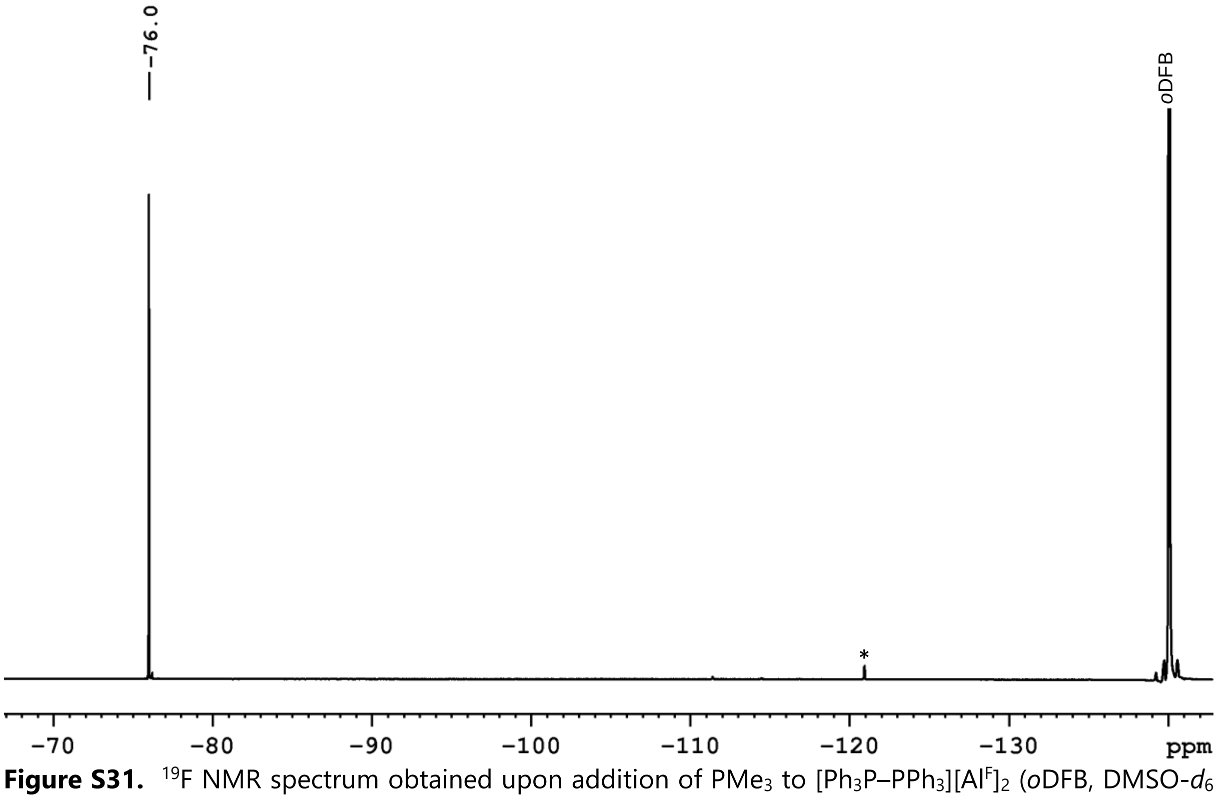

Figure S31. ^{19}F NMR spectrum obtained upon addition of PMe_3 to $[Ph_3P–PPh_3][Al^F]_2$ (*o*DFB, DMSO-d_6 capillary, 282.4 MHz; * trace impurity of *p*DFB in *o*DFB).

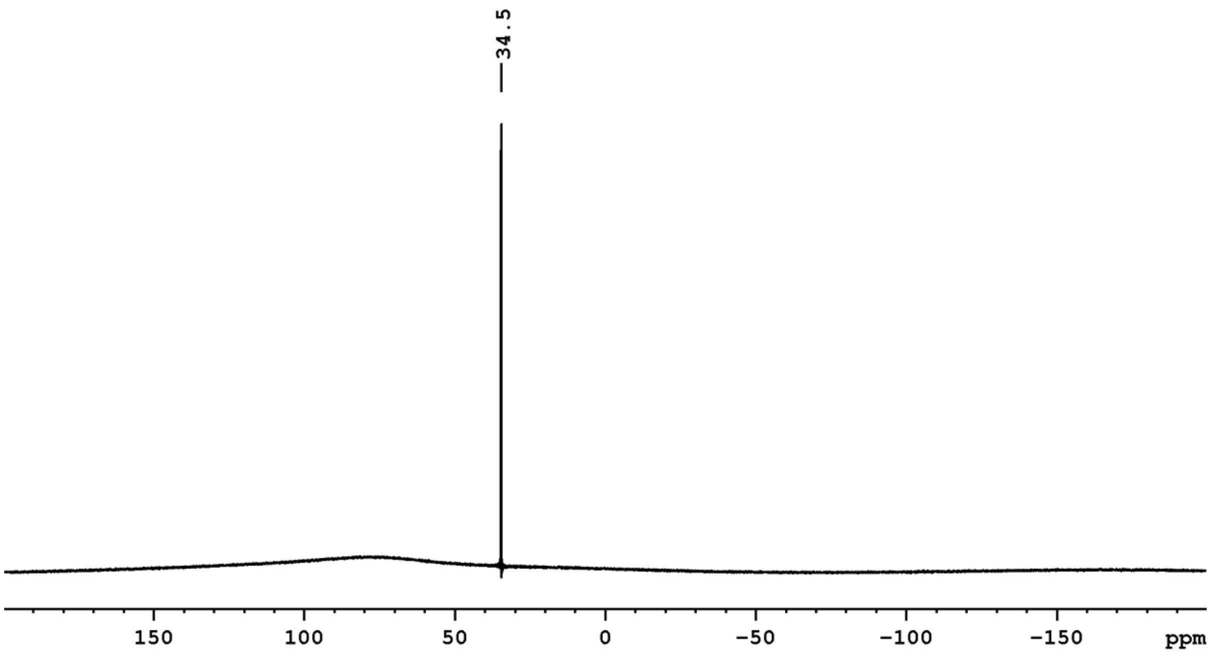

Figure S32. Crude ^{27}Al NMR spectrum obtained upon addition of PMe_3 to $[Ph_3P–PPh_3][Al^F]_2$ (*o*DFB, DMSO-d_6 capillary, 78.2 MHz).

2.6 Reaction of 1 with [PNP][OTf]: [Ph_3P–O–PPh_3][Al^F]$_2$, {(Ph_3P)$_2$O}(Tf)$_2$, and [PNP][Al^F]

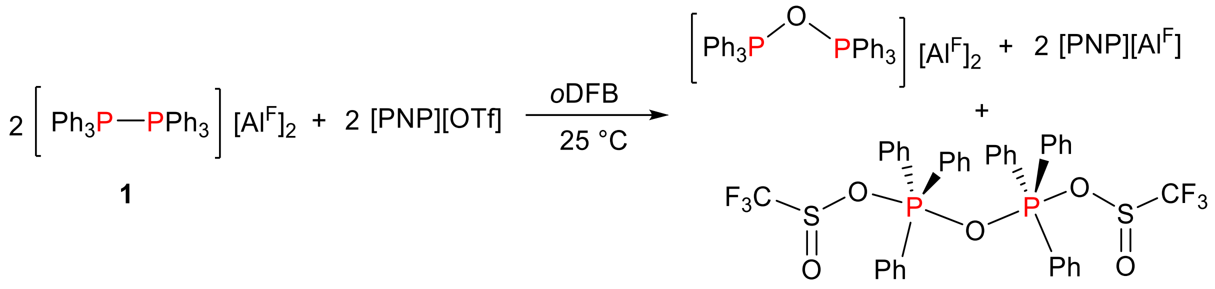

Inside the glovebox, [PNP][OTf] (11 mg, 0.02 mmol, 2 equiv.) was added to a suspension of 20 mg of [Ph_3P–PPh_3][Al^F]$_2$ (**1**, 1.0 equiv., 0.008 mmol) in 0.4 mL of *o*DFB in a *J. Young* NMR tube, upon which a faint yellow solution formed. The crude reaction mixture was analyzed by NMR spectroscopies and the signals for {(Ph_3P)$_2$O}(Tf)$_2$ assigned based on the literature.[12] **Note:** Over the course of further 8 h, [$FPPh_3$]$^+$ forms and the formation of a colorless precipitate is observed.

^{31}P{^{1}H} NMR (*o*DFB with DMSO-d_6 capillary, 121.5 MHz): δ = 75.4 (s, [Ph_3P–O–PPh_3][Al^F]$_2$), 43.3 (q, $^4J_{PF}$ = 4 Hz, {(Ph_3P)$_2$O}(Tf)$_2$), 20.5 (s, [PNP][Al^F]) ppm.

^{19}F NMR (*o*DFB with DMSO-d_6 capillary, 282.4 MHz): δ = –30.6 (d, $^4J_{FP}$ = 4 Hz, {(Ph_3P)$_2$O}(Tf)$_2$), −76.0 (s, Al^{F-}) ppm.

^{27}Al NMR (*o*DFB with DMSO-d_6 capillary, 104.2 MHz): δ = 34.5 (s, *Al*{OC(CF_3)$_3$}$_4$) ppm.

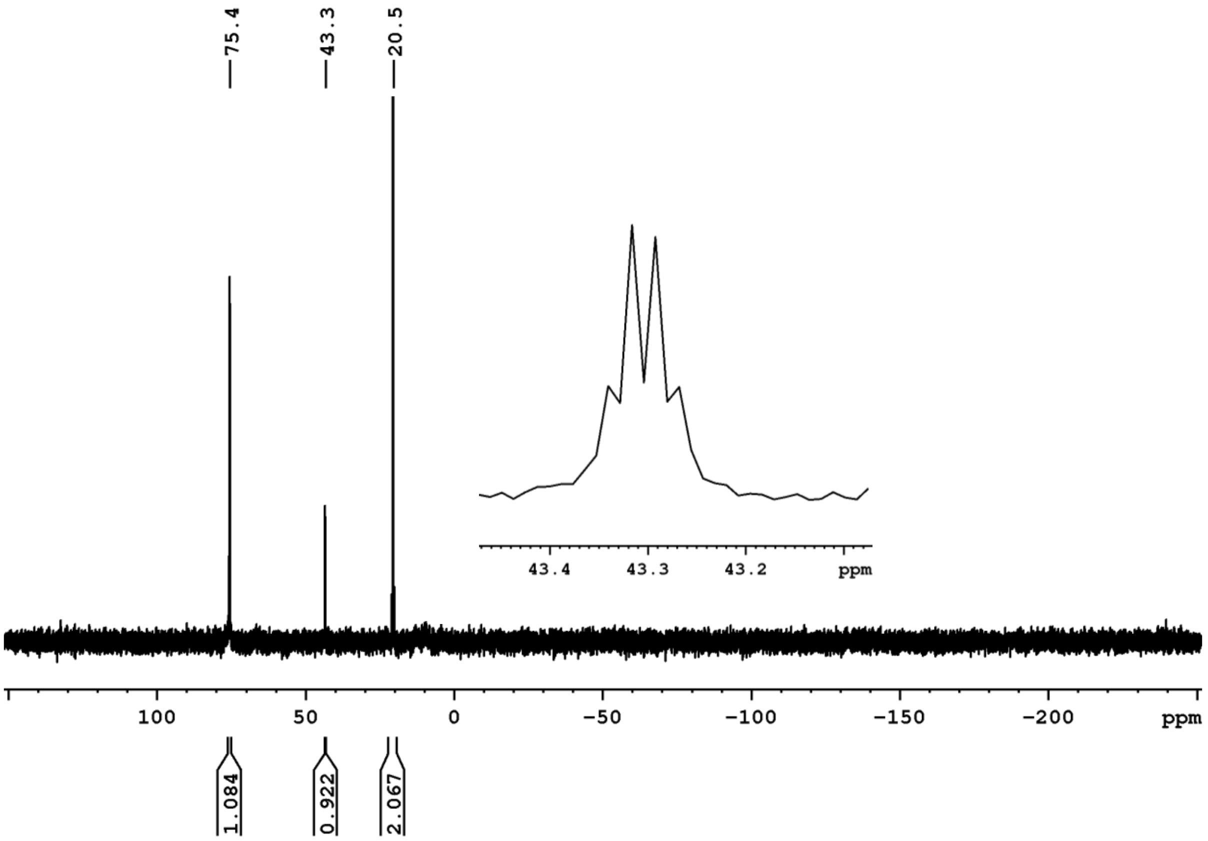

Figure S33. Crude $^{31}P\{^1H\}$ NMR spectrum obtained upon addition of [PNP][OTf] to $[Ph_3P–PPh_3][Al^F]_2$ (*o*DFB with DMSO-d_6 capillary, 121.5 MHz).

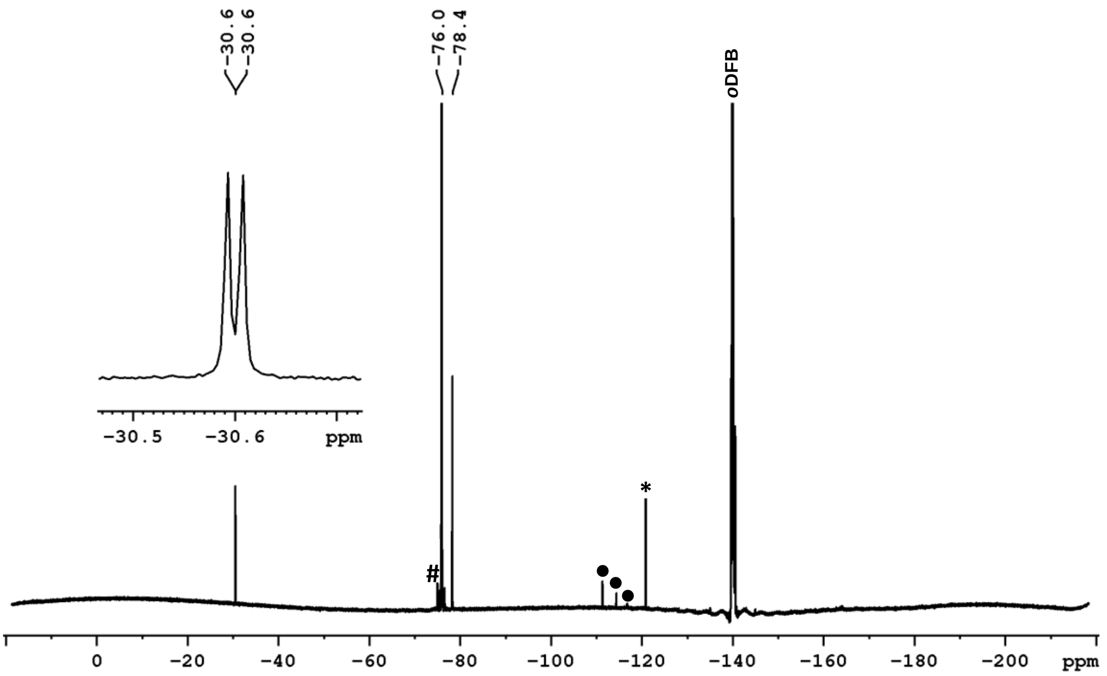

Figure S34. Crude ^{19}F NMR spectrum obtained upon addition of [PNP][OTf] to $[Ph_3P–PPh_3][Al^F]_2$ (*o*DFB with DMSO-d_6 capillary, 121.5 MHz; #trace impurity; ● unidentified impurity in *o*DFB; * trace impurity of *p*DFB in *o*DFB).

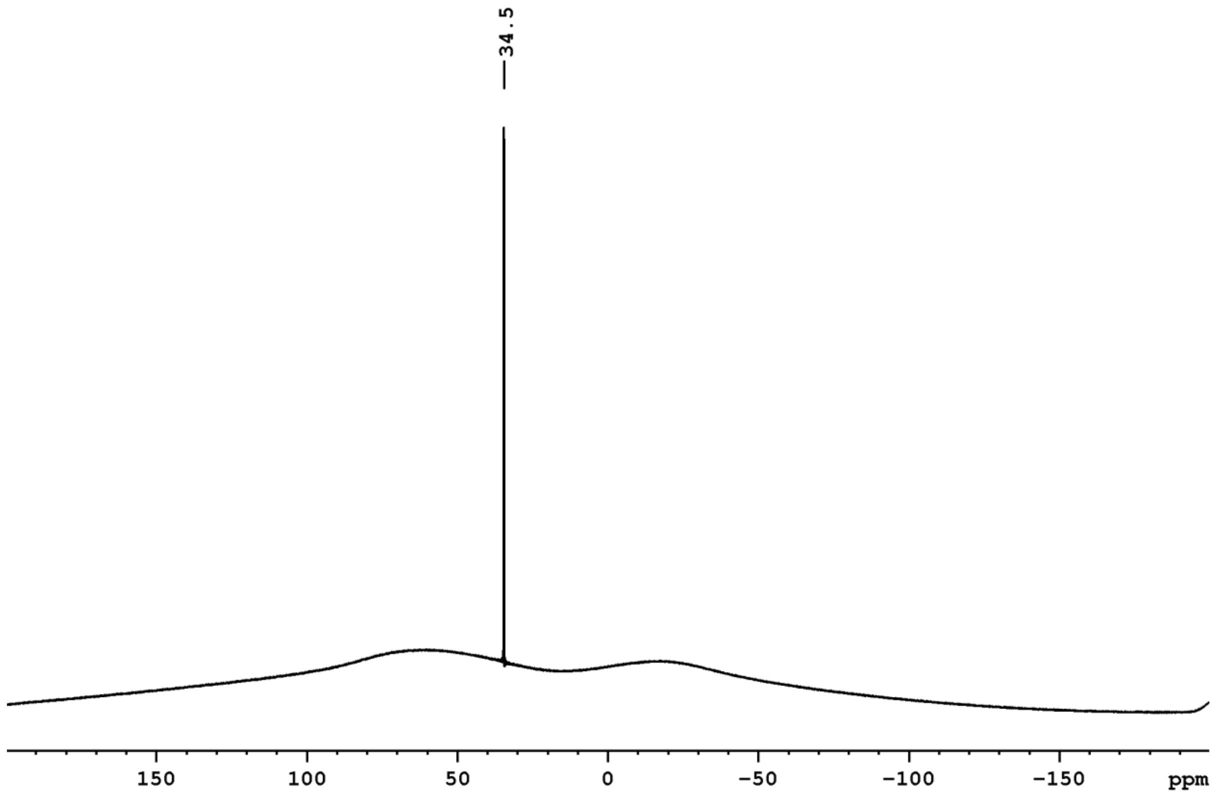

Figure S35. Crude ^{27}Al NMR spectrum obtained upon addition of [PNP][OTf] to $[Ph_3P–PPh_3][Al^F]_2$ (*o*DFB with DMSO-d_6 capillary, 104.2 MHz).

2.7 Reaction of 1 with TolCOOH

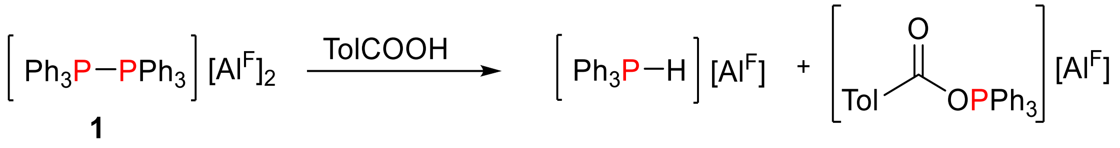

A stock solution (5 mg in 5 mL) of toluic acid (300 µL, 0.3 mg, 0.002 mmol, 0.5 eq.) in *o*DFB was added to a suspension of **1** (10 mg, 0.004 mmol, 1 eq) in *o*DFB (200 µL). The ^{31}P NMR spectroscopic analysis indicated the instantaneous and clean formation of a stoichiometric mixture of $[Ph_3PH]^+$ and a signal at 62.4 ppm, which was assigned to the acyloxyphosphonium salt based on the literature.[13] Heating to 90 °C for 90 minutes did not lead to a substantial change of the spectroscopic signature.

The solution was treated with KHMDS (4 mg, 0.020 mmol, 5 eq), which resulted in the generation of PPh_3 and $OPPh_3$, thus further corroborating the previous formation of the mixed anhydride.

^{31}P{^{1}H} NMR (*o*DFB with DMSO-d_6 capillary, 162.0 MHz): δ = 62.4 (s, $[TolCOOPPh_3]^+$, 7.7 (s, $[Ph_3PH]^+$ ppm.

^{31}P{^{1}H} NMR upon addition of KHMDS (*o*DFB with DMSO-d_6 capillary, 162.0 MHz): δ = 29.8 (s, $OPPh_3$), –5.9 (s, PPh_3) ppm.

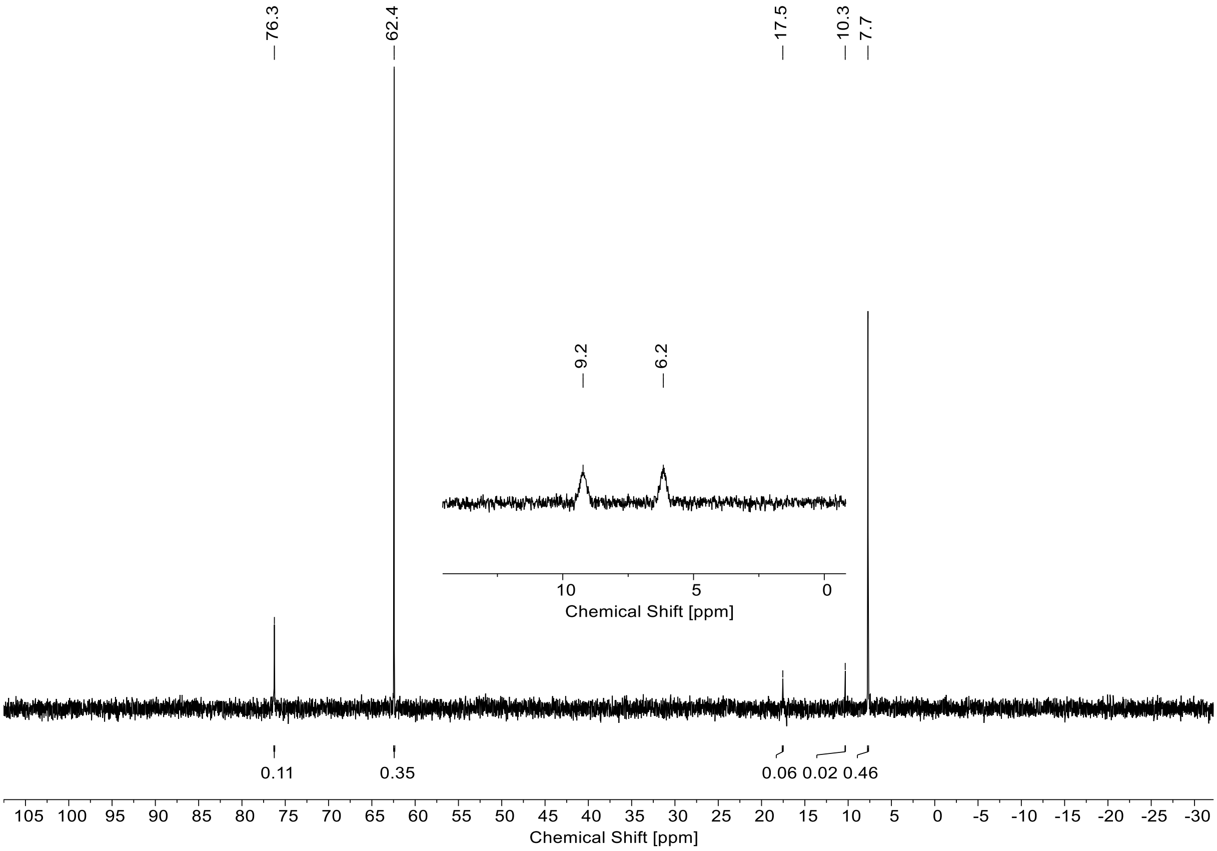

Figure S36. Crude $^{31}P\{^{1}H\}$ NMR spectrum (*o*DFB with DMSO-d_6 capillary, 162.0 MHz, D1 = 30 s) with inset ^{31}P NMR spectrum (*o*DFB with DMSO-d_6 capillary, 162.0 MHz) revealing the intermediate formation of $[TolCOOPPh_3]^+$ (δ = 62.4 ppm) as well as $[Ph_3PH]^+$ (δ = 7.7 ppm). The signals at δ = 76.3 ppm are assigned to $[Ph_3POPPh_3]^{2+}$ due to adventitious presence of H_2O; δ = 17.6 ppm to residual **1**, δ = 10.3 ppm to an unidentified impurity.

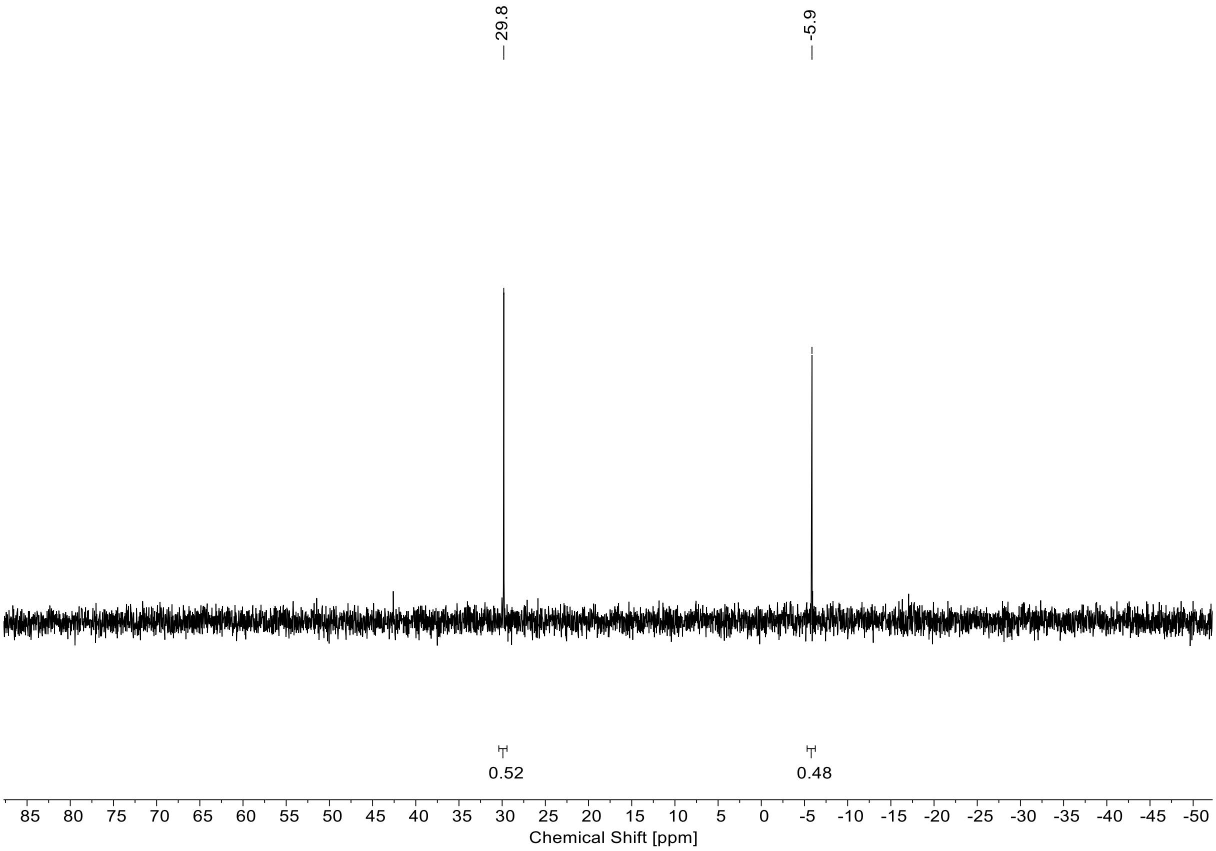

Figure S37. $^{31}P\{^1H\}$ NMR spectrum (*o*DFB with DMSO-d_6 capillary, 162.0 MHz, D1 = 30 s) obtained upon the addition of KHMDS, revealing the formation of $OPPh_3$ (δ = 29.8 ppm) as well as PPh_3 (δ = −5.9 ppm).

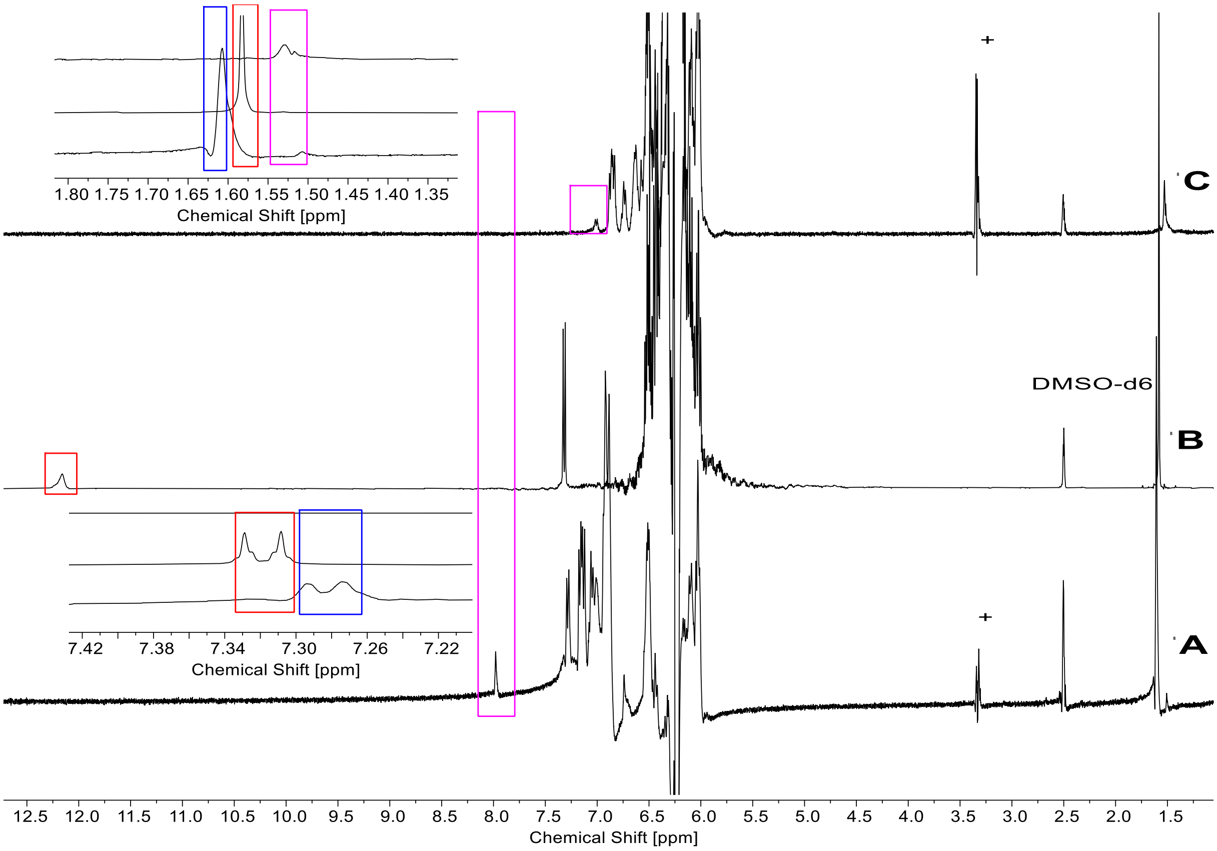

Figure S38. Stacked ^{1}H NMR spectra (*o*DFB with DMSO-d_6 capillary, 400.1 MHz) prior to the deprotonation with KHMDS (A), reference spectrum of TolCOOH (B), and spectrum obtained after deprotonation with KHMDS (C). The blue color highlights the high-field shift of resonances in the aromatic region and the low-field shift of the resonances corresponding to the methyl group in TolCOOH, pink assigns the $[Ph_3PH]^+$ proton and the high-field shifted resonances of the methyl group as a result of deprotonation of $[Ph_3PH]^+$ due to the release of Ph_3PO resulting from nucleophilic attack of $^-N(SiMe_3)_2$ at the acyloxyphosphonium salt.

2.8 Reaction of 1 with $OPEt_3$: $OPPh_3$, PPh_3 and $[Et_3P{-}O{-}PEt_3][Al^F]_2$

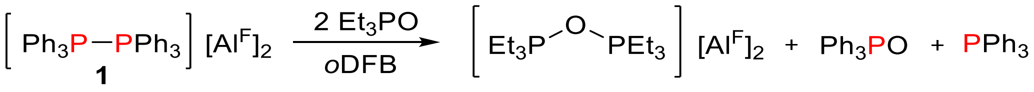

In a *J. Young* capped NMR tube, **1** (18 mg, 0.007 mmol, 1 eq) was suspended in *o*DFB (0.4 mL) and $OPEt_3$ (3 mg, 0.021 mmol, 3 eq) was added, upon which a colorless solution formed. The sample was analyzed by NMR spectroscopies. The volatiles were removed *in vacuo*, the oily residue was washed with benzene (3 × 0.5 mL) and *n*-pentane (2 × 0.5 mL), and then redissolved in CD_3CN for further NMR spectroscopic analysis.

$^{31}P\{^1H\}$ NMR (CD_3CN, 162.0 MHz): δ = 93 (br s, 2P, $[Et_3P{-}O{-}PEt_3]^{2+}$), ppm.

^1H NMR (CD_3CN, 400.1 MHz): δ =2.18 (m, 12H PCH_2CH_3), 1.20 (dtr, 18H $^3J_{HP}$ = 19 Hz, $^3J_{HH}$ = 8 Hz, PCH_2CH_3) ppm.

$^{13}C\{^1H\}$ NMR (CD_3CN, 100.6 MHz): δ = 17.8 (d, $^2J_{CP}$ = 59 Hz, PCH_2CH_3), 4.6 (d, $^3J_{CP}$ = 5 Hz, PCH_2CH_3) ppm.

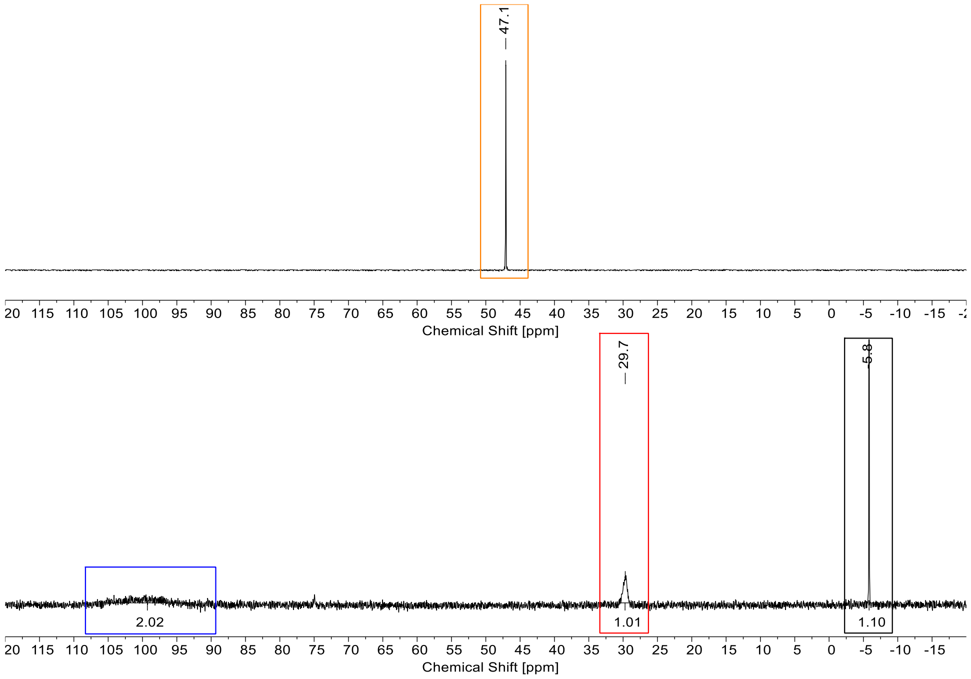

Figure S39. $^{31}P\{^1H\}$ NMR spectrum (*o*DFB with DMSO-d_6 capillary, 162.0 MHz) of the crude reaction of **1** with $OPEt_3$ (bottom), stacked with a reference spectrum of $OPEt_3$ (top). Color coding: blue, $[Et_3P{-}O{-}PEt_3]^{2+}$; red, $OPPh_3$; orange, $OPEt_3$; black, PPh_3.

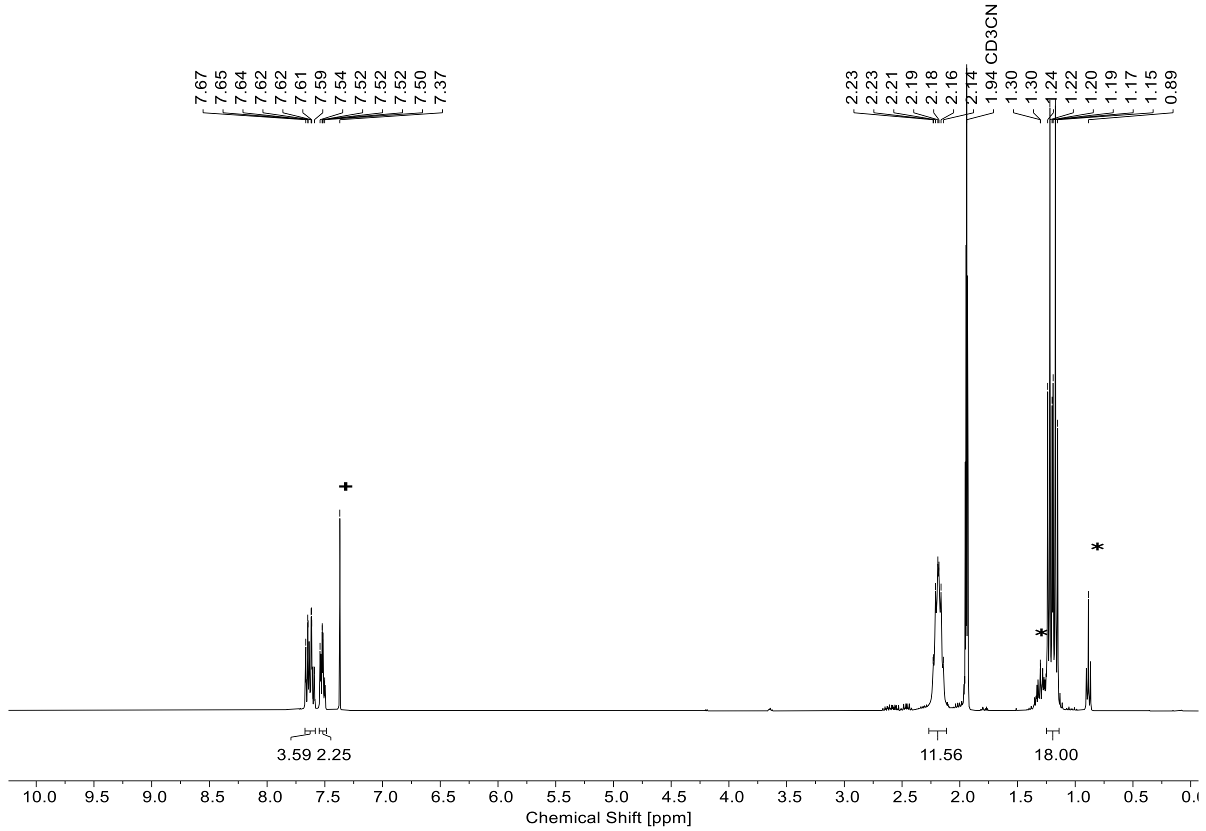

Figure S40. ^{1}H NMR spectrum obtained after washing with pentane (*) and benzene (+) (CD_3CN, 400.1 MHz).

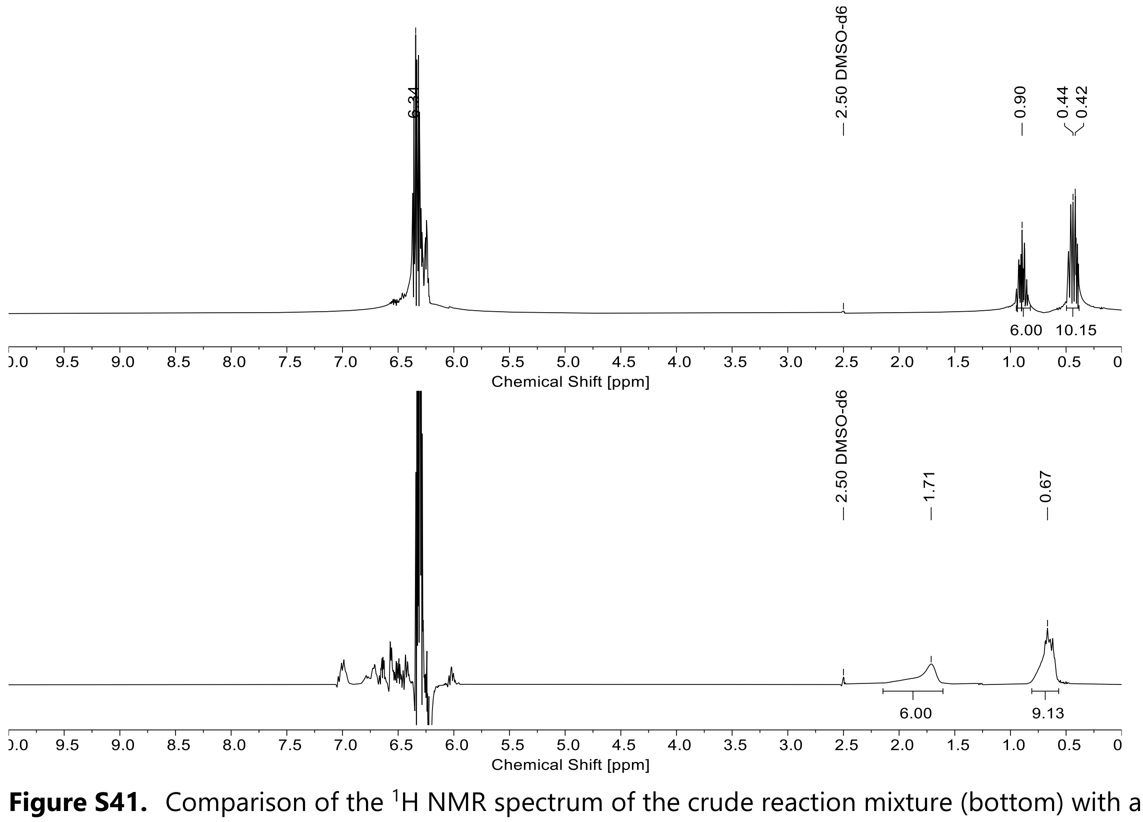

Figure S41. Comparison of the ^{1}H NMR spectrum of the crude reaction mixture (bottom) with a spectrum of $OPEt_3$ (*o*DFB with DMSO-d_6 capillary, 400.1 MHz; top).

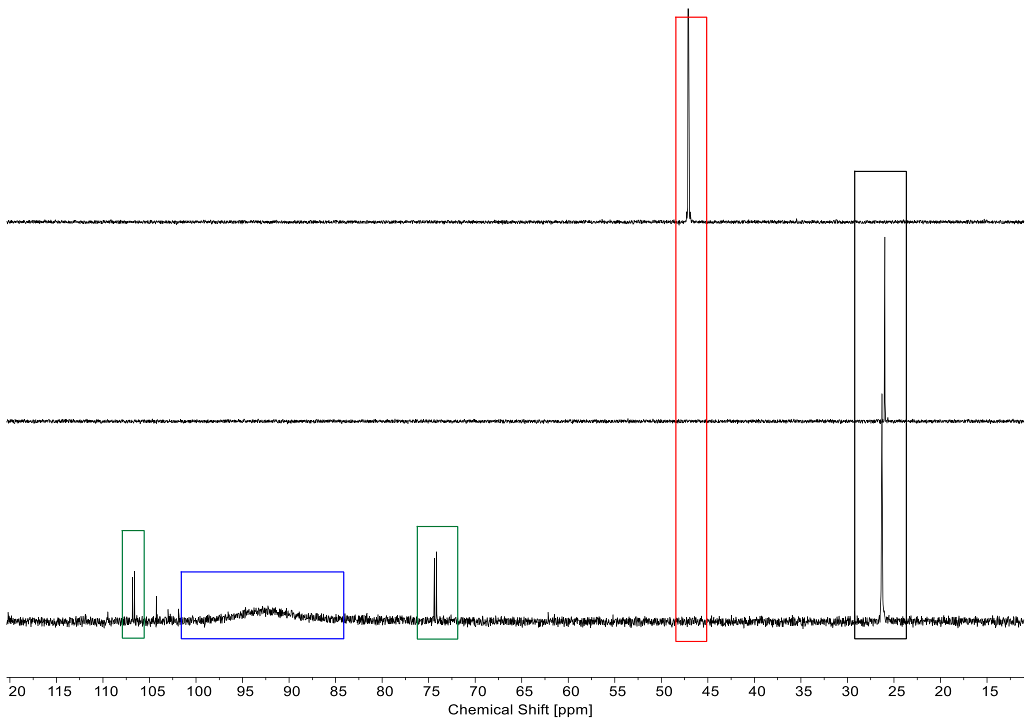

Figure S42. $^{31}P\{^{1}H\}$ NMR spectrum (CD_3CN, 162.0 MHz) of the reaction of **1** with $OPEt_3$ after washing with pentane and benzene (bottom), stacked with the reference spectra of $OPPh_3$ (middle) and $OPEt_3$ (top). Color coding: blue, $[Et_3P{-}O{-}PEt_3]^{2+}$; red, $OPEt_3$; black, $OPPh_3$; green, minor side product; tentative GUTMANN-BECKETT adduct $[Ph_3P{-}O{-}PEt_3]^{2+}$

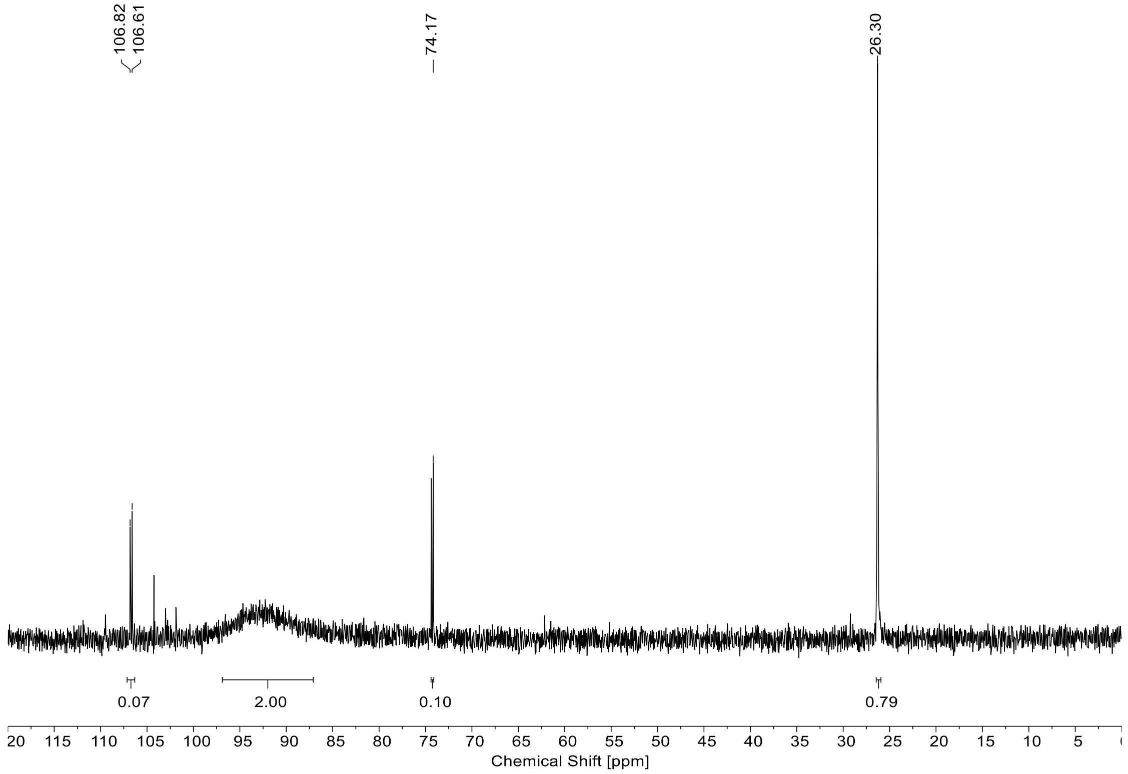

Figure S43. $^{31}P\{^{1}H\}$ NMR spectrum (CD_3CN, 162.0 MHz) of the reaction of **1** with $OPEt_3$ after washing with pentane and benzene, showing minor amounts of the tentative GUTMANN-BECKETT adduct $[Ph_3P{-}O{-}PEt_3]^{2+}$ (δ = 106.7 ppm; δ = 74.2 ppm) as well as residual $OPPh_3$ (δ = 26.3 ppm).

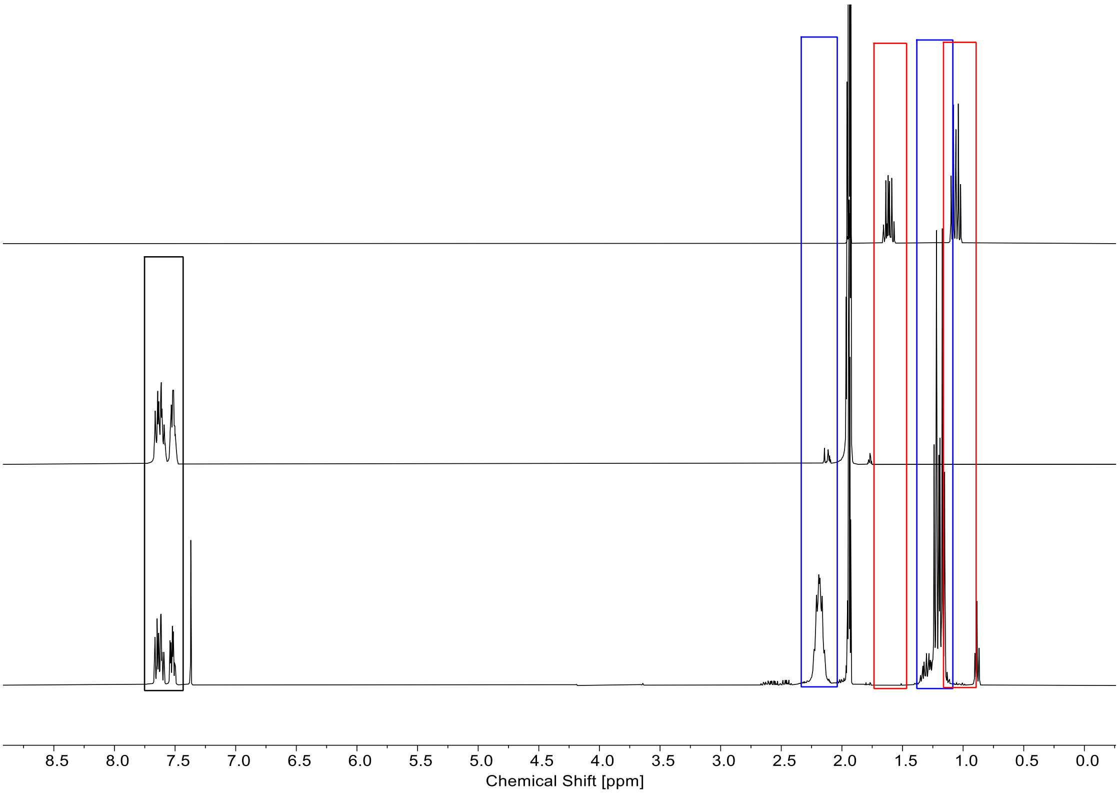

Figure S44. ^{1}H NMR spectrum (CD_3CN, 162.0 MHz) of the reaction of **1** with $OPEt_3$ after washing with pentane and benzene, stacked with reference spectra of $OPPh_3$ (middle) and $OPEt_3$ (top). Color coding: blue, $[Et_3P{-}O{-}PEt_3]^{2+}$; red, $OPEt_3$; black, $OPPh_3$.

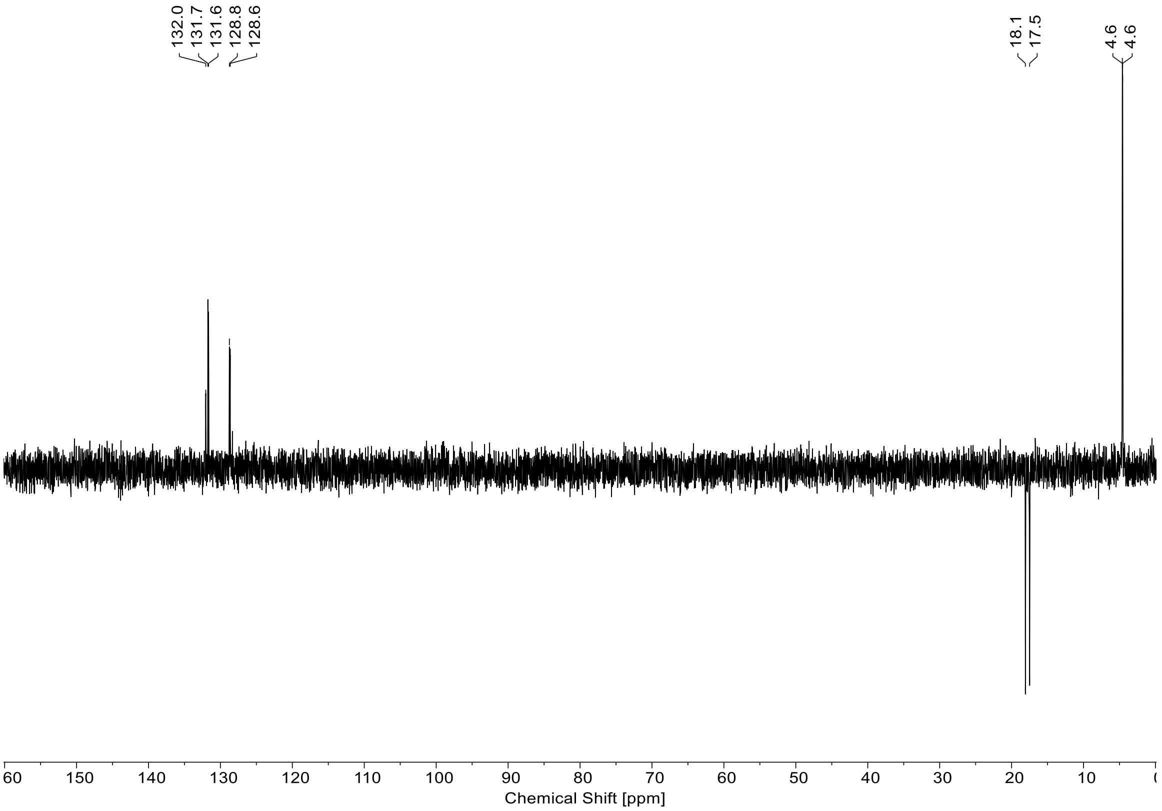

Figure S45. DEPT-135 NMR spectrum obtained after washing with benzene and pentane (CD_3CN, 100.6 MHz).

2.9 Absence of a Reaction of 1 with *p*-Fluorobenzonitrile

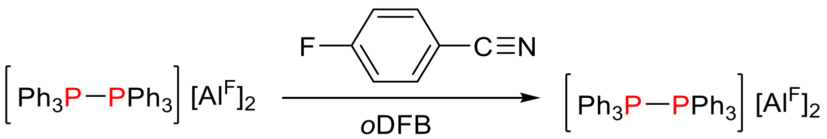

1

In a *J. Young* capped NMR tube containing a suspension of **1** (30 mg, 0.012 mmol, 1 equiv.) in *o*DFB, *para*-fluorobenzonitrile was added (6 mg, 0.0149, 4 equiv.), yet the $^{31}P\{^1H\}$ and ^{19}F NMR spectroscopic analysis revealed the absence of a reaction, even upon heating to reflux for 6 h.

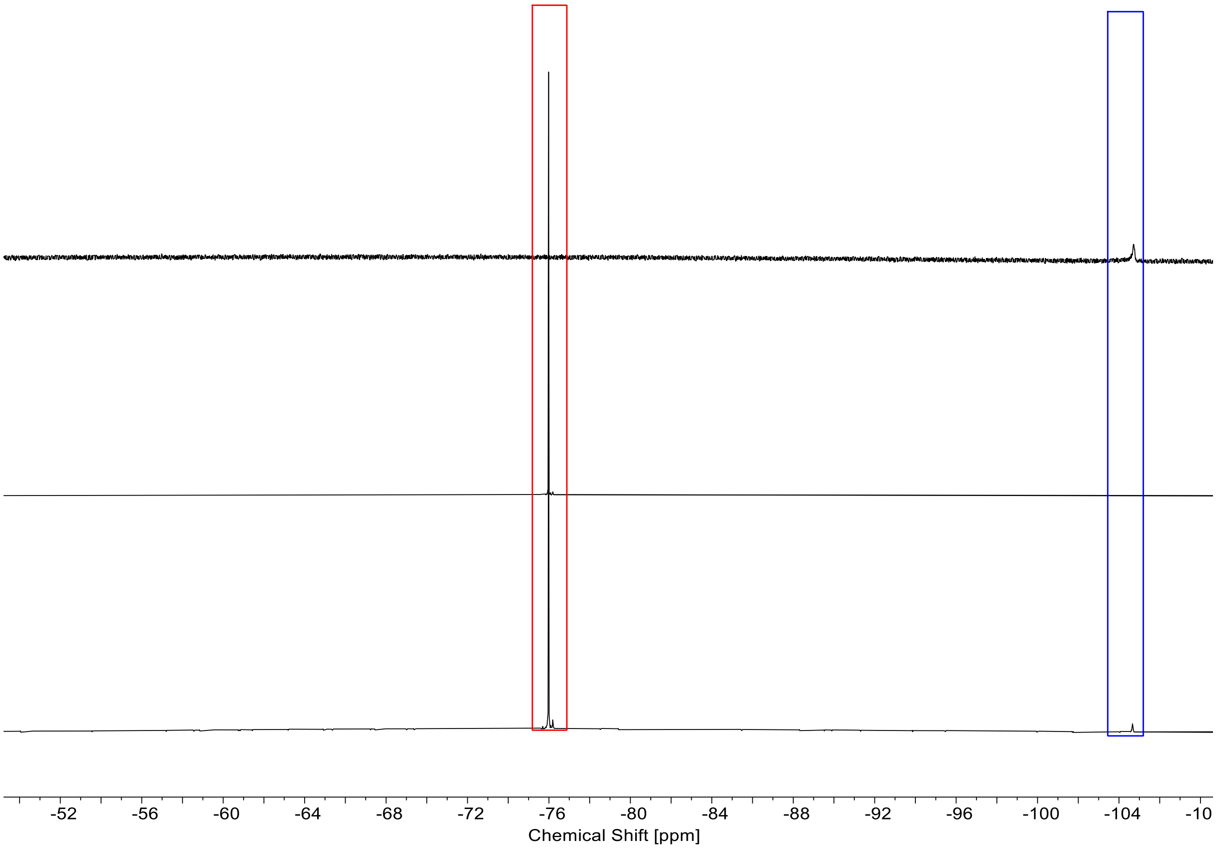

Figure S46. ^{19}F NMR spectra (*o*DFB with DMSO-d_6 capillary, 282.4 MHz) obtained after the addition of *para*-fluorobenzonitrile to a suspension of **1** in *o*DFB (bottom), and reference spectra of a suspension of **1** in *o*DFB (middle) and *para*-fluorobenzonitrile (top) in *o*DFB.

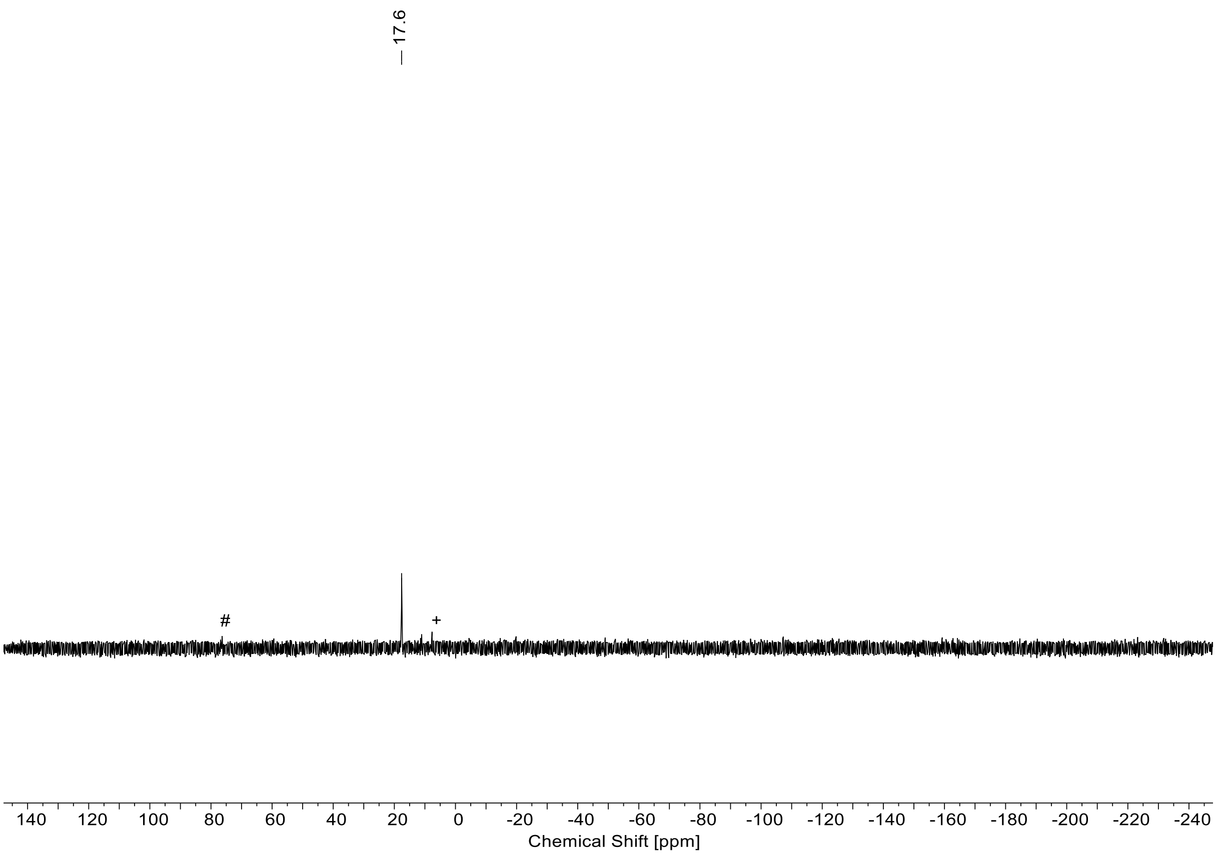

Figure S47. ^{31}P NMR spectrum (*o*DFB with DMSO-d_6 capillary, 162.0MHz, # = $[Ph_3P–O–PPh_3]^{2+}$, + = $[PPh_3H]^+$) after addition of *para*-fluorobenzonitrile to a suspension of **1** in *o*DFB and heating to reflux.

2.10 Reaction of 1 with $SbCl_6^-$ (3)

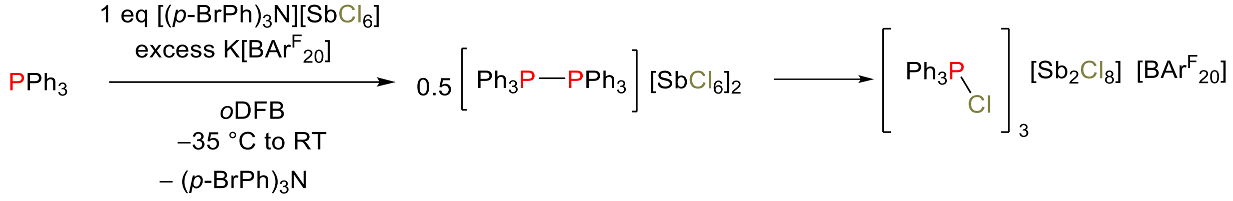

In a *J. Young* NMR tube, 32 mg of PPh_3 (1.0 eq, 0.122 mmol) were dissolved in 0.9 mL of *o*DFB at –35 °C and 100 mg of [(*p*-BrPh)$_3$N][$SbCl_6$] (1.0 eq, 0.122 mmol) were added. The color of the solution changed instantaneously from deep blue to green and then light-yellow. A colorless precipitate formed and the ^{31}P NMR spectroscopic analysis indicated the presence of $[Ph_3PCl]^+$ in the supernatant. Excess K[BAr^F_{20}] was added to the suspension, upon which the precipitate dissolved. The NMR spectroscopic analysis revealed the quantitative conversion of triphenylphosphine to $[Ph_3PCl]^+$. Few colorless, block-shaped crystals of $[PPh_3Cl]_2[Sb_2Cl_8]$ can be obtained directly from the *o*DFB solution at ambient temperature.

$^{11}B\{^1H\}$ NMR (*o*DFB with DMSO-d_6 capillary, 96.2 MHz): δ = –16.7 (s, $B(C_6F_5)_4$) ppm.

^{19}F NMR (*o*DFB with DMSO-d_6 capillary, 282.4 MHz): δ = –133.05 (br s, –C*F*), –164.37 (br s, –C*F*)*, –168.12 (br s, –C*F*)* ppm. *Poorly resolved triplet resonances.

$^{31}P\{^1H\}$ NMR (*o*DFB with DMSO-d_6 capillary, 121.5 MHz): δ = 65.30 (s, Ph_3P) ppm.

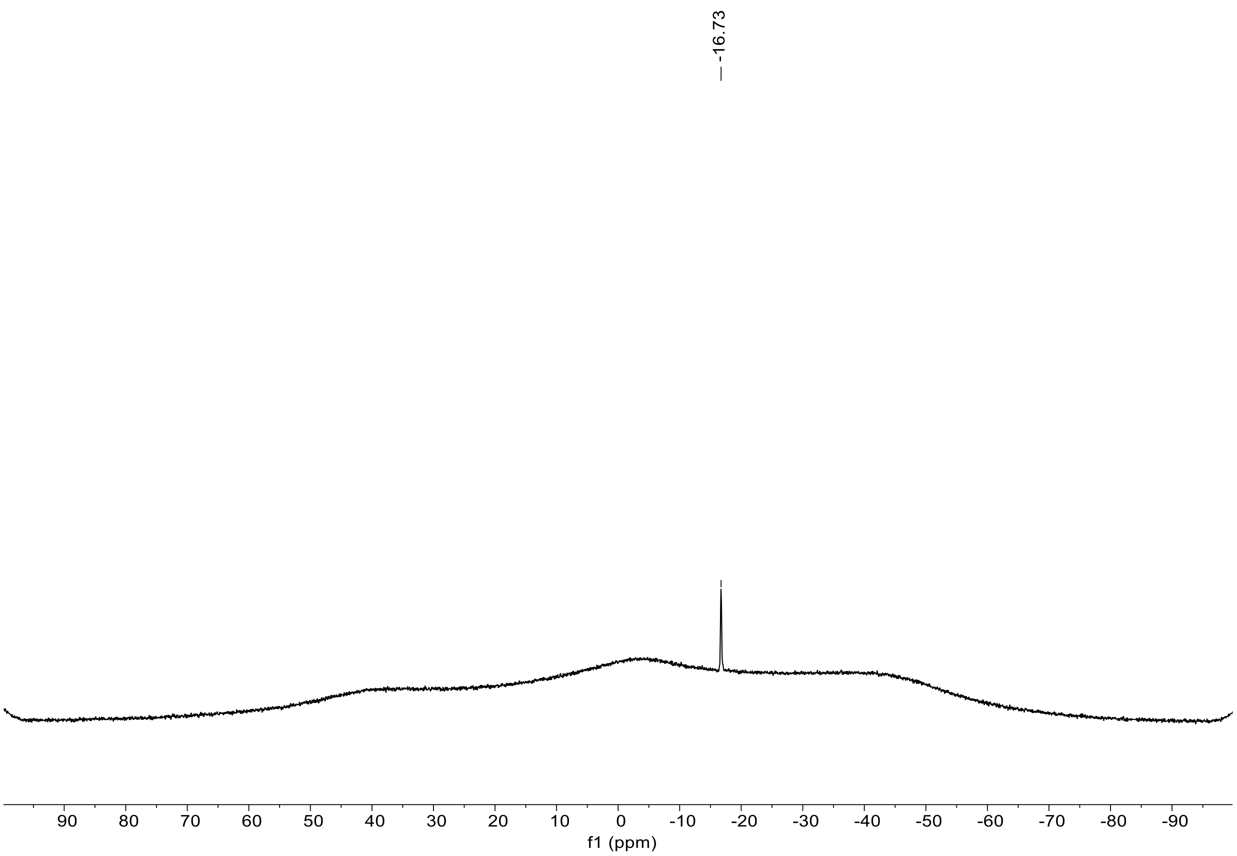

Figure S48. $^{11}B\{^1H\}$ NMR spectrum of $[Ph_3PCl]_3[BArF_{20}][Sb_2Cl_8]$ (*o*DFB with DMSO-d_6 capillary, 96.2 MHz).

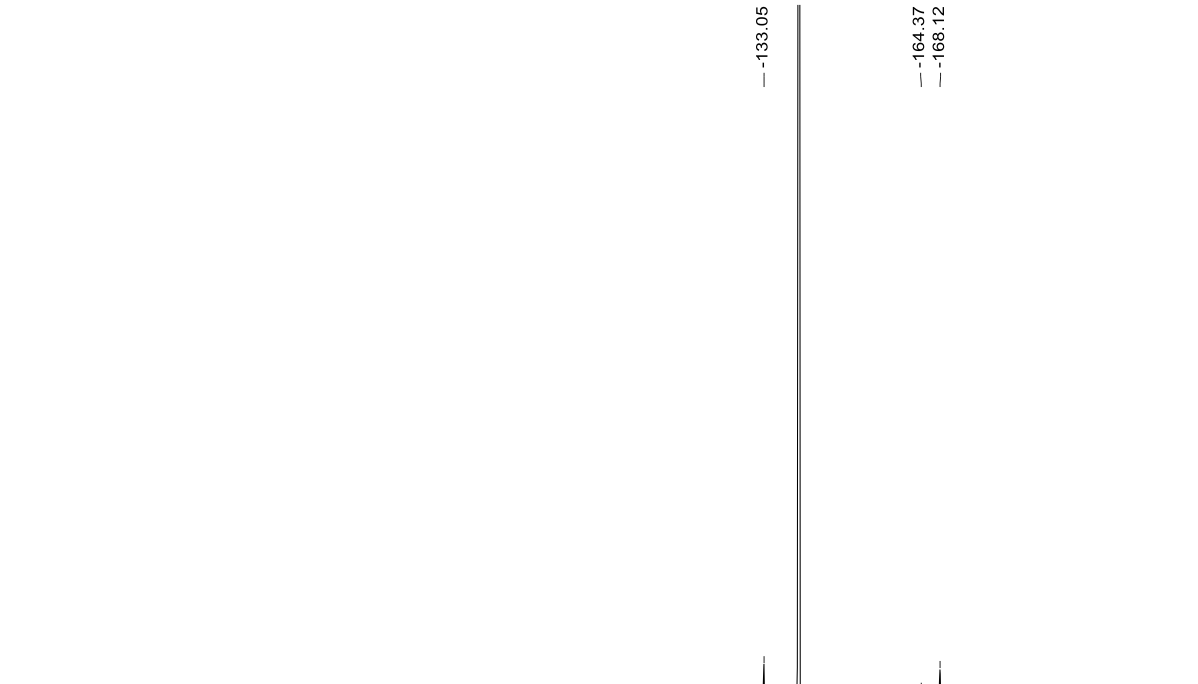

Figure S49. ^{19}F NMR spectrum of $[PPh_3Cl]_3[BArF_{20}][Sb_2Cl_8]$ (*o*DFB with DMSO-d_6 capillary, 282.4 MHz).

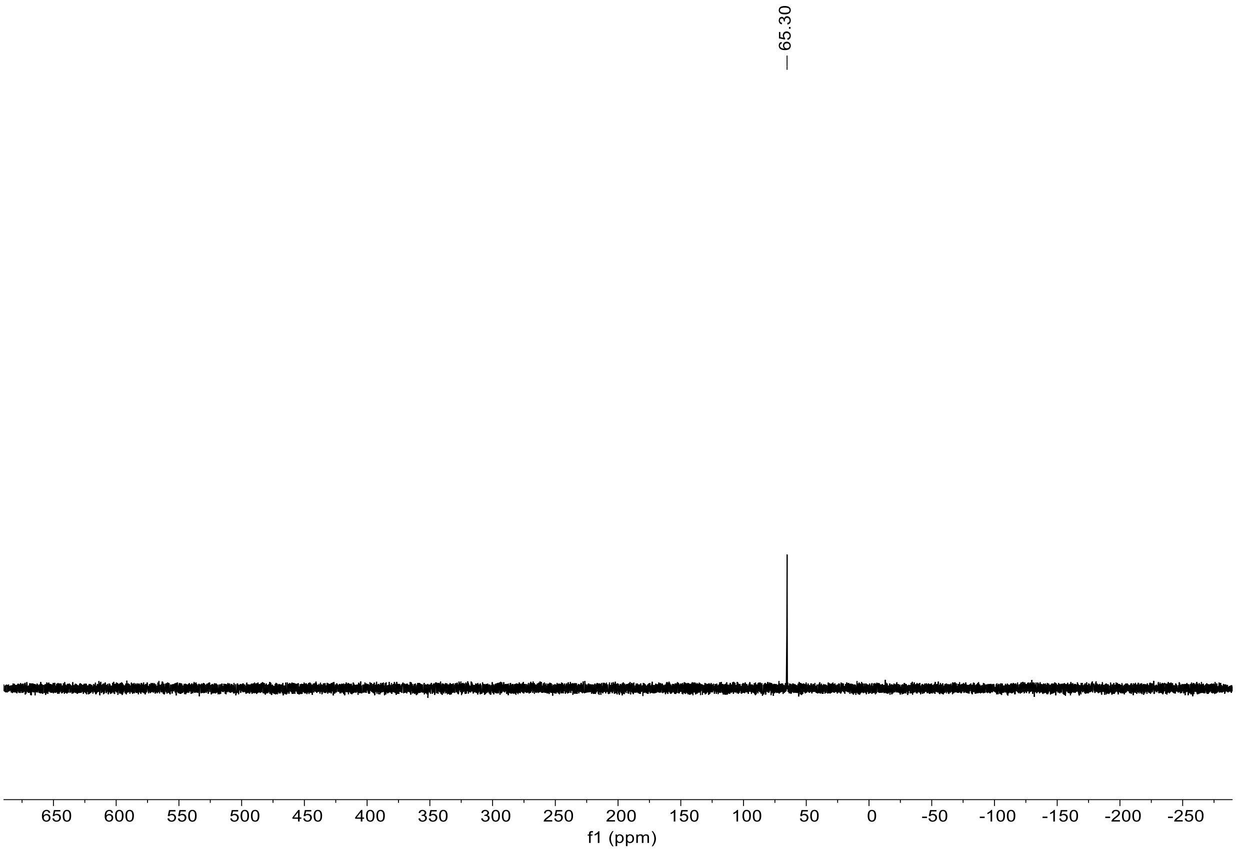

Figure S50. $^{31}P\{^{1}H\}$ NMR spectrum of $[PPh_3Cl]_3[BArF_{20}][Sb_2Cl_8]$ (*o*DFB with DMSO-d_6 capillary, 121.5 MHz).

2.11 Reaction of 1 with [Na(222crypt)][BF_4]: [$FPPh_3$][Al^F]

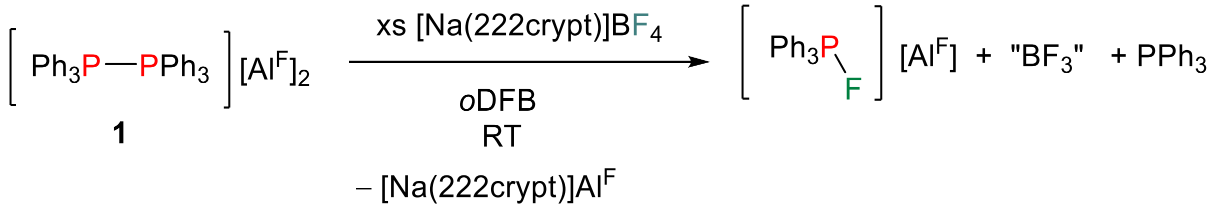

Freshly prepared [Na(222crypt)][BF_4] (1.5 eq, 0.0036) in TFB was added to a *J. Young* NMR tube containing 6 mg of [Ph_3P–PPh_3][Al^F]$_2$ (**1**, 1.0 eq, 0.0024 mmol). The solution changes to a pale-yellow color. Conversion of compound [Ph_3P–PPh_3][Al^F]$_2$ to [$FPPh_3$][Al^F] and PPh_3 is quantitative. Note: Over the course of three days, [$FPPh_3$][Al^F] further converts to [F_2PPh_3].

$^{11}B\{^1H\}$ NMR (*o*DFB with DMSO-d_6 capillary, 96.2 MHz): δ = 0.79 (br, $\mathit{B}F_3$ and/or $\mathit{B}F_3$ adduct), –0.6 (s, xsNa$\mathit{B}F_4$) ppm.

$^{31}P\{^1H\}$ NMR (*o*DFB with DMSO-d_6 capillary, 121.5 MHz): δ = 94.7 (d, $^+PPh_3\mathit{F}$), –5.8 (s, $\mathit{P}Ph_3$) ppm.

^{19}F NMR (*o*DFB with DMSO-d_6 capillary, 282.4 MHz): δ = –130.1 (d, $^+PPh_3\mathit{F}$), –76.9 (s, Al^F) ppm.

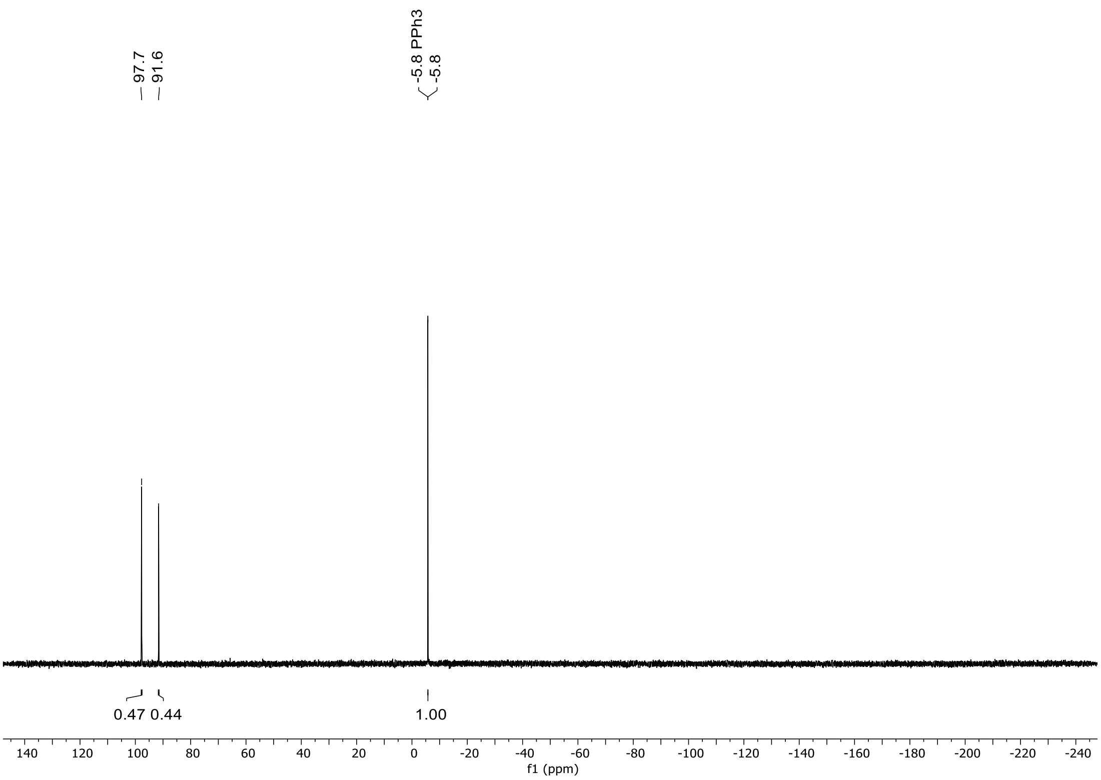

Figure S51. $^{31}P\{^{1}H\}$ NMR spectrum after conversion of $[Ph_3P–PPh_3][Al^F]_2$ with $[Na(222crypt)][BF_4]$ (*o*DFB with DMSO-d_6 capillary, 121.5 MHz, D1 = 30 s).

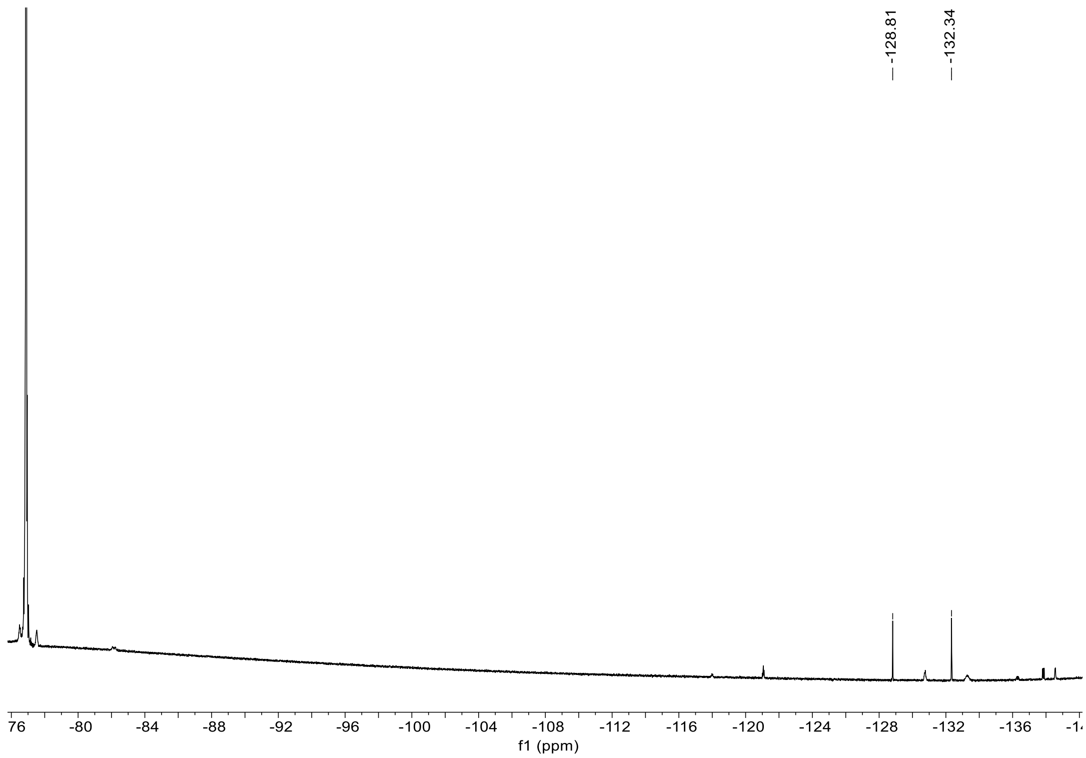

Figure S52. ^{19}F NMR spectrum after conversion of $[Ph_3P–PPh_3][Al^F]_2$ with $[Na(222crypt)]BF_4$ (*o*DFB with DMSO-d_6 capillary, 282.4 MHz). Unidentified minor signals are assigned to BF_3-species or solvent-impurities.

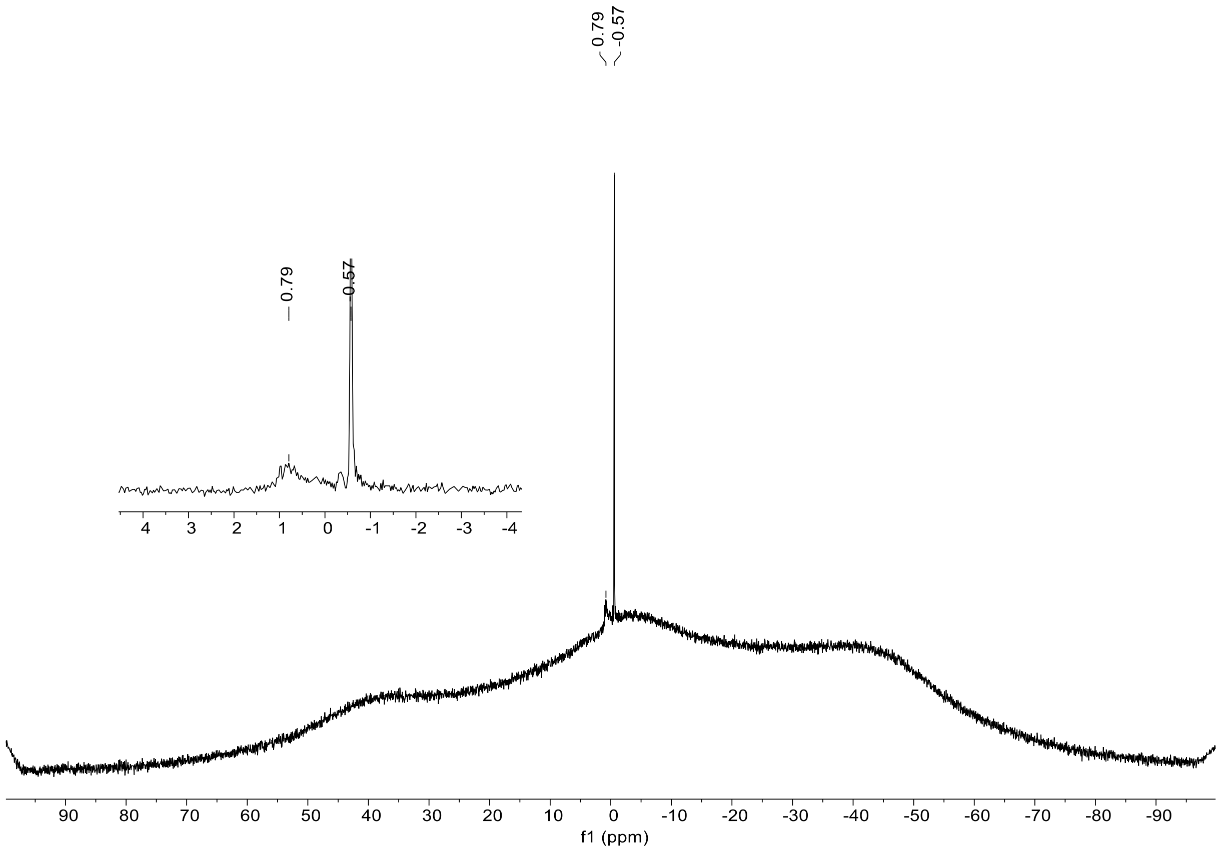

Figure S53. $^{11}B\{^{1}H\}$ NMR spectrum after conversion of $[Ph_3P–PPh_3][Al^F]_2$ with $[Na(222crypt)]BF_4$ (*o*DFB with DMSO-d_6 capillary, 96.2 MHz).

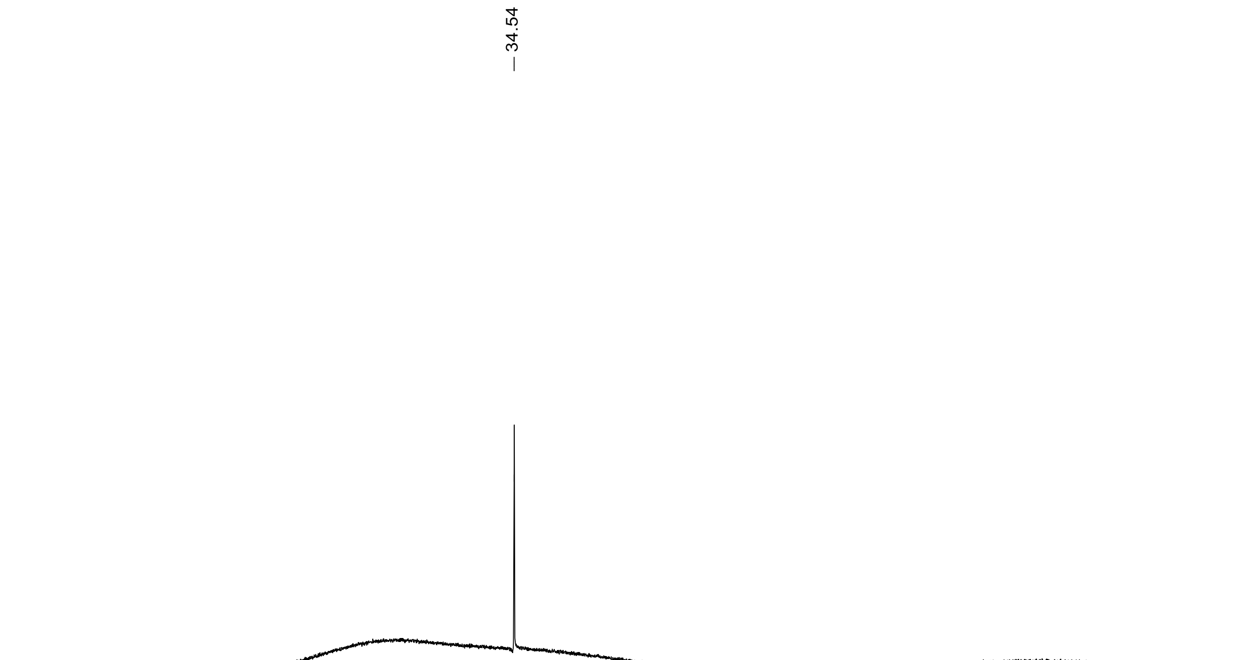

Figure S54. ^{27}Al NMR spectrum (*o*DFB with DMSO-d_6 capillary, 78.2 MHz).

2.12 Reaction of 1 with [Na(222crypt)][PF_6]: [$FPPh_3$][Al^F] and F_5PPPh_3

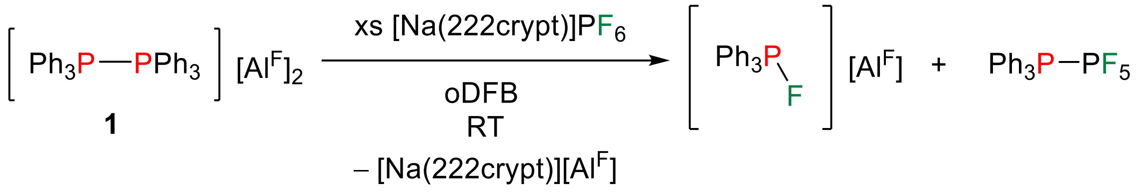

An excess of freshly prepared [Na(222crypt)]PF_6 (1.5 equiv., 0.0036) in *o*DFB was added to a *J. Young* NMR tube containing 6 mg of [Ph_3P–PPh_3][Al^F]$_2$ (**1**, 1.0 equiv., 0.0024 mmol), resulting in a pale-yellow colored solution. Conversion of [Ph_3P–PPh_3][Al^F]$_2$ is quantitative. The formation of [PPh_3F]$^+$ was detected as a characteristic doublet at 98.8 ppm in the ^{31}P NMR and –129.90 ppm in the ^{19}F NMR spectra with $^1J_{PF}$ = 996 Hz, upon running the reaction with [$BAr^F{}_{20}$]$^-$ instead of [Al^F] as anion.[14]

^{19}F NMR (*o*DFB with DMSO-d_6 capillary, 282.4 MHz): δ = –55.7 (ddd, –P$F_{equatorial}$ of Ph_3PPF_5, $^1J_{PF}$ = 760.8 Hz, $^2J_{FF}$ = 58.4 Hz, $^2J_{PF}$ = 7.0 Hz), –72.2 (d, P–*F* of xsNaPF_6, $^1J_{PF}$ = 710 Hz), –79.9 (dquint, –PF_{axial} of Ph_3PPF_5 $^1J_{PF}$ = 750.4 Hz, $^2J_{FF}$ = 58.5 Hz), –130.05 (d, $^1J_{PF}$ = 995 Hz, $^+PPh_3F$), –133.11 (br s, –C*F* of $BAr^F{}_{20}$), –164.43 (t, $^2J_{FF}$ = 20.2 Hz, –C*F* of $BAr^F{}_{20}$), –168.19 (t, $^2J_{FF}$ = 19.5 Hz, –C*F* of $BAr^F{}_{20}$) ppm.

^{31}P{^{1}H} NMR (*o*DFB with DMSO-d_6 capillary, 121.5 MHz): δ = 98.8 (d, $^1J_{PF}$ = 996 Hz, $^+PPh_3F$), 44.0 (ps dquint, *P*Ph_3PF_5, $^1J_{PP}$ = 24.0 Hz, $^2J_{PF}$ = 7.1 Hz), –143.8 (hept, $^1J_{PF}$ = 709.9 Hz, *P*–F of xsNaPF_6), –143.9 (ps qm, PPh_3*P*F_5) ppm.

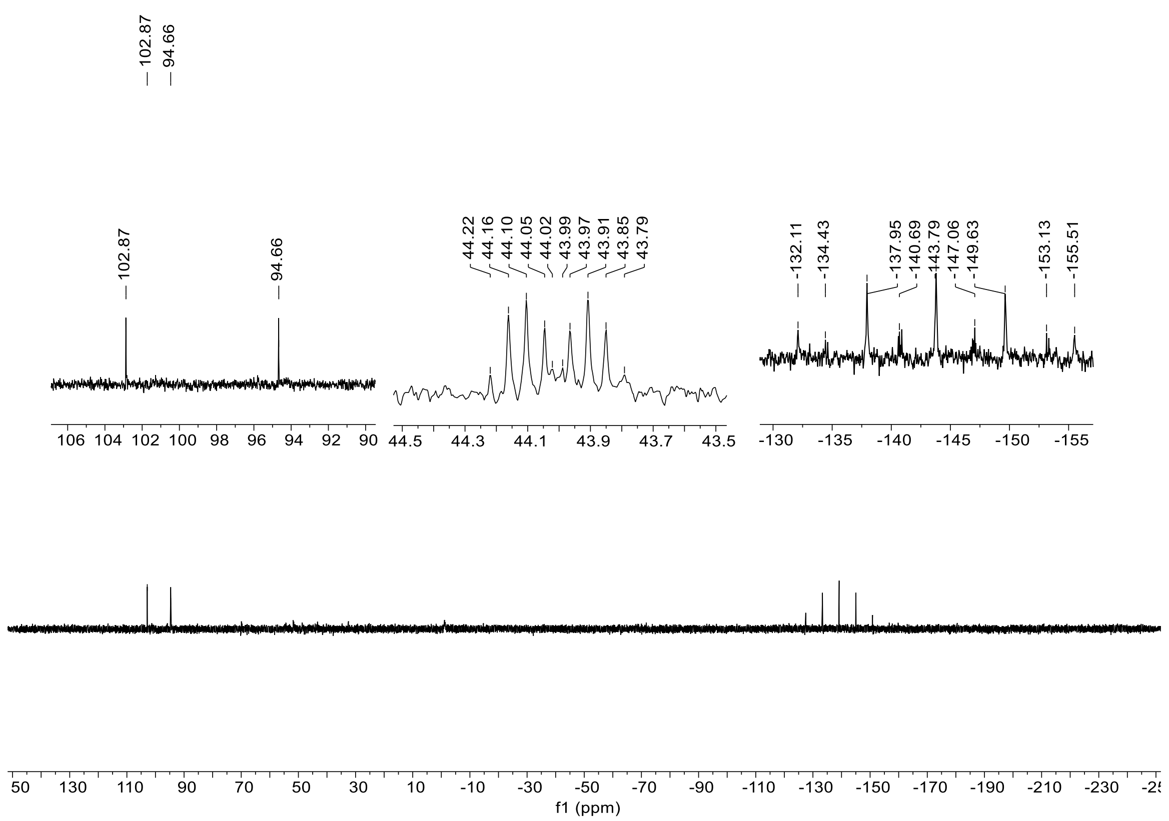

Figure S55. $^{31}P\{^1H\}$ NMR spectrum of the reaction conducted in the presence of $[BAr^F_{20}]$ anions for enhanced solubility of **1** (*o*DFB with DMSO-d_6 capillary, 121.5 MHz).

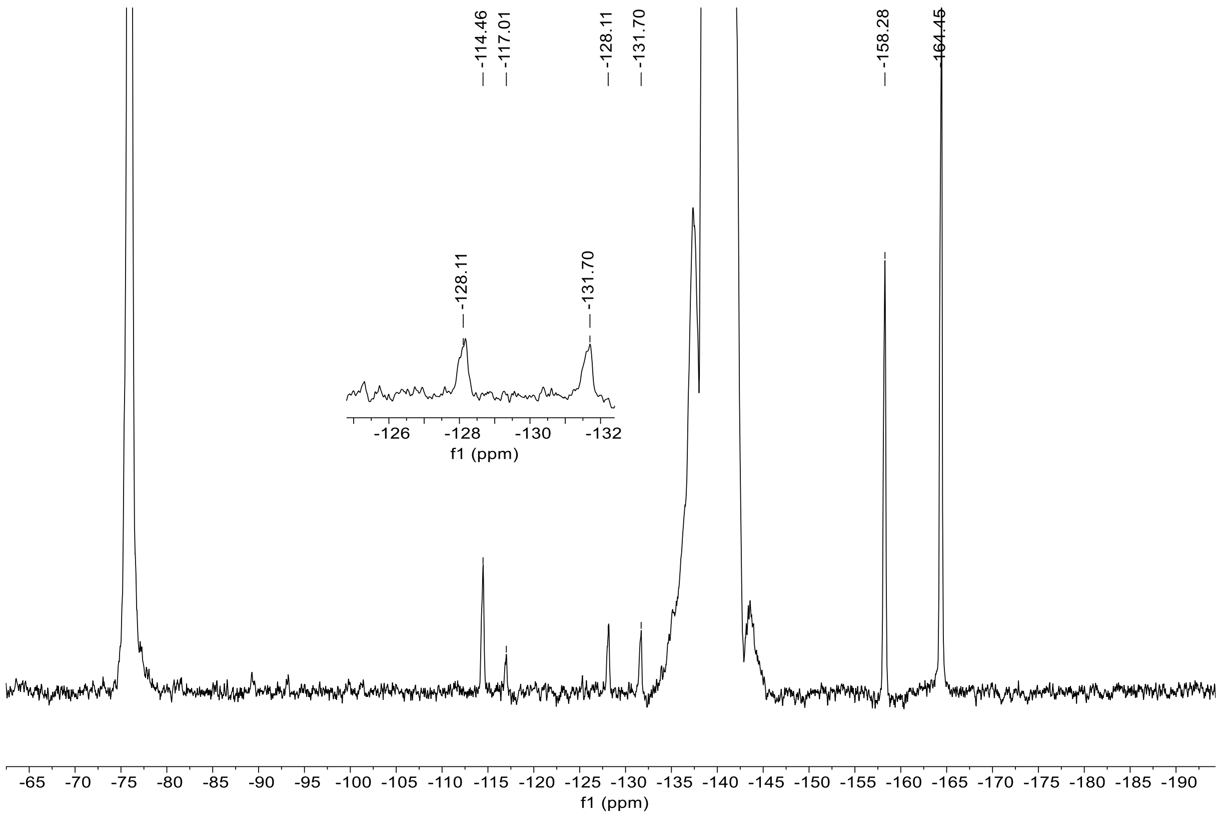

Figure S56. $^{19}F\{^1H\}$ NMR spectrum of the reaction in the presence of $[BAr^F_{20}]$ anions (*o*DFB with DMSO-d_6 capillary, 282.4 MHz).

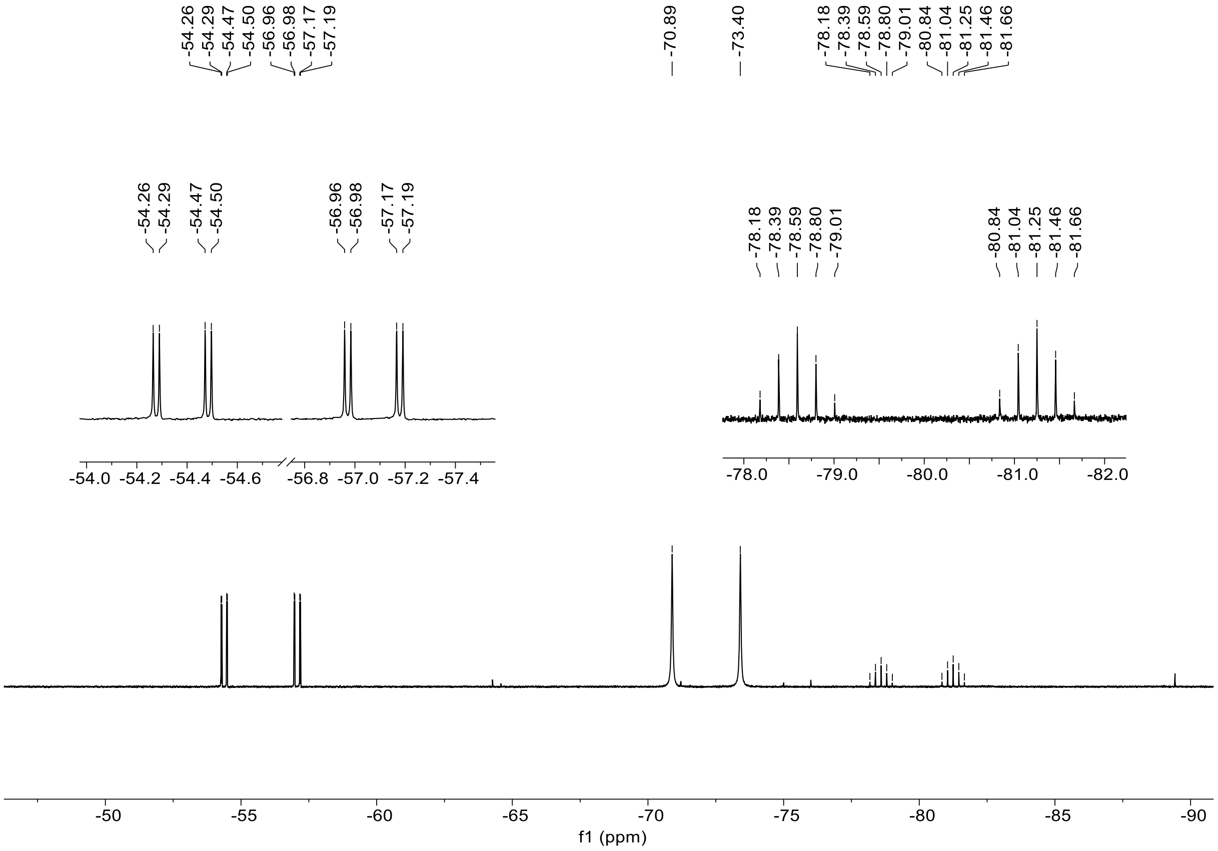

Figure S57. ^{19}F NMR spectrum visualizing coupling in [F_5P–PPh_3] of the reaction in the presence of [$BAr^F{}_{20}$] anions (*o*DFB with DMSO-d_6 capillary, 282.4 MHz).

2.13 Reaction of 1 with [PNP][SbF_6]: [$FPPh_3$][Al^F] and Ph_3PSbF_5

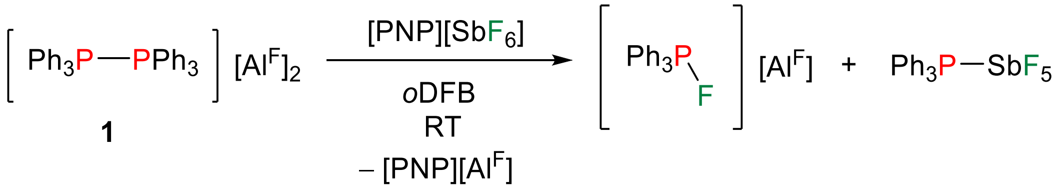

Inside the glovebox, [PNP][SbF_6] (15 mg, 0.018 mmol, 2 equiv.) was added to a suspension of 20 mg of [Ph_3P–PPh_3][Al^F]$_2$ (**1**, 1.0 equiv., 0.008 mmol) in 0.4 mL of *o*DFB in a *J. Young* NMR tube, upon which a minor amount of off-white precipitate formed. The supernatant of the reaction mixture was analyzed by NMR spectroscopies and revealed the stoichiometric formation of [Ph_3PF]$^+$ and tentative [PPh_3SbF_5] (whereof only the NHC derivative has been described in the literature; ^{19}F NMR spectrum: δ = −101.7 and −118.2 ppm)[15] based on the comparison with [$(PEt_3)_2SbF$][OTf]$_2$ (^{31}P NMR spectrum: singlet, δ = +38.0 ppm).[11] The precipitate seems to be insoluble in *o*DFB/TFB and hence its nature remains unknown.

^{31}P{^{1}H} NMR (*o*DFB with DMSO-d_6 capillary, 121.5 MHz): δ = 94.1 (d, $^1J_{PF}$ = 995 Hz, $^+PPh_3F$), 65.2 (s, [PPh_3–SbF_5]$^+$), 20.5 (s, [PN*P*]$^+$) ppm.

^{19}F NMR (*o*DFB with DMSO-d_6 capillary, 282.4 MHz): −77.3 (s, Al^{F-}), −107.1 (br, $SbF_{equatorial}$), −127.4 (br, SbF_{axial}), −130.1 (d, $^1J_{PF}$ = 996 Hz, $^+PPh_3F$) ppm.

^{27}Al NMR (*o*DFB with DMSO-d_6 capillary, 104.2 MHz): δ = 34.4 (s, *Al*{$OC(CF_3)_3$}$_4$) ppm.

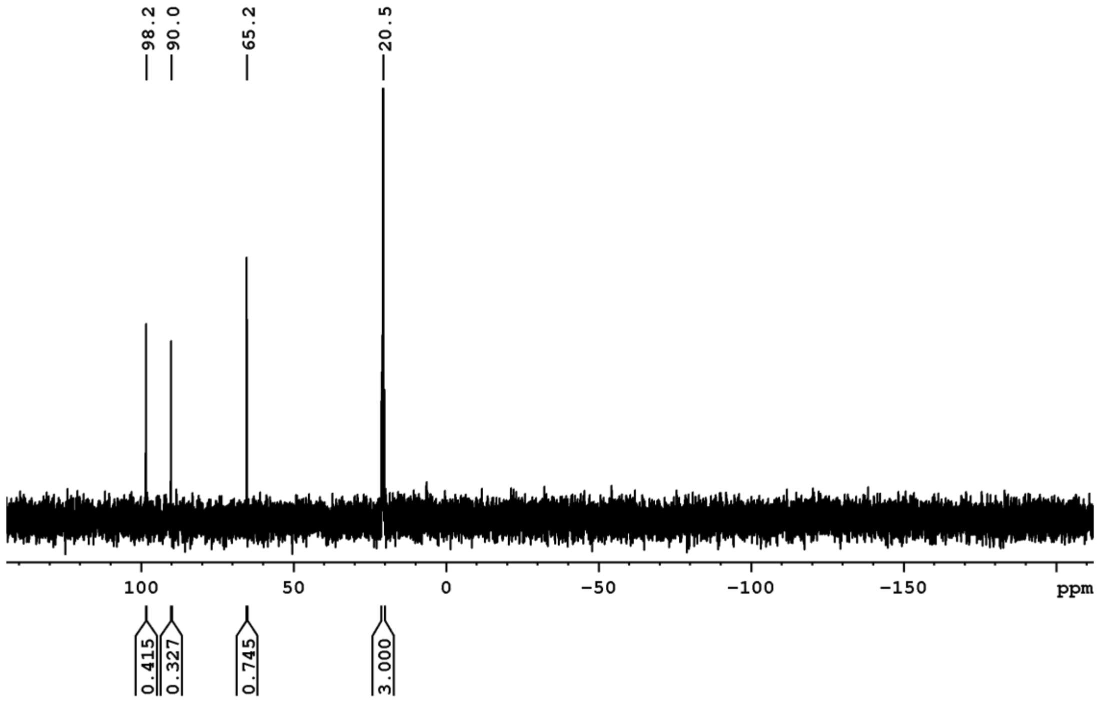

Figure S58. Crude $^{31}P\{^{1}H\}$ NMR spectrum obtained upon addition of [PNP][SbF_6] to [Ph_3P–PPh_3][Al^F]$_2$ (*o*DFB with DMSO-d_6 capillary, 121.5 MHz, D1 = 30s).

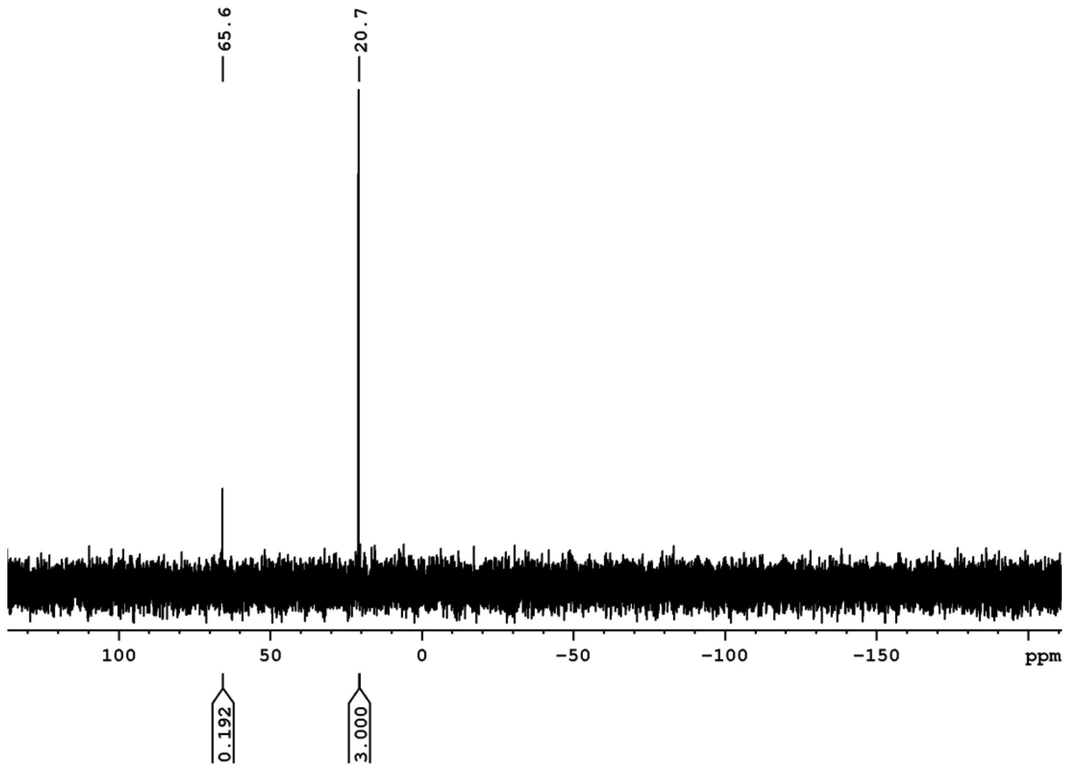

Figure S59. $^{31}P\{^{1}H\}$ NMR spectrum of the residue (TFB with DMSO-d_6 capillary, 121.5 MHz, D1 = 30 s).

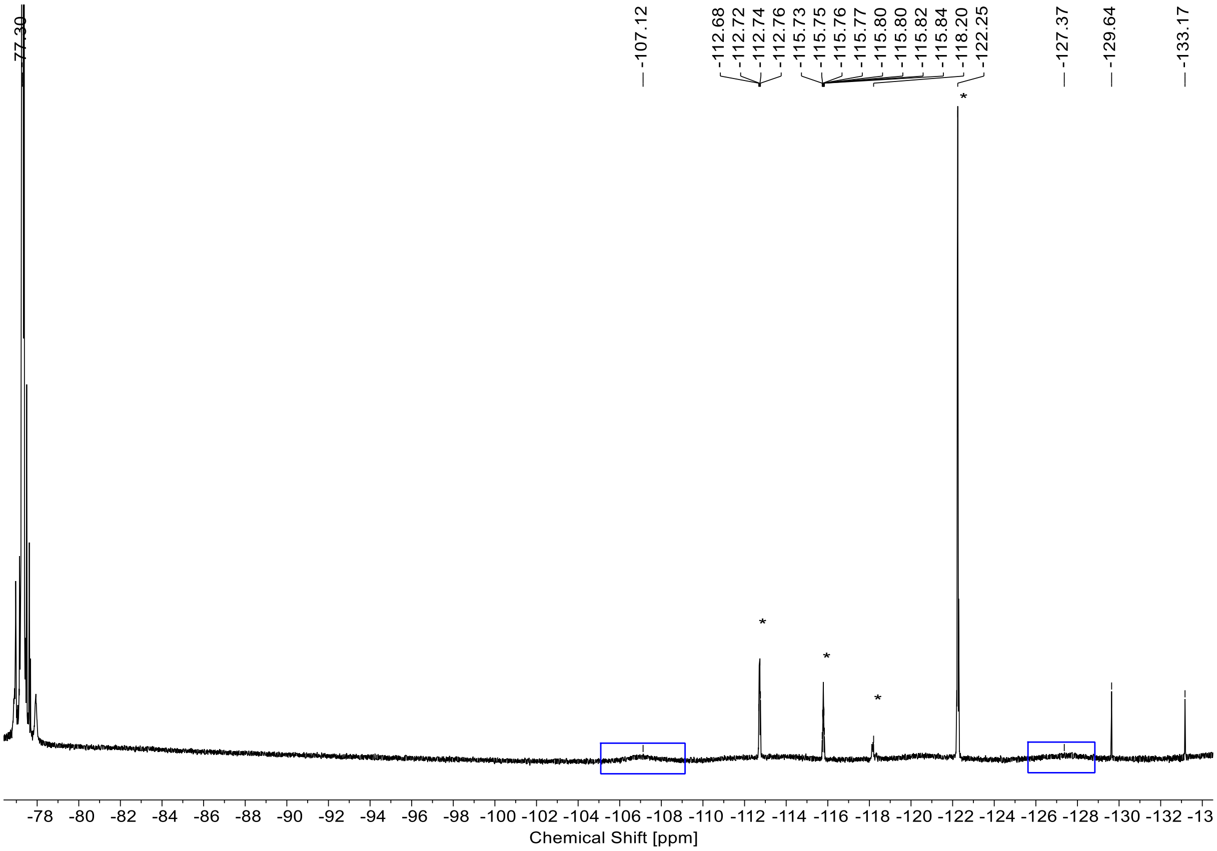

Figure S60. Crude ^{19}F NMR spectrum (*o*DFB with DMSO-d_6 capillary, 282.4 MHz). The blue rectangles highlight two broadened signals, which we attribute to the axial and equatorial set of fluorine atoms within the SbF_5 fragment. * = impurities present in *o*DFB.

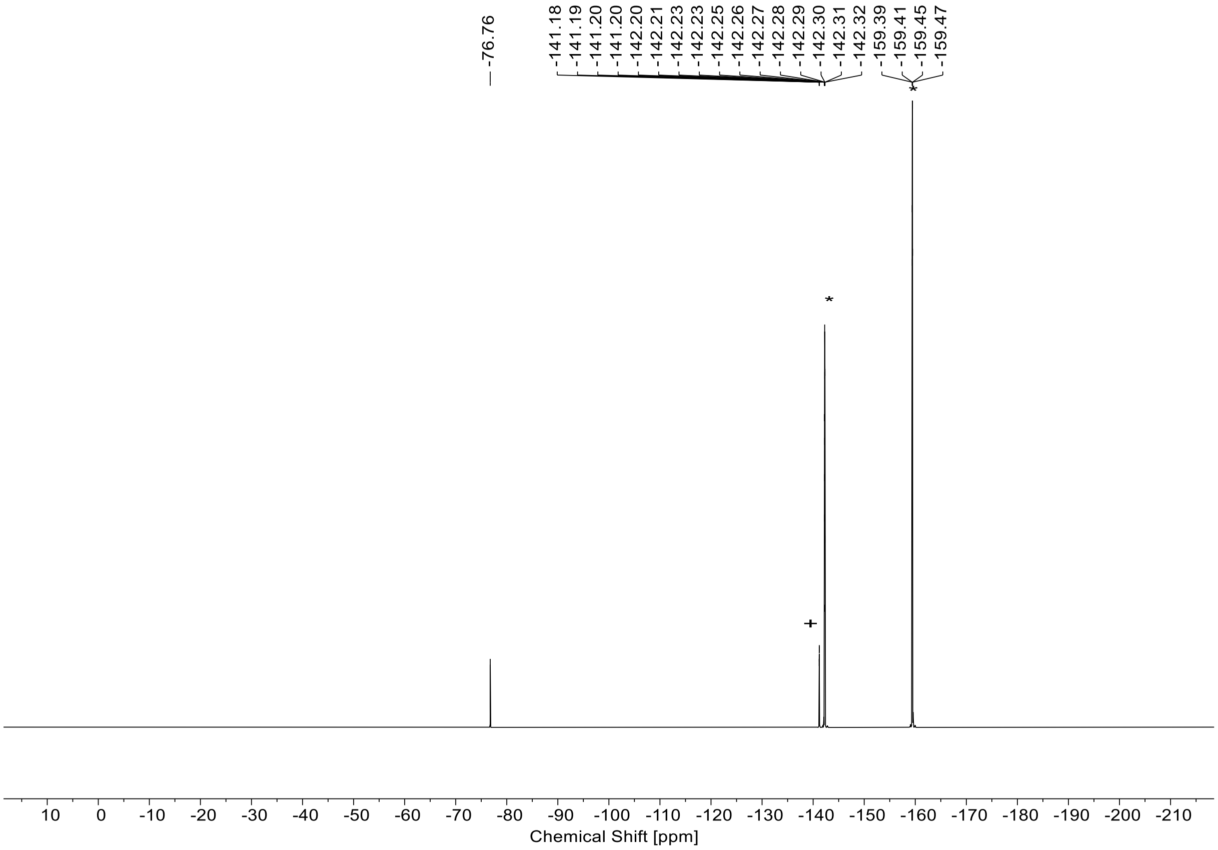

Figure S61. ^{19}F NMR spectrum of the residue (TFB with DMSO-d_6 capillary, 282.4 MHz, + = *o*DFB, * = TFB).

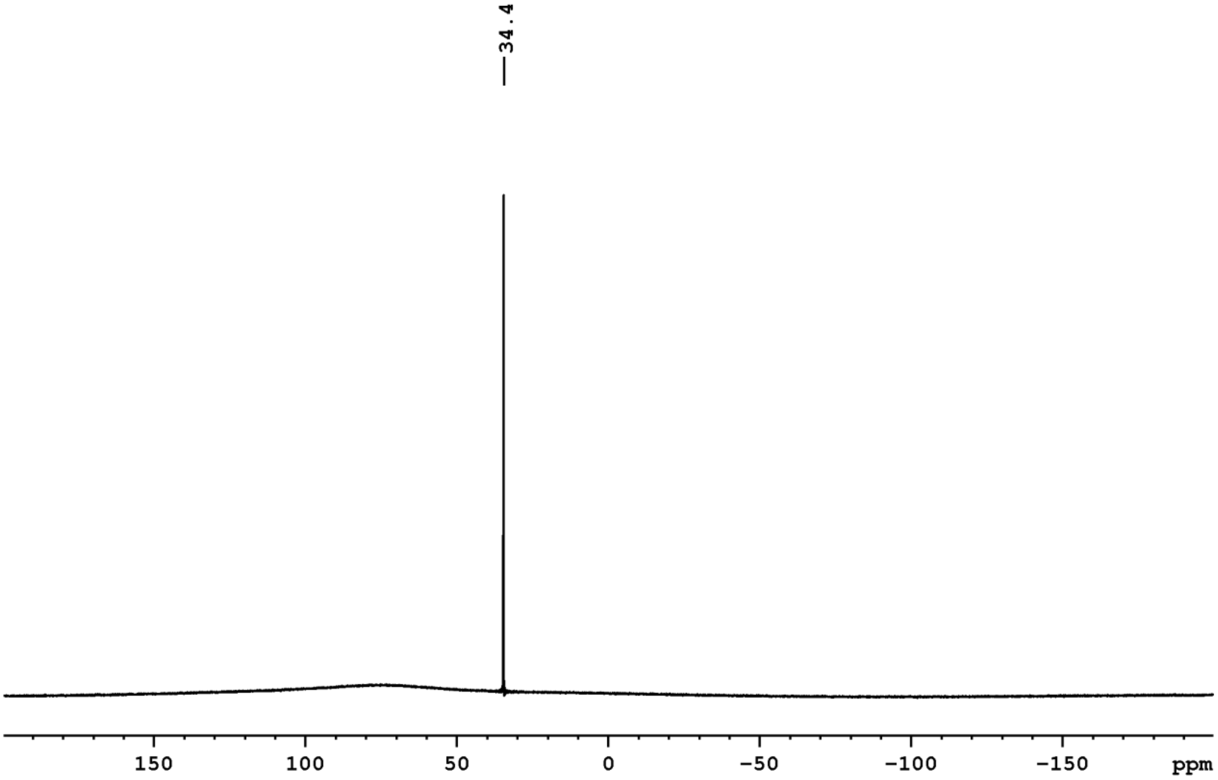

Figure S62. ^{27}Al NMR spectrum obtained upon addition of [PNP][SbF_6] to [Ph_3P–PPh_3][Al^F]$_2$ (*o*DFB with DMSO-d_6 capillary, 78.2 MHz).

2.14 Reaction of 1 with PhSSPh: $[Ph_3PSPh][Al^F]$

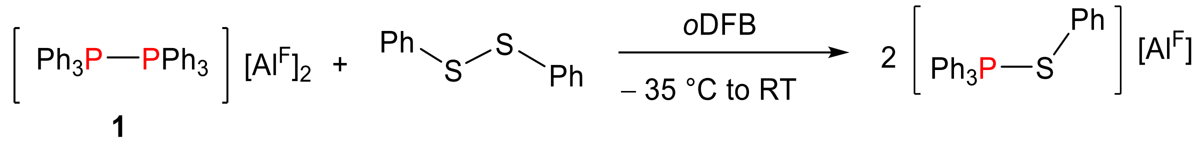

Inside the glovebox, freshly dried (under high vacuum for 1 day) 5 mg of Ph_2S_2 (2 eq, 0.0244 mmol) were added at low temperature (−35 °C) to a *J. Young* NMR tube containing 30 mg of $[Ph_3P–PPh_3][Al^F]_2$ (**1**, 1.0 eq, 0.0122 mmol) dissolved in *o*DFB. The solution changed from colorless to a pale-yellow. Conversion of $[P_2Ph_6][Al^F]_2$ to $[PPh_3SPh][Al^F]$ is quantitative according to the ^{31}P NMR spectroscopic analysis. For comparison with literature NMR spectra,[16] the reaction mixture was evaporated *in vacuo* and washed with *n*-pentane affording the product as a colorless solid (31 mg, 95%).

^{1}H NMR ($CDCl_3$, 300.1 MHz): δ = 7.66–7.61 (m, 3H, CH_{Ar}), 7.46-7.39 (m, 6H, CH_{Ar}), 7.30–7.23 (m, 6H, CH_{Ar}), 7.08-7.01 (m, 4H, CH_{Ar}), 6.91-6.89 (m, 1H, CH_{Ar}) ppm.

^{31}P{^{1}H} NMR ($CDCl_3$, 121.5 MHz): δ = 45.9 (s, Ph_3*P–SPh*$^+$) ppm.

^{19}F NMR ($CDCl_3$, 282.4 MHz): δ = –75.4 (s, –CF_3) ppm.

^{27}Al NMR ($CDCl_3$, 78.2 MHz): δ = 34.7(s, *Al*$\{OC(CF_3)_3\}_4$) ppm.

^{13}C{^{1}H} NMR ($CDCl_3$, 75.5 MHz): δ = 137.10 (d, $^{4}J_{CP}$ = 4.3 Hz), 136.61 (d, $^{5}J_{CP}$ = 3.6 Hz), 133.83 (d, $^{3}J_{CP}$ = 11 Hz), 132.78 (d, $^{4}J_{CP}$ = 3.8 Hz), 131.06 (d, $^{3}J_{CP}$ = 2.9 Hz), 130.82 (d, $^{2}J_{CP}$ = 13.2 Hz), 129.22, 127.67, 127.32, 119.47 (d, $^{2}J_{CP}$ = 7.0 Hz), 117.92 (d, $^{1}J_{CP}$ = 84.0 Hz) ppm.

m.p.: 109 °C

APPI-HRMS (in *o*DFB): *m/z* calcd for $C_{24}H_{20}PS^+$, 371.1023; found, 371.1014.

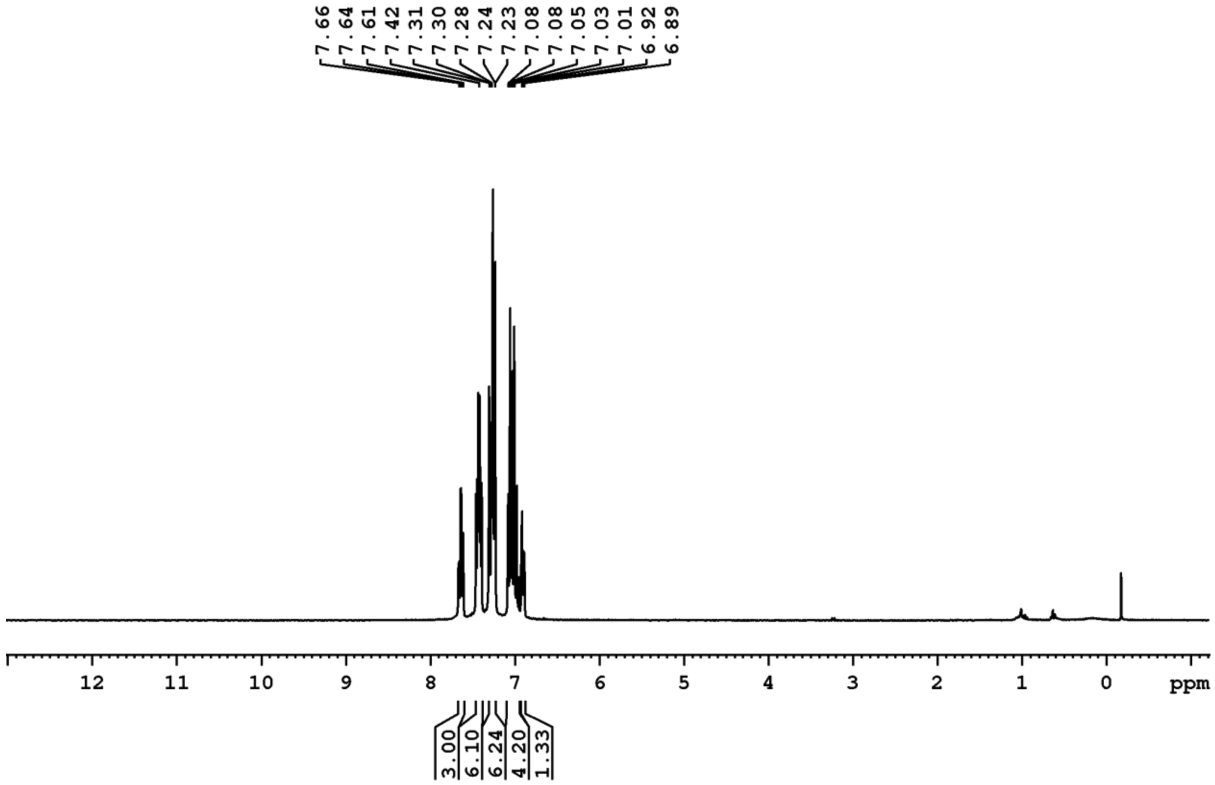

Figure S63. ¹H-NMR spectrum of [Ph_3P–SPh][Al^F] ($CDCl_3$, 300.1 MHz).

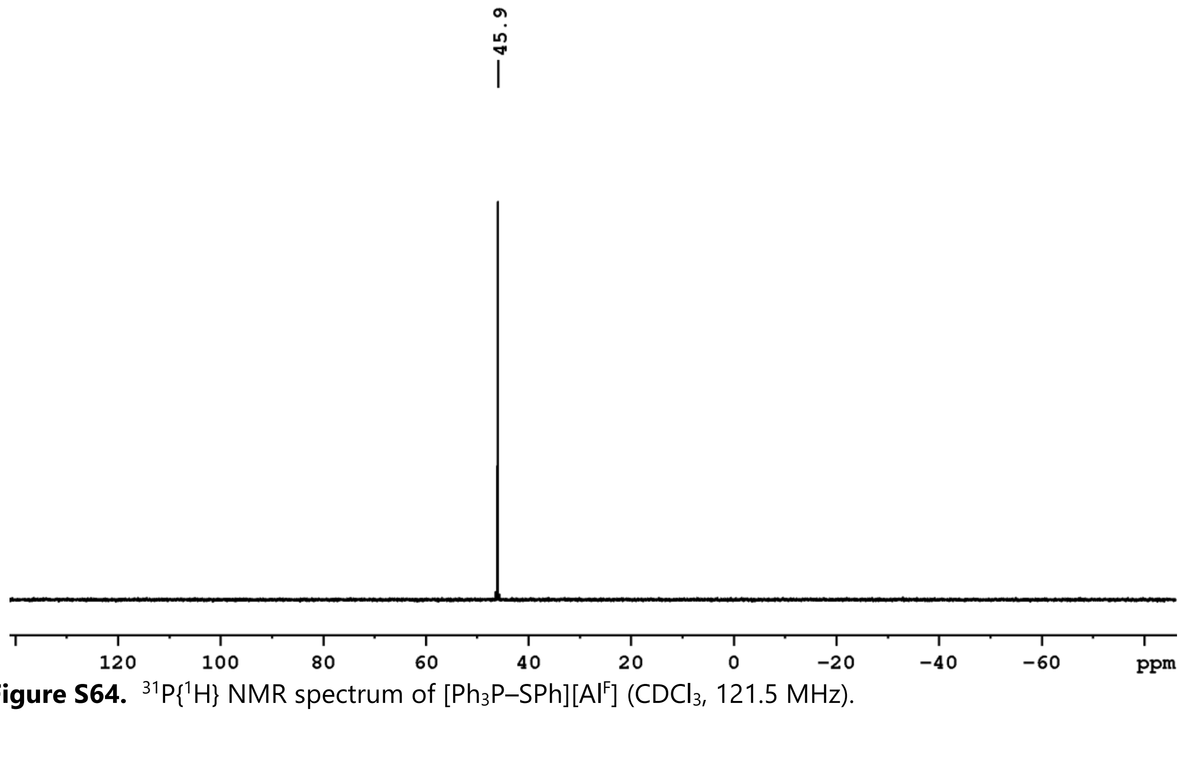

Figure S64. $^{31}P\{^1H\}$ NMR spectrum of [Ph_3P–SPh][Al^F] ($CDCl_3$, 121.5 MHz).

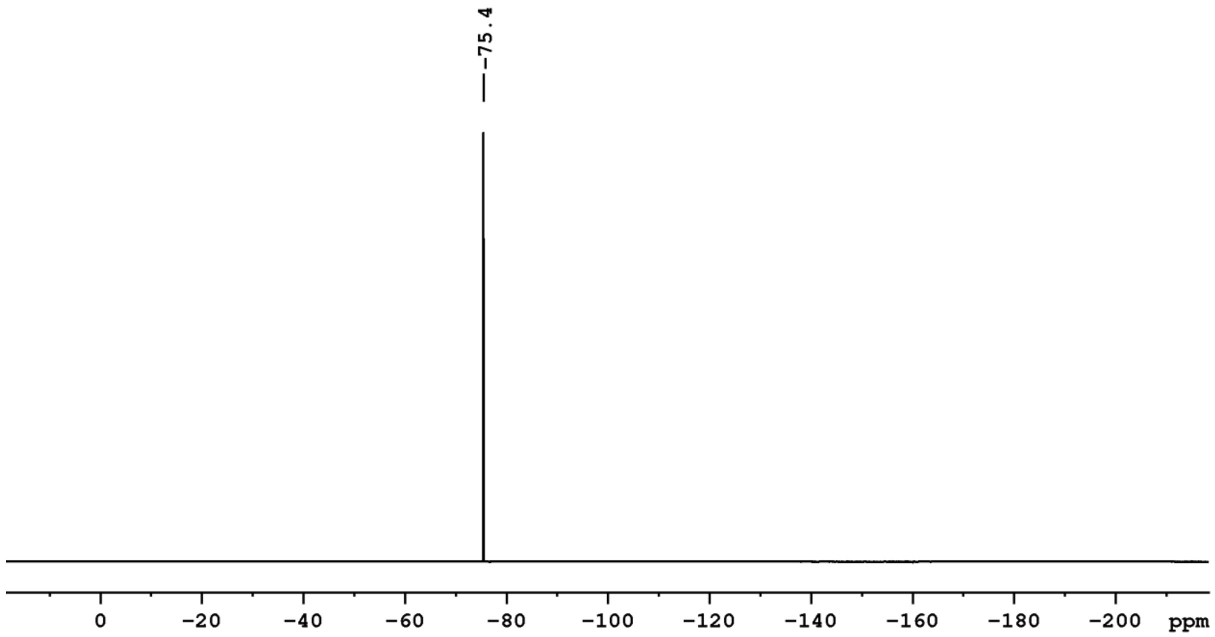

Figure S65. ^{19}F NMR spectrum of $[Ph_3P–SPh][Al^F]$ ($CDCl_3$, 282.4 MHz).

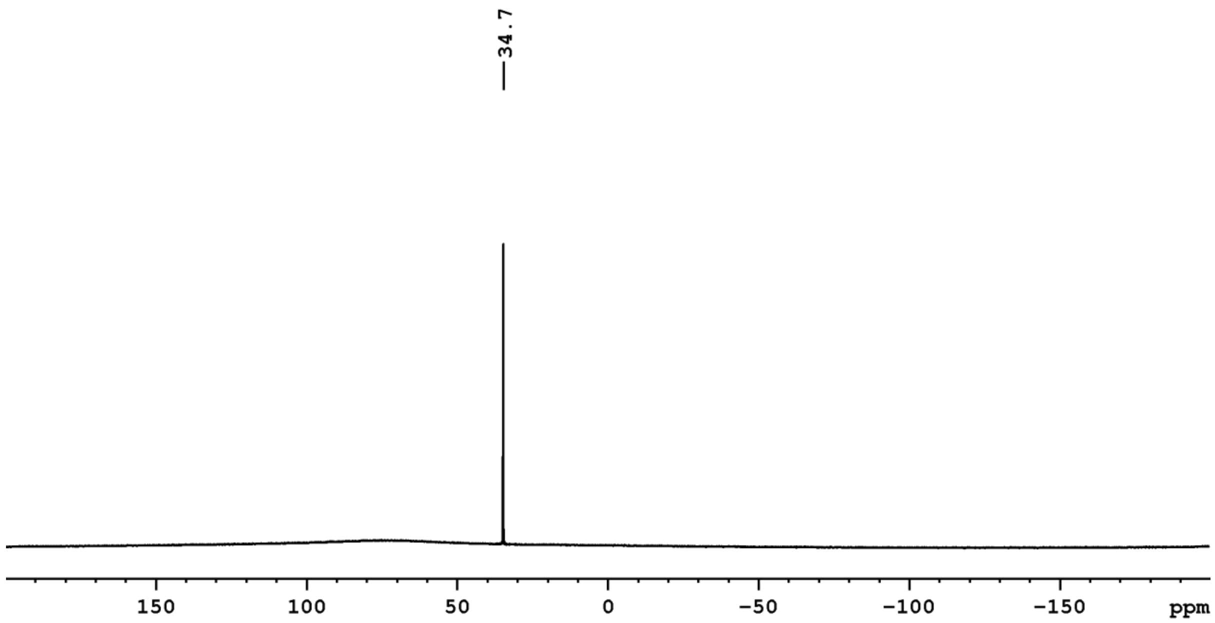

Figure S66. ^{27}Al NMR spectrum of $[Ph_3P–SPh][Al^F]$ ($CDCl_3$, 78.2 MHz).

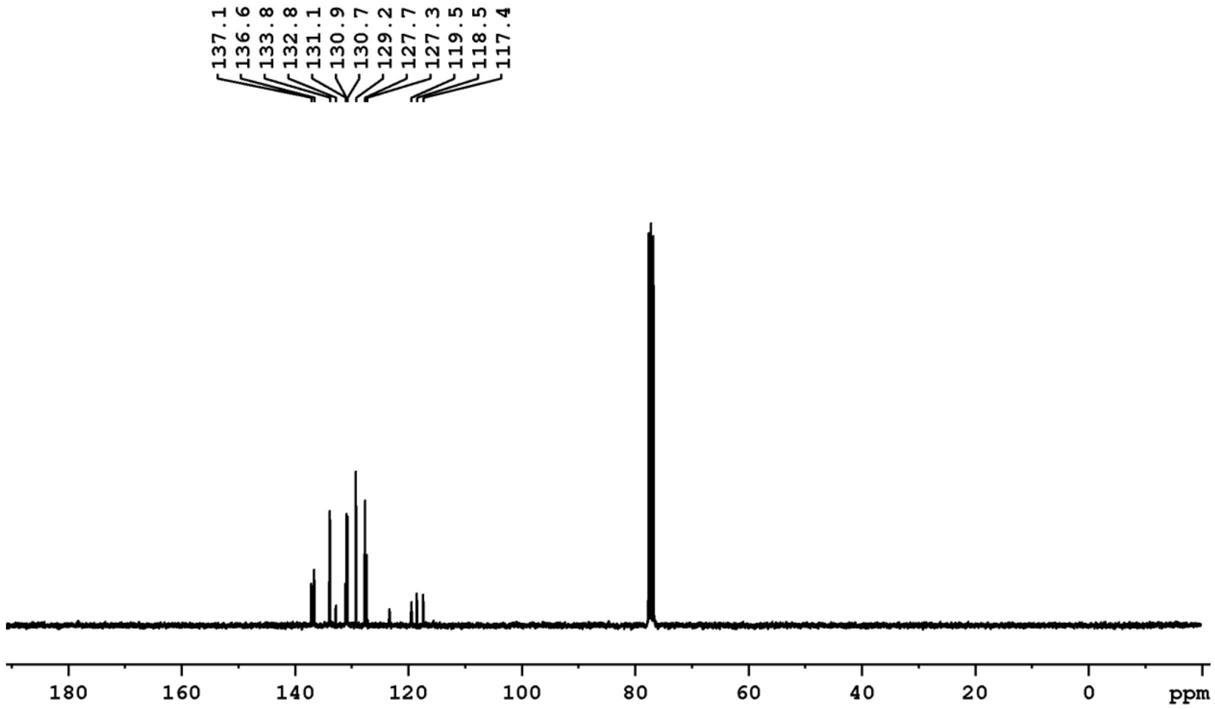

Figure S67. $^{13}C\{^{1}H\}$ NMR spectrum of $[Ph_3P–SPh][Al^F]$ ($CDCl_3$, 75.5 MHz).

2.15 Reaction of 1 with 1 equiv. Water: $[Ph_3PH][Al^F]$ and $[Ph_3P{-}O{-}PPh_3][Al^F]_2$

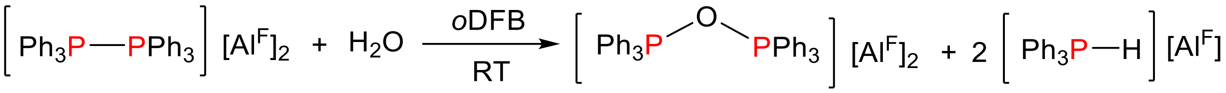

1

Solution: A saturated solution of **1** was prepared in slightly wet (*i.e.* about 1 equiv.) *o*DFB and analyzed by NMR spectroscopies.

Solid State: Solid **1** was exposed to moist air for 5 min. The subsequent NMR-spectroscopic analysis in dry *o*DFB revealed the quantitative and stoichiometric conversion to $[Ph_3PH][Al^F]$ and $[Ph_3POPPh_3][Al^F]_2$, just as was observed in the solution-phase experiment.

$^{31}P\{^1H\}$ NMR (*o*DFB with DMSO-d_6 capillary, 162.0 MHz): δ = 76.3 (s, $[Ph_3\mathit{P}O\mathit{P}Ph_3]^{2+}$, 7.7 [s, Ph_3PH]) ppm.

^{31}P NMR (*o*DFB with DMSO-d_6 capillary, 162.0 MHz): δ = 76.2 (s, $[Ph_3\mathit{P}O\mathit{P}Ph_3]^{2+}$, 7.7 [d, $^1J_{PH}$ = 496 Hz, $[Ph_3\mathit{P}H]^+$) ppm.

^{27}Al NMR (*o*DFB with DMSO-d_6 capillary, 104.2 MHz): δ = 34.4(s, $\mathit{Al}\{OC(CF_3)_3\}_4$) ppm.

ESI-MS (in *o*DFB):
m/z calcd for $C_{18}H_{16}P^+$, 263.0990; found, 263.0994.
m/z calcd for $C_{36}H_{30}OP_2^+$, 540.1766; found, 540.1760.

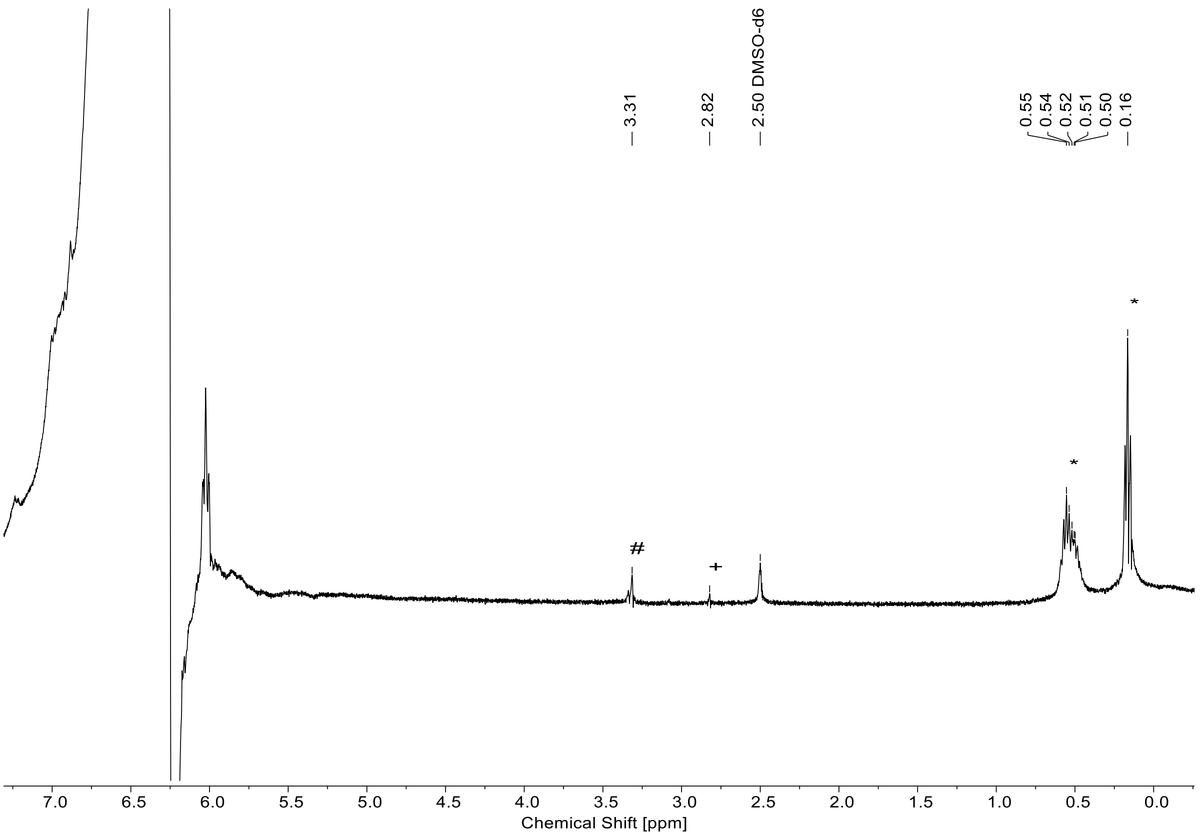

Figure S68. ^{1}H NMR spectrum of the careful hydrolysis of a saturated solution of $[Ph_3P–PPh_3][Al^F]_2$ (*o*DFB with DMSO-d_6 capillary, 400.1 MHz). The presence of minor amounts of H_2O (+) in *o*DFB is indicated by +; # = H_2O in DMSO-d_6 capillary, * = pentane.

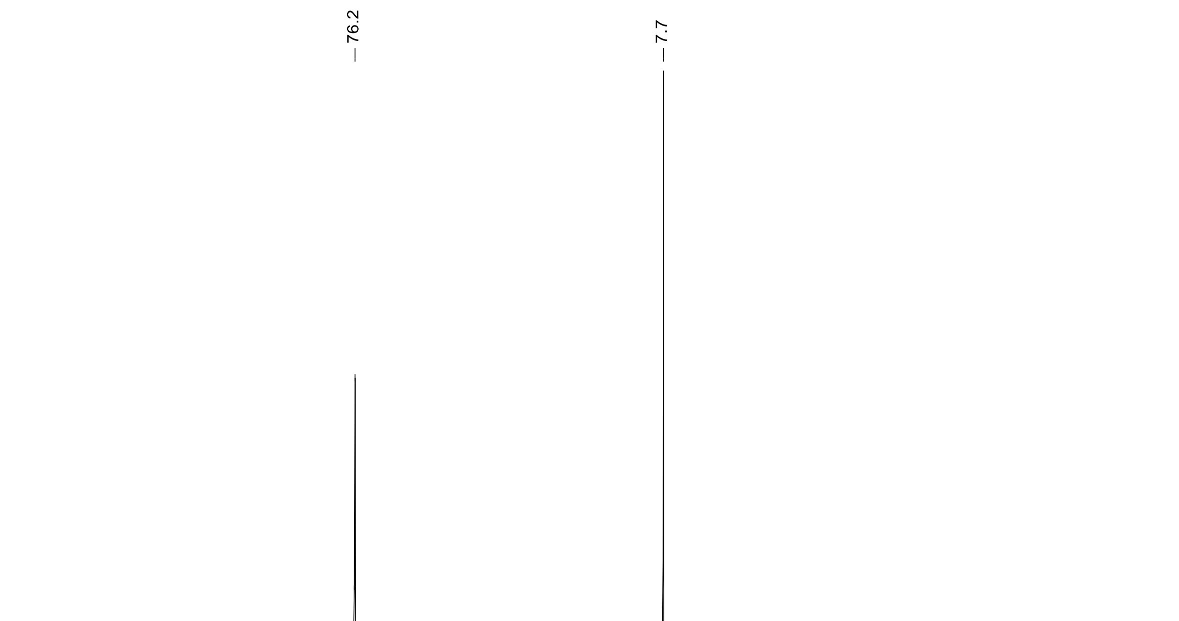

Figure S69. $^{31}P\{^{1}H\}$ NMR spectrum after careful hydrolysis of $[Ph_3P–PPh_3][Al^F]_2$ (*o*DFB with DMSO-d_6 capillary, 162.0 MHz, D1 = 30 s).

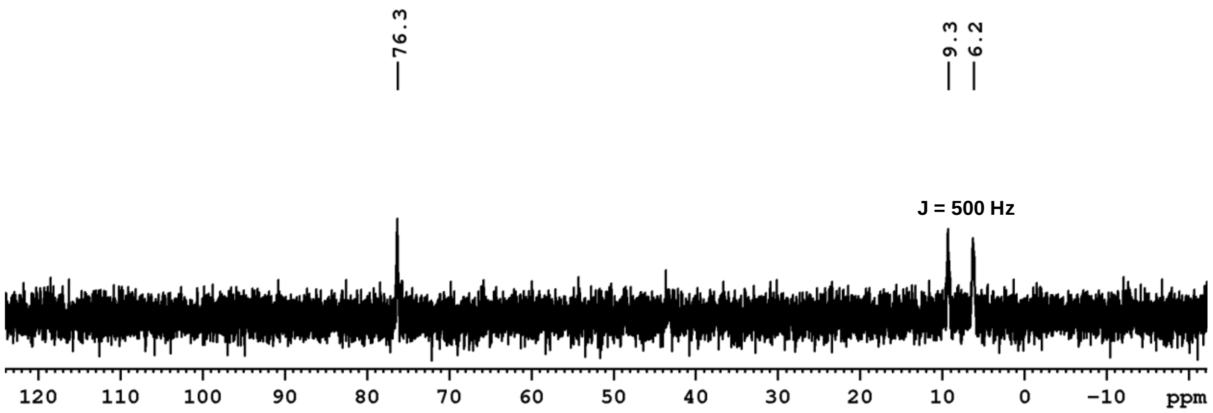

Figure S70. ^{31}P NMR spectrum after careful hydrolysis of $[Ph_3P–PPh_3][Al^F]_2$ (*o*DFB with DMSO-d_6 capillary, 162.0 MHz).

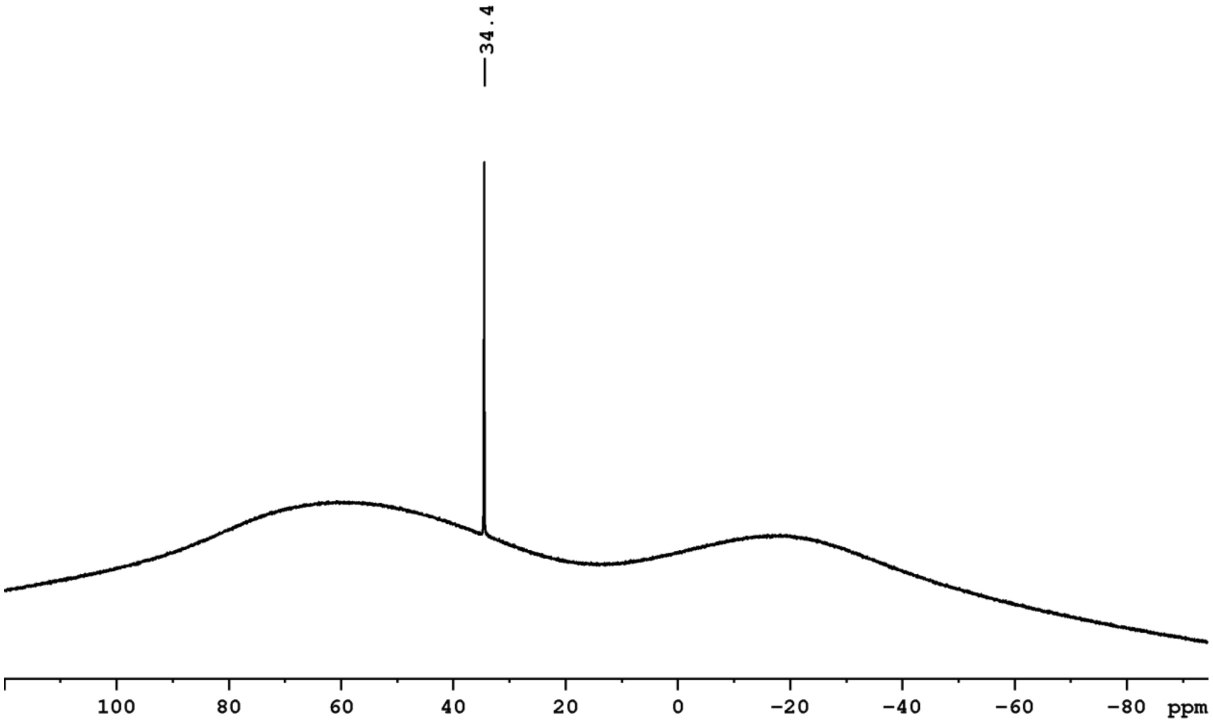

Figure S71. ^{27}Al NMR spectrum after exposing $[Ph_3P–PPh_3][Al^F]_2$ to moist air (*o*DFB with DMSO-d_6 capillary, 104.2 MHz).

2.16 Reaction of 1 with Benzonitrile (^{Ph}4)

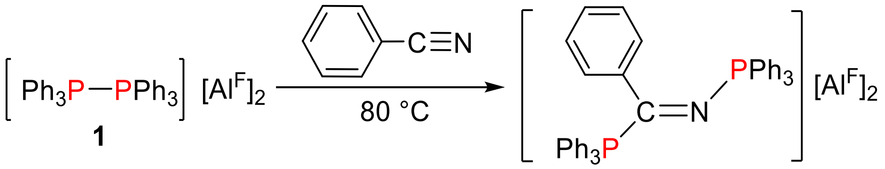

In a *J. Young* capped NMR tube, **1** (20 mg, 0.008 mmol) was dissolved in benzonitrile. The *in-situ* NMR spectroscopic analysis indicated a very slow reaction at room temperature, and only 30% conversion to the product upon heating to 40 °C for 3 h. Quantitative conversion to $^{\mathbf{Ph}}$**4** was obtained upon heating to 80 °C overnight. The solvent was removed *in vacuo*, and the crude product was purified by layering *n*-pentane onto a concentrated DCM solution of crude $^{\mathbf{Ph}}$**4** and storing the sample at −35 °C. Colorless crystals suitable for sc-XRD formed overnight, which were collected and then dried *in vacuo* (9 mg, 43%).

^{31}P{^{1}H} NMR (CD_3CN, 162.0 MHz): δ = 37.1 (d, $^{4}J_{PP}$ = 70 Hz, N*P*Ph_3), 24.9 (d, $^{4}J_{PP}$ = 70 Hz, C*P*Ph_3) ppm.

^{1}H NMR (CD_3CN, 400.1 MHz): δ = 8.79–8.77 (m, 1H, CH_{Ar}), 7.98–7.82 (m, 6H, CH_{Ar}), 7.74–7.60 (m, 14H, CH_{Ar}), 7.54–7.43 (m, 10H, CH_{Ar}), 7.36–7.32 (m, 2H, CH_{Ar}), 7.00-6.97 (m, 1H, CH_{Ar}), 6.78–6.76 (m, 1H, CH_{Ar}), ppm.

^{13}C{^{1}H} NMR (CD_3CN, 100.6 MHz): 137.7 (d, $^{4}J_{CP}$ = 3 Hz, CH_{Ar}), 137.4 (d, $^{4}J_{CP}$ = 3 Hz, CH_{Ar}), 136.5 (d, $^{3}J_{CP}$ = 11 Hz, CH_{Ar}), 135.2 (d, $^{3}J_{CP}$ = 11 Hz, CH_{Ar}), 134.9 (d, $^{3}J_{CP}$ = 11 Hz, CH_{Ar}),134.1 (d, $^{2}J_{CP}$ = 16 Hz, CH_{Ar}), 133.2, 131.5 (dd, $^{1}J_{CP}$ = 39 Hz, $^{3}J_{CP}$ = 13 Hz, $C_6H_5CPPh_3$), 130.3, 129.9, 128.4, 122.3 (q, $^{1}J_{CF}$ = 295 Hz, $-CF_3$), 118.9, 115.6 (d, $^{1}J_{CP}$ = 90 Hz, CH_{Ar}) ppm.

^{19}F NMR (*o*DFB with DMSO-d_6 capillary, 282.4 MHz): δ = −76.00 (s, –C*F*$_3$) ppm.

^{27}Al NMR (CD_3CN, 104.2 MHz) : δ = 34.6 ppm.

ESI-MS (in Acetonitrile):

m/z calcd. for $C_{43}H_{35}NP_2^{+}$: 313.6116; found: 313.6119.

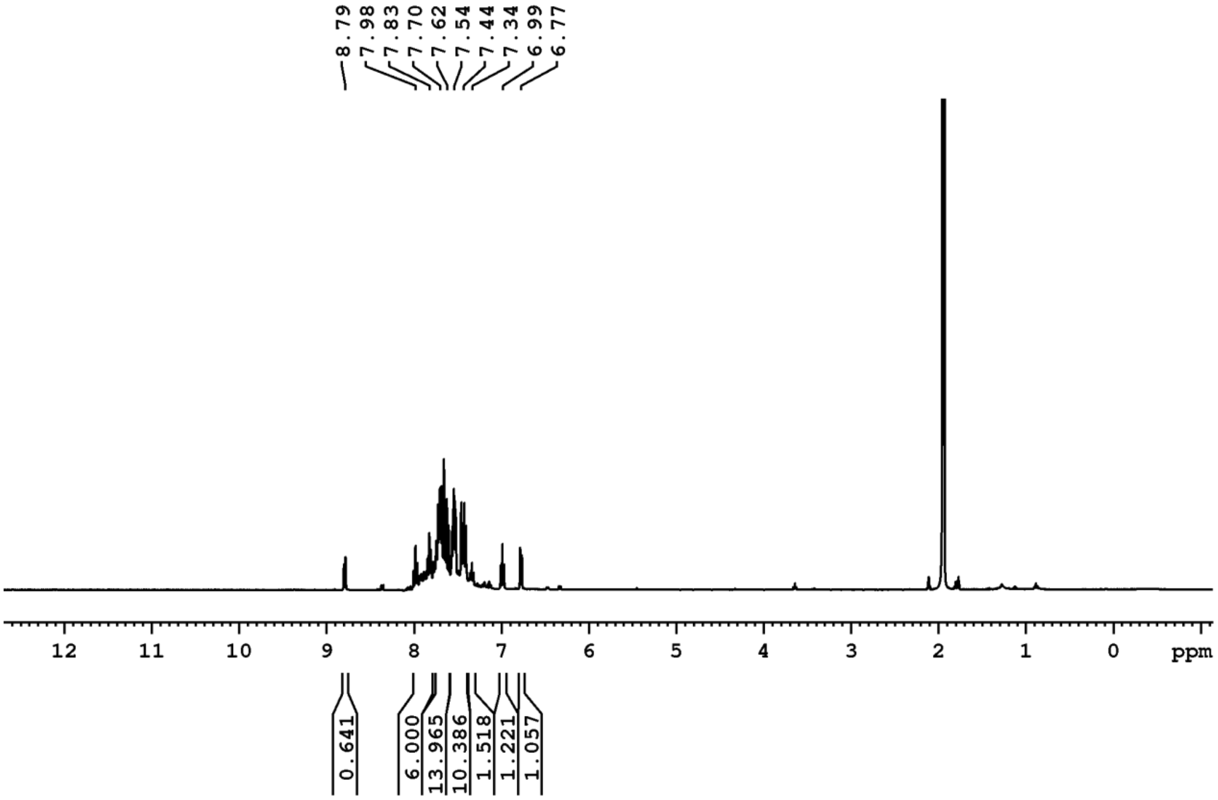

Figure S72. ^{1}H NMR spectrum of **^{Ph}4** (CD_3CN, 400.1 MHz).

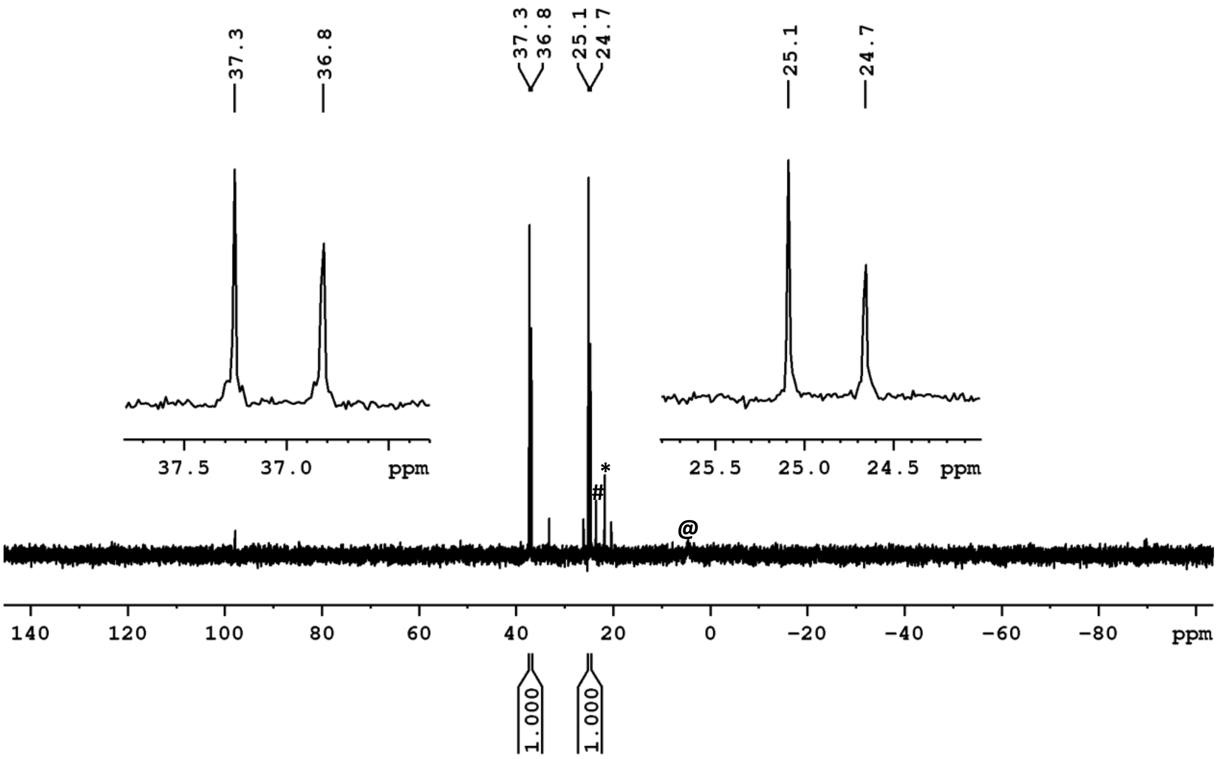

Figure S73. The $^{31}P\{^{1}H\}$ NMR spectrum of **^{Ph}4** (CD_3CN, 162.0 MHz; #Ph_3PO, *unknown, @Ph_3PH^+).

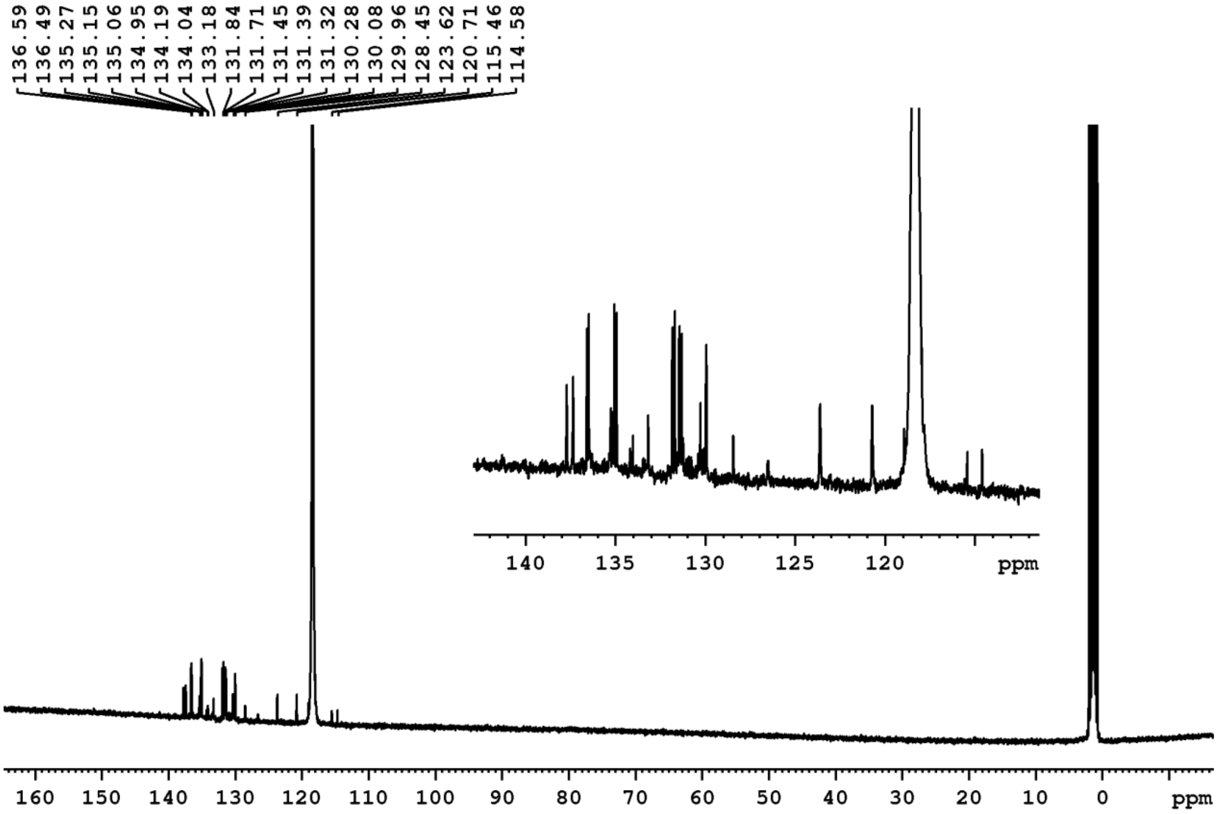

Figure S74. $^{13}C\{^{1}H\}$ NMR spectrum of **^{Ph}4** (CD_3CN, 100.6 MHz).

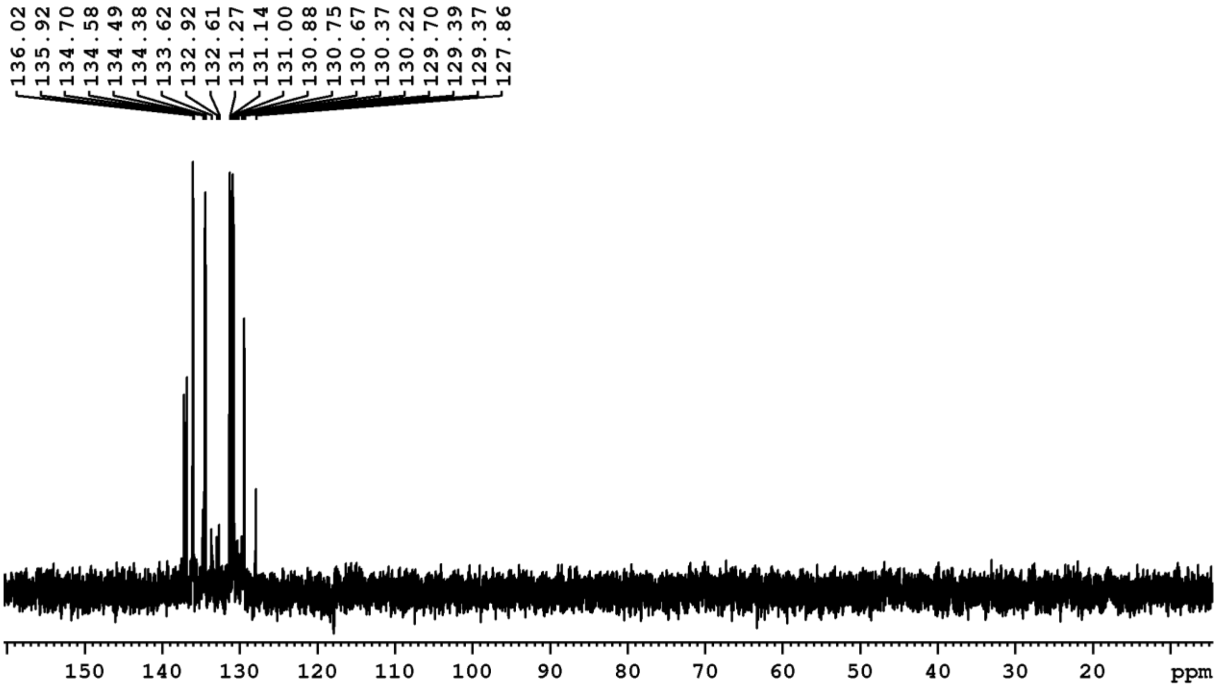

Figure S75. DEPT-135 NMR spectrum of **^{Ph}4** (CD_3CN, 100.6 MHz)

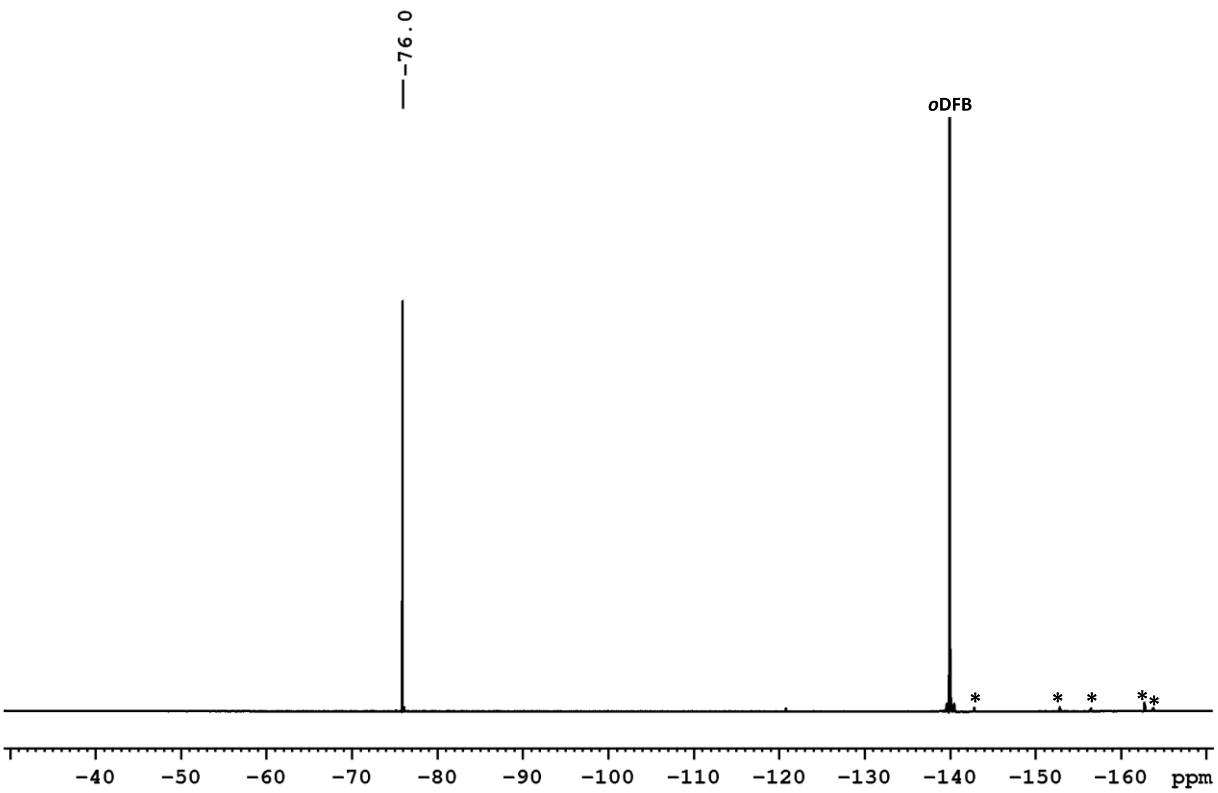

Figure S76. ^{19}F NMR spectrum of $^{\mathbf{Ph}}\mathbf{4}$ (*o*DFB with DMSO-d_6 capillary, 282.4 MHz, *trace Phenazine^F impurity).

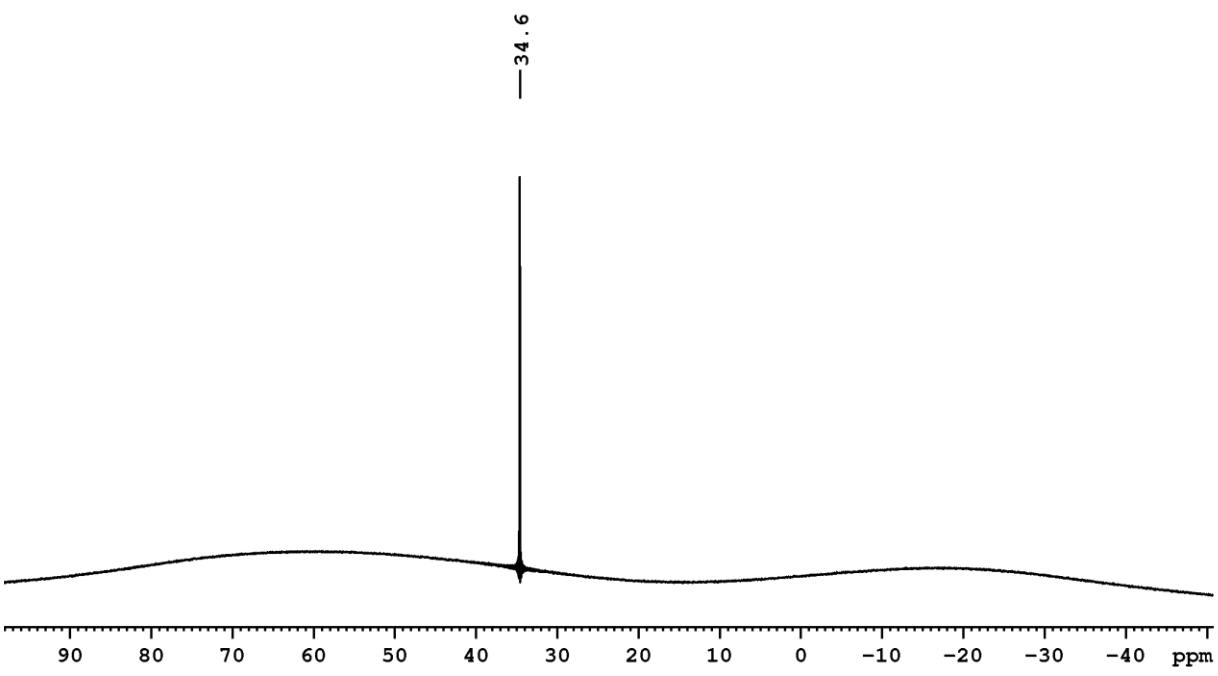

Figure S77. ^{27}Al NMR spectrum of $^{\mathbf{Ph}}\mathbf{4}$ (CD_3CN, 104.2 MHz).

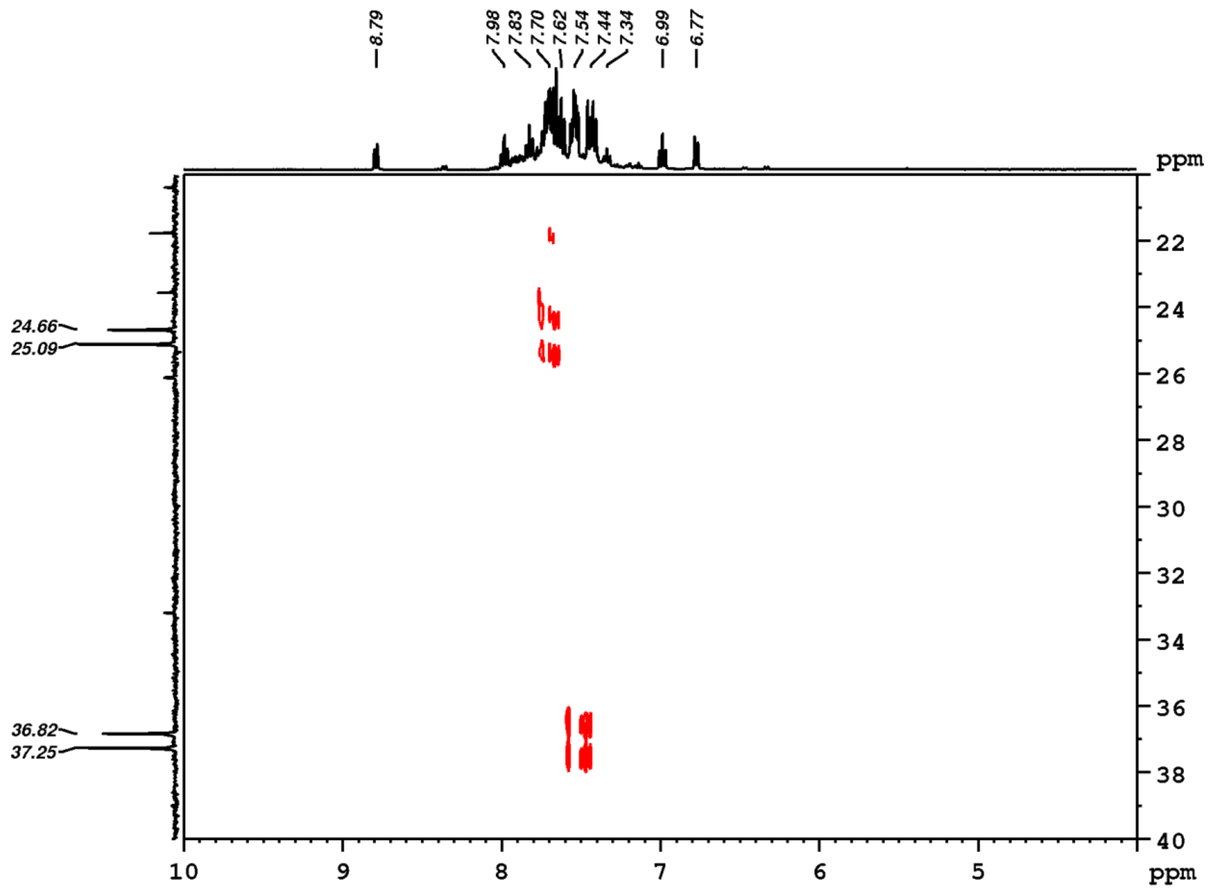

Figure S78. ^{1}H–^{31}P correlated HMBC NMR spectrum of **^{Ph}4** (CD_3CN, 400.1/162.0 MHz).

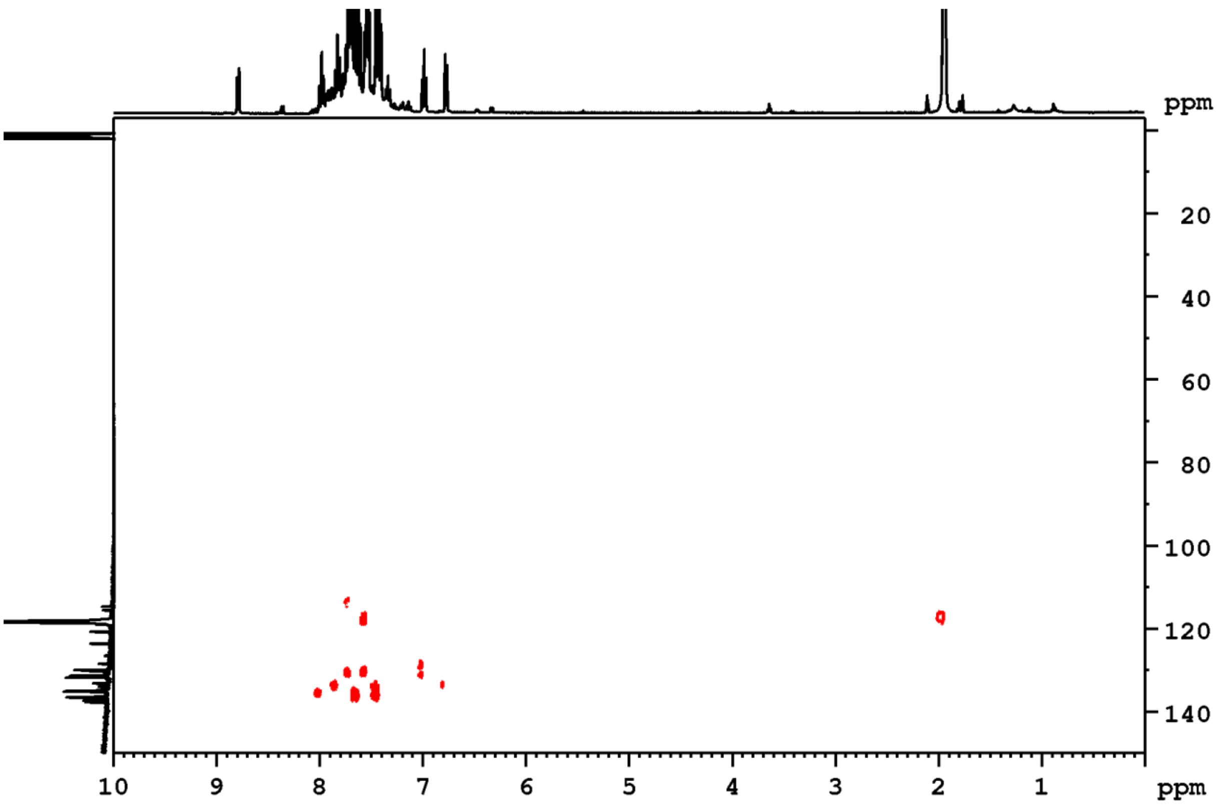

Figure S79. ^{1}H–^{13}C correlated HMBC NMR spectrum of **^{Ph}4** (CD_3CN, 400.1/100.6 MHz).

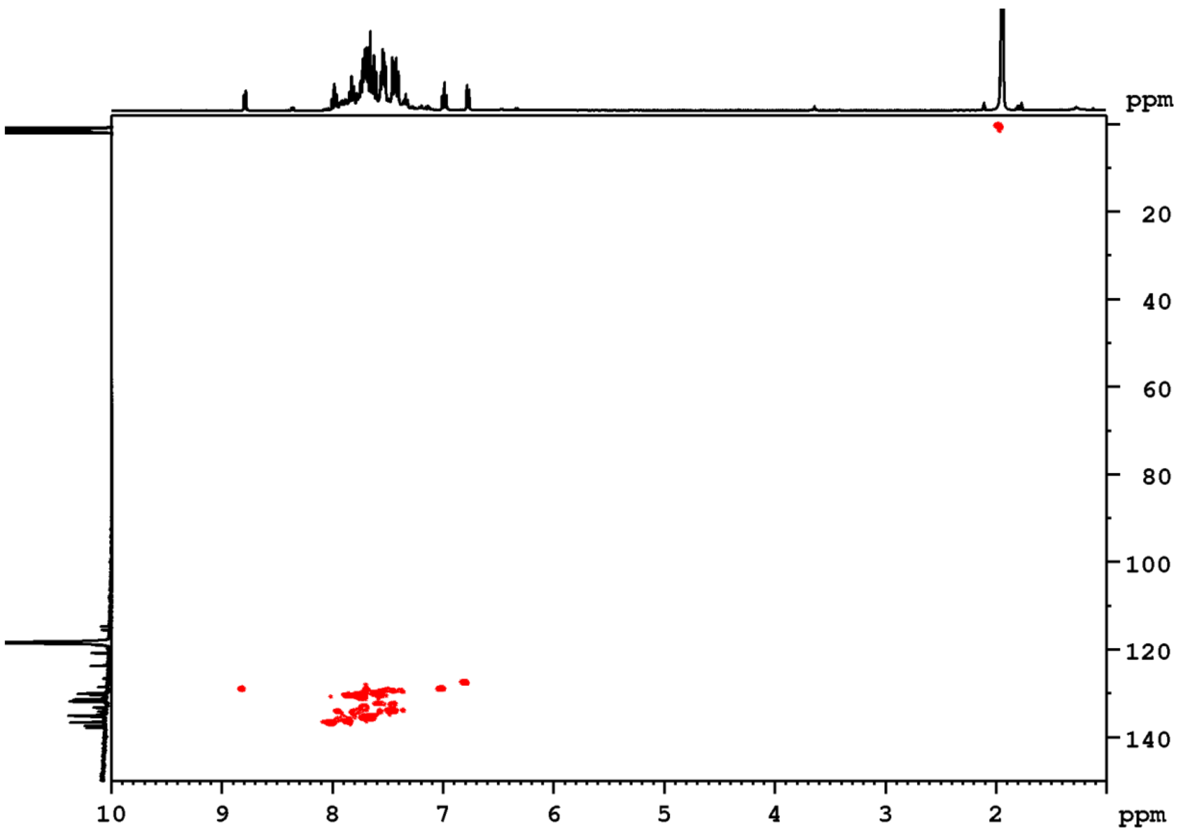

Figure S80. ^{1}H–^{13}C correlated HSQC NMR spectrum of **^{Ph}4** (CD_3CN, 400.1/100.6 MHz).

2.17 Reaction of 1 with Acetonitrile (^{Me}5)

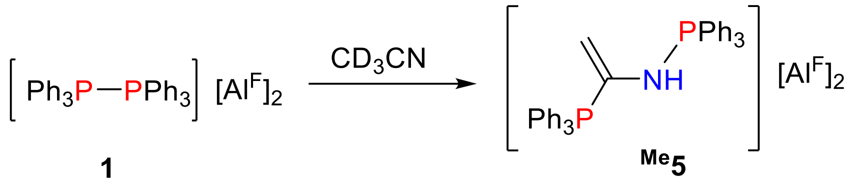

In a *J. Young* capped NMR tube, **1** (17 mg, 0.007 mmol) was dissolved in acetonitrile-*d*$_3$ (or proteo-acetonitrile), which led to the clean conversion to $^{\mathbf{Me}}$**5** over the course of two days. In the reaction with/in acetonitrile-*d*$_3$, a different set of signals appeared within the first hours in the ^{31}P{^{1}H} NMR spectrum. We attribute the two doublets at δ = 34.8 ppm ($^4J_{PP}$ = 78 Hz) and δ = 24.4 ppm ($^4J_{PP}$ = 78 Hz) to the transient imine, which then tautomerizes to $^{\mathbf{Me}}$**5**. After 48 h, the solvent was removed *in vacuo* and the compound was crystallized by adding dichloromethane (0.2 mL), which led to the formation of single crystals. These colorless crystals, which are suitable for sc-XRD, were collected and dried *in vacuo* (4 mg, 23%).

^{31}P{^{1}H} NMR (CD_3CN, 162.0 MHz): δ = 40.7 (d, $^4J_{PP}$ = 17 Hz, N*P*Ph$_3$), 25.9 (d, $^4J_{PP}$ = 17 Hz, C*P*Ph$_3$) ppm.

^{1}H NMR (CD_3CN, 400.1 MHz): δ = 7.95 (tr, $^3J_{HH}$ = 8 Hz, 6H, C*H*$_{Ar}$), 7.75–7.70 (m, 12H, C*H*$_{Ar}$), 7.67–7.53 (m, 12H, C*H*$_{Ar}$) 6.88 (d, $^2J_{HP}$ = 8 Hz, 1H, *H*NPPh$_3$), 6.26 (dddd, $^3J_{HP}$ = 37.6 Hz, $^2J_{HH}$(geminal) = 4.7 Hz, $^4J_{HH}$(allyl) = 1.3 Hz, $^4J_{HP}$(allyl) = 1.3 Hz, 1H, H$_{cis}$*H*$_{trans}$CCPPh$_3$), 5.94 (ddd, $^3J_{HP}$ = 13.7 Hz, $^2J_{HH}$(geminal) = 4.7 Hz, $^4J_{HP}$(allyl) = 1.8 Hz, 1H, *H*$_{cis}$H$_{trans}$CCPPh$_3$) ppm.

^{13}C{^{1}H} NMR (CD_3CN, 100.6 MHz): 137.4 (d, $^4J_{CP}$ = 3 Hz, *p*-CH$_{Ar}$), 137.3 (d, $^4J_{CP}$ = 3 Hz, *p*-CH$_{Ar}$), 136.1 (d, $^3J_{CP}$ = 11 Hz, *m*-CH$_{Ar}$), 135.2 (d, $^3J_{CP}$ = 11 Hz, *m*-CH$_{Ar}$), 134.8 (d, $^3J_{CP}$ = 5 Hz, *C*H$_2$CPPh$_3$)131.6 (d, $^2J_{CP}$ = 21 Hz, *o*-CH$_{Ar}$), 131.5 (d, $^2J_{CP}$ = 21 Hz, *o*-CH$_{Ar}$), 127.6 (dd, $^1J_{CP}$ = 96 Hz, $^3J_{CP}$ = 4 Hz CH$_2$CPPh$_3$) 122.2 (q, $^1J_{CF}$ = 292 Hz, –CF$_3$), 118.7 (d, $^1J_{CP}$ = 88 Hz, *ipso*-CH$_{Ar}$, partly superimposed by CD_3CN), 115.6 (d, $^1J_{CP}$ = 88 Hz, *ipso*-CH$_{Ar}$) ppm.

^{19}F NMR (CD_3CN, 282.4 MHz): δ = −76.0 (s, –C*F*$_3$), ppm.

ESI-HRMS (in Acetonitrile):
m/z calcd. for $C_{38}H_{33}NP_2^+$: 565.2088; found: 568.2073.
m/z calcd. for $C_{38}H_{34}NP_2^+$: 565.2166; found: 568.2150.

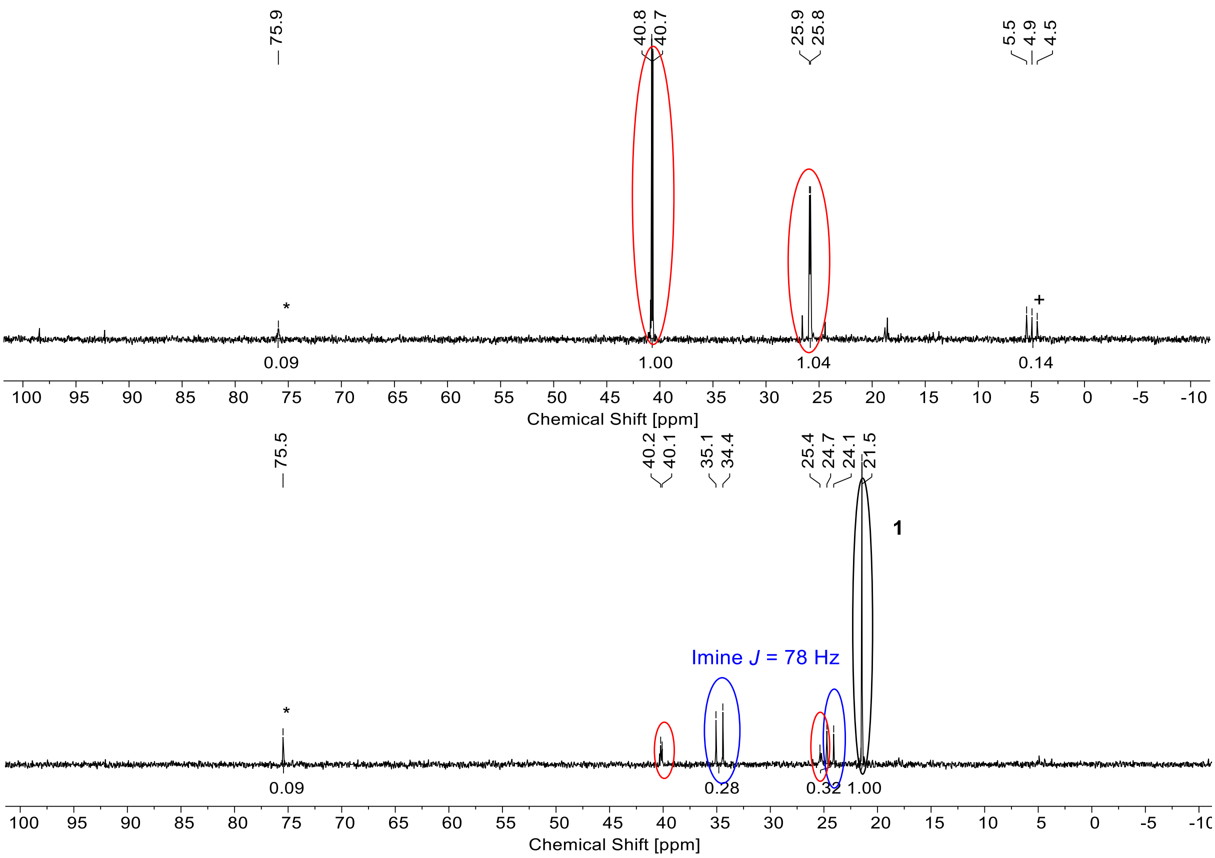

Figure S81. The $^{31}P\{^1H\}$ NMR spectrum (CD_3CN, 121.5 MHz, D1 = 30 s) obtained after 6 hours (bottom) shows the formation of the intermediate imine, and respective spectrum obtained after 48 hours (top) indicates the quantitative conversion to **^{Me}5** (* assigns $[Ph_3POPPh_3]^{2+}$, + assigns $[Ph_3PH]^+$; Color coding: blue, intermediate imine; red, **^{Me}5**).

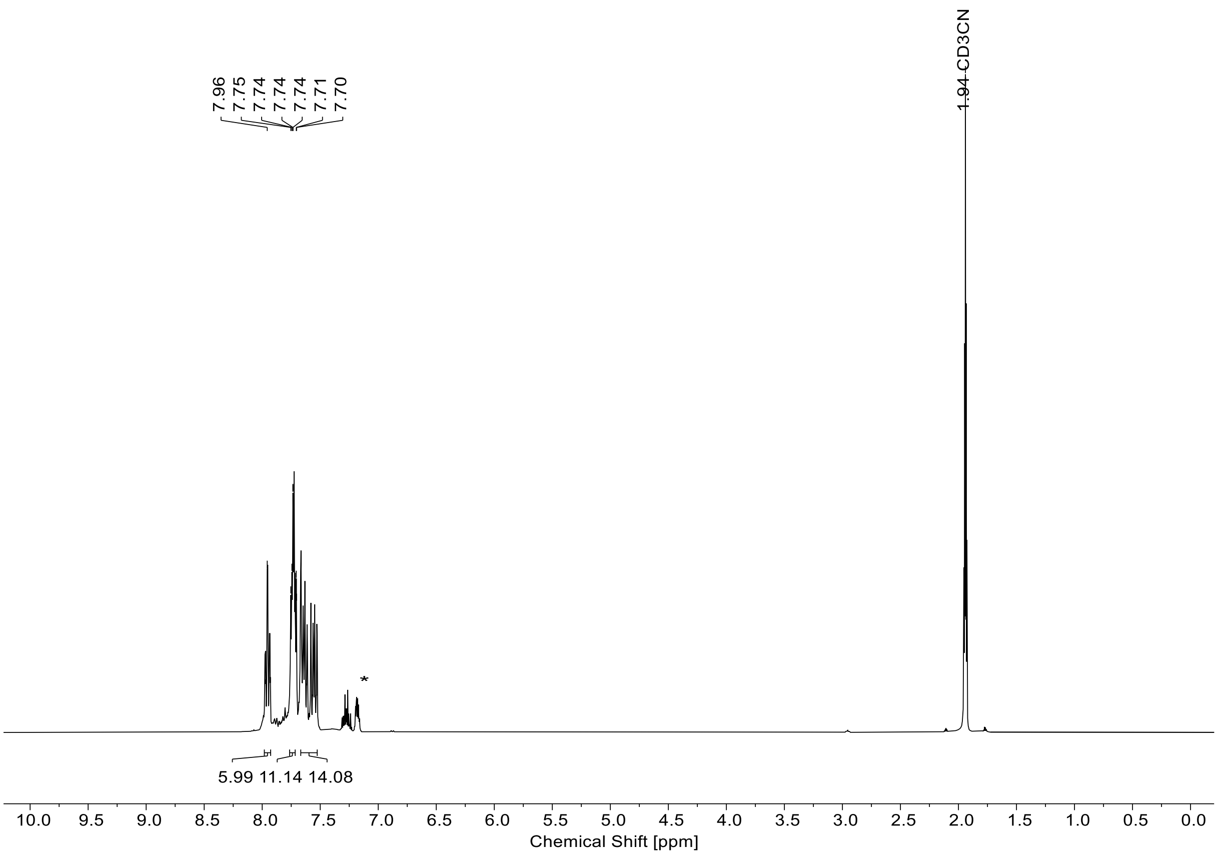

Figure S82. Crude ^{1}H NMR spectrum of ^{Me}**5** in acetonitrile-d_3 (CD_3CN, 400.1 MHz) (* = *o*DFB).

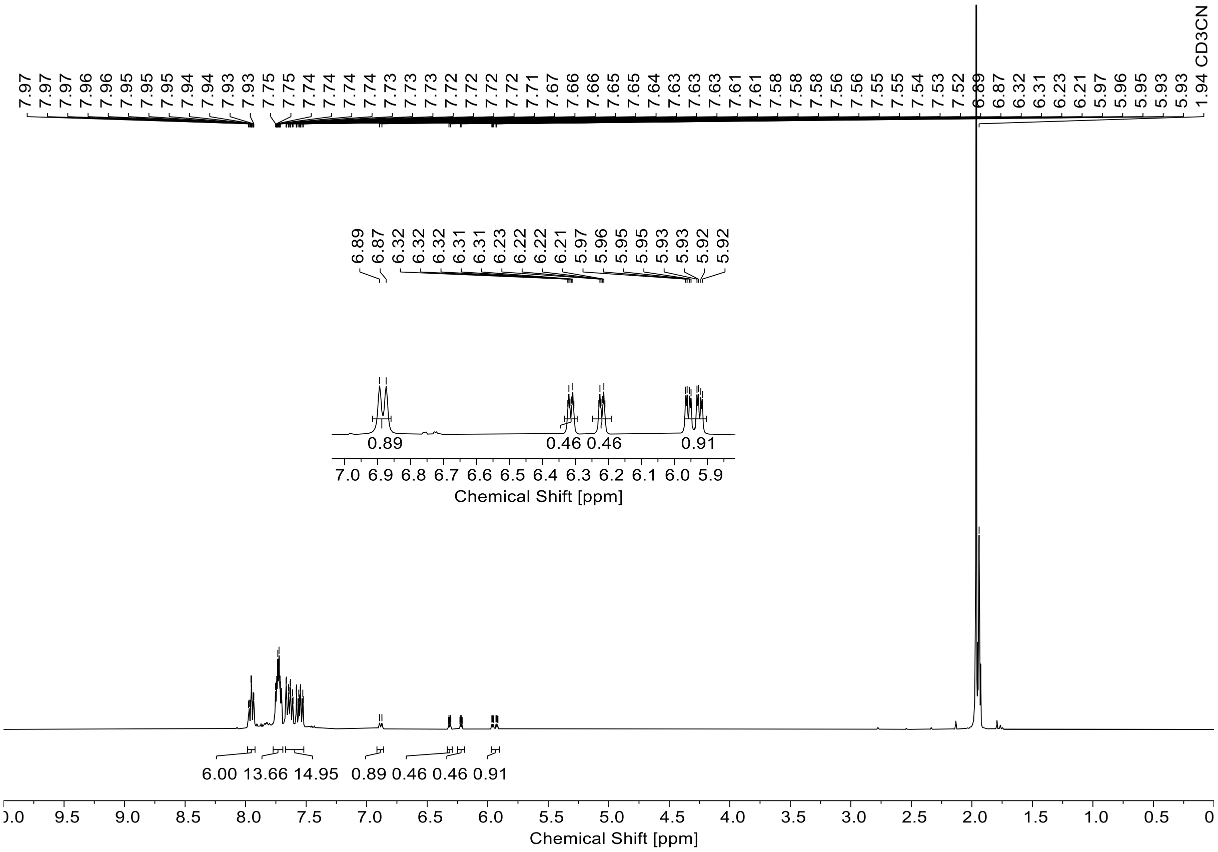

Figure S83. ^{1}H NMR spectrum of ^{Me}**5** isolated from the reaction in proteo-acetonitrile (CD_3CN, 400.1 MHz).

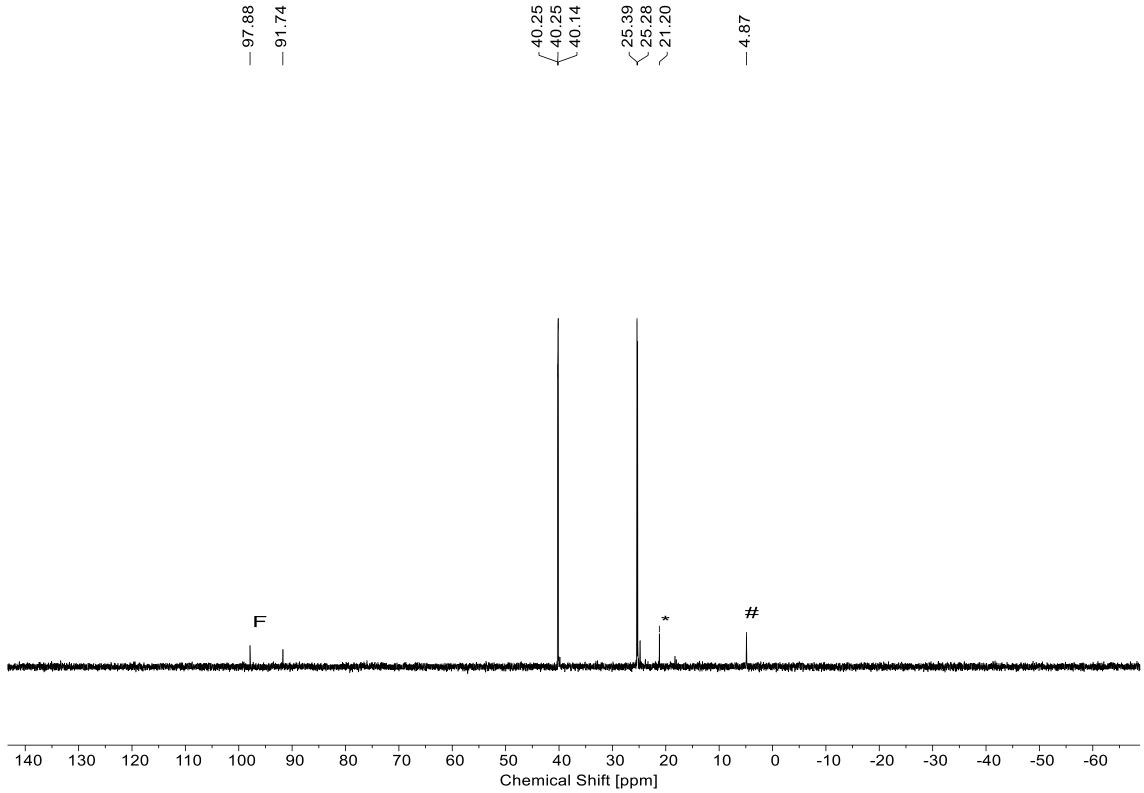

Figure S84. $^{31}P\{H\}$ NMR spectrum of **^{Me}5** (CD_3CN, 162.0 MHz; F = $[PPh_3F]^+$; * = $OPPh_3$; # = $[Ph_3PH]^+$).

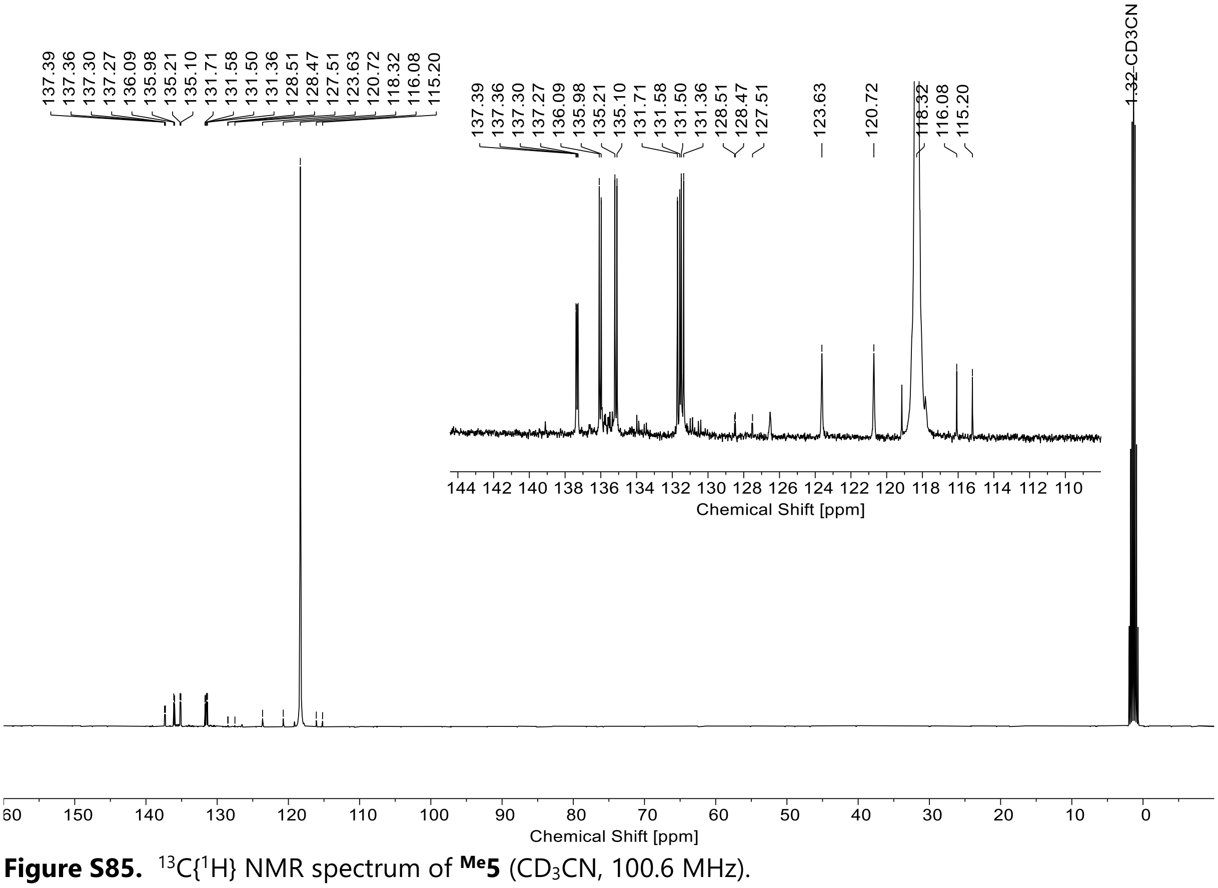

Figure S85. $^{13}C\{^1H\}$ NMR spectrum of **^{Me}5** (CD_3CN, 100.6 MHz).

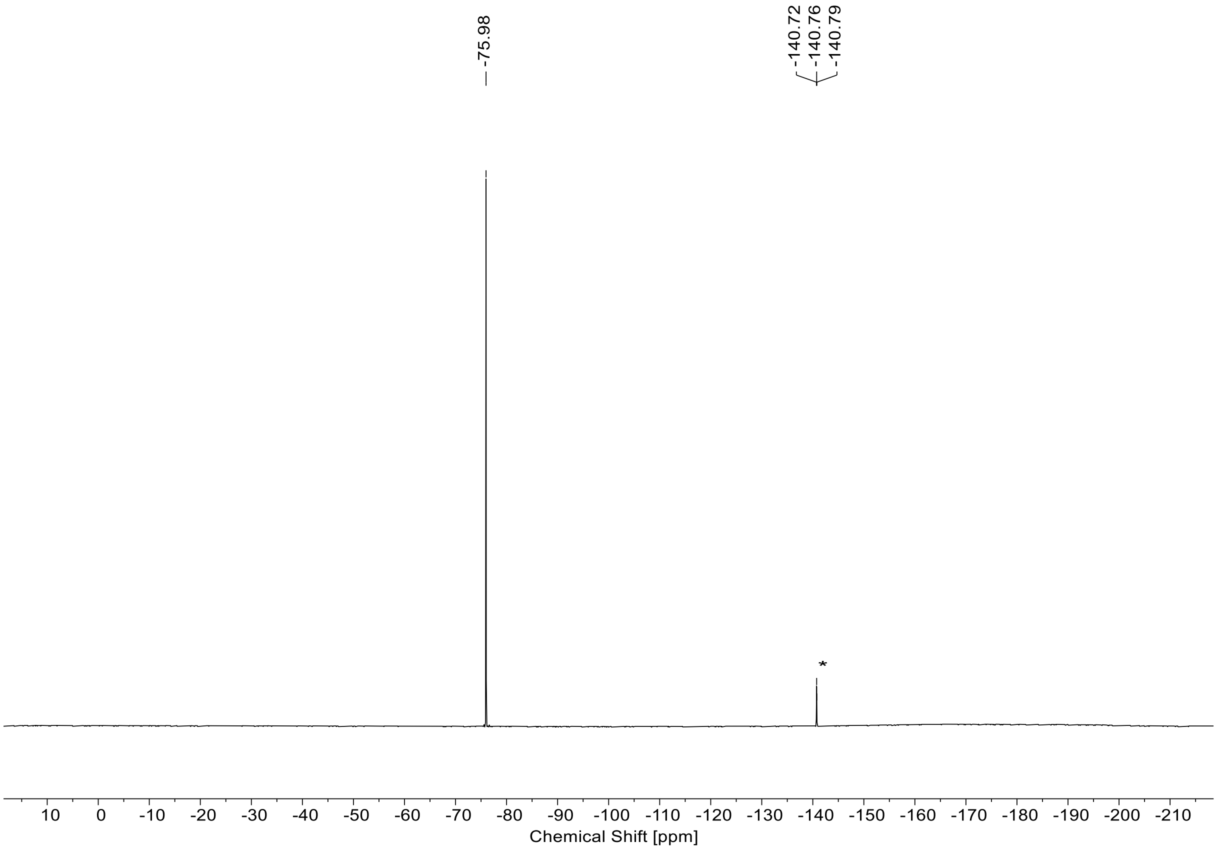

Figure S86. ^{19}F NMR spectrum (CD_3CN 282.4 MHz) of **[Me]5** (* = *o*DFB).

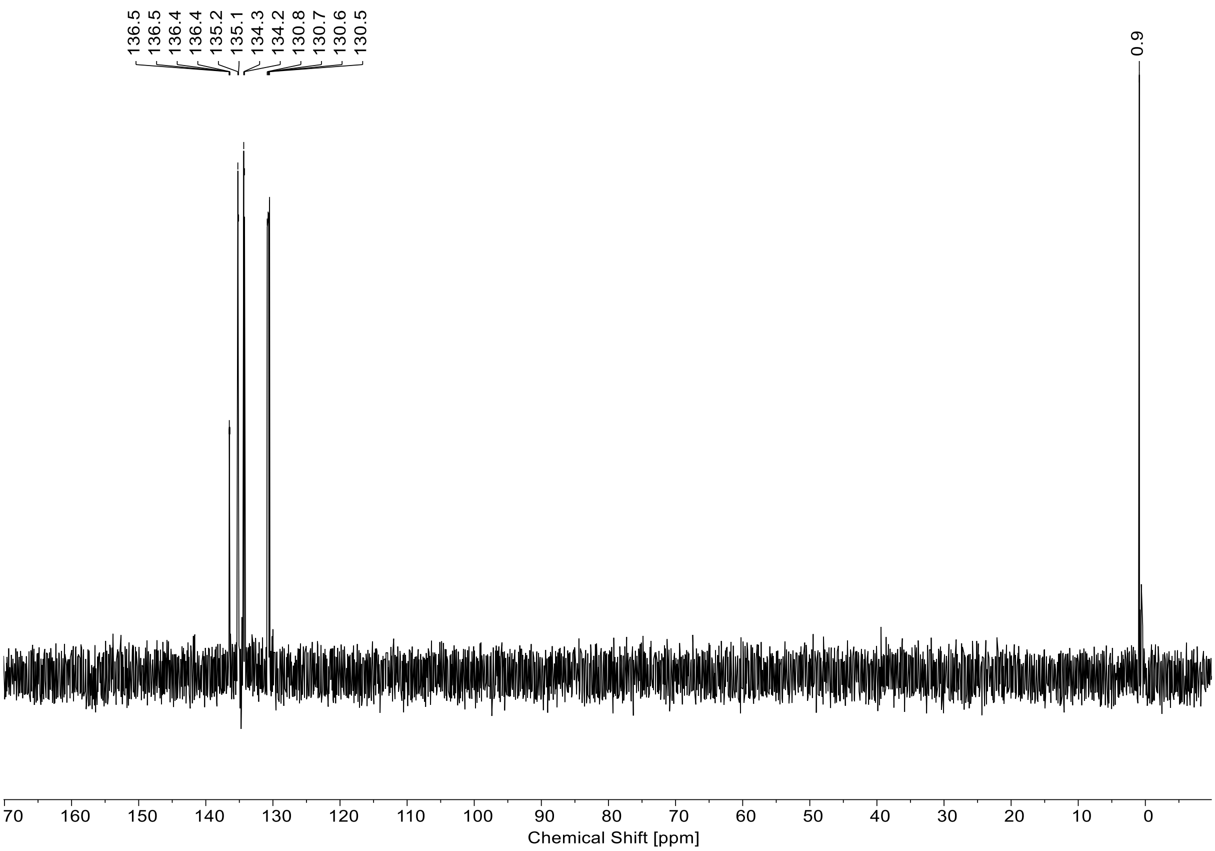

Figure S87. DEPT-135 NMR spectrum of **[Me]5** (CD_3CN, 100.6 MHz).

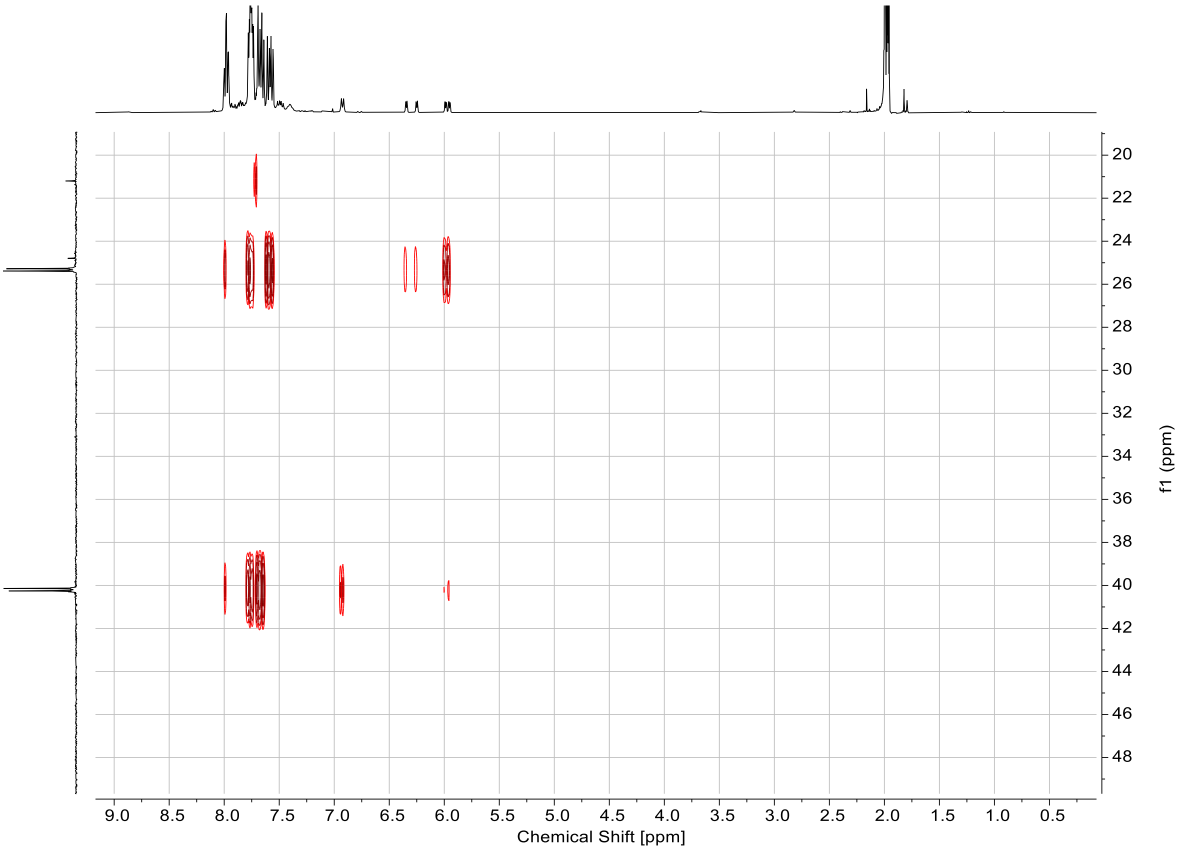

Figure S88. ^{1}H–^{31}P correlated HMBC NMR spectrum of **^{Me}5** (CD_3CN, 400.1/162.0 MHz).

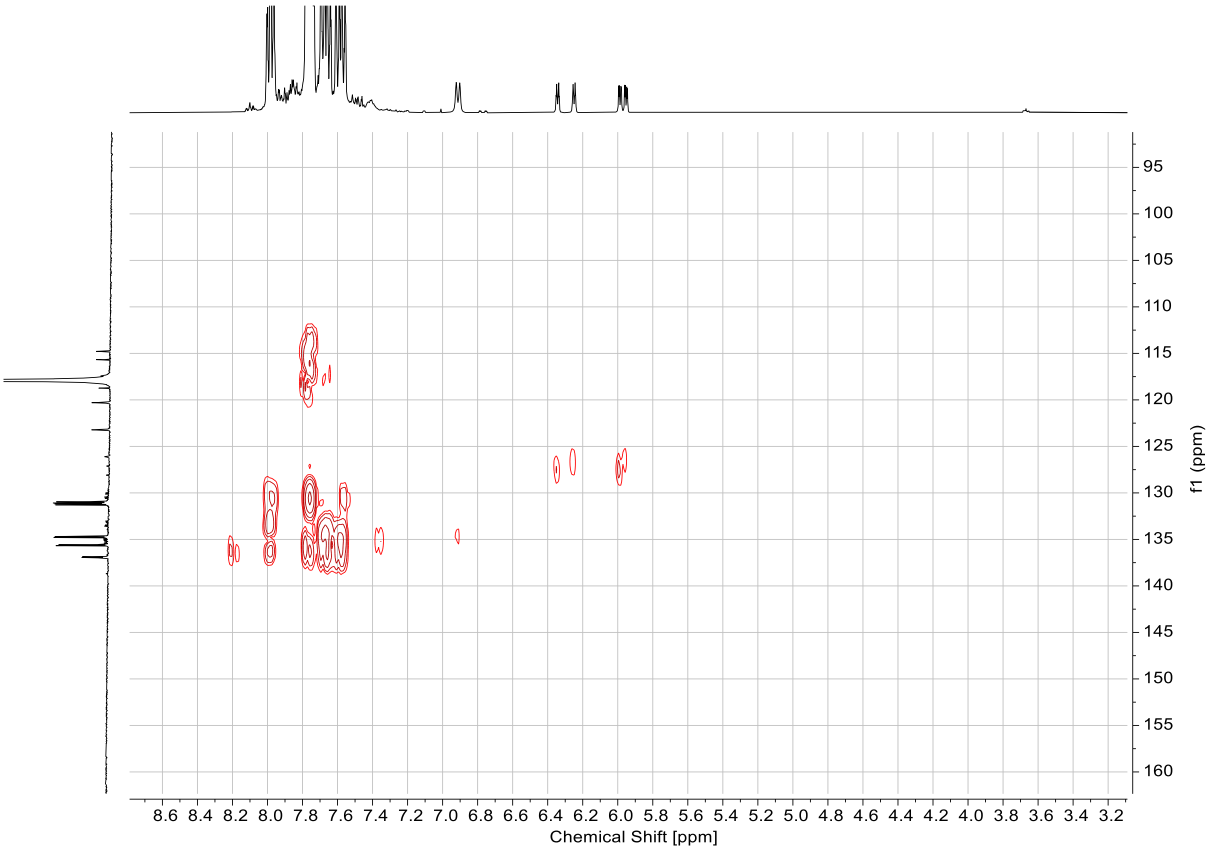

Figure S89. ^{1}H–^{13}C correlated HMBC NMR spectrum of **^{Me}5** (CD_3CN, 400.1/100.6 MHz).

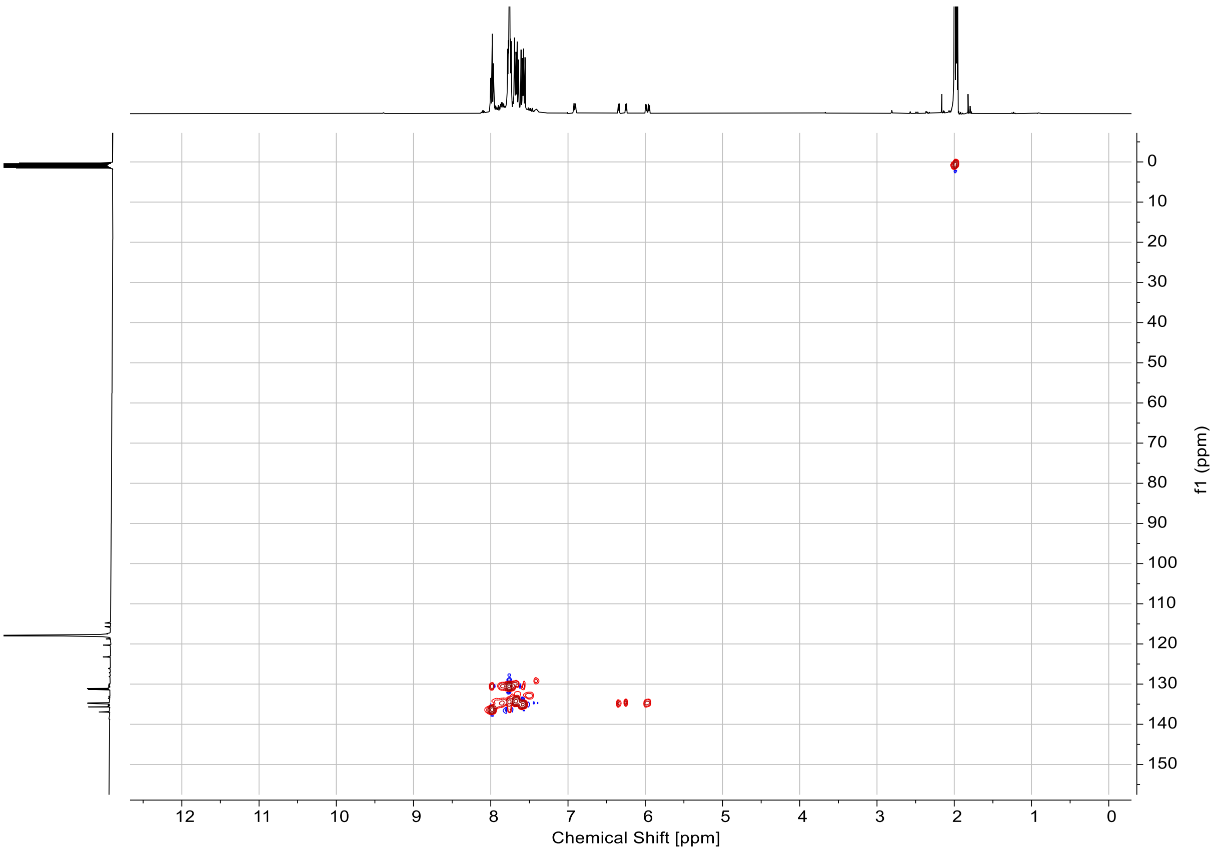

Figure S90. ^{1}H–^{13}C correlated HSQC NMR spectrum of **^{Me}5** (CD_3CN, 400.1/100.6 MHz).

3 Irradiation, EPR and Heating of 1

Heating and irradiation experiments were carried out in *J. Young* valve capped NMR tubes. The samples were irradiated with a xenon lamp (Quantum Design, LSE140/160.25C, 150 W). Saturated solutions of **1** were prepared by suspending **1** in 0.4 mL *o*DFB, and filtering over a glass fiber filter into another NMR tube (2.5 mg mL^{-1}).

3.1 EPR analysis of 1

Samples of **1** dissolved in *o*DFB proved unsuitable for the acquisition of EPR spectra due to tuning failure. Compound **1** was hence suspended in CH_2Cl_2 and dissolved by addition of NaBArF[20]. The EPR spectroscopic analysis indicated the absence of paramagnetic signatures. Irradiation in the cavity of the EPR instrument at room temperature neither did afford a signal in the EPR spectroscopic analysis, hence indicating that formed radicals are short-lived.

3.2 Irradiation and Heating of 1 in Dichloromethane

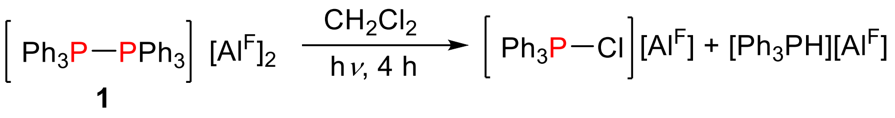

In a *J*-Young NMR tube, 8 mg of $[Ph_3P–PPh_3][Al^F]_2$ (**1**, 0.003 mmol) were suspended in 0.5 ml of CH_2Cl_2, which contained a capillary filled with triethylphosphineoxide in $CDCl_3$ as standard for the ^{31}P NMR spectroscopic analysis. The solution was heated in the dark under continuous shaking to 50 °C overnight, and the ^{31}P NMR spectroscopic analysis indicated the absence of any conversion. The reaction mixture was then irradiated at room temperature for 1 h under continuous shaking, upon which the mixture changed from colorless to pale-yellow, with still a moderate amount of colorless precipitate present. The NMR spectroscopic analysis indicated the formation of $[Ph_3PH]^+$ and $[Ph_3PCl]^+$. Further irradiation for 3 h led to a homogeneous yellow solution, which was also analyzed by NMR spectroscopies.

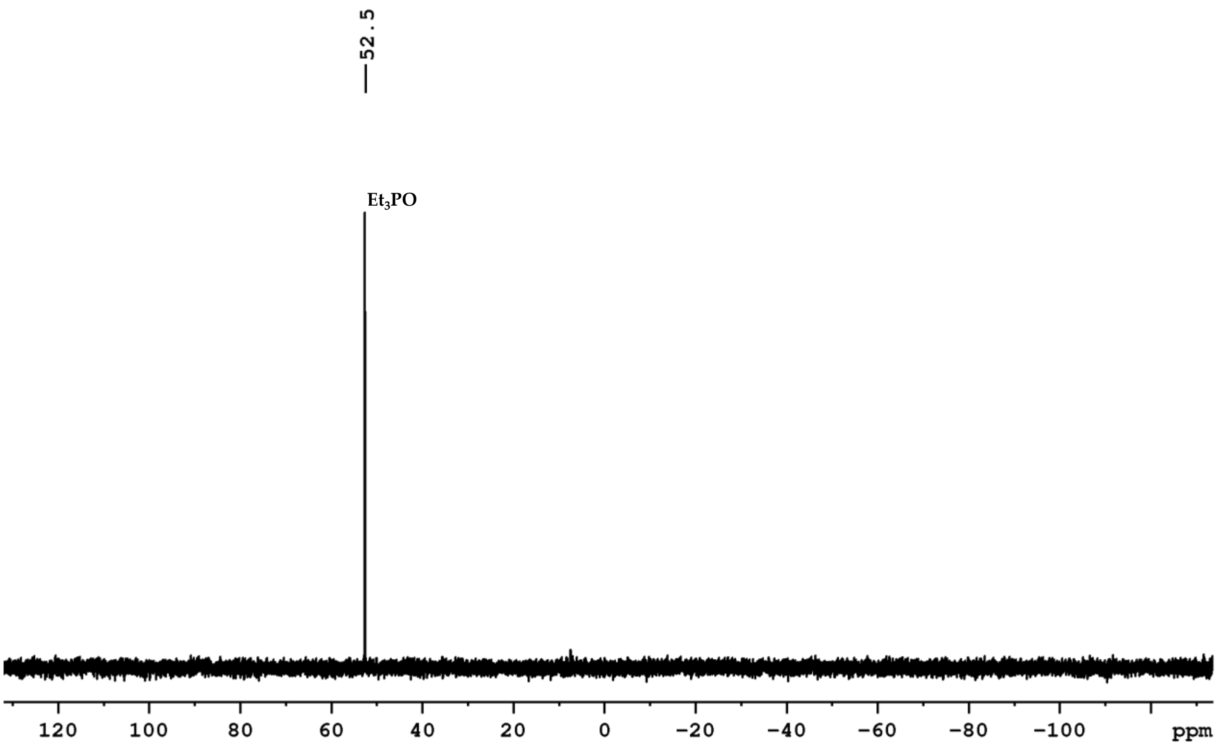

Figure S91. $^{31}P\{^1H\}$ NMR spectrum (CH_2Cl_2 with capillary containing Et_3PO as an external standard in $CDCl_3$, 162.0 MHz, D1 = 30s) of supernatant after refluxing of **1** in CH_2Cl_2 overnight. The spectrum only shows the Et_3PO standard and hence indicates the absence of a reaction, with the insoluble residue being the starting material.

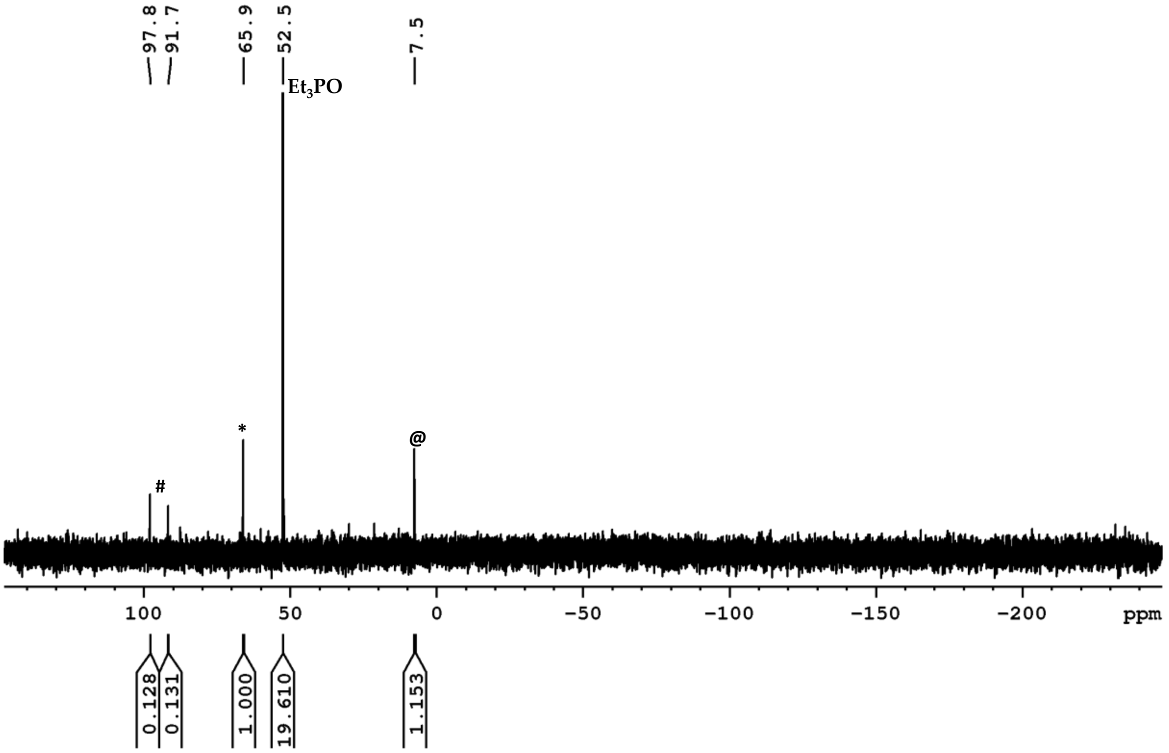

Figure S92. $^{31}P\{^{1}H\}$ NMR spectrum (CH_2Cl_2 with capillary containing Et_3PO as an external standard in $CDCl_3$, 162.0 MHz, D1 = 30s) of reaction mixture obtained after irradiation of **1** in CH_2Cl_2 for 1 h (#Ph_3PF^+, *PPh_3Cl^+, @Ph_3PH^+).

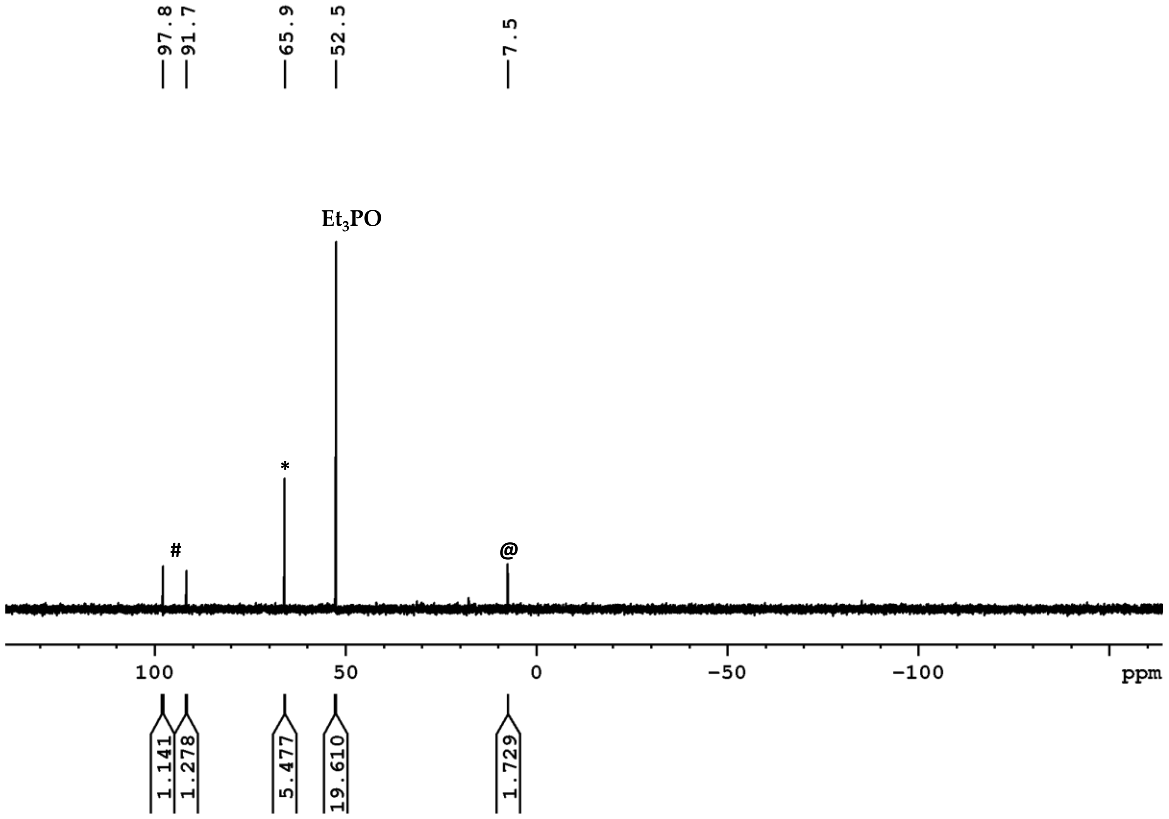

Figure S93. $^{31}P\{^{1}H\}$ NMR spectrum (CH_2Cl_2 with capillary containing Et_3PO as an external standard in $CDCl_3$, 162.0 MHz, D1 = 30s) of reaction mixture obtained after irradiation of **1** in CH_2Cl_2 for 4 h (#Ph_3PF^+, *PPh_3Cl^+, @Ph_3PH^+).

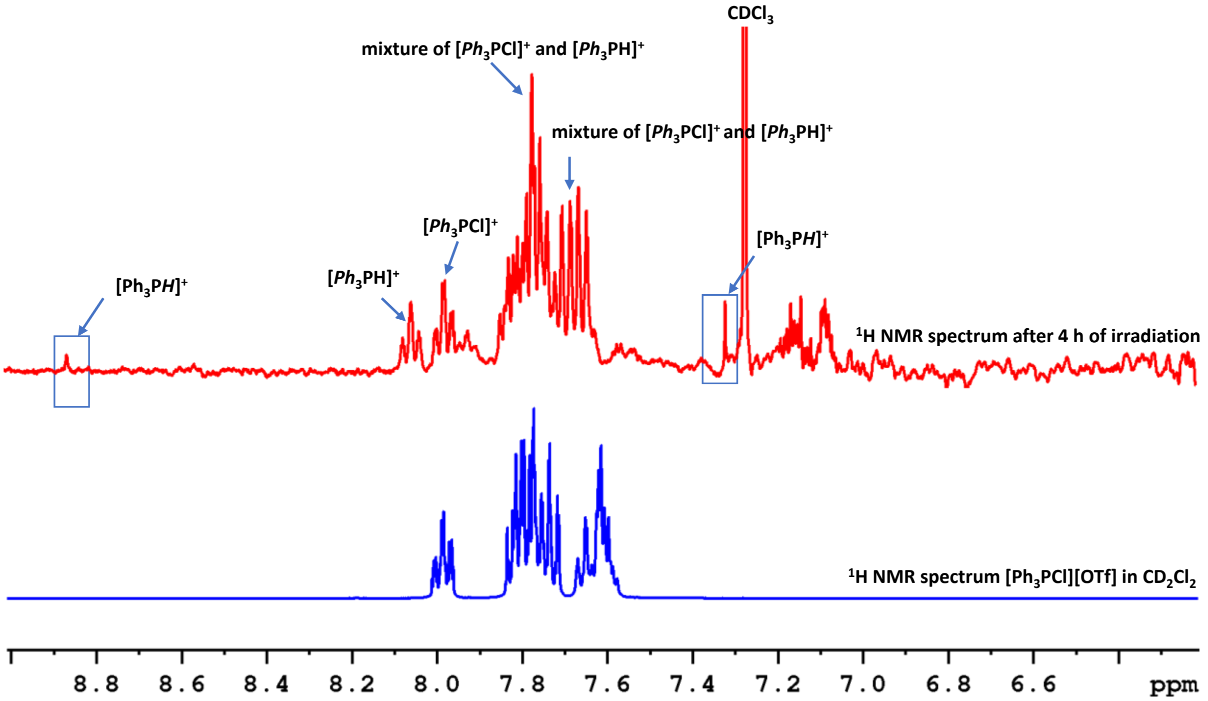

Figure S94. ^{1}H NMR spectrum (CH_2Cl_2 with capillary containing Et_3PO as an external standard in $CDCl_3$, 400.1 MHz, D1 = 30 s) of reaction mixture obtained after irradiation of **1** in CH_2Cl_2 for 4 h, stacked with the reference spectrum of $[Ph_3PCl][OTf]$

3.3 Irradiation of 1 in the Presence of DHA

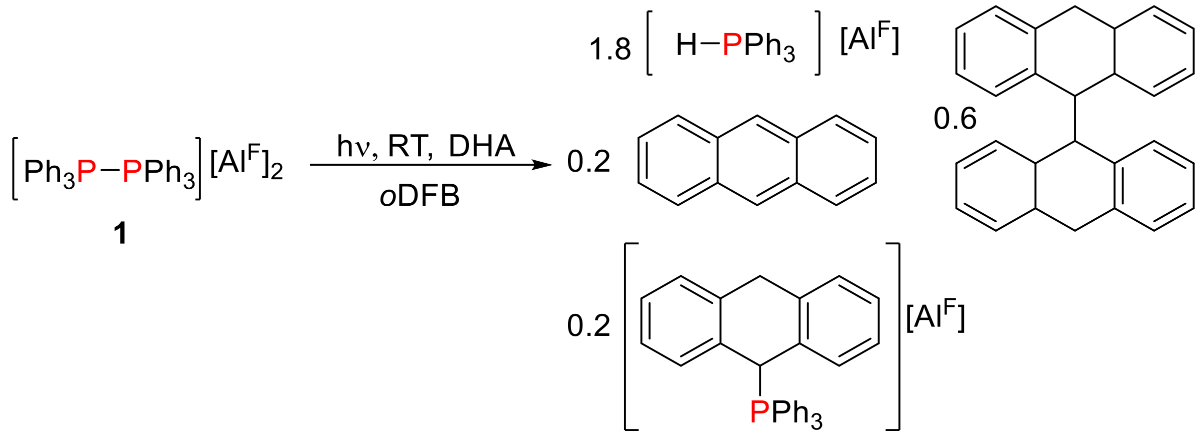

9,10-Dihydroanthracene (DHA, 3 mg, 0.017 mmol) was added to a *J. Young* capped quartz-NMR tube containing a saturated solution of **1** in *o*DFB (0.4 mL). The sample was irradiated, and the reaction progress was monitored by $^{31}P\{^1H\}$ and ^1H NMR spectroscopies. After quantitative conversion (30 minutes) to $[Ph_3PH]^+$, the solvent was removed *in vacuo* and C_6D_6 was added. After the $^{31}P\{^1H\}$ and ^1H NMR spectroscopic analysis, KHMDS (1 mg, 0.005 mmol) was added, which led to the formation of PPh_3 by deprotonating $[Ph_3PH]^+$.

Crude NMR mixture:

^{1}H NMR (*o*DFB with DMSO-d_6 capillary, 400.1 MHz): δ = 7.49 (d, 1H, $^1J_{HP}$ =496 Hz, Ph_3PH, partly superimposed by solvent), 7.03–6.95 (m, 3H, CH_{Ar}), 6.89–6.84 (m, 10H, CH_{Ar}) ppm

^{31}P{^{1}H} NMR (*o*DFB with DMSO-d_6 capillary, 162.0MHz): δ = 19.5 (s, Ph_3PAnt), 7.7 (s, Ph_3PH) ppm.

^{31}P NMR (*o*DFB with DMSO-d_6 capillary, 162.0 MHz): δ = 7.7 (d, $^1J_{HP}$ = 495 Hz, Ph_3PH) ppm.

After extraction with C_6D_6:

^{1}H NMR (C_6D_6, 400.1 MHz): δ = 8.16 (s, 2H, C*H*, anthracene), (m, 4H, C*H*, anthracene), 7.26 (m, 4H, C*H*, anthracene, superimposed), 6.30 (d, $^3J_{HP}$ = 496 Hz, 9H, Ph3PH, partly superimposed), 4.32 (s, 6H, 9,9´,10,10´-tetrahydrodianthranyl-9,9') 3.16 (d, 6H, $^2J_{HH}$ = 19 Hz, 9,9',10,10'-tetrahydrodianthranyl-9,9'); 2.43 (d, 6H, $^2J_{HH}$ = 19 Hz, 9,9',10,10'-tetrahydrodianthranyl-9,9') ppm.

^{31}P{^{1}H} NMR (C_6D_6, 162.0MHz): δ = 6.1 (s, Ph_3PH^+) ppm.

^{31}P NMR (C_6D_6, 162.0 MHz): δ = 19.8 (s, Ph$_3$*P*Ant), 6.1 (d,$^1J_{HP}$ = 495 Hz, Ph$_3$*P*H$^+$) ppm.

APPI-HRMS (in C_6D_6):

m/z calcd. for $C_{18}H_{16}P^+$: 263.0990; found 263.0999.

m/z calcd. for $C_{10}H_{14}^+$: 178.0783; found 178.0784.

m/z calcd. for $C_{46}H_{40}P^+$: 623.2868; found 623.2898 (weak signal).

ESI-HRMS (in C_6D_6):

m/z calcd. for $C_{32}H_{26}P^+$: 441.1772; found 441.1775.

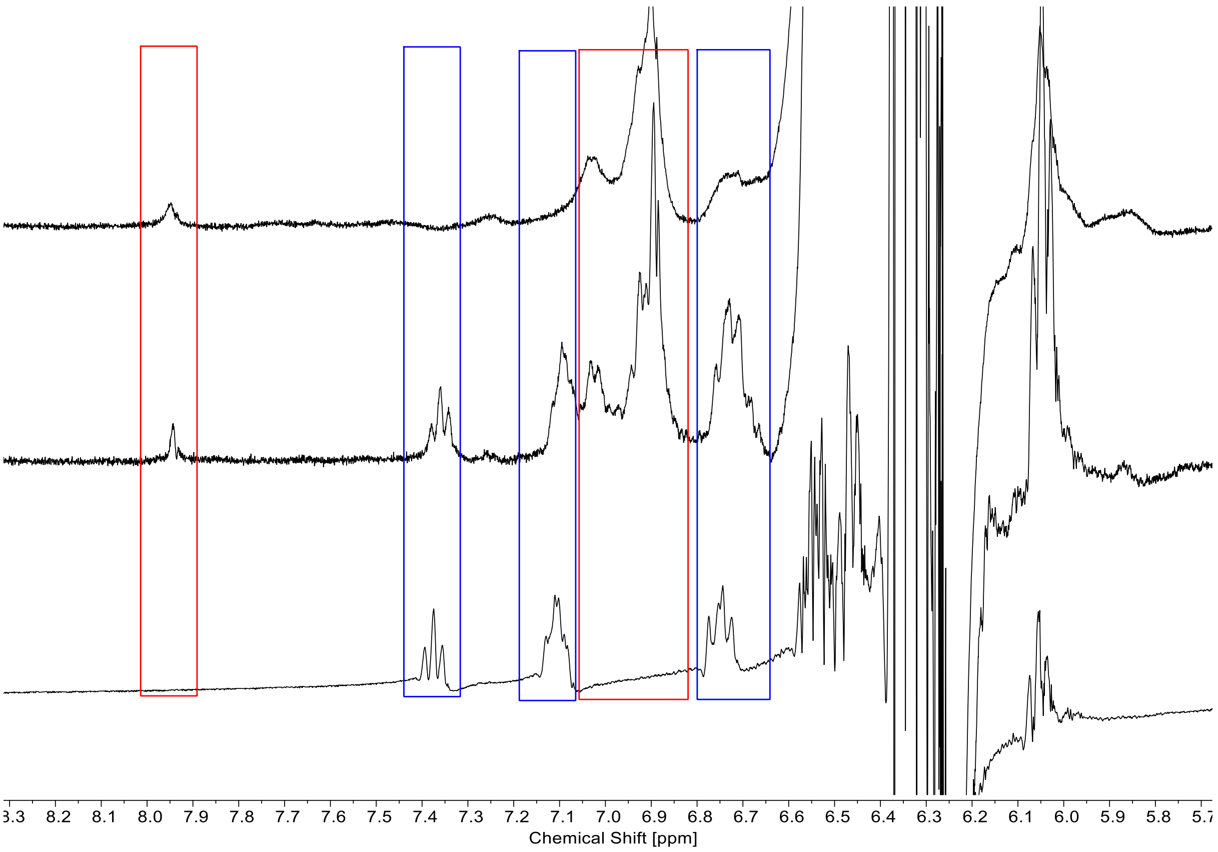

Figure S95. ^{1}H NMR spectrum (*o*DFB with DMSO-d_6 capillary, 400.1 MHz) prior to the irradiation of **1** in the presence of DHA (bottom), and spectra obtained after 15 minutes (middle) and 30 minutes (top) of irradiation. Color coding: red, **1**; blue, $[PPh_3H]^+$.

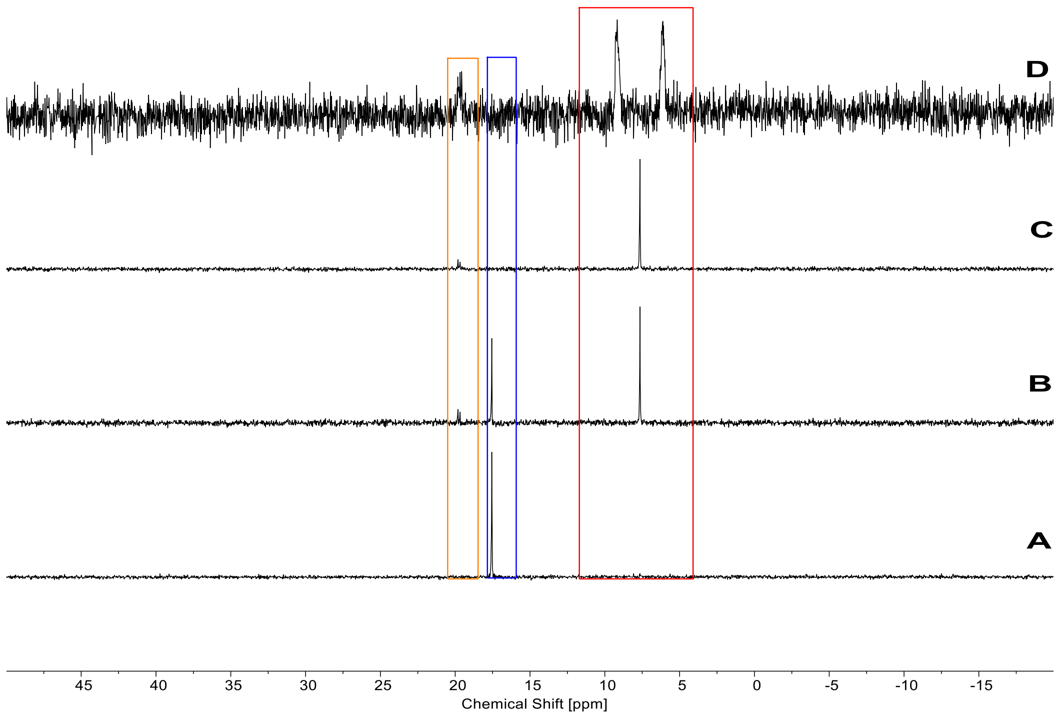

Figure S96. Stacked $^{31}P\{^{1}H\}$ NMR spectra (*o*DFB, 162.0 MHz, DMSO-d_6 capillary) of the irradiation experiment of **1** in the presence of DHA: $^{31}P\{^{1}H\}$ NMR spectrum prior to the irradiation (A), after 15 minutes (B), after 30 minutes (C), ^{31}P NMR spectrum after 30 minutes (D). Color coding: blue, **1**; red, $[Ph_3PH]^+$; orange, side product assigned to the phosphonium salt [anthracenyl–PPh_3][Al^F] (the corresponding mass was also found in the HRMS experiment; *vide supra*) resulting from the coupling of the transient triphenylphosphine radical with the 9-hydroanthracene radical.

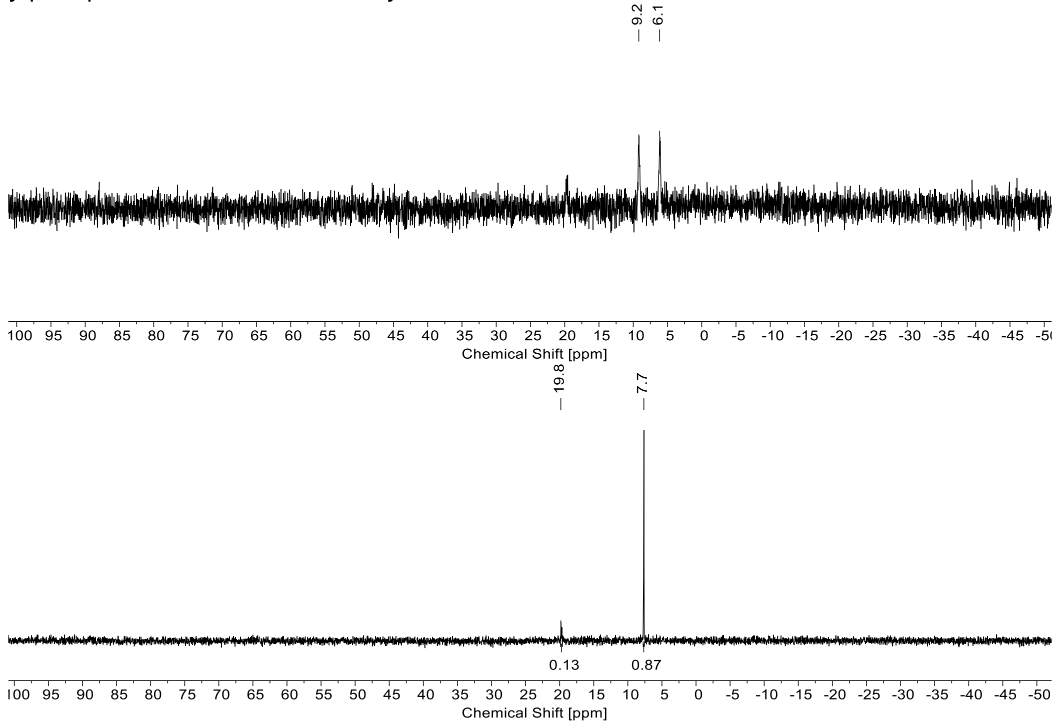

Figure S97. $^{31}P\{H\}$ NMR spectrum (bottom, *o*DFB with DMSO-d_6 capillary, 162.0 MHz, D1 = 30 s) obtained after 30 min of irradiation, stacked with the ^{31}P NMR spectrum (*o*DFB with DMSO-d_6 capillary, 162.0 MHz). The minor side-product at 19.8 ppm is tentatively assigned to the phosphonium salt [anthracenyl–PPh_3][Al^F] (the corresponding mass was also found in the HRMS experiment; *vide supra*) resulting from the coupling of the transient triphenylphosphine radical with the 9-hydroanthracene radical.

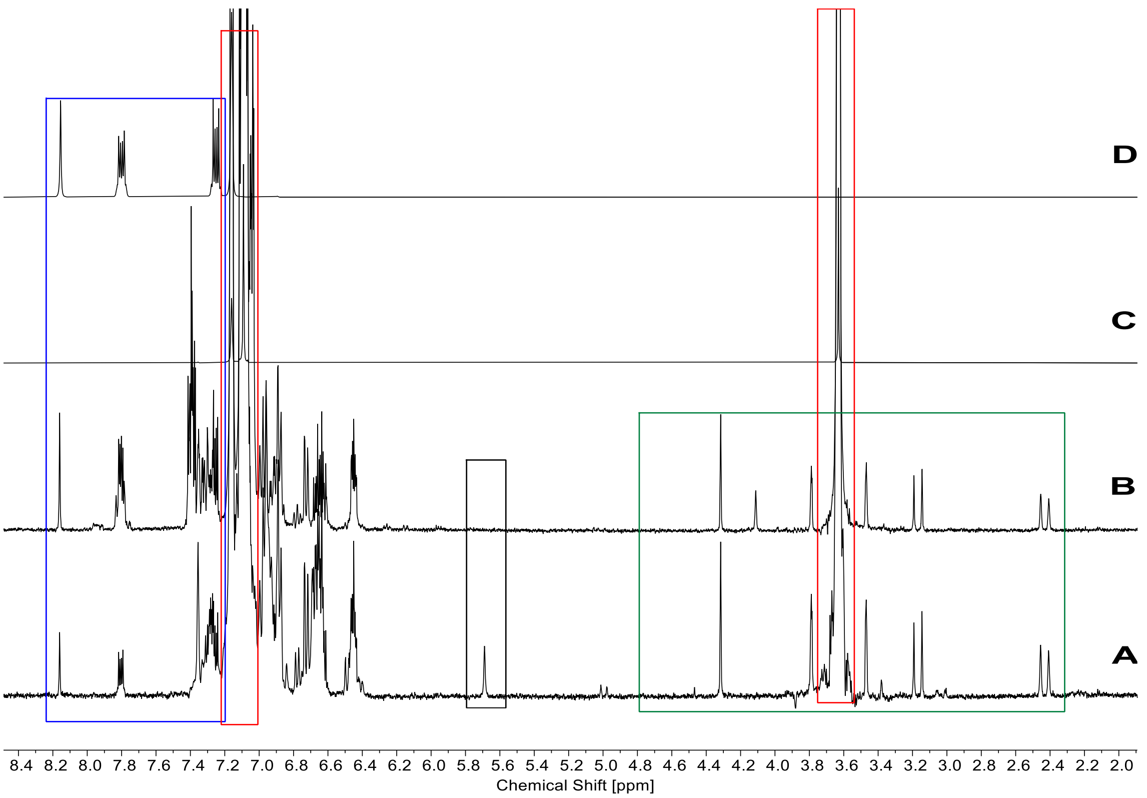

Figure S98. Stacked ^{1}H NMR spectra (C_6D_6, 400.1 MHz) of the C_6D_6 extract of the irradiation of **1** after quantitative conversion (A), after treating with KHMDS (B), and reference spectra of DHA (C) and anthracene (D). Color coding: blue, anthracene; red, DHA; black, one part of doublet corresponding to $[Ph_3PH]^+$; green, 9,9′,10,10′-tetrahydrodianthranyl-9,9′.[17]

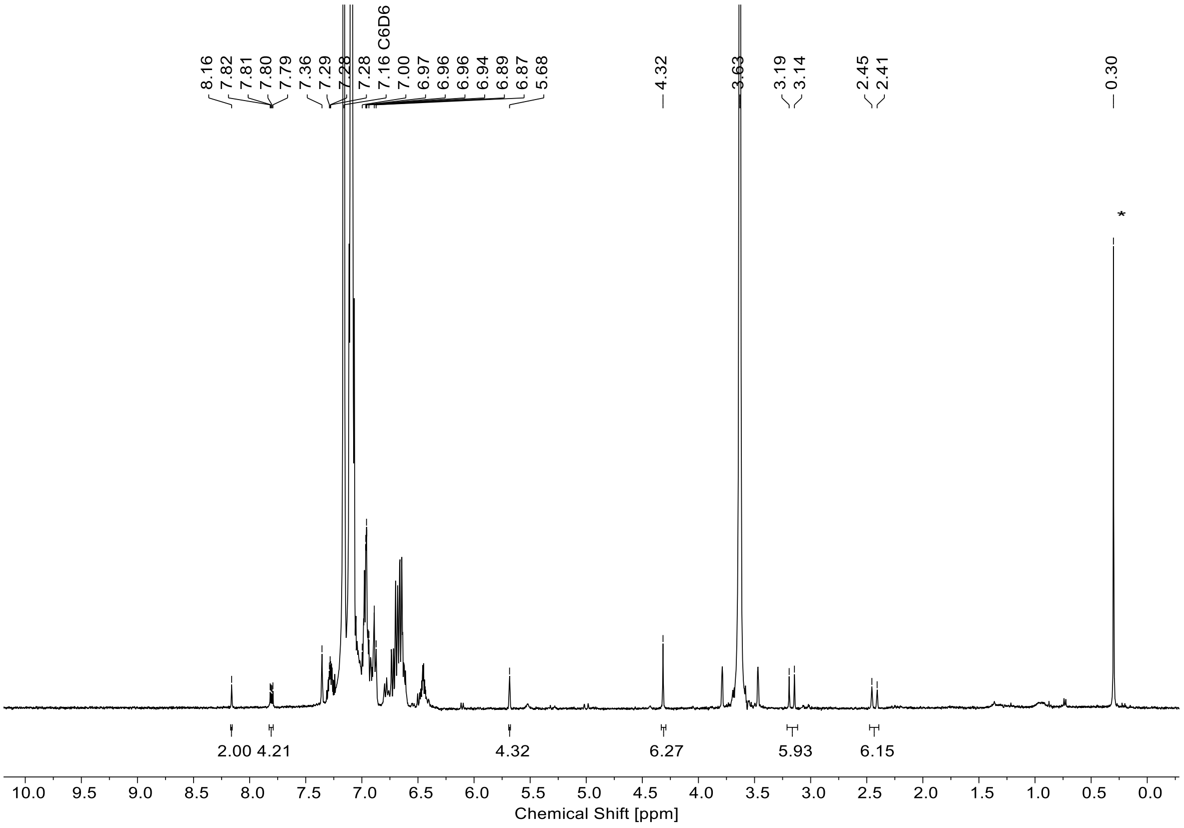

Figure S99. ^{1}H NMR spectrum of the C_6D_6 extract of the irradiation reaction (C_6D_6, 400 MHz, * = grease). The integration suggests the formation of a 1:3 mixture of anthracene and the anthracene C–C coupling product 9,9′,10,10′-tetrahydrodianthranyl-9,9′.[17]

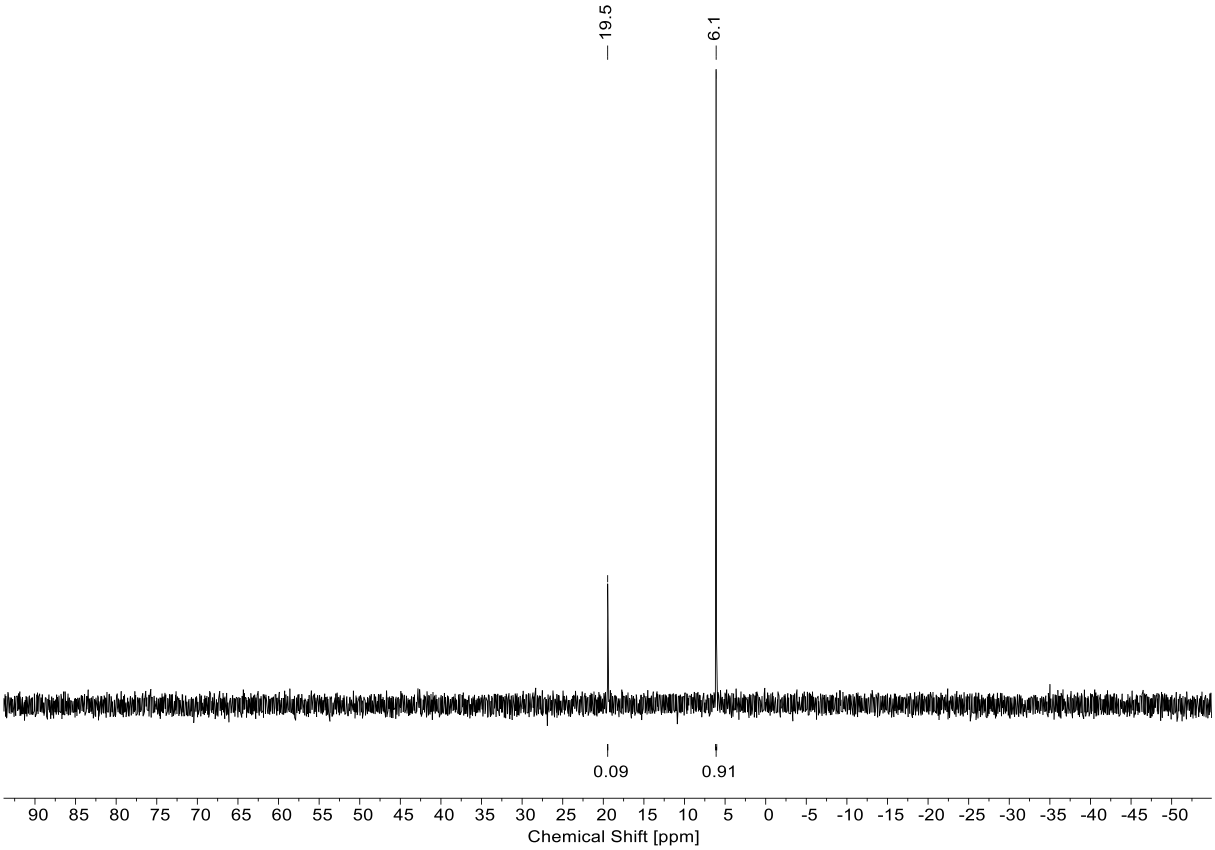

Figure S100. $^{31}P\{^{1}H\}$ NMR spectrum of the C_6D_6 extract of the irradiation reaction (C_6D_6, 162.0 MHz).

3.4 Irradiation of 1 in the Absence of DHA

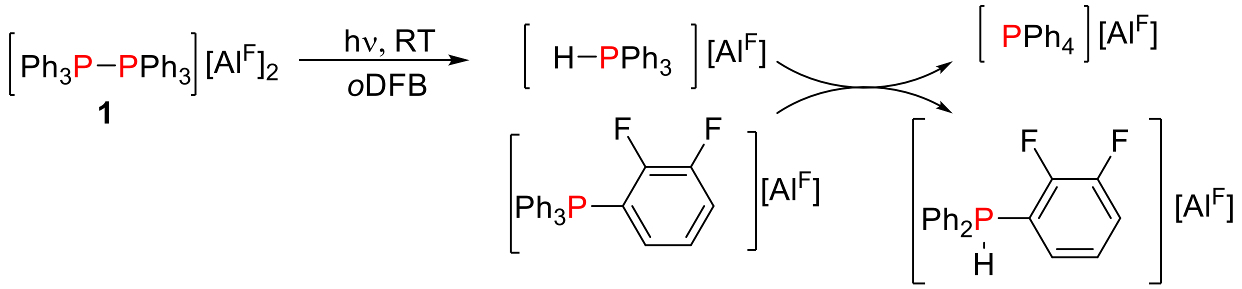

A saturated solution of **1** in *o*DFB (0.4 mL) was irradiated in a *J. Young* capped NMR tube, and the reaction progress was followed by $^{31}P\{^{1}H\}$ NMR spectroscopy. After three hours, quantitative conversion was obtained. After NMR spectroscopic analysis, the volatiles were removed *in vacuo* and the colorless residue was dissolved in $CDCl_3$. Phosphoranylation is indicated by the appearance of three doublets with $^{4}J_{PF}$ = 4.9 Hz (Figure S101). The doublet at δ = 7.6 ppm is assigned to $[Ph_2(C_6H_3F_2)PH]^+$, resulting from the ligand metathesis reaction of $[Ph_3(C_6H_3F_2)P]^+$ with $[Ph_3PH]^+$ under formation of $[PPh_4]^+$.[9]

^{31}P{^{1}H} NMR (*o*DFB with DMSO-*d*$_6$ capillary, 162.0 MHz): δ = 23.4 (d, $^{4}J_{PF}$ = 4.9 Hz, $[Ph_3(C_6H_3F_2)\mathit{P}]^+$), 22.7 (s, $[\mathit{P}Ph_4]^+$), 7.7 (s, $Ph_3\mathit{P}H$), 7.6 (d, $^{4}J_{PF}$ = 4.9 Hz, $[Ph_2(C_6H_3F_2)\mathit{P}H]^+$, ppm.

^{19}F NMR ($CDCl_3$, 282.4 MHz): δ = 121.6 (m, *o*-F, $[Ph_3(C_6H_3F_2)P]^+$), 129.1 ppm (m, *m*-F, $[Ph_3(C_6H_3F_2)P]^+$).

^{19}F{^{1}H} NMR ($CDCl_3$, 282.4 MHz): δ = 121.6 (d, $^{3}J_{FF}$ = 21.0 Hz, *o*-F, $[Ph_3(C_6H_3F_2)P]^+$), 129.1 ppm (dd, $^{3}J_{FF}$ = 21.0 Hz, $^{4}J_{FP}$ = 4.9 Hz, *m*-F, $[Ph_3(C_6H_3F_2)P]^+$,).

APPI-HRMS (in C_6D_6):
m/z calcd. for $C_{18}H_{16}P^+$: 263.0990; found 263.0999.
m/z calcd. for $C_{24}H_{18}F_2P^+$: 375.1114; found 375.1141.

ESI-HRMS (in $CDCl_3$):
m/z calcd. for $C_{24}H_{18}F_2P^+$: 375.1114; found 375.1137.
m/z calcd. for $C_{24}H_{20}P^+$: 339.1303; found 339.1327 (weak signal).

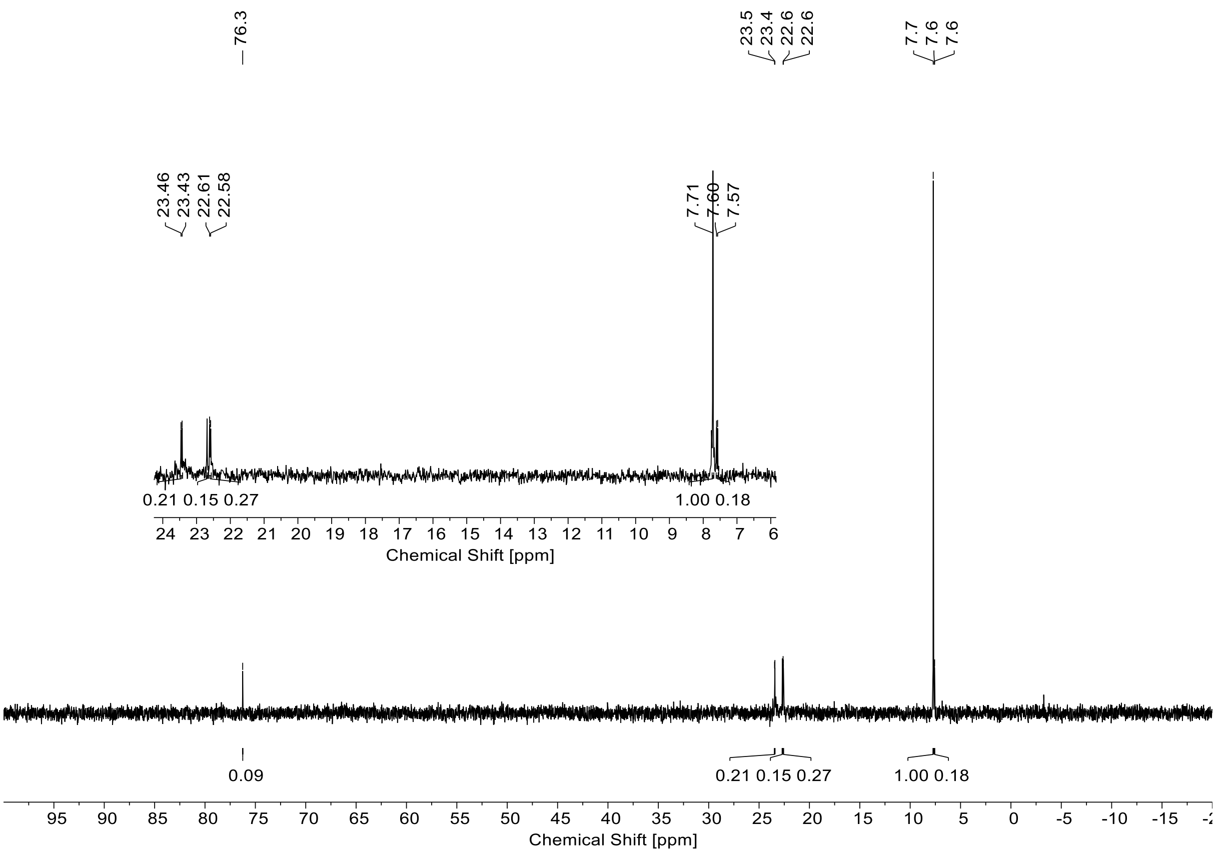

Figure S101. $^{31}P\{^{1}H\}$ NMR spectrum after the quantitative conversion to $[Ph_3PH]^+$ (*o*DFB with DMSO-d_6 capillary , 162.0 MHz, D1 = 30 s). The doublet at δ = 23.4 ppm is attributed to the phosphoranylation product $[Ph_3(C_6H_3F_2)P]^+$,[9] Doublets at δ =22.6 ppm and δ =7.6 ppm are attributed to the phosphonium salts $[Ph_2(C_6H_3F_2)_2P]^+$ and $[Ph_2(C_6H_3F_2)PH]^+$, resulting from ligand metathesis reactions, in line with the formation of $[PPh_4]^+$ (s at δ =22.7 ppm). The signal at δ = 76.3 ppm is assigned to $[Ph_3POPPh_3]^{2+}$ due to the adventitious presence of H_2O.

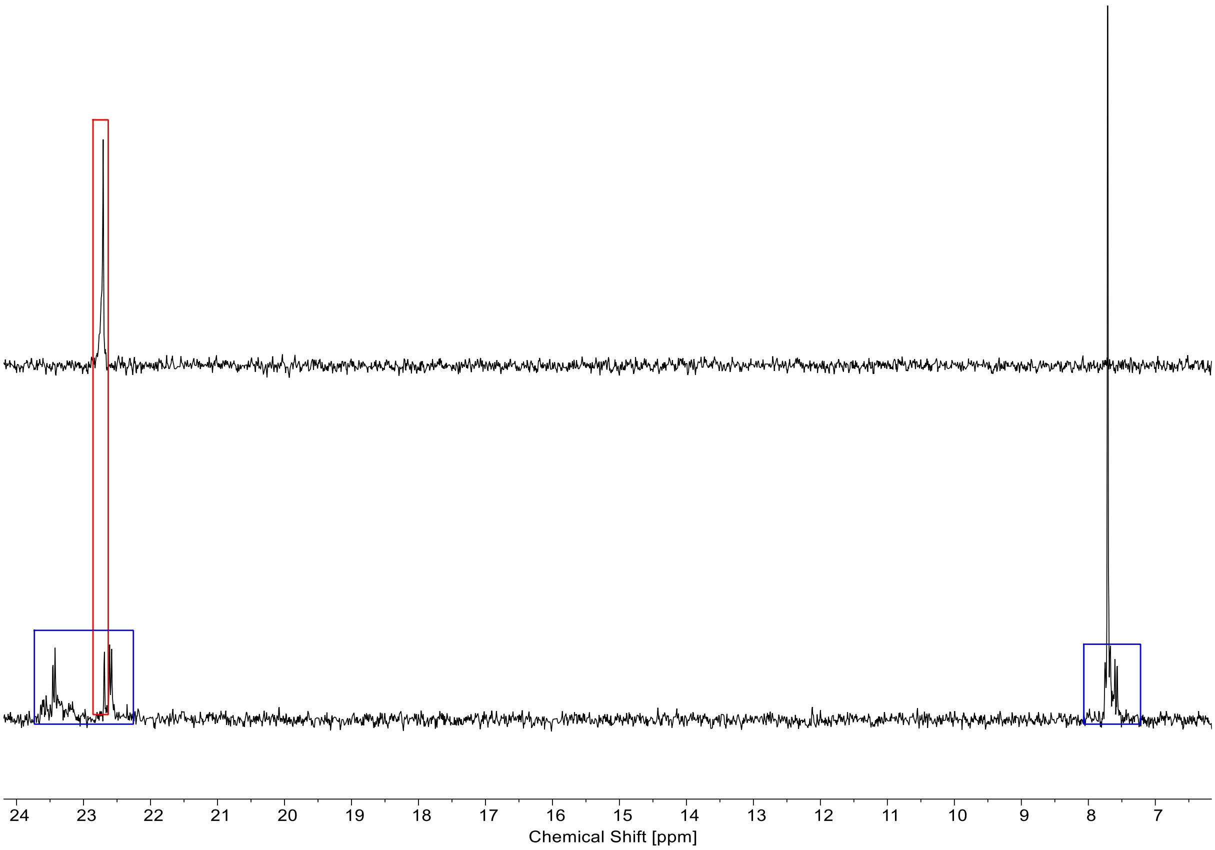

Figure S102. $^{31}P\{^1H\}$ NMR spectra obtained after the quantitative conversion to $[Ph_3PH]^+$ (*o*DFB, 162.0 MHz, DMSO-d_6 capillary) and reference spectrum of $[PPh_4][Br]$ in the presence of $[Li][Al^F]$ (*o*DFB with DMSO-d_6 capillary, 162.0 MHz, top). Color coding: red, $[PPh_4]^+$; blue, phosphonium salts derived from phosphoranylation of *o*DFB and ligand metathesis reactions, with a coupling constant of $^4J_{PF}$ = 4.9 Hz.[9]

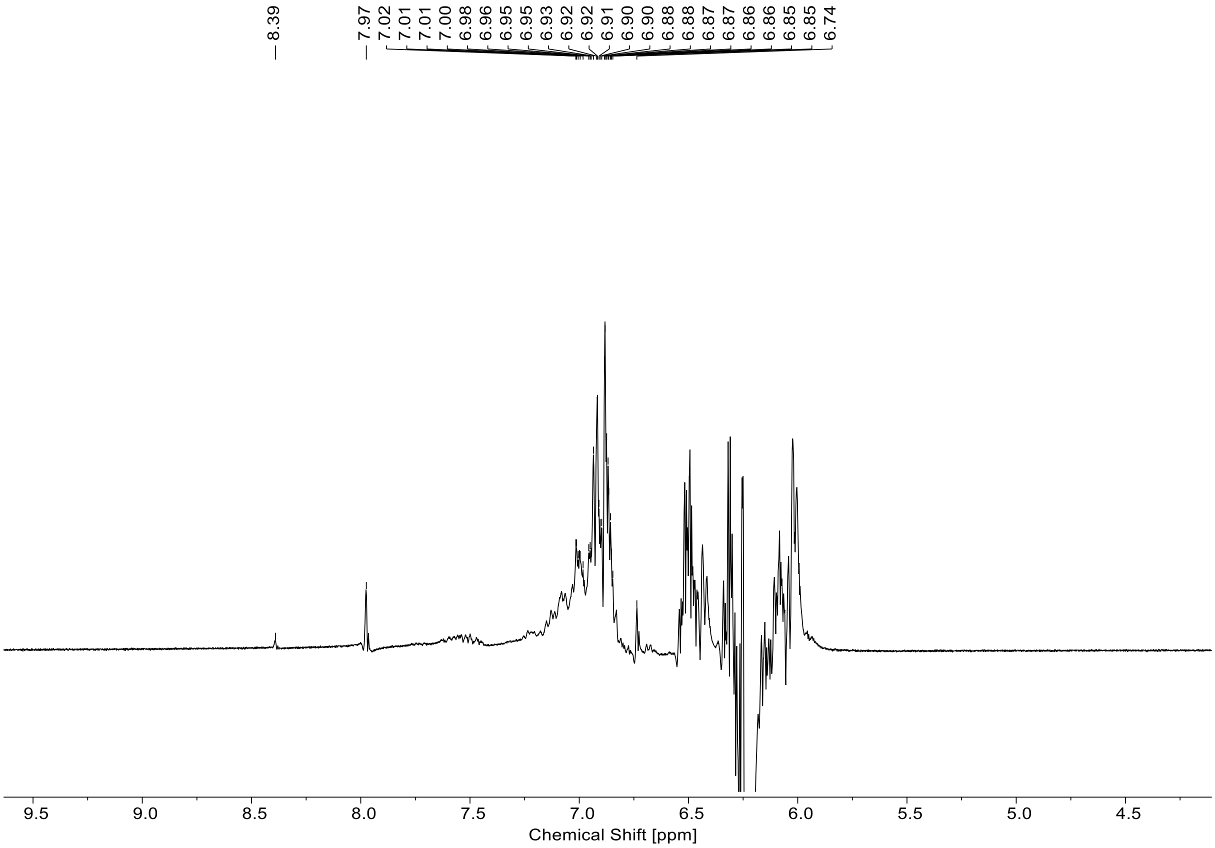

Figure S103. Crude ^{1}H NMR spectrum (*o*DFB with DMSO-d_6 capillary, 400.1 MHz), revealing two doublets at δ =7.36 ppm ($[PH_3PH]^+$) and δ =7.77 ($[Ph_2(C_6H_3F_2)PH]^+$).

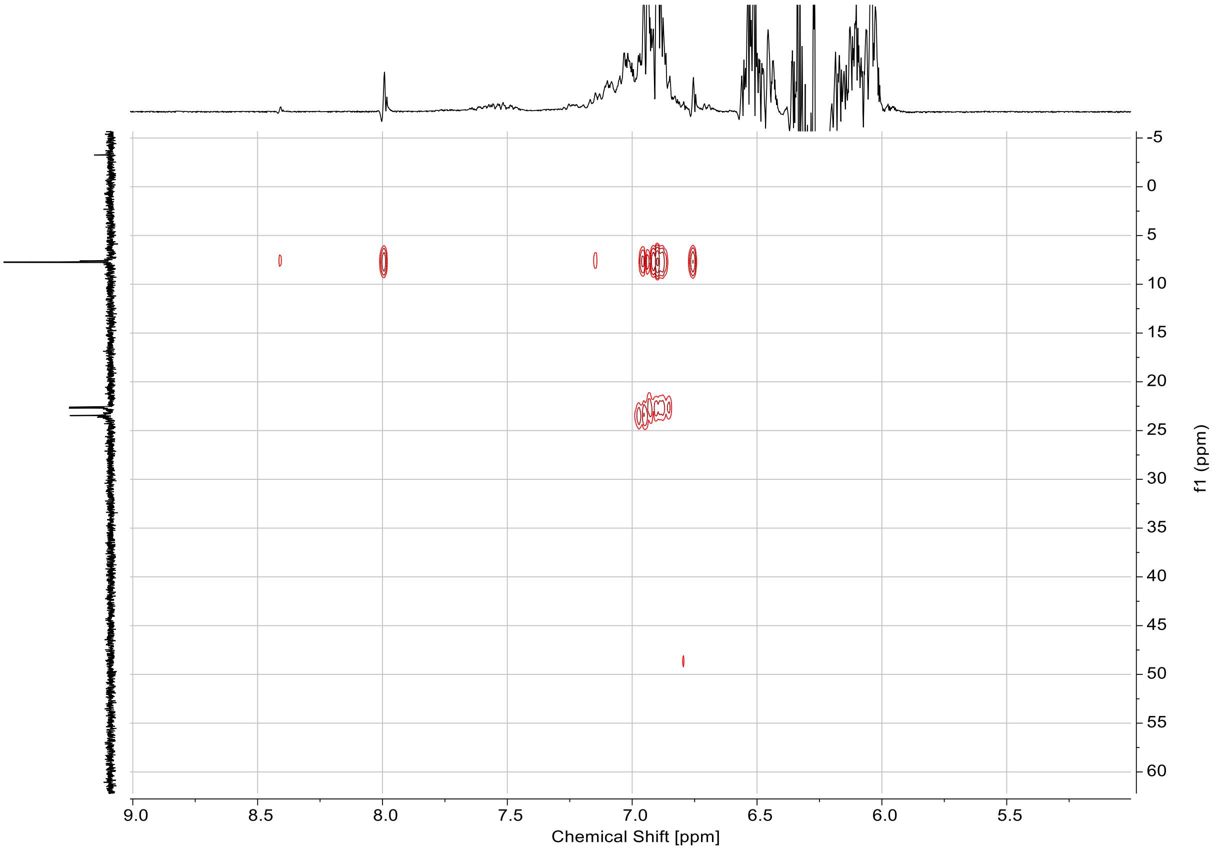

Figure S104. Crude ^{1}H–^{31}P correlated HMBC NMR spectrum (oDFB with DMSO-d_6 capillary, 400.1/162.0 MHz).

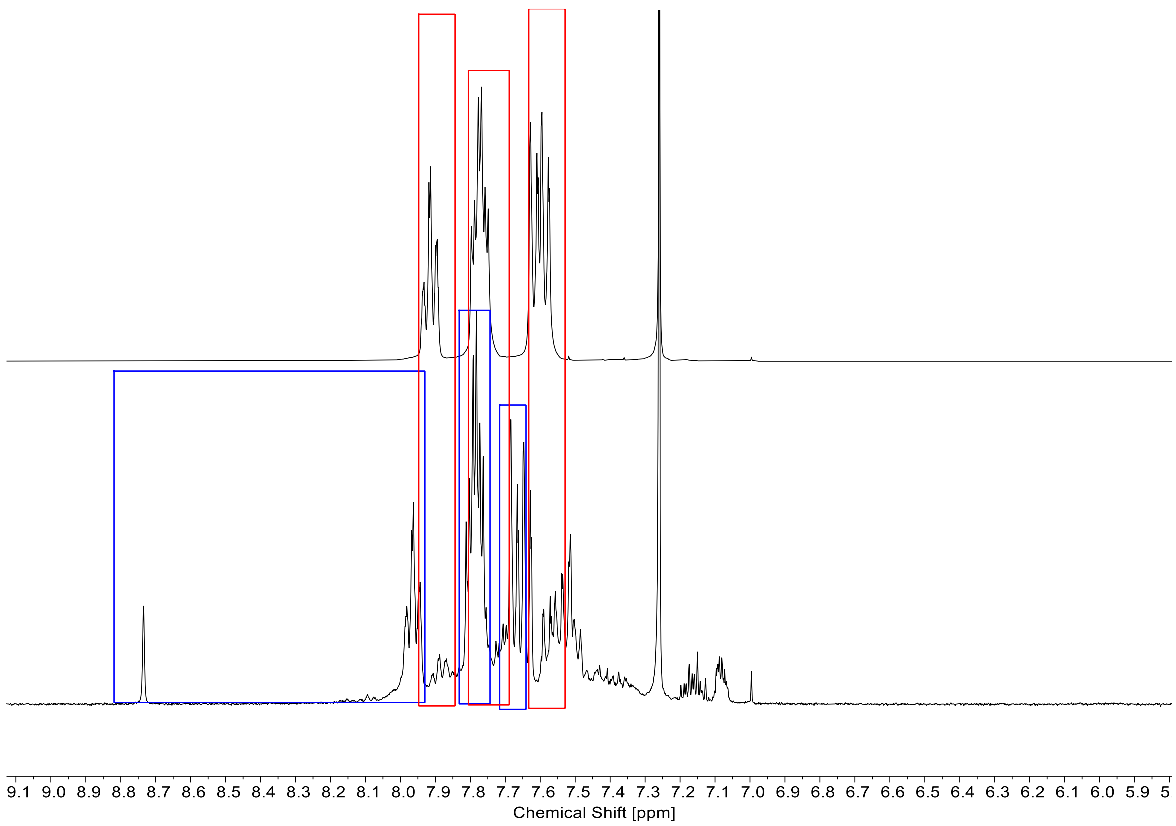

Figure S105. The ^{1}H NMR spectrum after removing the solvent and redissolving in $CDCl_3$ ($CDCl_3$, 400.1 MHz) confirms the formation of $[Ph_3PH]^+$ and tetraphenylphosphonium salts (bottom, blue) as revealed by the stacked reference spectrum of $[PPh_4][Cl]$ in the presence of $[Li][Al^F]$ ($CDCl_3$, 400.1 MHz)(top, red).

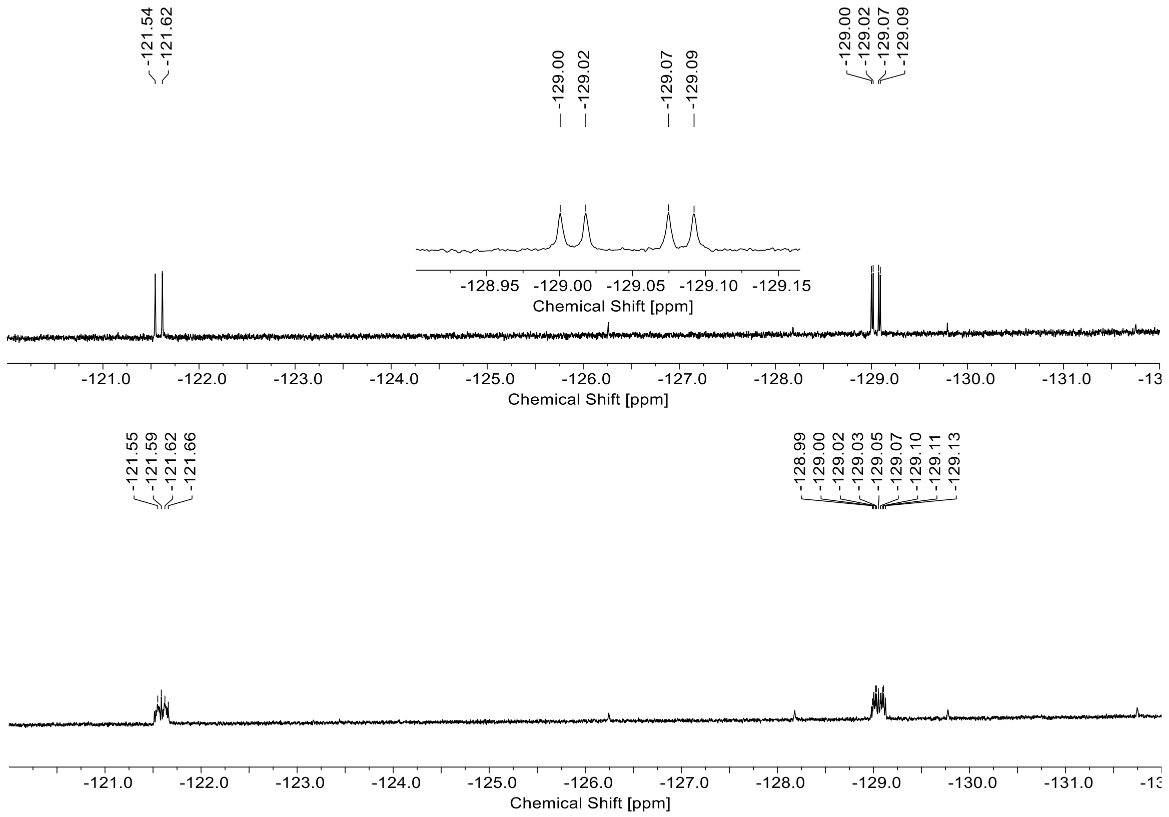

Figure S106. ^{19}F NMR spectrum after full conversion and addition of $CDCl_3$ ($CDCl_3$, 282.4 MHz) (bottom) and corresponding $^{19}F\{^1H\}$ NMR spectrum ($CDCl_3$, 282.4 MHz) (top) showing the fluorine resonances of the phosphonium salt $[Ph_3(C_6H_3F_2)P]^+$.[9]

3.5 Heating 1 in the Presence of DHA

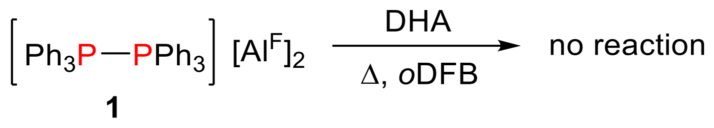

Dihydroanthracene (DHA, 1 mg, 0.006 mmol) was added to a saturated solution of **1** in *o*DFB (0.4 mL). The mixture was heated to reflux in a *J. Young* capped NMR tube for 26 hours. The ^{31}P NMR and ^{31}H NMR spectroscopic analysis revealed the absence of a reaction with significant conversion.

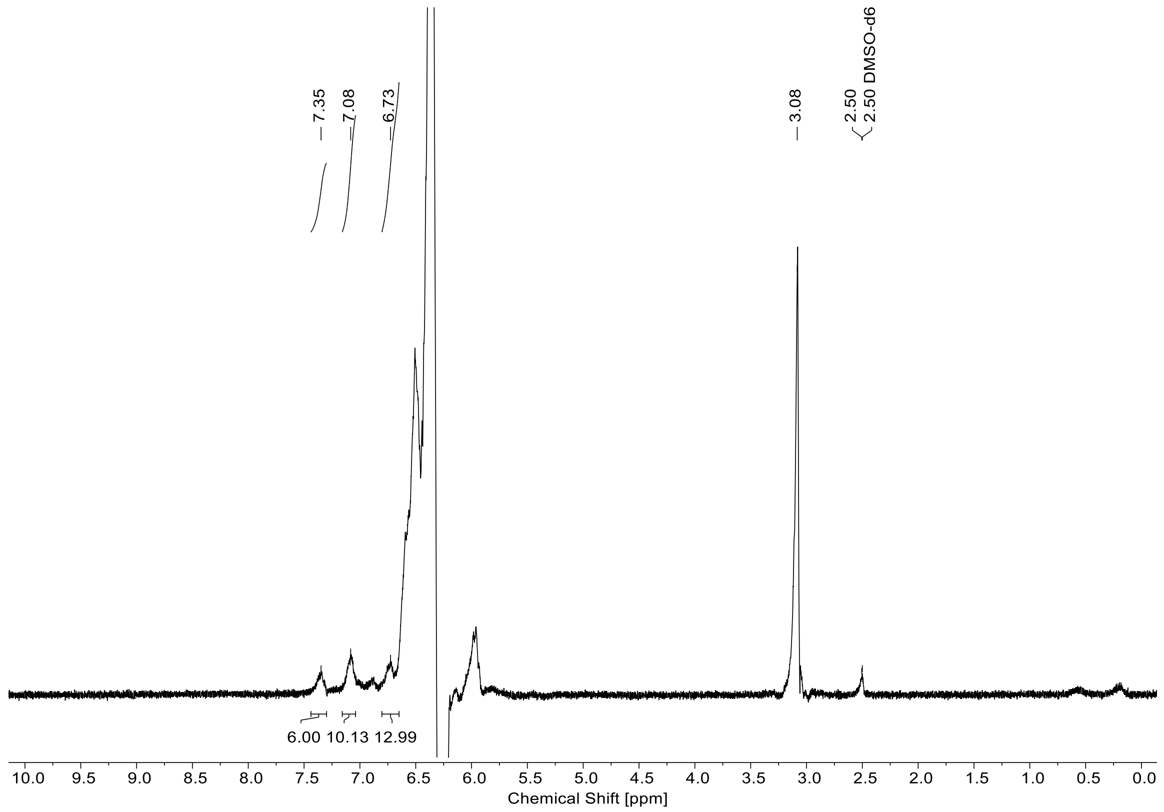

Figure S107. ^{1}H NMR (*o*DFB with DMSO-d_6 capillary, 400.1 MHz) spectrum obtained after refluxing a concentrated solution of **1** in *o*DFB for 26 hours in the presence of DHA.

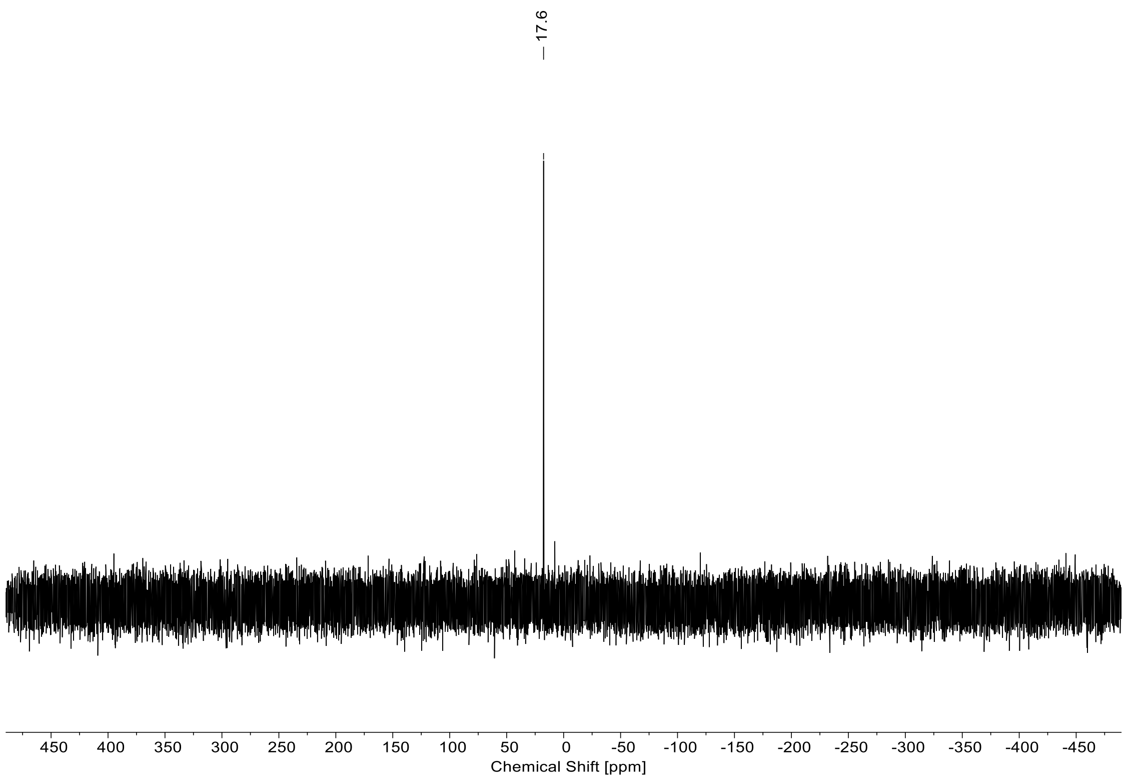

Figure S108. $^{31}P\{H\}$ NMR spectrum (*o*DFB with DMSO-d_6 capillary, 162.0 MHz, D1 = 30 s) obtained after refluxing a concentrated solution of **1** in *o*DFB for 26 hours in the presence of DHA.

4 Electrochemical Studies

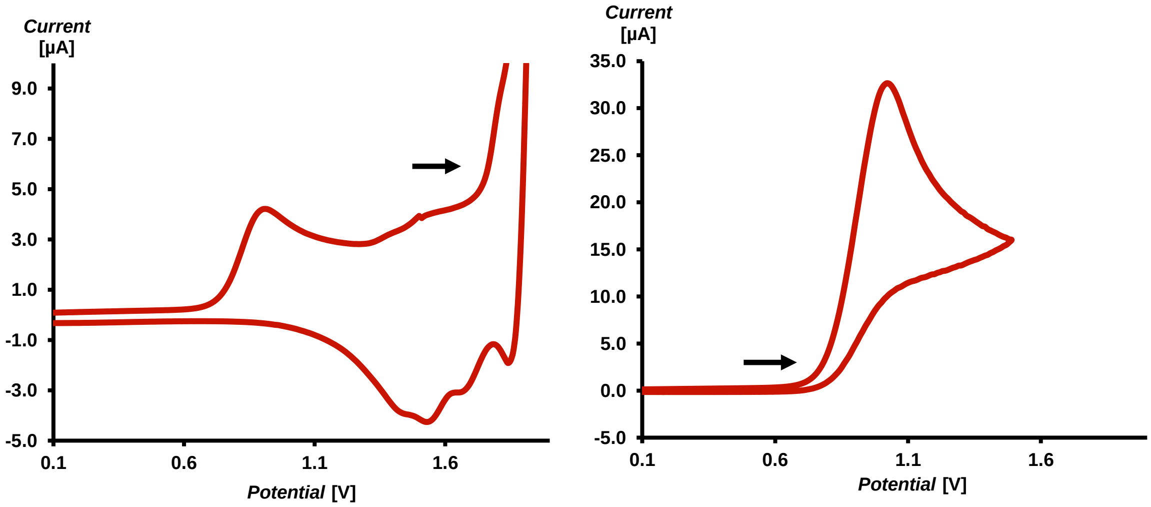

Figure S109. Cyclic voltammogram of **1** ($E_{p/2}$ = 0.81 V) (left) and PPh_3 ($E_{p/2}$ = 0.89 V) (right), referenced *vs.* the Fc/Fc^+ redox couple, using [*n*Bu_4][Al^F] as a supporting electrolyte (0.1 m in *o*DFB) at 100 mV s^{-1} scan rate.

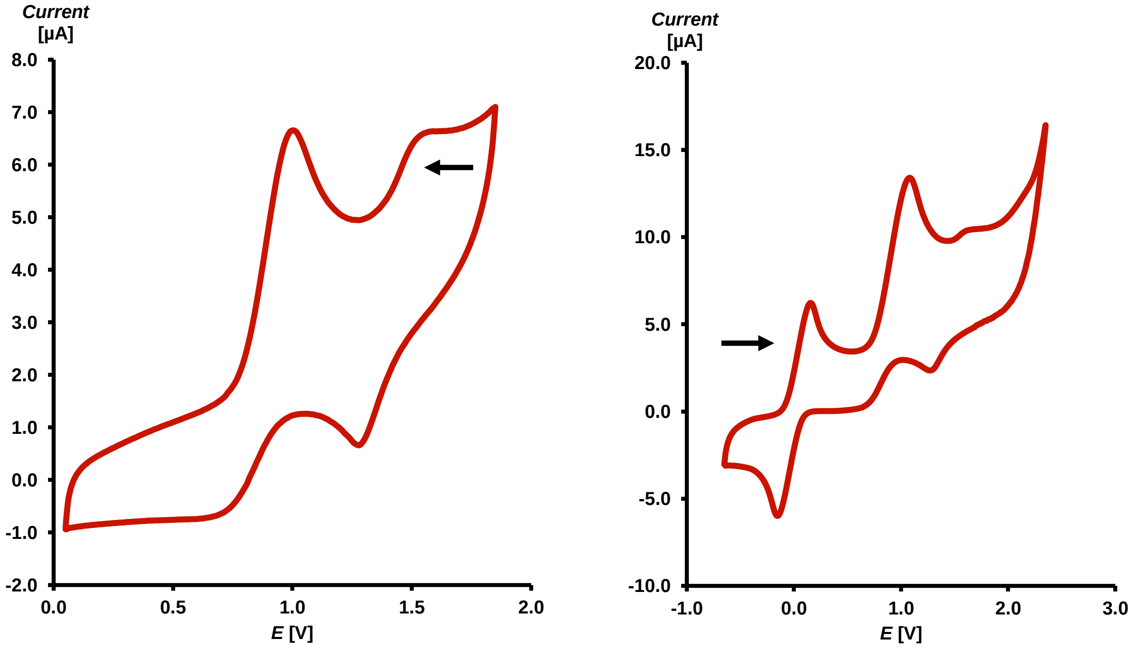

Figure S110. Cyclic voltammogram of **1** ($E_{p/2}$ = 0.88 V; $E_{1/2}$ = 1.44 V) (left) referenced *vs.* the Fc/Fc^+ redox couple, using [*n*Bu_4][Al^F] as a supporting electrolyte (0.1 m in PFB) at 750 mV s^{-1} scan rate (left), and cyclic voltammogram of **1** in the presence of ferrocene (right).

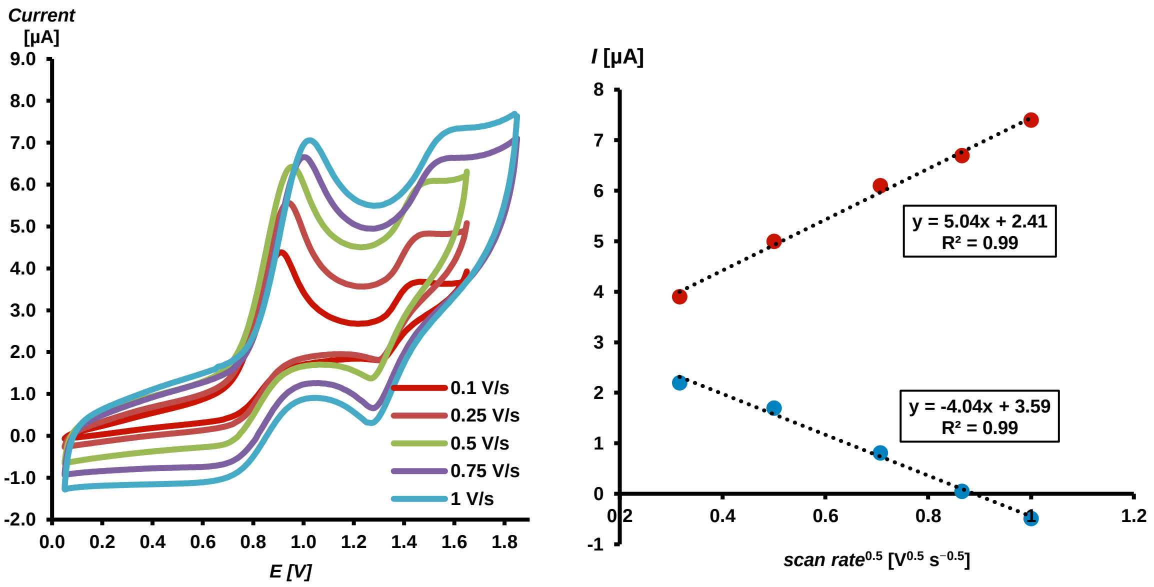

Figure S111. Cyclic voltammogram of **1** (left) referenced *vs.* the Fc/Fc^{+} redox couple, using [*n*Bu_4][Al^F] as a supporting electrolyte (0.1 m in PFB) at different scan rates (left), and corresponding RANDLES–ŠEVČÍK analysis of the second oxidation wave (right), which indicates irreversible (quasi-reversible, respectively) behavior.

5 X-ray structure elucidation and refinement

X-ray Structure Determination: X-ray quality crystals were selected in Fomblin YR-1800 perfluoroether (Alfa Aesar) at ambient temperature. The samples were cooled in all cases, yet to different temperatures (please see Table S1 and special refinement details). The data were collected on a Bruker D8 Venture diffractometer with a microfocus sealed tube and a Photon II detector ($^{\mathbf{Me}}$**5**), Bruker Apex II diffractometer (compounds **1** and **3**) or a Rigaku XtaLAB Synergy-S Diffractometer (compounds **2**, $^{\mathbf{Ph}}$**4**) using monochromated MoK_{α} (λ = 0.71073 Å) radiation. The structures were solved by intrinsic phasing (SHELXT)[18] and refined by full matrix least squares procedures (SHELXL)[19] within the Olex2 or ShelXle platforms.[20] Semi-empirical absorption corrections for **1**, **3**, $^{\mathbf{Ph}}$**4** and $^{\mathbf{Me}}$**5** (multiscan and additional spherical absorption correction) were applied to the diffraction data using the SADABS application within the APEX3 or APEX4 platforms (Bruker Devices).[21] Absorption correction for **2** was done after data reduction using the CrysAlisPro program and implemented SCALE3 ABSPACK suite (RIGAKU device).[22] Molecular structures represent the 50% probability level and have been plotted with the DIAMOND software package.[23] A summary on standard crystallographic parameters as well as the CSD entry numbers within the Cambridge Crystallographic Data Centre (CCDC) is subsequently provided in Table S1.

Special Refinement Details

1: $[Ph_3P–PPh_3][Al^F]_2 \cdot C_6H_2F_4$

All non-H-atoms were located in the electron density maps and refined anisotropically. C-bound H atoms were placed in positions of optimized geometry and treated as riding atoms. The anionic part of the structure is intrinsically disordered caused by tilting and rotation of the CF_3 groups. To fix the disorder, several SADI, SIMU and RIGU restraints were used to stabilize the refinement. All [–O(C(CF_3)$_3$] arms were summarized using the RESI command. The anion was described as a fully disordered entity and a split position for each atom including the central aluminium atom was used. Eight RESI's were used in total. Both [Al^F] entities were allowed to refine freely using the FVAR2 instruction. Finally, occupancies of 0.58 and 0.42 were obtained. A co-crystalline solvent molecule of 1,2,3,4-tetrafluorobenzene (TFB) is disordered across the inversion centre (symmetry generation *via* –x, 1–y and 2–z) and was described with a suitable split position. The occupancies were constrained to reasonable occupancies of 0.666 (F) and 0.333 (H) to account for the 2:1 ratio of fluorine and hydrogen atoms. These occupancies allow the TFB molecule to represent all six possible disorder positions. The overall data/parameter ratio is comparably low due to the difficulties we experienced during data collection

(*crystal cracking at low temperature, disorder of anions*), thus causing an Alert level B of IUCRs "Checkcif": PLAT088_ALERT_3_B). We nevertheless judge the crystallographic data of sufficient quality (*vide infra*) for the presented structural discussion.
Also, we explicitly note that the disorder is only problematic for the anion as well as the solvent molecule. The dication of the structure is nicely ordered. Half of the cation is symmetry generated *via* 1–x, –y and 1–z. We are also aware, that the R_1 and especially the wR_2 (all data) values are high. This can be reasoned with the high amount of disorder and restraints used to fix the refinement. Weak reflections have thus also been omitted and an overall resolution of 0.82 Å was chosen for refinement of the data. Possibly, even more split positions may be needed to fully describe the anionic part of the structure. Obvious (non-merohedral) twinning or modulation can be ruled out after a brief check of the reciprocal space of the structure. Also note its fine internal *R*-value (R_{int}) of 2.18 %. Such problems are known to be intrinsically caused by perfluorinated systems. Another issue which complicated the disorder problematic was the measurement temperature. Cracking of the crystals occurs at temperature lower than 190K. Hence, the measurements could not be performed at lower temperatures. Under these circumstances, we think the structure of $[Ph_3P–PPh_3][Al^F]_2$ is reasonably well described, especially considering the cationic part, which shows no disorder and can therefore also be discussed regarding its metrics.

2: [DMAP@PPh$_3$][Al^F]$_2$

All non-H-atoms were located in the electron density maps and refined anisotropically. C-bound H atoms were placed in positions of optimized geometry and treated as riding atoms. The CF_3 groups of the two crystallographically independent $[Al^F]$ anions show disorder through rotation and tilting of one $[–O(C(CF_3)_3]$ unit each. To fix disorder, a free variable (FVAR) in combination with SADI, SIMU and RIGU restraints were employed. According to FVAR2 and FVAR3, respectively, the final occupancies 0.68 and 0.32 (FVAR2) or 0.65 and 0.35 (FVAR3) are obtained.

3: [PPh$_3$Cl]$_2$[Sb$_2$Cl$_8$]

The structure contains four crystallographically independent $[Ph_3PCl]^+$ cations and two $[Sb_2Cl_8]^{2–}$ anions. No special refinement details need to be noted.

^{Ph}4: [Ph$_3$P(NC-Ph)PPh$_3$][Al^F]$_2$

All non-H-atoms were located in the electron density maps and refined anisotropically. C-bound H atoms were placed in positions of optimized geometry and treated as riding atoms. The CF_3 groups of one of the two crystallographically independent $[Al^F]$ anions

show disorder through rotation and tilting of one [–O(C(CF_3)$_3$] unit. To fix disorder, a free variable (FVAR) in combination with SADI, SIMU, FLAT and RIGU restraints were employed. According to FVAR3, final occupancies of 0.75 and 0.25 are obtained. The dicationic part of the structure shows disorder across the CN moiety, which affects three phenyl rings bound to phosphorous as well as the phenyl ring of benzonitrile (see .cif for details). A split position is described for $[Ph_3P(NC\text{-}Ph)PPh_3]^{2+}$ accordingly (PART1 and 2). Both parts have been refined freely using the FVAR2 instruction, and final occupancies of 0.61 and 0.39 are obtained. SADI restraints were included for both orientations to account for similar atomic distances.

^{Me}5: [$Ph_3P(CD_2$-C=ND)PPh_3][Al^F]$_2$·CD_3CN

All non-H/D-atoms were located in the electron density maps and refined anisotropically. C-bound H or D atoms were placed in positions of optimized geometry and treated as riding atoms. Deuterium atoms have been attached to the acetonitrile fragments due to use of acetonitrile-d_3 in the synthesis of **^{Me}5**. The enamine N–D atom distance was fixed with a DFIX 0.88 command. The anionic part of the structure is intrinsically disordered caused by tilting and rotation of the CF_3 groups. To fix the disorder, several SADI, SIMU and DELU restraints were used to stabilize the refinement. One of the two anions was described as a fully disordered entity and a split position for each atom including the central aluminium atom was used. The other anion is more well-behaved and only shows the disorder of one [–O(C(CF_3)$_3$] arm. All [–O(C(CF_3)$_3$] arms were summarized using RESI commands. 13 RESI's have been employed in total due to the presence of formally 13 different [–O(C(CF_3)$_3$] moieties. The fully disordered [Al^F] entity was allowed to refine freely using the FVAR3 instruction. Occupancies of 0.666 and 0.333 were obtained. The disordered perfluoroether in the second [Al^F] entity was treated as FVAR2 and final occupancies of 0.71 and 0.29 were obtained, respectively. We are aware that the overall data/parameter ratio is comparably low, yet it is still in a reasonable window. We are also aware, that the R_1 and especially the wR_2 (all data) values are comparatively high. This can be reasoned with the high degree of disorder and restraints used to fix the refinement. Weak reflections have thus also been omitted and an overall resolution of 0.84Å was chosen for refinement of the data. Possibly, even more split positions may be needed to fully describe the anionic part of the structure. Such problems are known to be intrinsically caused by perfluorinated systems. Overall, we think that the structure of [$Ph_3P(D_2C$–C=ND)PPh_3][Al^F]$_2$ is reasonably well described, especially considering the cationic part, which shows no disorder.

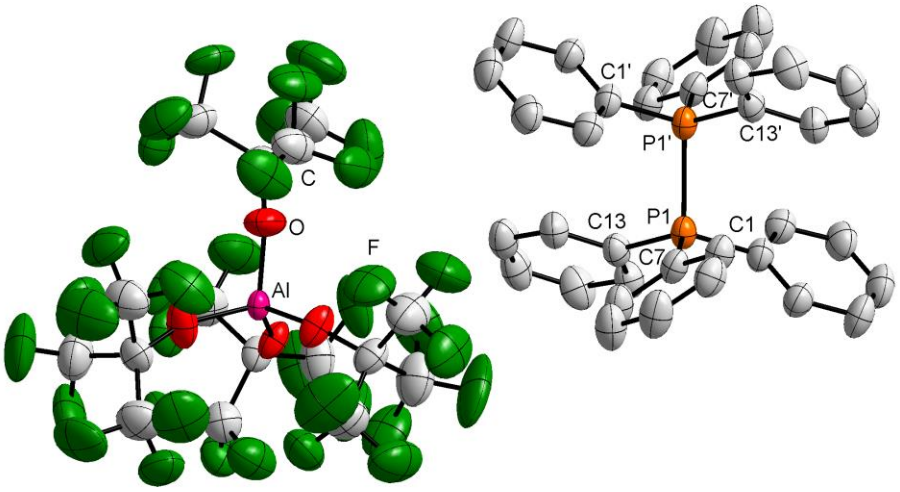

Figure S112. Molecular structure representation of **1**. A co-crystalline solvent molecule (1,2,3,4-TFB), a second, symmetry generated [Al^F]$^-$ anion as well as disordered parts are omitted for clarity. Atoms marked with " ' " are symmetry generated using 1-x, -y, 1-z. Selected atom distances [Å] and angles [°]: P1-P1′ 2.262(2), P1-C1 1.793(4), P1-C7 1.785(4), P1-C13 1.793(4), C1-P1-P1′ 107.0(2), C7-P1-P1′ 106.6(1), C13-P1-P1′ 105.7(1), C1-P1-C13 112.2(2), C1-P1-C7 111.4(2), C7-P1-C13 113.2(2).

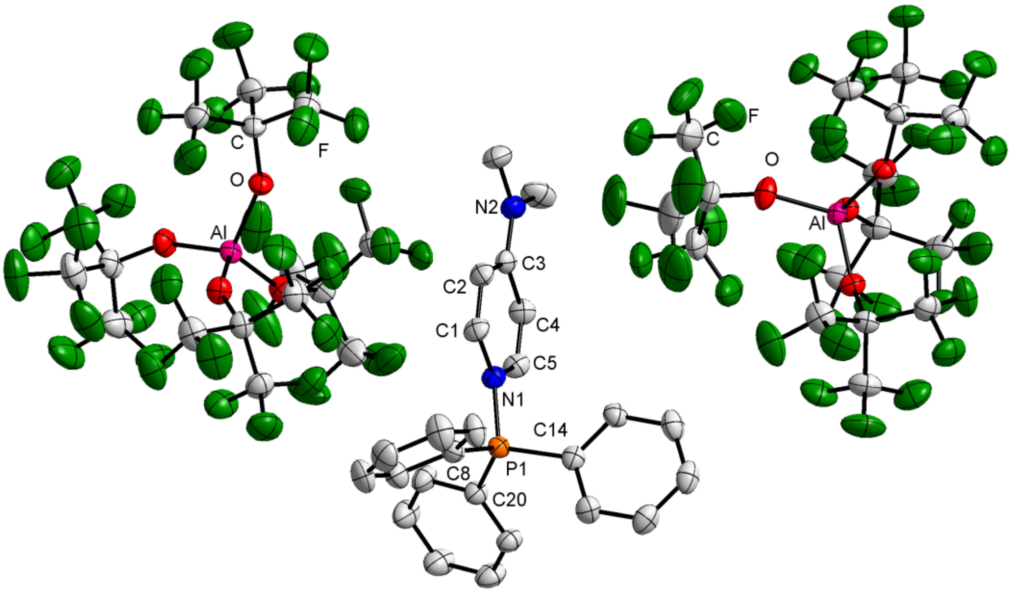

Figure S113. Molecular structure representation of **2**. Disordered parts of the anions are omitted for clarity. Selected atom distances [Å] and angles [°]: P1-N1 1.719(2), P1-C8 1.778(3), P1-C14 1.780(3), P1-C20 1.778(3), N1-C1 1.383(4), N1-C5 1.387(4), C1-C2 1.339(4), C2-C3 1.422(4), C3-C4 1.432(4), C4-C5 1.328(4), C3-N2 1.320(4). C8-P1-N1 104.0(1), C14-P1-N1 106.7(1), C20-P1-N1 104.0(1), C8-P1-C14 109.9(1), C8-P1-C20 113.9(1), C14-P1-C20 113.5(1), N2-C3-C2 122.2(2), N2-C3-C4 122.5(2).

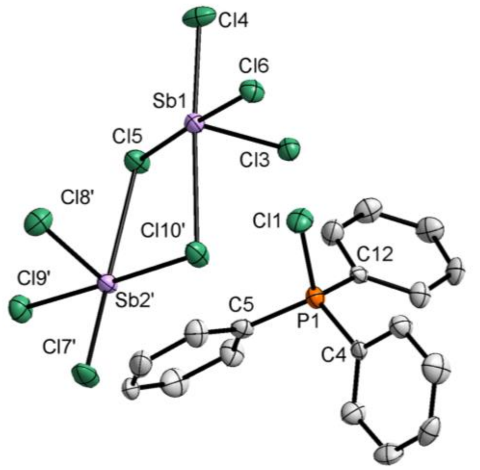

Figure S114. Molecular structure representation of **3**. The structure is constrained to one of four $[PPh_3Cl]^+$ cations and the anion is shown as a properly connected set of atoms including a symmetry generated $[SbCl_4]^-$ fragment. Atoms marked with " ' " are symmetry generated using 1-x, 1-y, 1-z. Selected atom distances [Å] and angles [°]: P1–Cl1 1.995(2); P1–C4 1.780(5); P1-C5 1.757(7); P1-C12 1.776(7); Sb1-Cl3 2.369(2); Sb1-Cl4 2.420(2); Sb1-Cl5 2.775(2); Sb1-Cl6 2.492(2); Sb2′-Cl7′ 2.394(2); Sb2′-Cl8′ 2.381(2); Sb2′-Cl9′ 2.488(2); Sb2′-Cl5 2.986(2); Sb2′-Cl10′ 2.776(2); Cl1-P1-C4, 107.64(2); Cl1-P1-C5 107.2(2); Cl1-P1-C12 107.1(2).

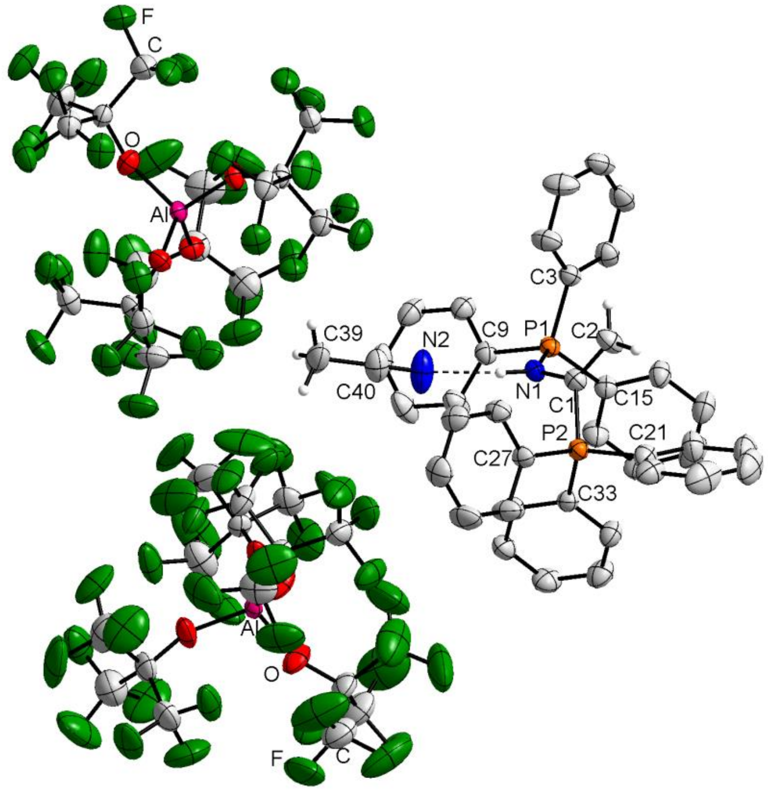

Figure S115. Molecular structure representation of ^{Me}**5**. Selected atom distances [Å] and angles [°]: P1-N1 1.650(4), P1-C3 1.783(4), P1-C9 1.787(5), P1-C15 1.788(5), N1-C1 1.424(6), C1-C2 1.324(7), P2-C1 1.817(4), P2-C21 1.790(5), P2-C27 1.793(5), P2-C33 1.794(5), C39-C40 1.457(9), C40-N2 1.119(9), N1···N2 2.844(6), P1-N1-C1 123.6(3), N1-C1-P2 116.3(3), N1-C1-C2 123.9(4), C39-C40-N2 179.4(7), C3-P1-C9 108.8(2), C3-P1-C15 108.8(2), C9-P1-C15 114.8(2), C3-P1-N1 112.4(2), C9-P1-N1 103.5(2), C15-P1-N1 108.3(2), P2-C1-N1 116.3(3), P2-C1-C2 119.7(3), C21-P2-C27 111.7(2), C21-P2-C33 111.7(2), C27-P2-C33 107.4(2), C1-P2-C27 108.4(2), C1-P2-C21 106.6(2), C21-P2-C27 111.7(2), C1-P2-C27 108.4(2).

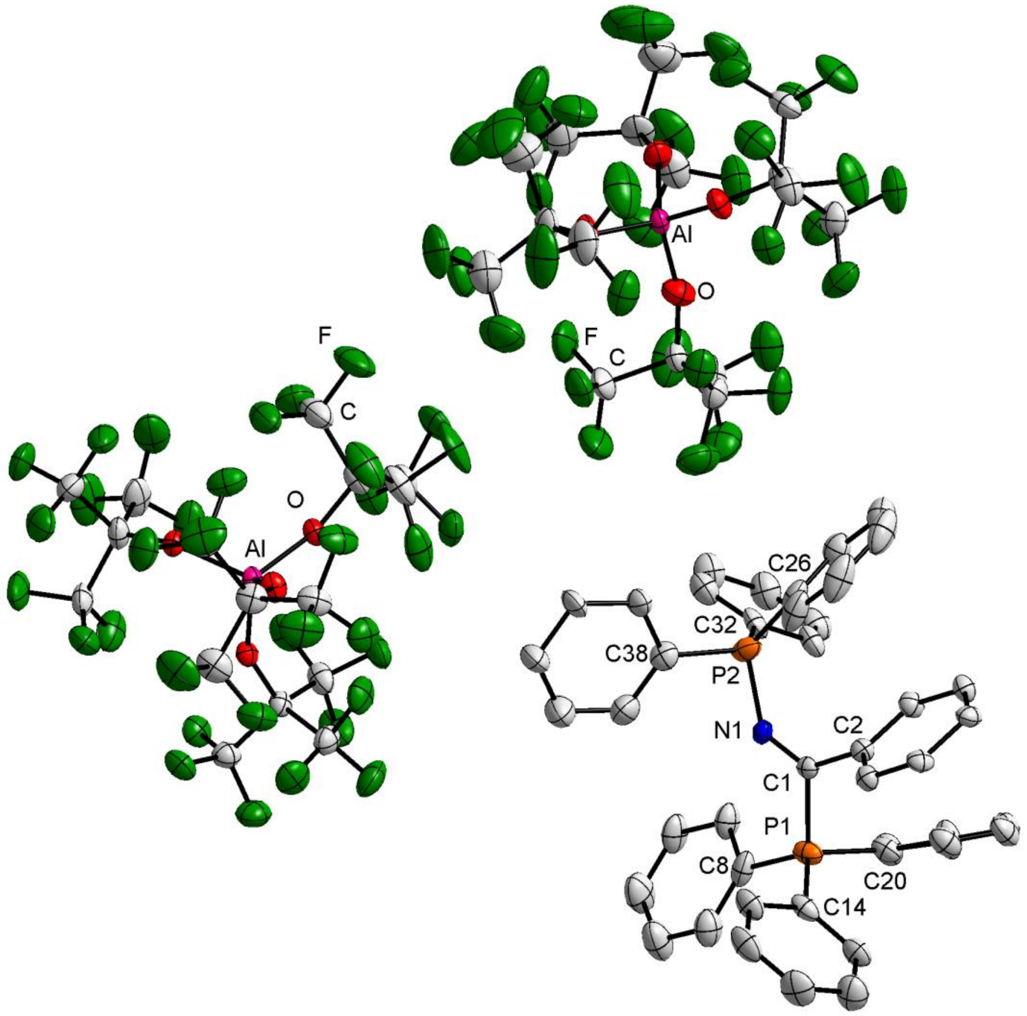

Figure S116. Molecular structure representation of **^{Ph}4**. Selected atom distances [Å] and angles [°]: P1-C1 1.890(8), P1-C8 1.764(15), P1-C14 1.771(8), P1-C15 1.788(7), N1-C1 1.251(11), C1-C2 1.494(13), P2-N1 1.716(9), P2-C26 1.784(5), P2-C32 1.785(28), P2-C33 1.783(18), P1-C1-N1 110.8(7), P1-C1-C2 118.7(7), C1-P1-C8 115.9(7), C1-P1-C14 103.2(3), C1-P1-C20 99.9(4), P2-N1-C1 128.6(7), N1-C1-C2 129.9(9), N1-P2-C26 124.8(3), N1-P2-C32 95.9(10), N1-P2-C38 99.3(9). Note: All atom distances need to be interpreted carefully. Restraints were employed (see special refinement details and .cif file).

Table S1. Crystallographic details for compounds **1**, **2**, **3** and **^{Me}5**.

**Compound**	**1**	**2**	**3**	**^{Me}5**
Empirical formula	$C_{74}H_{32}O_8F_{76}Al_2P_2$ *	$C_{57}H_{25}N_2PAl_2O_8F_{72}$	$C_{36}H_{30}Cl_{10}P_2Sb_2$	$C_{72}H_{30}D_6N_2O_8F_{72}Al_2P_2$
Formula weight	1304.45	2318.72	1122.54	2546.96
Temperature/K	190(2)	130(2)	150(2)	133(2)
Crystal system	triclinic	monoclinic	triclinic	monoclinic
Space group	*P*–1	*P*2₁/n	*P*–1	*P*2₁/*c*
a/Å	14.1232(7)	15.3829(4)	12.1169(3)	25.2854(12)
b/Å	14.1360(7)	20.8474(7)	18.4773(5)	16.4210(8)
c/Å	14.3817(7)	25.1505(8)	19.9902(5)	22.8826(11)
α/°	65.742(2)	90	97.0870(10)	90
β/°	66.514(2)	92.389(3)	107.2470(10)	105.279(2)
γ/°	66.224(2)	90	90.4960(10)	90
Volume/Å^3	2300.3(2)	8058.6(4)	4236.82(19)	9165.3(8)
Z	2	4	4	4
ρ_{calc}g/cm^3	1.883	1.911	1.760	1.846
μ/mm^{-1}	0.275	0.275	2.008	0.268
F(000)	1280.0	4536.0	2192.0	5000.0
Crystal size/mm^3	0.3 × 0.3 × 0.2	0.18 × 0.16 × 0.02	0.14 × 0.11 × 0.1	0.24 × 0.2 × 0.12
Radiation	MoKα (λ = 0.71073)	MoKα (λ = 0.71073)	MoKα (λ = 0.71073)	MoKα (λ = 0.71073)
2*Θ* range for data collection/°	3.234 to 51.362	4.542 to 54.29	2.152 to 50.804	4.16 to 50.054
Index ranges	-17 ≤ h ≤ 16, -17 ≤ k ≤ 17, -17 ≤ l ≤ 17	-19 ≤ h ≤ 19, -24 ≤ k ≤ 26, -32 ≤ l ≤ 31	-13 ≤ h ≤ 14, -22 ≤ k ≤ 22, -24 ≤ l ≤ 24	-30 ≤ h ≤ 30, -19 ≤ k ≤ 19, -26 ≤ l ≤ 27
Reflections collected	32432	59216	60541	210778
Independent reflections	8731 [R_{int} = 0.0218, R_{sigma} = 0.0241]	17777 [R_{int} = 0.0561, R_{sigma} = 0.0558]	15581 [R_{int} = 0.0779, R_{sigma} = 0.0922]	16173 [R_{int} = 0.0508, R_{sigma} = 0.0198]
Data/restraints/parameters	8731/7258/1253 **	17777/9378/1529 **	15581/0/901	16173/16376/2069 **
Goodness-of-fit on F^2	1.492	1.011	1.011	1.038
Final R indexes [*I*>=2σ (I)]	R_1 = 0.0990, wR_2 = 0.3180	R_1 = 0.0551, wR_2 = 0.1280	R_1 = 0.0468, wR_2 = 0.0836	R_1 = 0.0946, wR_2 = 0.2600
Final R indexes [all data]	R_1 = 0.1157, wR_2 = 0.3398	R_1 = 0.0916, wR_2 = 0.1446	R_1 = 0.0975, wR_2 = 0.0999	R_1 = 0.1120, wR_2 = 0.2812
Largest diff. peak/hole /e Å^{-3}	1.01/-0.81	0.52/-0.33	1.57/-0.79	1.51/-1.18
Absolute structure parameter	–	–	–	–
CCDC #	2411876	2411877	2411879	2411878

* Sum formula in CIF according to asymmetric unit.

**High number of restraints necessary to fix disorder of the $[Al^F]^-$ anions.

Table S2. Crystallographic details for compound **^{Ph}4**.

**Compound**	**^{Ph}4**
Empirical formula	$C_{75}H_{35}O_8F_{72}Al_2P_2N$
Formula weight	2561.94
Temperature/K	133(2)
Crystal system	Monoclinic
Space group	$P2_1$
a/Å	15.8174(6)
b/Å	20.9543(4)
c/Å	15.8763(6)
α/°	90
β/°	119.004(5)
γ/°	90
Volume/Å^3	4602.1(3)
Z	2
ρ_{calc}g/cm^3	1.849
μ/mm^{-1}	0.267
F(000)	2520.0
Crystal size/mm^3	0.16 × 0.14 × 0.01
Radiation	MoKα (λ = 0.71073)
2*Θ* range for data collection/°	4.87 to 54.252
Index ranges	-20 ≤ h ≤ 20, -26 ≤ k ≤ 25, -20 ≤ l ≤ 20
Reflections collected	71417
Independent reflections	19928 [R_{int} = 0.0678, R_{sigma} = 0.0616]
Data/restraints/parameters	19928/8692/1794*
Goodness-of-fit on F^2	1.028
Final R indexes [*I*>=2σ (I)]	R_1 = 0.0566, wR_2 = 0.1212
Final R indexes [all data]	R_1 = 0.0812, wR_2 = 0.1310
Largest diff. peak/hole /e Å^{-3}	0.88/-0.34
Absolute structure parameter	0.04(6)**
CCDC #	2430879

* High number of restraints necessary to fix disorder of the $[Al^F]^-$ anion as well as dicationic part of the structure.

** Flack parameter indicates no centre of inversion and does not indicate inversion twinning.

6 Computational Details

The geometry optimizations were performed with Gaussian 16.C01.[24] All calculated structures were verified as true minima by the absence of imaginary eigenvalues in the harmonic vibrational frequency analysis. The geometry optimizations were performed using the BP86,[25] B3LYP,[26] PBE0,[27] M06-2X[28] and ωB97XD[29] density functional approximations in combination with def2-SVP basis set. Grimme's D3 dispersion correction[30] with Becke-Johnson damping function (BJ) was applied for BP86 and PBE0. Single point calculations were carried out at the PBE0-D3BJ/def2-TZVPP//PBE0-D3(BJ)/def2-SVP level of theory. For reaction mechanisms, implicit solvation correction (fluorobenzene or acetonitrile) was applied with the CPCM model[31]. The energies of all compounds were standard-state corrected by 8 kJ mol^{-1} to account for the transfer from the gas phase to the solution, and furthermore entropy-corrected by 14 kJ mol^{-1} (fluorobenzene) and 16.5 kJ mol^{-1} (acetonitrile) to account for the solvent suppression on the rotational and translational freedoms of the solutes.[32] CAS calculations were performed with PySCF[33] and the def2-TZVP basis set, the EDA-NOCV calculations were carried out with ADF 2019.103.[34] All-electron basis sets of triple-ζ quality, augmented with two set of polarization functions (TZ2P), were used for all elements.[35] The charge transfer (Δq) was obtained using Hirshfeld atom definitions.[36]

The Effective Oxidation State (EOS)[37] analysis was conducted using the Topological Fuzzy Voronoi Cells (TFVC) atomic definition,[38] and 40 × 146 atomic grid for the numerical integrations.

The DLPNO-CCSD(T) calculations were carried out with Orca v.6.0[39] in combination with the correlated-consistent cc-pVQZ basis set.[40]

Table S3. Structural Benchmark – PBE0 affords the best fit with the structural parameters found by sc-XRD analysis.

**Method**	**P–P [Å]**
sc-XRD	2.262(2)
BP86-D3(BJ)/def2-SVP	2.286
B3LYP-D3(BJ)/def2-SVP	2.284
PBE0-D3(BJ)/def2-SVP	2.269
M062X/def2-SVP	2.282
ωB97XD/def2-SVP	2.276

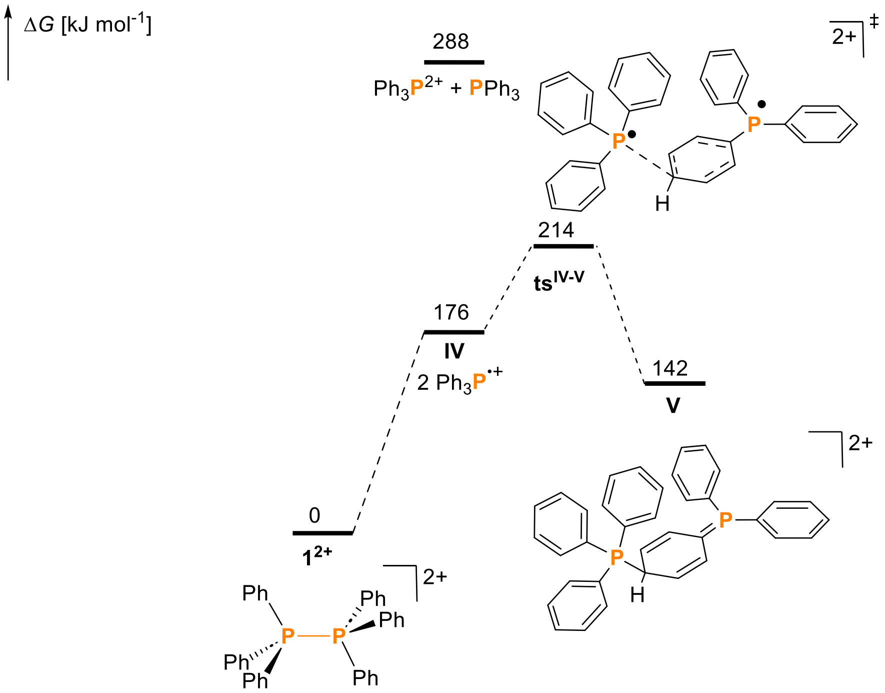

Figure S117. Gibbs Free Energy profile for the isomerization of $\mathbf{1}^{2+}$ to **V** according to the PBE0-D3(CPCM=PhF)/def2-TZVPP//PBE0-D3/def2-SVP level of theory.

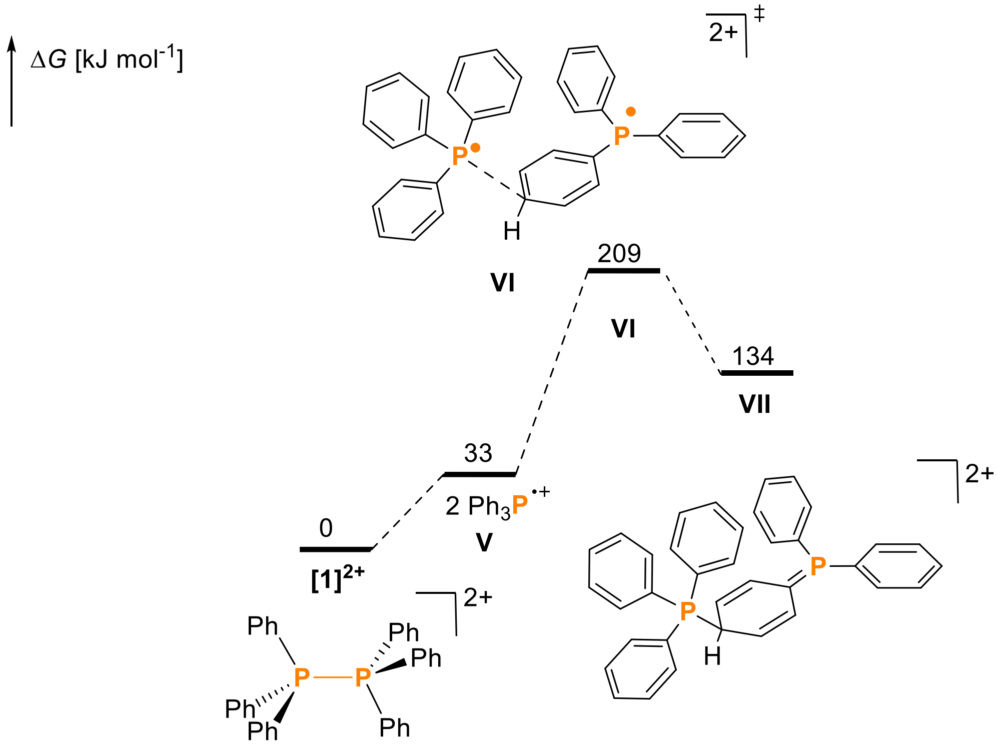

Figure S118. Gas phase Gibbs Free Energy profile for the isomerization of $\mathbf{1}^{2+}$ to **VII** according to the PBE0-D3/def2-TZVPP//PBE0-D3/def2-SVP level of theory.

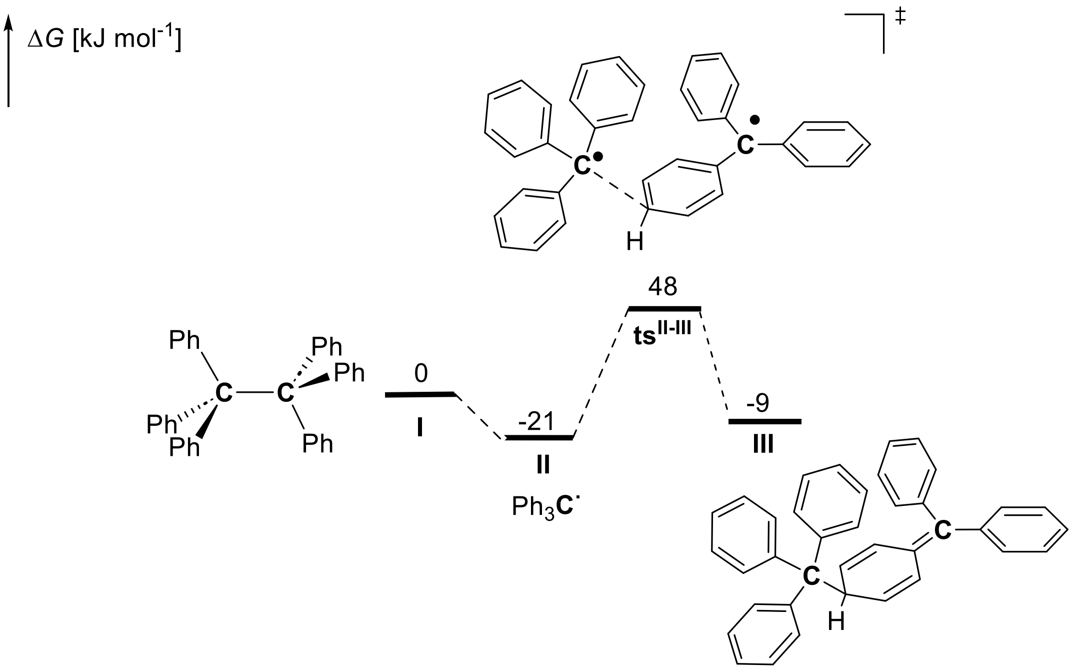

Figure S119. Gibbs Free Energy profile for the isomerization of **I** to **III** according to the PBE0-D3(CPCM=PhF)/def2-TZVPP//PBE0-D3/def2-SVP level of theory.

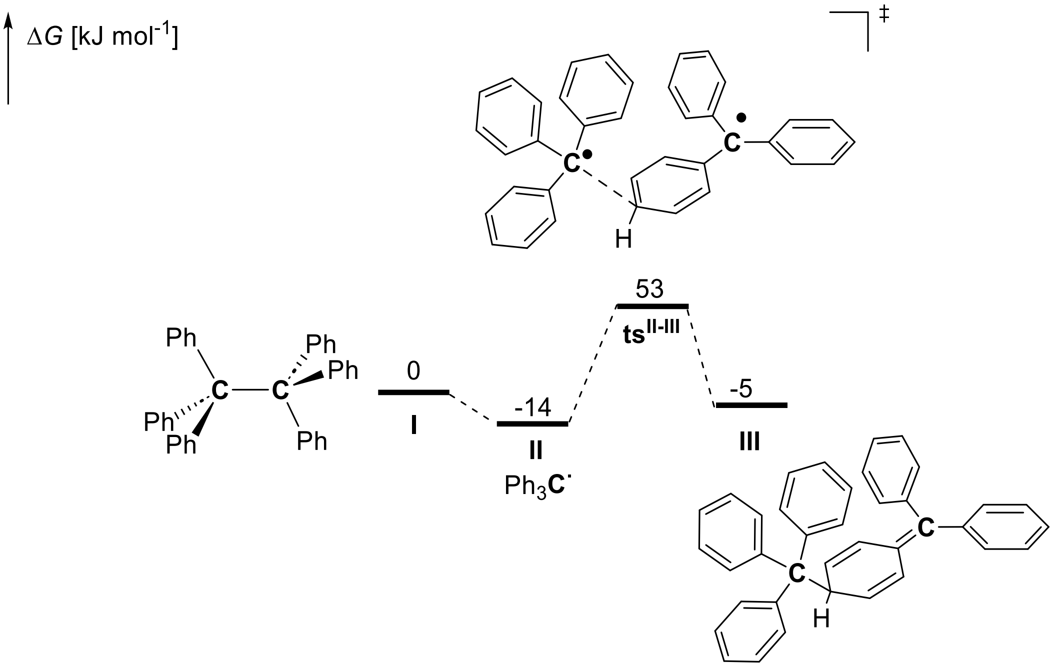

Figure S120. Gas phase Gibbs Free Energy profile for the isomerization of **I** to **III** according to the PBE0-D3/def2-TZVPP//PBE0-D3/def2-SVP level of theory.

Table S4. Thermal correction to free energy (TCG) at the PBE0-D3(BJ)/def2-SVP level of theory, and energies and <S^2> values according to the PBE0-D3(CPCM=PhF)/def2-TZVPP//PBE0-D3(BJ)/def2-SVP level of theory. Energies and <S^2> values according to the PBE0-D3(CPCM=PhF)/def2-TZVPP//PBE0-D3/def2-SVP level of theory. [a] Standard-State and Entropy corrected Gibbs energies.

	TCG [Eh]	*E* [Eh]	*G* [Eh]	*G*^{corr}[kJ·mol^{-1}][a]	<S^2>
**[1]$^{2+}$**	0.491099	–2070.95731	–2070.46621	–5430411	0.00
**IV ([PPh$_3$]$^{+\cdot}$)**	0.230566	–1035.43432	–1035.20376	–2715196	0.77
**[PPh$_3$]^0**	0.230031	–1035.64339	–1035.41336	–2715873	0.00
**[PPh$_3$]$^{2+}$**	0.232333	–1035.1839	–1034.95157	–2714662	0.00
**ts^{IV-V}**	0.483123	–2070.86789	–2070.38477	–5430202	0.80
**V**	0.486039	–2070.89806	–2070.41202	–5430278	0.00
**I**	0.506434	–1464.92596	–1464.41953	–3841135	0.00
**II (monomer)**	0.236932	–732.454879	–732.217947	–1920582	0.79
**ts^{II-III}**	0.498713	–1464.89979	–1464.40108	–3841082	0.25
**III**	0.502623	–1464.92574	–1464.42312	–3841141	0.00

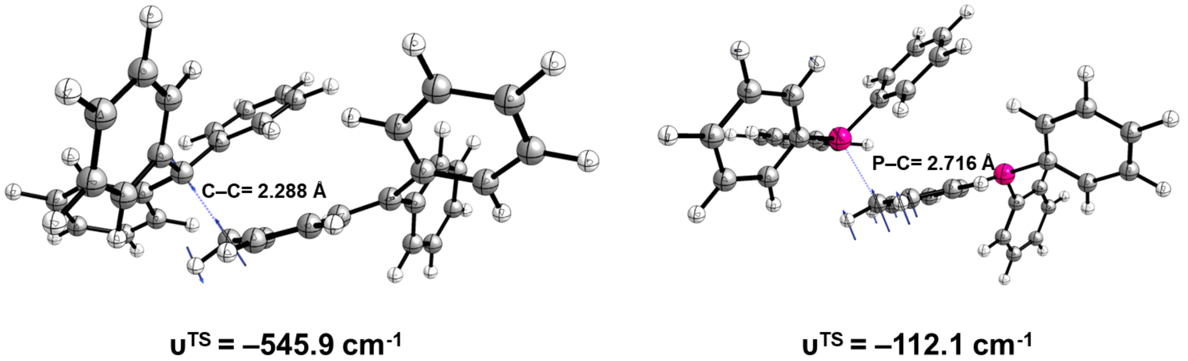

Figure S121. Transition state geometries of **ts^{II-III}** (right) and **ts^{IV-V}** (left) with their corresponding imaginary frequencies according to PBE0-D3(BJ)/def2-SVP level of theory.

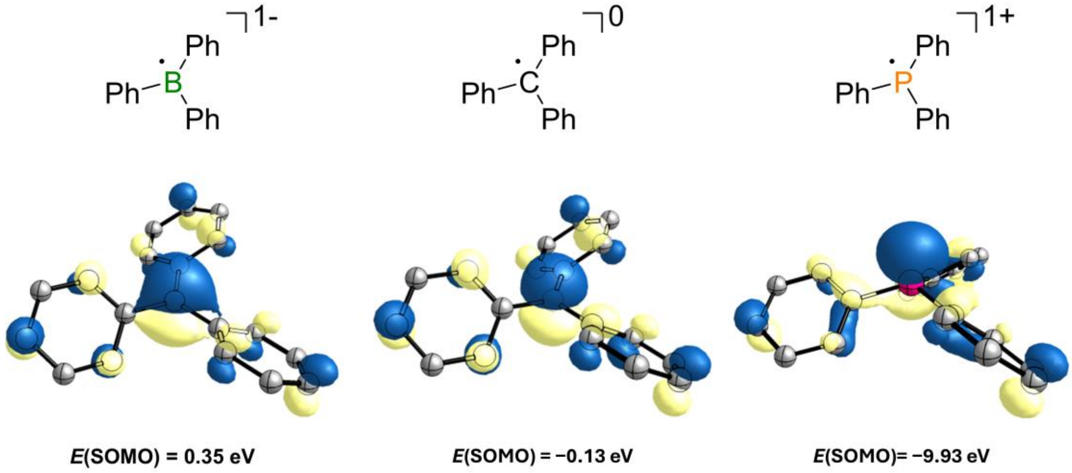

Figure S122. SOMOs of [BPh$_3$]$^-$, [CPh$_3$]^0, [PPh$_3$]$^+$ according to the PBE0-D3/def2-TZVP//PBE0-D3/def2-SVP level of theory; isovalue=0.05.

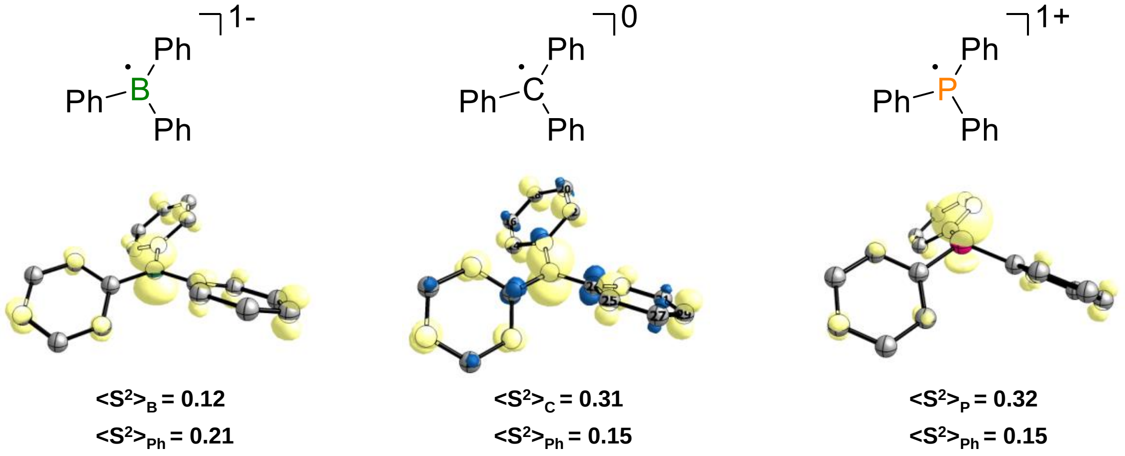

Figure S123. Spin densities and Local Spin Analysis (LSA) of $[BPh_3]^-$, $[CPh_3]^0$, $[PPh_3]^+$ according to the PBE0-D3/def2-TZVP//PBE0-D3/def2-SVP level of theory; isovalue=0.007.

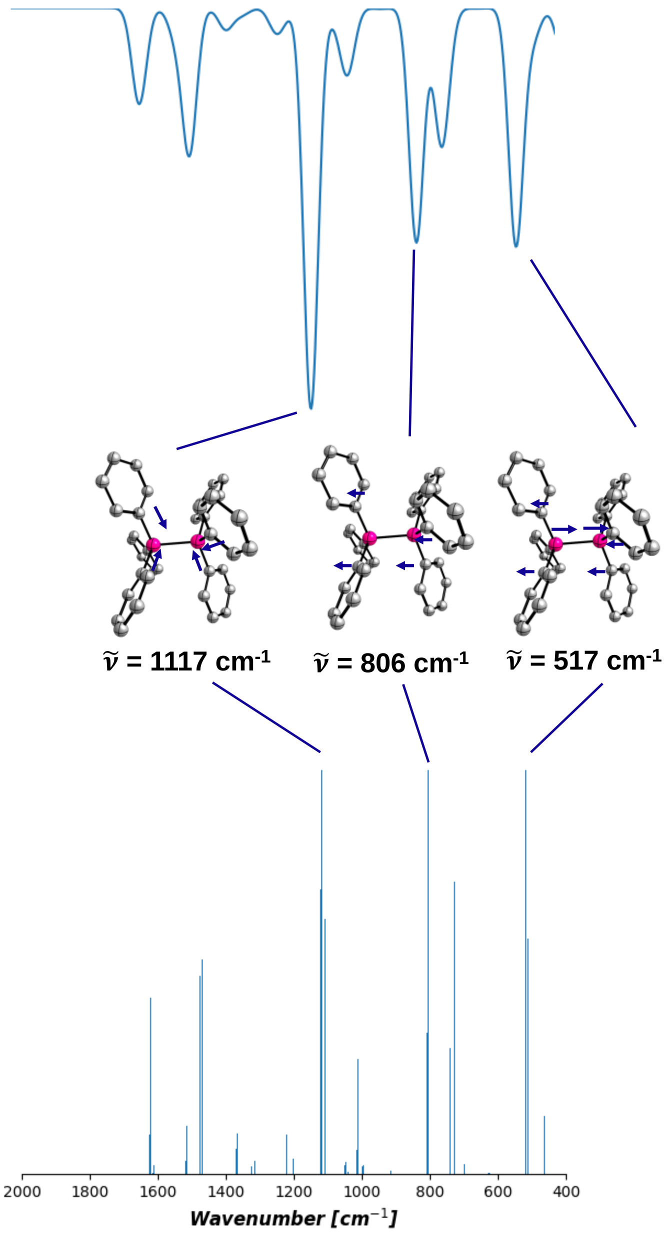

Figure S124. IR spectrum as obtained by the PBE0-D3/def2-SVP level of theory. The gaussian line broadening was applied with a bandwidth of 50.

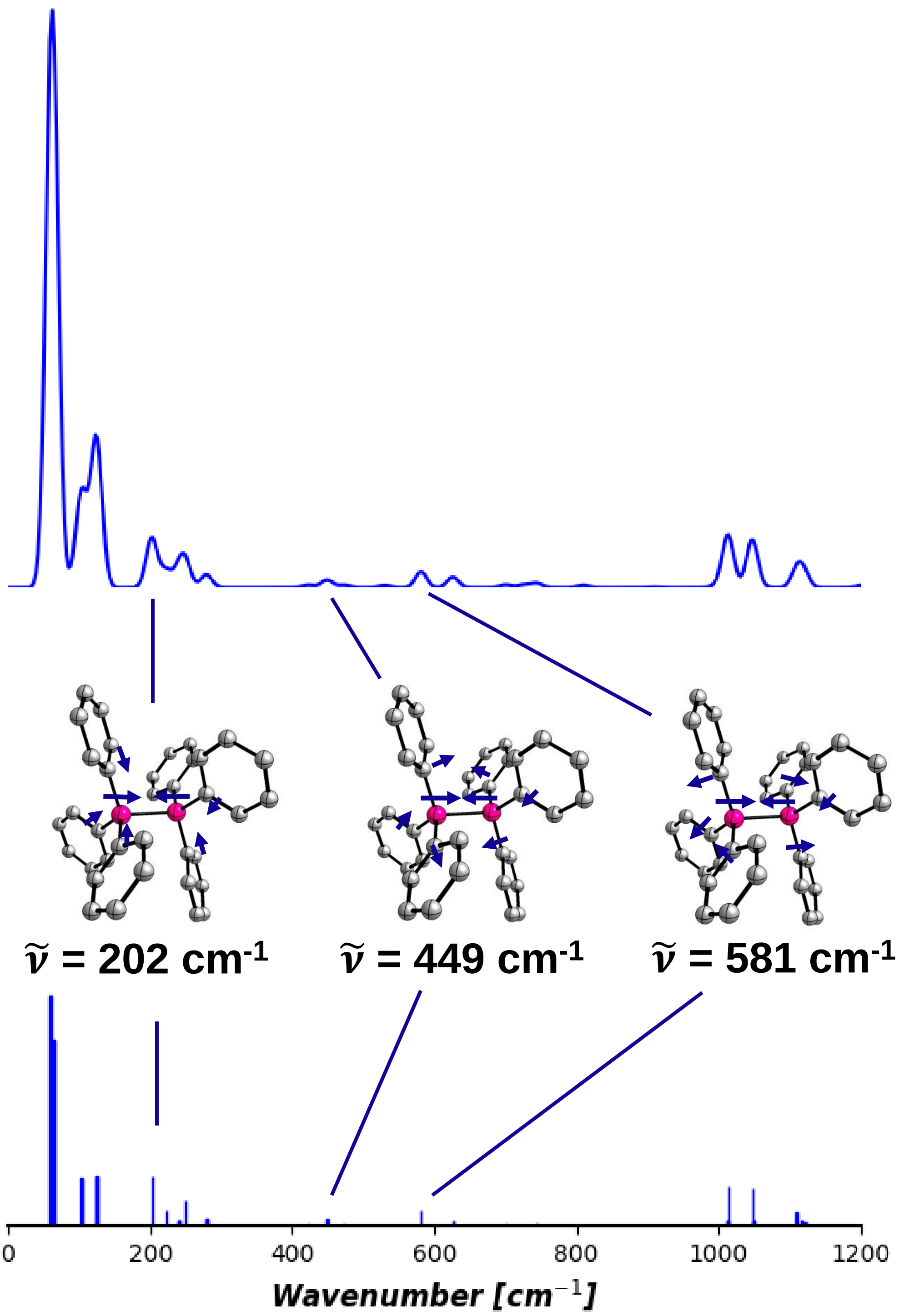

Figure S125. Raman spectrum of $[Ph_3P–PPh_3]^{2+}$ with the assignment of the coupled P–P stretches as predicted by the PBE0-D3/def2-SVP level of theory. The gaussian line broadening was applied with a bandwidth of 19.

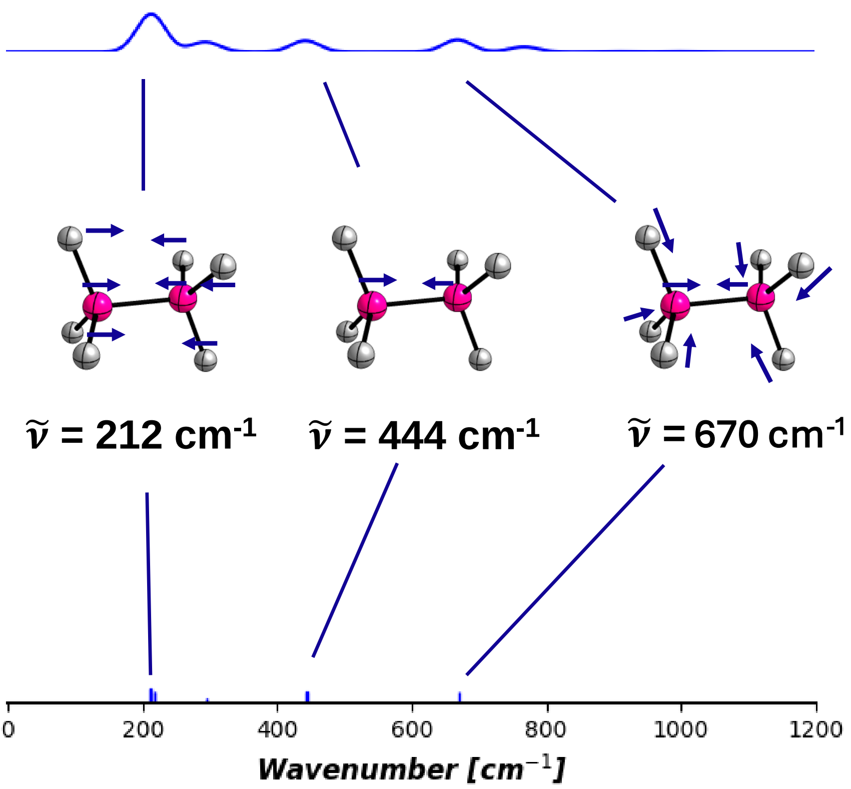

Figure S126. Raman spectrum of $[Me_3P–PMe_3]^{2+}$ with the assignment of the coupled P–P stretches as predicted by the PBE0-D3/def2-SVP level of theory. The gaussian line broadening was applied with a bandwidth of 50.

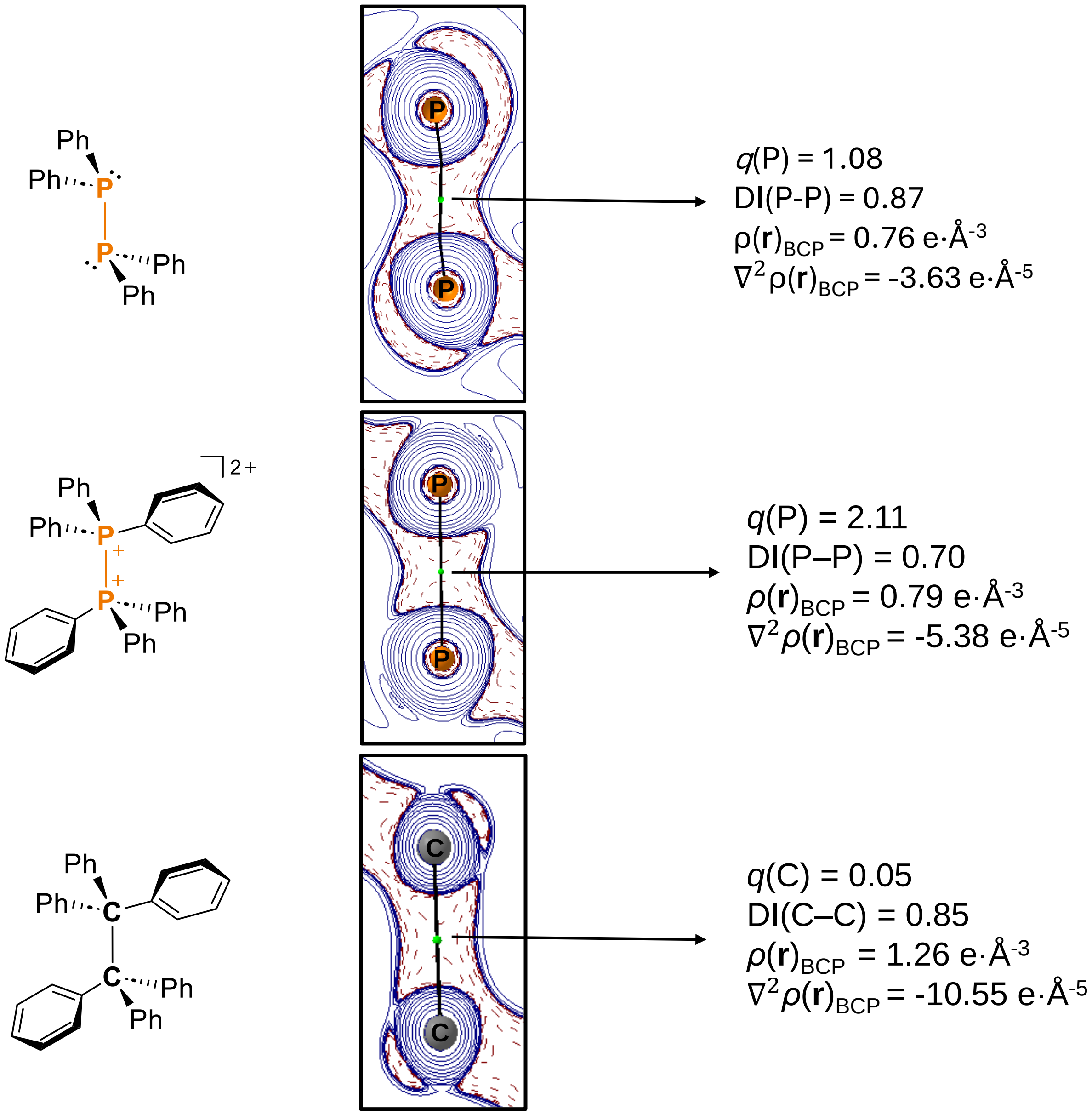

Figure S127. Representation of the 2D of the Laplacian distribution($\nabla^2\rho(\mathbf{r})_{BCP}$) of the P–P and C–C bonds as obtained at the PBE0-D3/def2-TZVPP//PBE0-D3/def2-SVP level of theory. The dashed red lines indicate charge concentration ($\nabla^2\rho(\mathbf{r})_{BCP}<0$), while solid blue lines indicate charge depletion ($\nabla^2\rho(\mathbf{r})_{BCP}>0$). The bond paths are depicted with solid black lines, and the Bond Critical Points (BCP) is designated with a green dot.

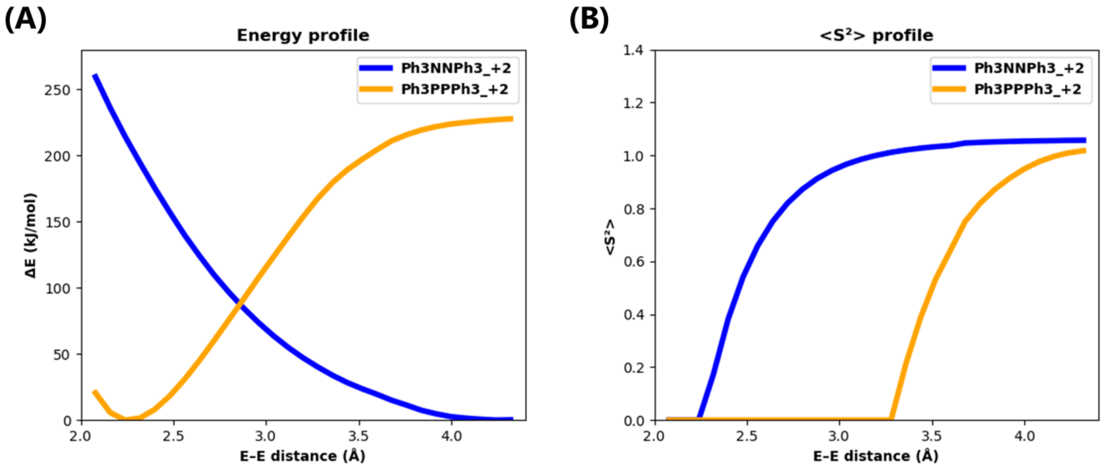

Figure S128. **(A)** Energy profiles along the E–E bond in $[Ph_3E–EPh_3]^{2+}$(E = N, P) at the CPCM(FBz)-PBE0-D3(BJ)/def2-TZVPP//PBE0-D3(BJ)/def2-SVP level of theory. **(B)** $\langle S^2 \rangle$ profiles along the E–E bond in $[Ph_3E–EPh_3]^{2+}$(E = N, P) at the CPCM(FBz)-PBE0-D3(BJ)/def2-TZVPP//PBE0-D3(BJ)/def2-SVP level of theory.

Table S5. Energy Decomposition Analysis in combination with Natural Orbitals for Chemical Valence (EDA-NOCV) of compounds **$[1]^{2+}$**, **$[Me_3P–PMe_3]^{2+}$**, **I $Ph_3C–CPh_3$**, **$[Ph_3B–BPh_3]^{2-}$**, **$Ph_2P–PPh_2$** at the ZORA-PBE0-D3/TZ2P//PBE0-D3/def2-SVP level of theory. The fragments are considered open-shell (doublet). Energies are given in [kJ mol^{-1}]. The Δq values were extracted using the Hirshfeld Atom in Molecules definition. [a] The values in parentheses give the percentage contribution to the total attractive interactions ΔE_{elstat}+ ΔE_{Disp}+ ΔE_{Orb} + $\Delta E_{Orb\text{-}HF}$. [b] The values in parentheses give the percentage contribution to ΔE_{Orb}. [c] The ΔE_{elstat} is not accounted in the attractive interaction percentage since it is repulsive. [d] The homolytic fragmentation is considered.

	**$[1]^{2+}$**	**$[Me_3P–PMe_3]^{2+}$**	**I**	**$[Ph_3B–BPh_3]^{2-}$**	**$Ph_2P–PPh_2$**	**$[Ph_3P\text{-}PPh_2]^{+}$[d]**
$-D_e$	–87.3	–19.1	–49.3	+116.0	–225.6	–308.9
ΔE_{Prep}	+34.3	+22.9	+328.9	+166.6	+43.1	41.1
ΔE_{int}	–121.6	–42.0	–378.2	–50.6	–268.7	–350.0
ΔE_{Pauli}	792.0	543.2	1213.4	586.1	846.8	846.3
ΔE_{Elstat}[a]	–206.4 (23%)	+7.4 (0%)[c]	–664.9 (42%)	–146.2 (23%)	–503.1 (45%)	–488.3 (41%)
ΔE_{Disp}[a]	–79.9 (9%)	–26.8 (4%)	–80.6 (5%)	–79.6 (5%)	–45.1 (4%)	–62.4 (5%)
ΔE_{Orb}[a]	–627.7	–566.6	–846.5	–411.1	–567.3	–645.7
$\Delta E_{Orb\text{-}HF}$	+0.3	+0.7	+0.3	+0.2	+0.0	0.2
$\Delta E_{Orb\text{-}corr}$[a]	–627.4 (69%)	–566.0 (94%)	–846.1 (53%)	–410.9 (26%)	–567.2 (51%)	–645.5 (54%)
$\Delta E_{Orb\text{-}\sigma}$[b]	–482.9 (77%)	–486.3 (86%)	–701.5 (83%)	–331.1 (81%)	–464.8 (82%)	–505.5 (78%)
$\Delta E_{Orb\text{-}rest}$[b]	–144.5 (23%)	–79.6 (14%)	–144.6 (17%)	–79.8 (19%)	–102.5 (18%)	–140.0 (22%)
$\Delta q(\sigma)$	0.82	0.71	0.79	0.78	0.81	0.80

Table S6. Deformation densities associated with the σ-bonding channel of **[1]$^{2+}$** (isovalue 0.003) as obtained at the ZORA-PBE0-D3/TZ2P//PBE0-D3/def2-SVP level of theory. The associated energies ΔE_{orb} are given in [kJ mol^{-1}], and ν in [a.u.]. The red color designates charge density depletion, while blue shows charge density density accumulation. The shape of the most important interacting occupied and vacant orbitals of the fragments are depicted (isovalue 0.05 a.u.). Hydrogen atoms are omitted for clarity.

**Deformation density**	**$[Ph_3P]^+$ (doublet)**	**$[Ph_3P]^+$ (doublet)**
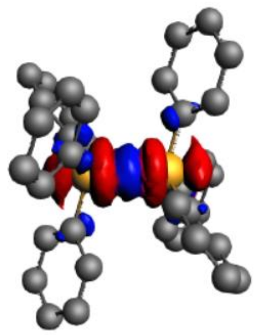	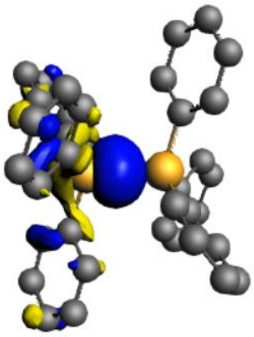	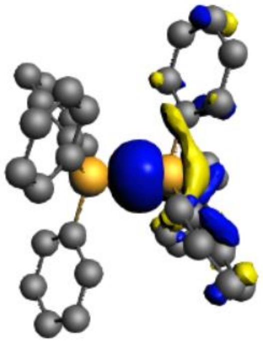
$\Delta E_{orb\text{-}\sigma}$ = −482.9; $\lvert\nu_{\alpha\beta}\rvert$=1.3	ν_α = −0.55; ν_β=+0.55<br>*E*(SOMO) = −9.11 eV	ν_α=+0.55 ; ν_β = −0.55<br>*E*(SOMO) = −9.11 eV

Table S7. Deformation densities associated with the σ- bonding channel of **$[Me_3P–PMe_3]^{2+}$** (isovalue 0.003) as obtained at the ZORA-PBE0-D3/TZ2P//PBE0-D3/def2-SVP level of theory. The associated energies ΔE_{orb} are given in [kJ mol^{-1}], and ν in [a.u.]. The red color designates charge density depletion, while blue shows charge density accumulation. The shape of the most important interacting occupied and vacant orbitals of the fragments are depicted (isovalue 0.05 a.u.). Hydrogen atoms are omitted for clarity.

**Deformation density**	**$[Me_3P]^+$ (doublet)**	**$[Me_3P]^+$ (doublet)**
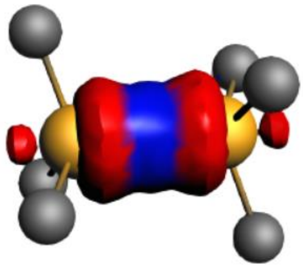	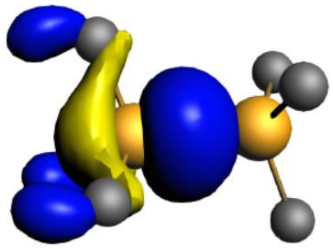	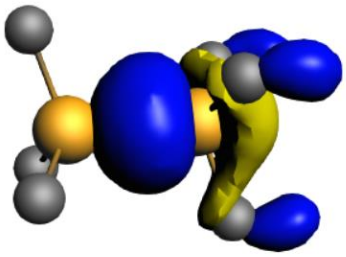
$\Delta E_{orb\text{-}\sigma}$ = −486.3; $\vert\upsilon_{\alpha\beta}\vert$=1.1	ν_α = −0.54; ν_β=+0.54<br>*E*(SOMO) = −11.04 eV	ν_α=+0.54 ; ν_β=−0.54<br>*E*(SOMO) = −11.04 eV

Table S8. Deformation densities associated with the σ- bonding channel of **I $Ph_3C–CPh_3$** (isovalue 0.003 a.u.) as obtained at the ZORA-PBE0-D3/TZ2P//PBE0-D3/def2-SVP level of theory. The associated energies ΔE_{orb} are given in [kJ mol^{-1}] and ν in a.u.. The red color designates charge density depletion, while blue shows charge density accumulation. The shape of the most important interacting occupied and vacant orbitals of the fragments are depicted (isovalue 0.05 a.u.). Hydrogen atoms are omitted for clarity.

**Deformation density**	**$[Ph_3C]$ (doublet)**	**$[Ph_3C]$ (doublet)**
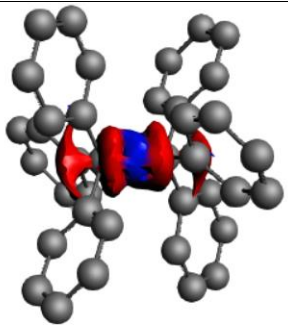	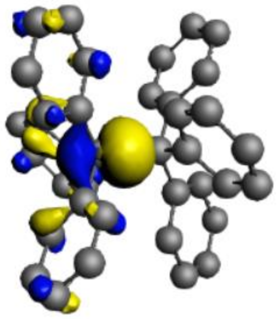	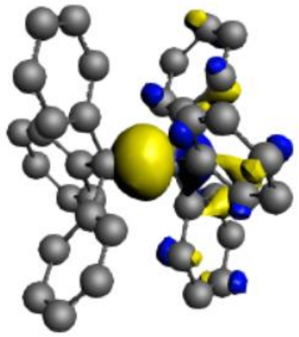
$\Delta E_{orb\text{-}\sigma}$= −701.5; $\vert\upsilon_{\alpha\beta}\vert$=1.3	ν_α=-0.55; ν_β=+0.55<br>*E*(SOMO) =−4.58 eV	ν_α=+0.55 ; ν_β= −0.55<br>*E*(SOMO) = −4.58 eV

Table S9. Deformation densities associated with the σ- bonding channel of compound **$[Ph_3B–BPh_3]^{2-}$** (isovalue 0.003 a.u.) as obtained at the ZORA-PBE0-D3/TZ2P//PBE0-D3/def2-SVP level of theory. The associated energies ΔE_{orb} are given in [kJ mol^{-1}] and ν in a.u.. The red color designates charge density depletion, while blue shows charge density accumulation. The shape of the most important interacting occupied and vacant orbitals of the fragments are depicted (isovalue 0.05 a.u.). Hydrogen atoms are omitted for clarity.

**Deformation density**	**$[Ph_3B]^-$ (doublet)**	**$[Ph_3B]^-$ (doublet)**
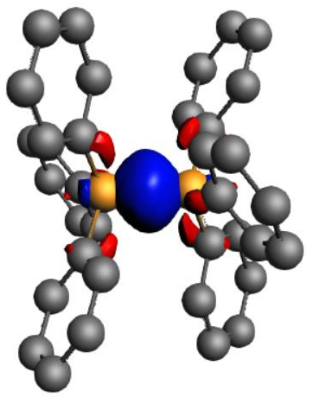	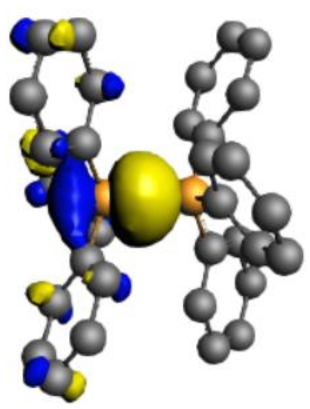	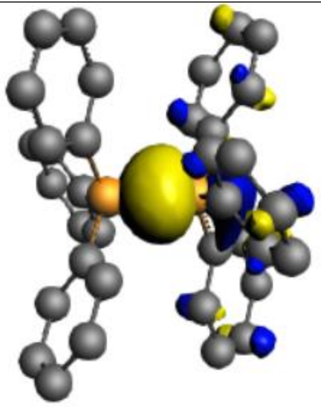
$\Delta E_{orb\text{-}\sigma}$= −331.1; $\lvert\upsilon_{\alpha\beta}\rvert$=1.24	ν_α=-0.51 ; ν_β=+0.51<br>E(SOMO) = 0.85 eV	ν_α=+0.51 ; ν_β= −0.51<br>SOMO=0.85 eV

Table S10. Deformation densities associated with the σ-bonding channel of compound $Ph_2P–PPh_2$ (isovalue 0.003 a.u.) as obtained at the ZORA-PBE0-D3/TZ2P//PBE0-D3/def2-SVP level of theory. The associated energies ΔE_{orb} are given in [kJ mol^{-1}] and ν in a.u.. The red color designates charge density depletion, while blue shows charge density accumulation. The shape of the most important interacting occupied and vacant orbitals of the fragments are depicted (isovalue 0.05 a.u.). Hydrogen atoms are omitted for clarity.

**Deformation density**	**Ph_2P (doublet)**	**Ph_2P (doublet)**
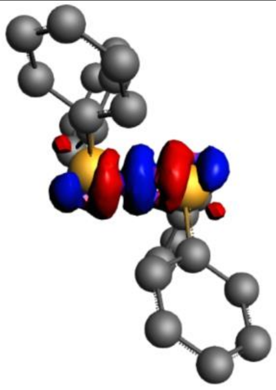	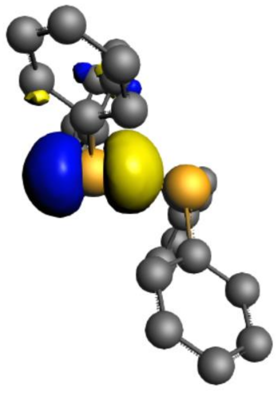	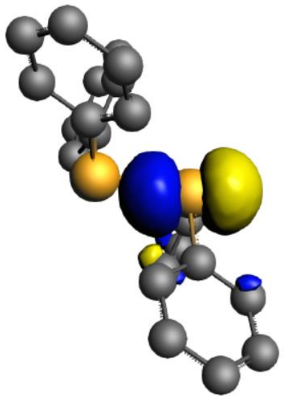
$\Delta E_{orb\text{-}\sigma}$ = −464.8; $\lvert\upsilon_{\alpha\beta}\rvert$ = 1.3	ν_α = −0.65 ; ν_β= +0.65<br>E(SOMO) = −4.45 eV	ν_α =+0.65 ; ν_β = −0.65<br>E(SOMO) = −4.45 eV

Table S11. NOCV decomposition of the orbital interaction energy in **[1]$^{2+}$** and [Me_3P–PMe_3]$^{2+}$ gives the following contributions (collective secondary orbital contributions, respectively) in [kJ mol^{-1}].

NOCV #	**R=Ph**	**R=Me**
1	-482.9	-486.8
2	-34.6	-8.2
3	-24.9	-24.0
4	-24.8	-24.0
5	-7.4	-3.7
6	-7.4	-3.7
7	-6.2	-2.8
8	-4.2	-2.3
9	-4.4	-2.3
10	-4.4	-1.3
11	-2.5	-1.5
12	-2.5	-0.5
13	-2.4	-0.5
14	-1.6	-1.0
15	-1.2	-0.6
16	-1.2	-0.6
17	-1.1	-0.6
18	-1.1	-0.6
19	-0.8	-0.4

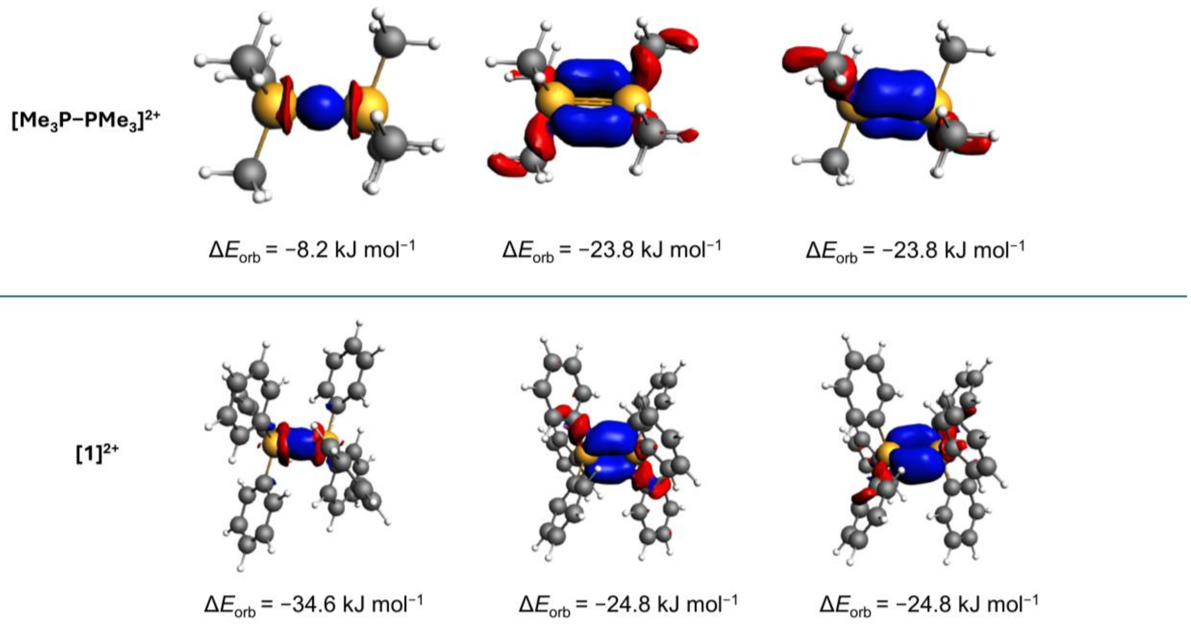

Figure S129. Deformation densities of the NOCV bondings channels #2, #3 and #4 as well as their orbital interaction energies (collective secondary orbital contributions, respectively) of **[1]$^{2+}$** and [Me_3P-PMe_3]$^{2+}$.

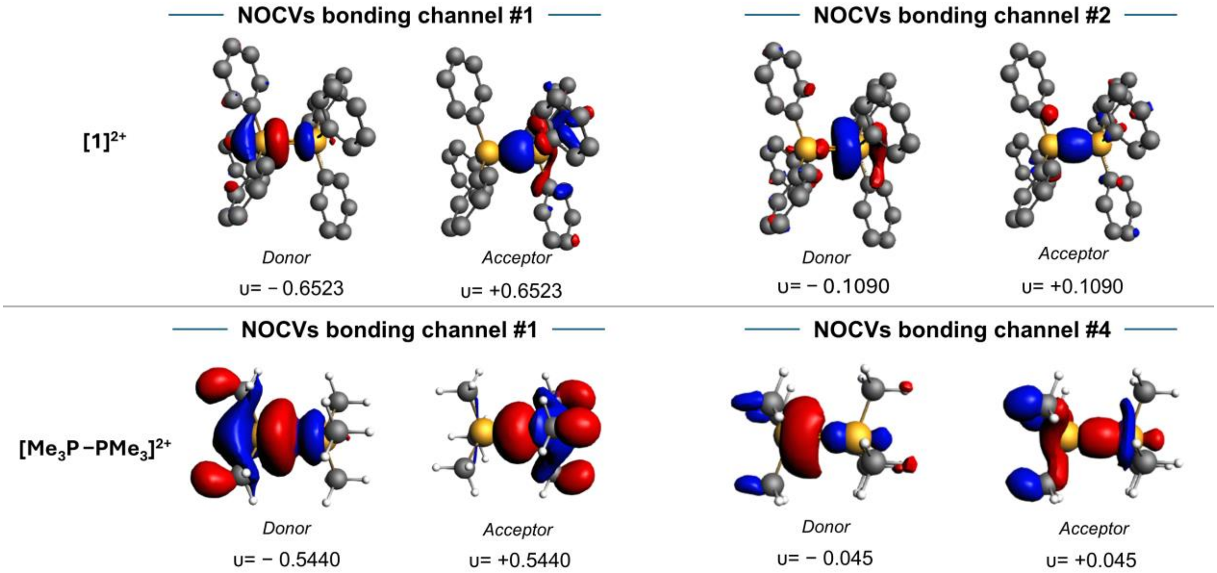

Figure S130. The donor and acceptor α-NOCV orbitals with their corresponding electron flows (eigenvalues υ) of the σ-deformation densities **[1]$^{2+}$** (top) as well as [$Me_3P–PMe_3$]$^{2+}$ (bottom).

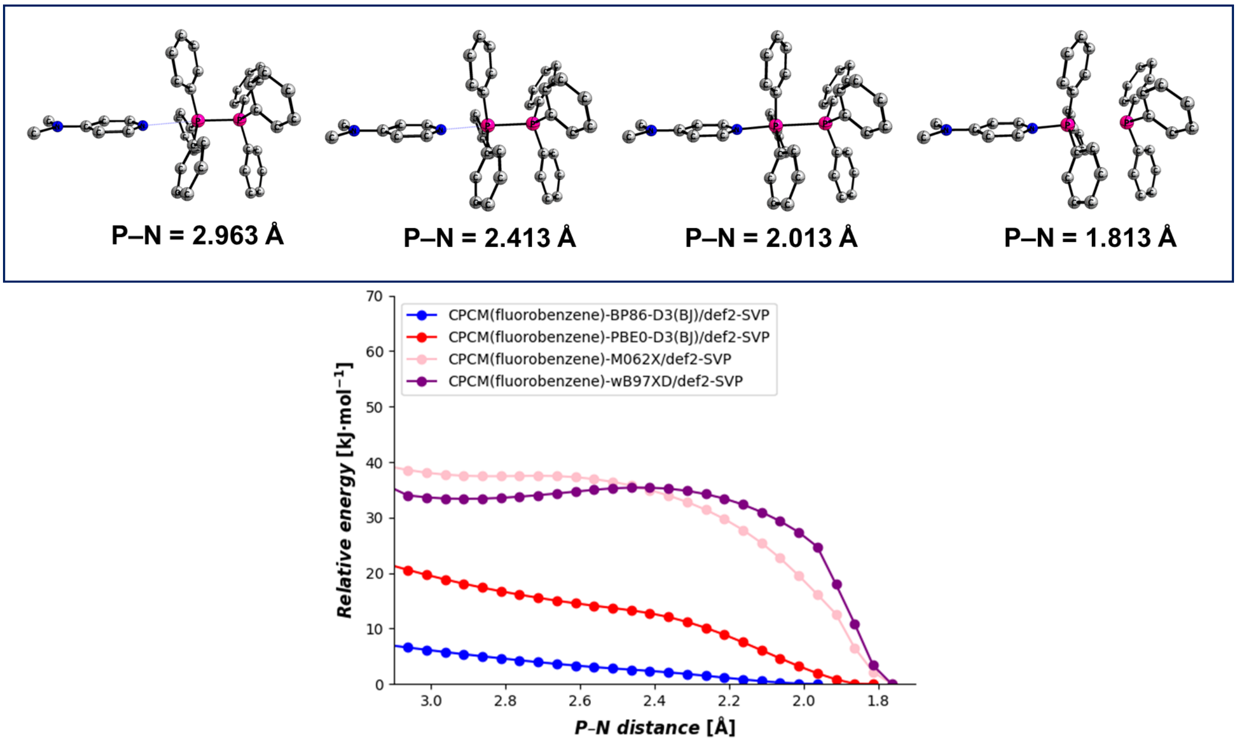

Figure S131. Scans of the P–N bond illustrating barrierless replacement of PPh_3 by DMAP. The scans were performed using different density functional approximations (BP86-D3(BJ), PBE0-D3(BJ), M062X, ωB97XD) with the def2-SVP basis set. The optimizations include implicit solvation effects in fluorobenzene (CPCM). On top of the image, snapshots of geometries along the scan are represented.

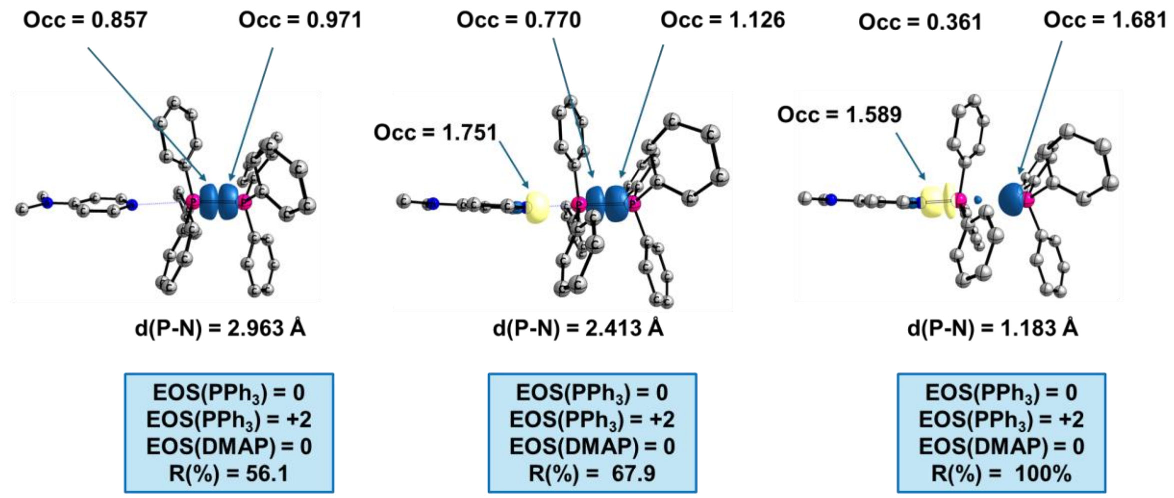

Figure S132. EOS analysis, σ-type EFOs and its corresponding net occupations at different snapshots of geometries along the reaction coordinates for the substitution of PPh_3 by DMAP as obtained at the CPCM(PhF)-PBE0-D3(BJ)/def2-SVP level of theory.

Table S12. *FIA* and *HIA* values of **[1]$^{2+}$**, **[PPh$_3$]$^{2+}$** and Stephan's bridged diphosphonium dication[41] computed at the DLPNO-CCSD(T)/cc-pVQZ//PBE0-D3(BJ)/def2-SVP level of theory. The Me_3SiF and Me_3SiH are used as anchor molecules. (ΔH($Me_3SiF \rightarrow Me_3Si^+ + F^-$) = 952.5 kJ mol^{-1} ; ΔH($Me_3SiH \rightarrow Me_3Si^+ + H^-$) = 924 kJ · mol^{-1}). The *FIA/HIA* values are given in [kJ mol^{-1}], and either referenced versus $[PPh_3]^{2+}$ or **[1]$^{2+}$**. [a] $[Compound]^q + Me_3SiF \rightarrow Me_3Si^+ + [Compound\text{-}F]^{q-1}$; [b] $[Compound]^q + Me_3SiH \rightarrow Me_3Si^+ + [Compound\text{-}H]^{q-1}$.[42]

	FIA	*FIA*$_{solv}$	$\Delta G^{[a]}$	*HIA*	*HIA*$_{solv}$	$\Delta G^{[b]}$
**[PPh$_3$]$^{2+}$**	1237	511	67	1318	574	–44
**[1]$^{2+}$**	807	196	–297	888	260	–409
**Stephan's diphosphonium dication**	855	193	88	950	597	–44

Table S13. Conceptual DFT indices[43] for **[1]$^{2+}$** and Stephan's bridged diphosphonium dication[41] as obtained at the triple-ζ level of theory. ε_{HOMO}, highest occupied molecular orbital energy; ε_{LUMO}, lowest unoccupied molecular orbital energy; μ, chemical potential; η, chemical hardness; ω, global electrophilicity index.[44]

Method	Compound	ε_{HOMO} [eV]	ε_{LUMO} [eV]	μ [eV]	η [eV]	ω [eV]
PBE0-D3(CPCM=PhF)	**[1]$^{2+}$**	-9.0	-3.9	-6.4	5.1	**4.1**
	**[Stephan's]$^{2+}$**	-8.5	-3.8	-6.1	4.7	**4.0**
MP2(CPCM=PhF)	**[1]$^{2+}$**	-10.8	-0.5	-5.6	10.3	**1.6**
	**[Stephan's]$^{2+}$**	-10.1	-0.3	-5.2	9.7	**1.4**

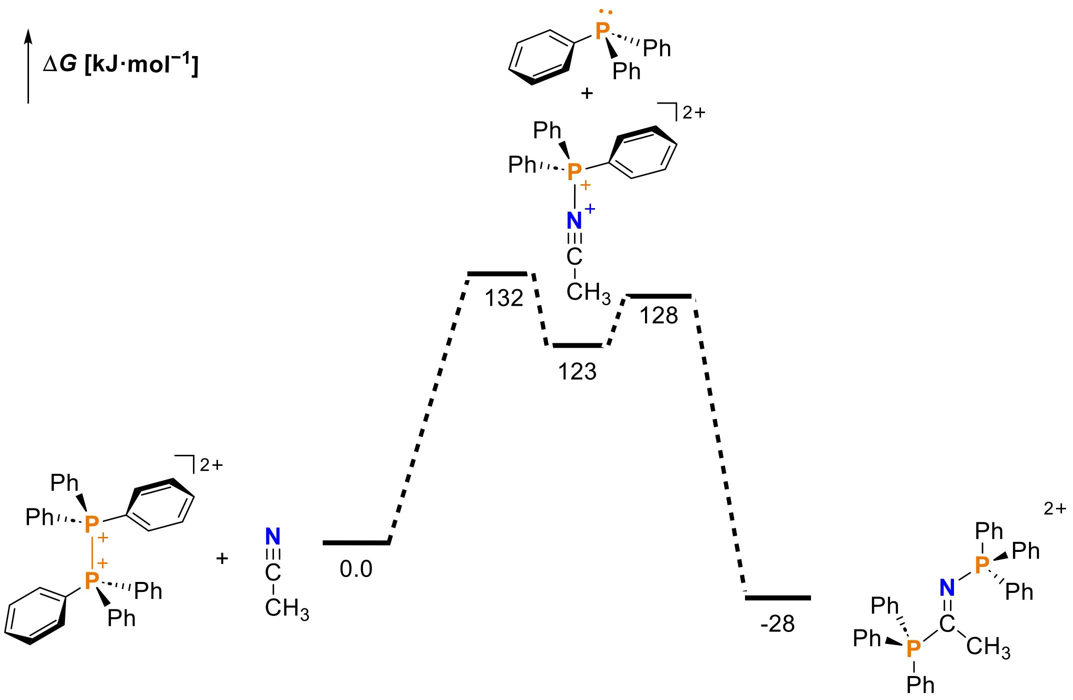

Figure S133. Gibbs Free Energy profile for the addition of **[1]**$^{2+}$ to MeCN according to the PBE0-D3(CPCM=Acetonitrile)/def2-TZVPP//PBE0-D3/def2-SVP level of theory. The Gibbs free energies are Standard-State and Entropy corrected.

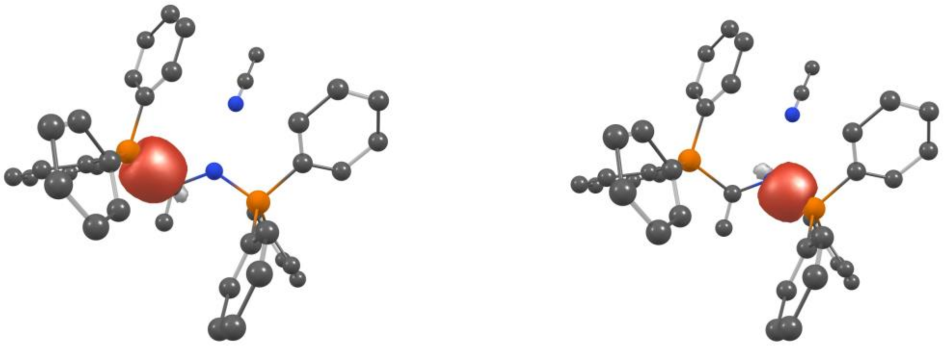

C : P = 0.5 : 0.5 (Löwdin) N : P = 0.6 : 0.4 (Löwdin)

$q_{P(C)}$ = +1.0 a.u. $q_{P(N)}$ = +0.9 a.u.

Figure S134. Population analysis of pertinent IBOs of addition product ^{Me}**5**$^{2+}$ (PBE0-D4/def2-TZVPP//PBE0-D3/def2-SVP).

8 References

1. Romanato, P.; Duttwyler, S.; Linden, A.; Baldridge, K. K.; Siegel, J. S. Intramolecular Halogen Stabilization of Silylium Ions Directs Gearing Dynamics. *J. Am. Chem. Soc.* **2010**, *132*, 7828-7829.
2. Hwang, S. J.; Powers, D. C.; Maher, A. G.; Nocera, D. G. Tandem redox mediator/Ni(ii) trihalide complex photocycle for hydrogen evolution from HCl. *Chem. Sci.* **2015**, *6*, 917-922.
3. Schorpp, M.; Heizmann, T.; Schmucker, M.; Rein, S.; Weber, S.; Krossing, I. Synthesis and Application of a Perfluorinated Ammoniumyl Radical Cation as a Very Strong Deelectronator. *Angew. Chem. Int. Ed.* **2020**, *59*, 9453-9459.
4. Everett, J. P.; Schmidt, D. L.; Rose, G. D.; Argritis, P.; Aidinis, C. J.; Hatzakis, M. Synthesis of some onium salts and their comparison as cationic photoinitiators in an epoxy resist. *Polymer* **1997**, *38*, 1719-1723.
5. Connelly, N. G.; Geiger, W. E. Chemical redox agents for organometallic chemistry. *Chem. Rev.* **1996**, *96*, 877-910.
6. Bard, A. J. F., L. R.; White, H. S., *Electrochemical Methods: Fundamentals and Applications*. John Wiley & Sons,Hoboken, NJ, USA, 2022.
7. Raabe, I.; Wagner, K.; Guttsche, K.; Wang, M.; Grätzel, M.; Santiso-Quiñones, G.; Krossing, I. Tetraalkylammonium Salts of Weakly Coordinating Aluminates: Ionic Liquids, Materials for Electrochemical Applications and Useful Compounds for Anion Investigation. *Chem. Eur. J.* **2009**, *15*, 1966-1976.
8. Sellin, M.; Friedmann, C.; Mayländer, M.; Richert, S.; Krossing, I. Towards clustered carbonyl cations $[M_3(CO)_{14}]^{2+}$ (M = Ru, Os): the need for innocent deelectronation. *Chem. Sci.* **2022**, *13*, 9147-9158.
9. (a) Rabideau, B. D.; Soltani, M.; Parker, R. A.; Siu, B.; Salter, E. A.; Wierzbicki, A.; West, K. N.; Davis, J. H. Tuning the melting point of selected ionic liquids through adjustment of the cation's dipole moment. *Phys. Chem. Chem. Phys.* **2020**, *22*, 12301-12311. (b) Xu, J.; Cao, J.; Wu, X.; Wang, H.; Yang, X.; Tang, X.; Toh, R. W.; Zhou, R.; Yeow, E. K. L.; Wu, J. Unveiling Extreme Photoreduction Potentials of Donor–Acceptor Cyanoarenes to Access Aryl Radicals from Aryl Chlorides. *J. Am. Chem. Soc.* **2021**, *143*, 13266-13273. (c) Zhivetyeva, S. I.; Goryunov, L. I.; Bagryanskaya, I. Y.; Grobe, J.; Shteingarts, V. D.; Würthwein, E. U. Phosphinodefluorination of polyfluorobenzenes by silylphosphines Ph(R)PSiMe$_3$ (R=Me, Ph): Further experimental and computational evidences for the concerted ANDN mechanism of aromatic nucleophilic substitution. *J. Fluor. Chem.* **2014**, *164*, 58-69. (d) Park, S.; Pontier-Johnson, M.; Roundhill, D. M. Regioselective carbon-fluorine bond cleavage reactions from the interaction of transition metal fluorocarbon complexes with nucleophiles. *Inorg. Chem.* **1990**, *29*, 2689-2697.

10. Krossing, I. The Facile Preparation of Weakly Coordinating Anions: Structure and Characterisation of Silverpolyfluoroalkoxyaluminates AgAl(ORF)4, Calculation of the Alkoxide Ion Affinity. *Chem. Eur. J.* **2001**, *7*, 490-502.
11. Chitnis, S. S.; Robertson, A. P. M.; Burford, N.; Weigand, J. J.; Fischer, R. Synthesis and reactivity of cyclo-tetra(stibinophosphonium) tetracations: redox and coordination chemistry of phosphine–antimony complexes. *Chem. Sci.* **2015**, *6*, 2559-2574.
12. Moussa, Z.; Ahmed, S. A.; ElDouhaibi, A. S.; Al-Raqa, S. Y. NMR Studies and electrophilic properties of triphenylphosphine–trifluoromethanesulfonic anhydride; a remarkable dehydrating reagent system for the conversion of aldoximes into nitriles. *Tetrahedron Lett.* **2010**, *51*, 1826-1831.
13. Yang, Z.; Chen, S.; Yang, F.; Zhang, C.; Dou, Y.; Zhou, Q.; Yan, Y.; Tang, L. PPh_3/Selectfluor-Mediated Transformation of Carboxylic Acids into Acid Anhydrides and Acyl Fluorides and Its Application in Amide and Ester Synthesis. *Eur. J. Org. Chem.* **2019**, *2019*, 5998-6002.
14. Dyke, J. M.; Emsley, J. W.; Greenacre, V. K.; Levason, W.; Monzittu, F. M.; Reid, G.; De Luca, G. Tertiary Phosphine and Arsine Complexes of Phosphorus Pentafluoride: Synthesis, Properties, and Electronic Structures. *Inorg. Chem.* **2020**, *59*, 4517-4526.
15. (a) Arduengo Iii, A. J.; Davidson, F.; Krafczyk, R.; Marshall, W. J.; Schmutzler, R. Carbene Complexes of Pnictogen Pentafluorides and Boron Trifluoride. *Monatshefte für Chemie / Chemical Monthly* **2000**, *131*, 251-265. (b) Rowsey, R. A.; Hilgar, J. D.; Romero, N. A. Silylimidazolium Hexafluorophosphate Salts as Synthetic Precursors to N-Heterocyclic Carbene Pentafluorophosphorus Adducts. *Org. Lett.* **2024**, *26*, 4750-4755.
16. Wang, W.; Zhu, H.; Feng, L.; Yu, Q.; Hao, J.; Zhu, R.; Wang, Y. Dual Chalcogen–Chalcogen Bonding Catalysis. *J. Am. Chem. Soc.* **2020**, *142*, 3117-3124.
17. (a) Mörsdorf, J.-M.; Ballmann, J. Coordination-Induced Radical Generation: Selective Hydrogen Atom Abstraction via Controlled Ti–C σ-Bond Homolysis. *J. Am. Chem. Soc.* **2023**, *145*, 23452-23460. (b) Li, P.-C.; Wang, T.-S.; Lee, G.-H.; Liu, Y.-H.; Wang, Y.; Chen, C.-T.; Chao, I. Theoretical Study and X-ray Determination of Bianthrones: Long C−C Bond Length and Preferred Gauche Conformation. *J. Org. Chem.* **2002**, *67*, 8002-8009.
18. Sheldrick, G. SHELXT - Integrated space-group and crystal-structure determination. *Acta Crystallogr A* **2015**, *71*, 3-8.
19. Sheldrick, G. M. Crystal structure refinement with SHELXL. *Acta Crystallogr. C Struct. Chem.* **2015**, *71*, 3-8.
20. (a) Dolomanov, O. V.; Bourhis, L. J.; Gildea, R. J.; Howard, J. A. K.; Puschmann, H. OLEX2: a complete structure solution, refinement and analysis program. *J. Appl. Crystallogr.* **2009**, *42*, 339-341. (b) Hubschle, C. B.; Sheldrick, G. M.; Dittrich, B. ShelXle: a Qt graphical user interface for SHELXL. *J. App. Crystallogr.* **2011**, *44*, 1281-1284.
21. *Bruker (2012). APEX3/4. Bruker AXS Inc., Madison, Wisconsin, USA.* .

22. *Oxford Diffraction (2018). CrysAlisPro (Version 1.171.40.37a). Oxford Diffraction Ltd., Yarnton, Oxfordshire, UK.*
23. *K. Brandenburg, DIAMOND, V. 4.6.8. 2022.*
24. M. J. Frisch, G. W. T., H. B. Schlegel, G. E. Scuseria, M. A. Robb, J. R. Cheeseman, G. Scalmani, V. Barone, G. A. Petersson, H. Nakatsuji, X. Li, M. Caricato, A. V. Marenich, J. Bloino, B. G. Janesko, R. Gomperts, B. Mennucci, H. P. Hratchian, J. V. Ortiz, A. F. Izmaylov, J. L. Sonnenberg, D. Williams-Young, F. Ding, F. Lipparini, F. Egidi, J. Goings, B. Peng, A. Petrone, T. Henderson, D. Ranasinghe, V. G. Zakrzewski, J. Gao, N. Rega, G. Zheng, W. Liang, M. Hada, M. Ehara, K. Toyota, R. Fukuda, J. Hasegawa, M. Ishida, T. Nakajima, Y. Honda, O. Kitao, H. Nakai, T. Vreven, K. Throssell, J. A. Montgomery, Jr., J. E. Peralta, F. Ogliaro, M. J. Bearpark, J. J. Heyd, E. N. Brothers, K. N. Kudin, V. N. Staroverov, T. A. Keith, R. Kobayashi, J. Normand, K. Raghavachari, A. P. Rendell, J. C. Burant, S. S. Iyengar, J. Tomasi, M. Cossi, J. M. Millam, M. Klene, C. Adamo, R. Cammi, J. W. Ochterski, R. L. Martin, K. Morokuma, O. Farkas, J. B. Foresman, and D. J. Fox, *Gaussian 16, Revision C.0.1*, Gaussian, Inc.: Wallingford CT, 2019.
25. Becke, A. D. Density-functional exchange-energy approximation with correct asymptotic behavior. *Phys. Rev. A* **1988**, *38*, 3098-3100.
26. Lee, C. T.; Yang, W. T.; Parr, R. G. Development of the Colle-Salvetti Correlation-Energy Formula into a Functional of the Electron-Density. *Phys. Rev. B: Condens. Matter* **1988**, *37*, 785-789.
27. (a) Perdew, J. P.; Ernzerhof, M.; Burke, K. Rationale for mixing exact exchange with density functional approximations. *J. Chem. Phys.* **1996**, *105*, 9982-9985. (b) Adamo, C.; Barone, V. Toward reliable density functional methods without adjustable parameters: the PBE0 model. *J. Chem. Phys.* **1999**, *110*, 6158-6170.
28. Zhao, Y.; Truhlar, D. G. The M06 suite of density functionals for main group thermochemistry, thermochemical kinetics, noncovalent interactions, excited states, and transition elements: two new functionals and systematic testing of four M06-class functionals and 12 other functionals. *Theor. Chem. Acc.* **2008**, *120*, 215-241.
29. (a) Chai, J. D.; Head-Gordon, M. Systematic optimization of long-range corrected hybrid density functionals. *J. Chem. Phys.* **2008**, *128*, 084106. (b) Caldeweyher, E.; Ehlert, S.; Hansen, A.; Neugebauer, H.; Spicher, S.; Bannwarth, C.; Grimme, S. A generally applicable atomic-charge dependent London dispersion correction. *J. Chem. Phys.* **2019**, *150*, 154122.
30. Grimme, S.; Antony, J.; Ehrlich, S.; Krieg, H. A consistent and accurate ab initio parametrization of density functional dispersion correction (DFT-D) for the 94 elements H-Pu. *J. Chem. Phys.* **2010**, *132*, 154104.
31. Barone, V.; Cossi, M. Quantum Calculation of Molecular Energies and Energy Gradients in Solution by a Conductor Solvent Model. *J. Chem. Phys. A* **1998**, *102*, 1995-2001.
32. Cramer, C. J., *Essentials of Computational Chemistry: Theories and Models*. Wiley-VCH, Chichester, 2008; 2nd ed.

33. Sun, Q.; Berkelbach, T. C.; Blunt, N. S.; Booth, G. H.; Guo, S.; Li, Z.; Liu, J.; McClain, J. D.; Sayfutyarova, E. R.; Sharma, S.; Wouters, S.; Chan, G. K.-L. PySCF: the Python-based simulations of chemistry framework. *WIREs Comput. Mol. Sci.* **2018**, *8*, e1340.
34. te Velde, G.; Bickelhaupt, F. M.; Baerends, E. J.; Fonseca Guerra, C.; van Gisbergen, S. J. A.; Snijders, J. G.; Ziegler, T. Chemistry with ADF. *J. Comput. Chem.* **2001**, *22*, 931-967.
35. Van Lenthe, E.; Baerends, E. J. Optimized Slater-type basis sets for the elements 1–118. *J. Comput. Chem.* **2003**, *24*, 1142-1156.
36. Couzijn, E. P. A.; Lai, Y.-Y.; Limacher, A.; Chen, P. Intuitive Quantifiers of Charge Flows in Coordinate Bonding. *Organometallics* **2017**, *36*, 3205-3214.
37. Ramos-Cordoba, E.; Postils, V.; Salvador, P. Oxidation States from Wave Function Analysis. *J. Chem. Theory Comput.* **2015**, *11*, 1501-1508.
38. Salvador, P.; Ramos-Cordoba, E. Communication: An approximation to Bader's topological atom. *J. Chem. Phys.* **2013**, *139*, 071103
39. (a) Neese, F. Software update: The ORCA program system—Version 5.0. *WIREs Comput. Mol. Sci.* **2022**, *12*, e1606. (b) Neese, F. The ORCA program system. *Wiley Interdiscip. Rev.: Comput. Mol. Sci.* **2012**, *2*, 73-78.
40. Woon, D. E.; Dunning, T. H., Jr. Gaussian basis sets for use in correlated molecular calculations. III. The atoms aluminum through argon. *J. Chem. Phys.* **1993**, *98*, 1358-1371.
41. Holthausen, M. H.; Bayne, J. M.; Mallov, I.; Dobrovetsky, R.; Stephan, D. W. 1,2-Diphosphonium Dication: A Strong P-Based Lewis Acid in Frustrated Lewis Pair (FLP)-Activations of B–H, Si–H, C–H, and H–H Bonds. *J. Am. Chem. Soc.* **2015**, *137*, 7298-7301.
42. (a) Erdmann, P.; Schmitt, M.; Sigmund, L. M.; Krämer, F.; Breher, F.; Greb, L. How to Deal with Charge in the Ranking of Lewis Acidity: Critical Evaluation of an Extensive Set of Cationic Lewis Acids. *Angew. Chem. Int. Ed.* **2024**, *63*, e202403356. (b) Erdmann, P.; Greb, L. Multidimensional Lewis Acidity: A Consistent Data Set of Chloride, Hydride, Methide, Water and Ammonia Affinities for 183 p-Block Element Lewis Acids. *ChemPhysChem* **2021**, *22*, 935-943. (c) Erdmann, P.; Leitner, J.; Schwarz, J.; Greb, L. An Extensive Set of Accurate Fluoride Ion Affinities for p-Block Element Lewis Acids and Basic Design Principles for Strong Fluoride Ion Acceptors. *ChemPhysChem* **2020**, *21*, 987-994.
43. a) Geerlings, P.; De Proft, F.; Langenaeker, W. *Chem. Rev.* **2003**, *103*, 1793–1874; b) Chattaraj, P. K.; Sarkar, U.; Roy, D. R. Electrophilicity Index *Chem. Rev.* **2006**, *106*, 2065-2091.
44. (a) Jupp, A. R.; Johnstone, T. C.; Stephan, D. W. *Dalton Trans.* **2018**, *47*, 7029-7035. (b) Jupp, A. R.; Johnstone, T. C.; Stephan, D. W. *Inorg. Chem.* **2018**, *57*, 14764-14771.
